# High‐Throughput Computational Evaluation of Low Symmetry Pd_2_L_4_ Cages to Aid in System Design**

**DOI:** 10.1002/anie.202106721

**Published:** 2021-08-11

**Authors:** Andrew Tarzia, James E. M. Lewis, Kim E. Jelfs

**Affiliations:** ^1^ Department of Chemistry Molecular Sciences Research Hub Imperial College London White City Campus, Wood Lane London W12 0BZ UK

**Keywords:** cage compounds, computational screening, high-throughput, low-symmetry, self-assembly

## Abstract

Unsymmetrical ditopic ligands can self‐assemble into reduced‐symmetry Pd_2_L_4_ metallo‐cages with anisotropic cavities, with implications for high specificity and affinity guest‐binding. Mixtures of cage isomers can form, however, resulting in undesirable system heterogeneity. It is paramount to be able to design components that preferentially form a single isomer. Previous data suggested that computational methods could predict with reasonable accuracy whether unsymmetrical ligands would preferentially self‐assemble into single cage isomers under constraints of geometrical mismatch. We successfully apply a collaborative computational and experimental workflow to mitigate costly trial‐and‐error synthetic approaches. Our rapid computational workflow constructs unsymmetrical ligands and their Pd_2_L_4_ cage isomers, ranking the likelihood for exclusively forming *cis*‐Pd_2_L_4_ assemblies. From this narrowed search space, we successfully synthesised four new, low‐symmetry, *cis*‐Pd_2_L_4_ cages.

## Graphical abstract

Towards the development of sophisticated metal‐organic hosts, low‐symmetry metallo‐supramolecular cages assembled from unsymmetrical ligands are of emerging interest. Designing systems to avoid forming isomeric mixtures, however, is challenging. We report the use of a rapid computational workflow to predict self‐assembly outcomes, informing experimental decisions and optimising synthetic realisation of low‐symmetry, *cis*‐Pd_2_L_4_ cages.

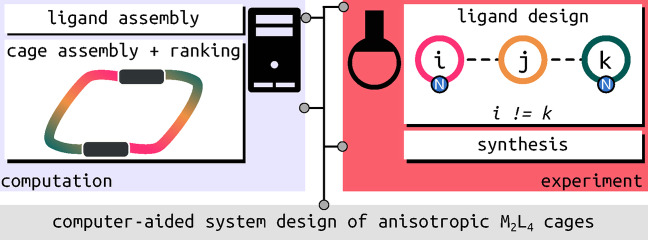

## Introduction

Nature has evolved spectacular control over self‐assembly processes to produce biological machinery for which high‐fidelity of composition and structure is essential for effective functionality. The exploitation of non‐covalent interactions allows complex architectures, such as enzymes, to exhibit high substrate specificity through precise control of binding‐site size, shape and positioning of functional groups. Over the last few decades, chemists have made great strides in developing approaches to utilise these principles for artificial systems. Metallo‐supramolecular chemistry has become a prevalent method for assembling ever‐more‐complex architectures using the predictable coordination geometry of transition metal ions.[^1^, ^2^, ^3^]

Since first being reported over twenty years ago,^[4]^ lantern‐type Pd_2_L_4_ cages,[^5^, ^6^, ^7^] assembled from “naked” Pd^II^ ions and ditopic ligands (L), have become an extensively studied class of metal‐organic polyhedra (MOPs).[^8^, ^9^, ^10^, ^11^, ^12^] Wide‐ranging applications for these cages have been investigated, including in drug delivery,[^13^, ^14^, ^15^] biomedicine,[^16^, ^17^, ^18^, ^19^, ^20^, ^21^] catalysis,[^22^, ^23^, ^24^, ^25^] and guest encapsulation/recognition.[^26^, ^27^, ^28^, ^29^, ^30^, ^31^, ^32^] To simplify the self‐assembly process, most previous reports have focussed on high‐symmetry systems derived from single, symmetrical ligands. However, it is expected that through the controlled introduction of asymmetry, cages could be designed with more intricate, anisotropic binding sites with specific shapes and functionalities.^[33]^

**Figure 1:**
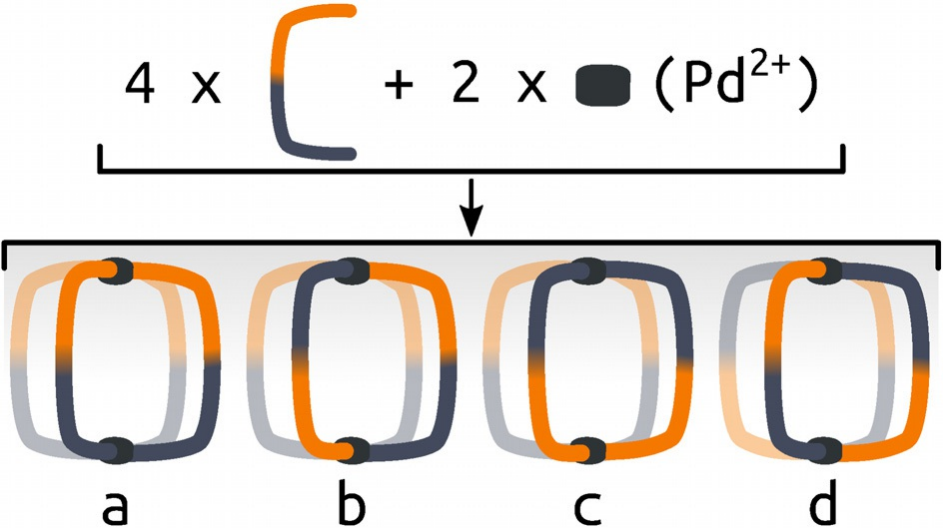
Representation of the self‐assembly of an unsymmetrical ditopic ligand and palladium(II) into four possible isomers of the homoleptic Pd_2_L_4_ cage: a) “all‐up”, b) “three‐up‐one‐down”, c) *cis* and d) *trans*. Orange and navy indicate inequivalent ligand fragments.

Pore asymmetry in M_2_L_4_ systems has been introduced through the controlled assembly of heteroleptic[^34^, ^35^, ^36^] and heteronuclear architectures.[^37^, ^38^] Mixed‐ligand [Pd_2_L^a^_2_L^b^_2_] assemblies have been realised through both steric[^39^, ^40^] and geometric control.[^41^, ^42^] Clever and co‐workers have demonstrated the effectiveness of pore asymmetry for improved binding of bent guests over linear counterparts.^[43]^ Crowley and co‐workers recently reported a [PdPtL_4_] cage in which the different labilities of the two metal ions allowed selective sequestration of the Pd^II^ ions to open the cage without complete dissociation of the ligands.^[44]^ We[^45^, ^46^] and others[^47^, ^48^, ^49^, ^50^] have recently begun to explore an alternative approach that uses unsymmetrical ligands to access lower symmetry structures.^[51]^ The lack of bilateral symmetry introduced into the ligand structure means four possible isomers of the resultant dipalladium cage can form (Figure 1). As with heteroleptic structures, high‐fidelity self‐sorting into a single isomer can be achieved using steric and/or geometric constraints. However, the inherent difficulty in designing such ligands that will reliably self‐sort helps explain the paucity of examples in the literature.

Density functional theory (DFT) calculations have been previously used to rationalise experimentally observed self‐sorting in low symmetry metallo‐supramolecular systems by exploring the relative energies of the potential configurational isomers.[^41^, ^43^, ^45^, ^47^, ^48^] Indeed, in recent work, we found that the formation of a single Pd_2_L_4_ cage isomer from the self‐assembly of unsymmetrical ditopic ligands with Pd^II^ ions only occurred when there was a significant difference in the calculated energies (on the order of at least 5 kJ mol^−1^) of the possible isomers.^[45]^ As such, we envisaged that a high‐throughput computational workflow could be used to rapidly explore the chemical space of low symmetry MOPs and aid in their design and minimise trial‐and‐error experimental efforts.

Computational screening has been successfully applied to aid in the rationalisation and/or prediction of self‐sorting outcomes of porous organic cages using the relative energetics of possible products.[^52^, ^53^, ^54^, ^55^] Until recently, however, it has not been possible to develop equivalent screening workflows for MOPs because no open‐source structure generation software was available. Some of us previously developed the supramolecular toolkit (*stk*),[^56^, ^57^] an open‐source Python framework that handles the structure prediction of supramolecular architectures. Here, we highlight the first use of *stk* to screen candidate MOPs. Young and co‐workers also recently developed the software *cgbind*, which performs structure prediction of M_2_L_4_ cages.^[58]^ The generalisability of *stk*, however, makes it ideal for this work, where we aim to explore a diverse set of ligand and cage structures.

In this work we present a high‐throughput computational workflow that was used to construct 60 unsymmetrical, ditopic ligands and the four possible Pd_2_L_4_ cage isomers for each in silico. Using metrics of geometrical stability and relative cage energies, the ligands were ranked based on their likelihood to form a single Pd_2_L_4_ isomer. A selection of five ligands with a range of rankings and chemistries were subsequently realised experimentally, and their self‐assembly examined. This computer‐aided approach facilitates an experimental design with a high success rate, leading to several new unsymmetrical *cis*‐Pd_2_L_4_ cages being prepared. In this manner, the discovery of interesting candidates can be accelerated by providing a likelihood of success, expediting the synthesis of low symmetry MOPs with desirable structural properties. Indeed, this work highlights that a computer‐aided approach allows a more efficient exploration of a larger chemical space of potential candidates than a purely experimental approach.

## Results and Discussion

**Figure 2:**
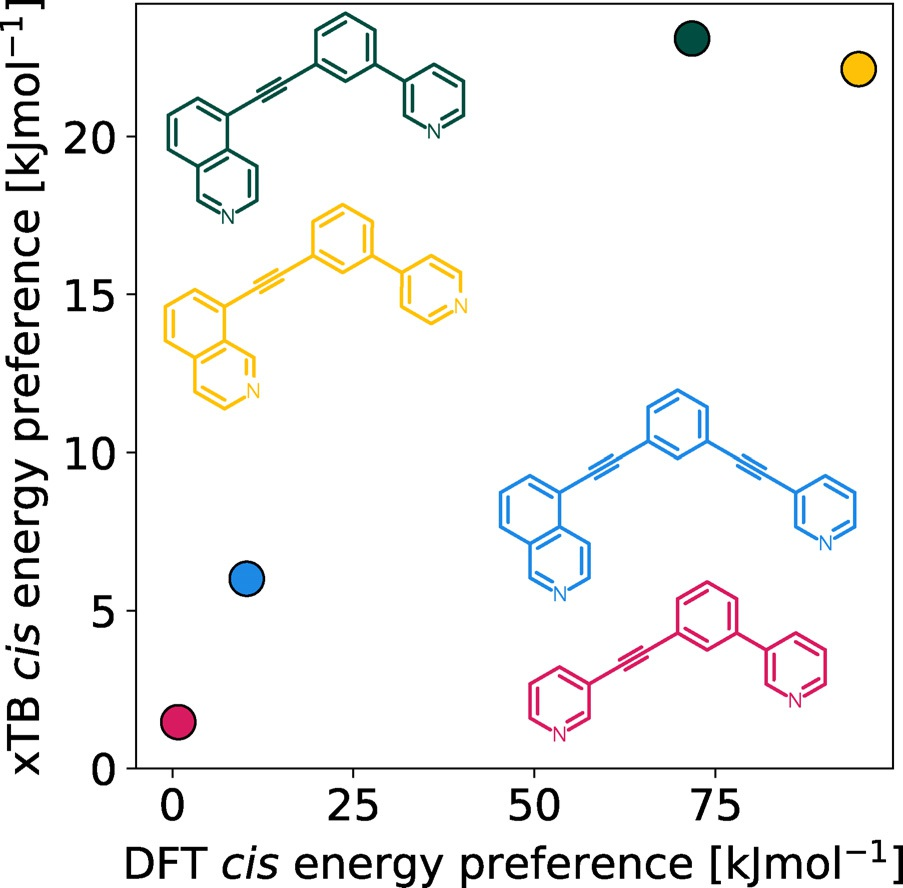
Comparison of GFN2‐xTB (DMSO) and DFT (PBE0/def2‐SVP/D3BJ/CPCM(DMSO)) energy difference between the *cis* and next most stable isomer of cages formed in ref. [45] from the ligands **3D1** (crimson), **4D2** (yellow), **5D1** (dark green) and **5D3** (blue).

In previous work it was found that the energy separations of the isomers of [Pd_2_L_4_]^4+^ cages assembled from unsymmetrical ligands, calculated using DFT methods, correlated well with experimental observations of single isomer formation.^[45]^ For larger data sets, it would be desirable to use efficient, semi‐empirical methods for geometry optimisations and energy calculations to reduce computational cost and increase throughput. Therefore, we tested whether the xTB family of semi‐empirical methods^[59]^ could capture the same relative energy differences between Pd_2_L_4_ cage isomers as DFT methods. The xTB methods are tight‐binding quantum chemical methods for the geometry optimisation of systems containing elements up to *Z=*86, and represent a robust and significantly cheaper alternative to DFT for metal‐containing species.^[60]^ A comparison of the xTB (specifically GFN2‐xTB) and DFT‐calculated energies of previously reported systems was undertaken (structures were taken directly from the computational workflow described below). DFT energies were obtained from single‐point energy evaluations of xTB geometries using similar methods to those recently applied to related systems^[25]^ (the final selected method uses the PBE0^[61]^ level of theory using the Ahlrichs basis set def2‐SVP,[^62^, ^63^] Grimme's D3BJ dispersion correction^[64]^ and the polarizable continuum model (PCM)^[65]^ implicit solvation representing DMSO; more details and a comparison to the B97‐3c composite method are available in Supporting Information Section S3). The same trends in relative energies were found from both methods (Figure 2) and for free energies calculated using GFN2‐xTB (Table S2), suggesting that GFN2‐xTB can reasonably represent the relative energetics of the studied cage systems. Therefore, xTB methods were applied throughout this work for geometry optimisations and for energy evaluations toward a high‐throughput workflow.

**Figure 3:**
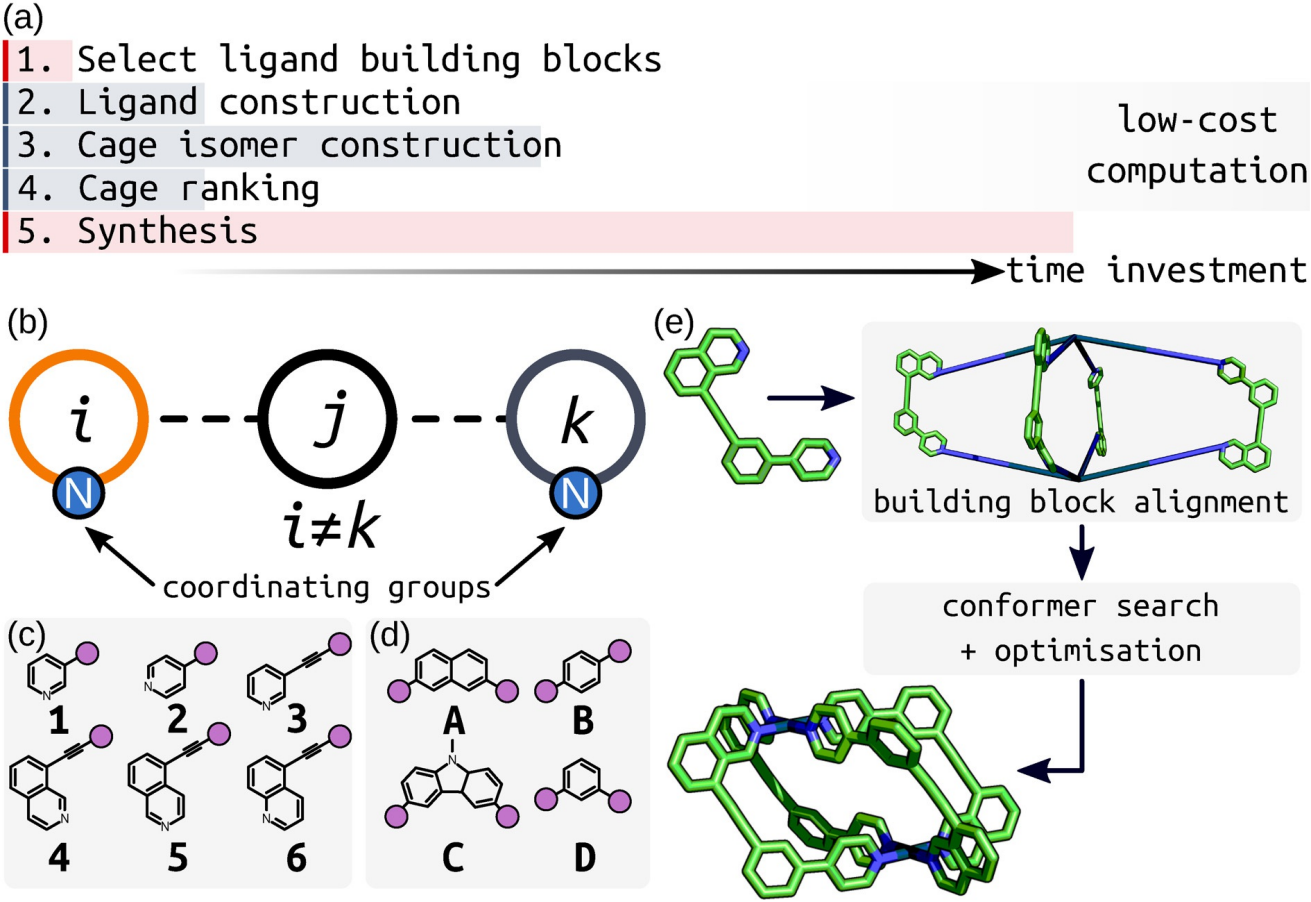
a) The joint experimental and computational workflow showing relative time frames of each step. b) Unsymmetrical cage ligands *ijk* formed from three building blocks with the nitrogen coordinating groups highlighted. The library of c) coordinating building blocks and d) core building blocks used to construct the cage ligands in this study. Purple circles are the connection points between coordinating and core building blocks. e) Assembly of a *cis*‐Pd_2_L_4_ structure from an unsymmetrical ligand. Firstly, the Pd^II^ ions and ligands are placed and aligned on the M_2_L_4_ topology vertices by *stk*, generating an expanded structure. Geometry optimisation, coupled with a conformer search, was then performed on this structure to give the optimised geometry.

In this work, a joint computational and experimental workflow (Figure 3 a) was implemented to facilitate the search for new unsymmetrical ligands with sufficient geometrical constraints to drive the exclusive formation of single Pd_2_L_4_ cage isomers. This approach started with an initial experimental choice of building blocks. These were combined in silico to form the candidate ligands and their possible Pd_2_L_4_ cage isomers. The cage systems were then analysed using computationally cheap structural parameters to assess the likelihood of successful self‐assembly into a single isomer, thus assisting in the synthetic decision‐making process. A focus was placed on using relatively low‐cost computational approaches before any experimental investment to minimise wasted efforts. Such an approach opens up the ability to search for new unsymmetrical cages with desirable properties, increasing ligand design efficiency (which is currently based on very few experimental examples).

A major goal of this computational workflow is generalisability, rendering it applicable to a large chemical space that can be adapted for future iterations of the process. To this end, *stk*,[^56^, ^57^] UFF4MOF (Universal Force Field for MOFs[^66^, ^67^]) and the xTB family of semi‐empirical methods[^59^, ^68^] were used for the assembly and geometry optimisation of ligand and cage structures. *stk* assembles building blocks onto topology graphs; this process includes the placement, alignment and reaction of the building blocks. Before optimisation with xTB, conformer searches were performed on the Pd_2_L_4_ systems using a broadly applicable force field that can handle common metal complex geometries (UFF4MOF implemented in the General Utility Lattice Program (GULP)[^69^, ^70^]). Coupling the above methods affords low‐cost structure generation, conformer searching and geometry optimisation (Figure 3 e). For each cage, the construction, conformer search and optimisation (at the xTB level) processes took approximately 2–3 hours on a workstation with an i7‐9700K CPU (3.60 GHz), which allows for the evaluation of a ligand and its four isomers overnight on standard computer hardware.

To construct a range of unsymmetrical ditopic cage ligands using *stk*, the ligand structure was partitioned into three building blocks (Figure 3 b): a core (**A**–**D** in Figure 3 d) separating two inequivalent coordinating building blocks (**1**–**6** in Figure 3 c). The building blocks were selected from structures commonly used in metallo‐supramolecular systems. In this initial study, a relatively small selection of building blocks was used, giving a combinatorial library of 60 unsymmetrical ditopic ligands. With four possible Pd_2_L_4_ cage isomers for each ligand, a library of 240 cages was generated. Through enumeration of this set of common building blocks, a set of unconventional unsymmetrical cage ligands were generated and tested. The step‐wise computational assembly and optimisation of ligands and cages (see Supporting Information Section S2) through *stk* is automated from the point of input of the ligand building blocks as text‐based SMILES strings (Table S1). Therefore, this work is straightforward to extend to a larger chemical space using our hierarchical approach.

In the final step of the computational workflow, the assembled cages associated with each ligand were analysed to determine if a single Pd_2_L_4_ isomer (targeting the *cis* isomer) would be expected to form to the exclusion of others. Our validation of GFN2‐xTB (see above) for providing relative energies of different Pd_2_L_4_ structures suggested that a GFN2‐xTB energy separation of ca. 6 kJ mol^−1^ between the two lowest energy isomers (Δ*E*) appeared to be sufficient to drive exclusive formation of the lowest energy cage isomer. Of the 60 ligands in the library, 34 (57 %), including three previously reported examples, had Δ*E* values of at least 6 kJ mol^−1^, indicating that they might be promising candidates for experimental synthesis.

**Figure 4:**
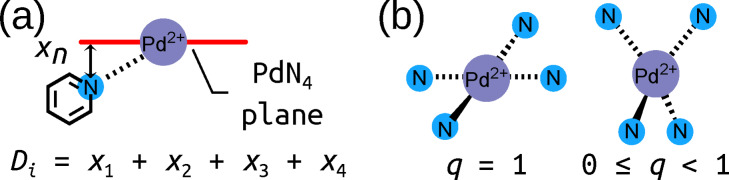
a) Representation of *D*_i_ as the sum of the distance of four nitrogen atoms (one is shown) and the palladium atom from the plane defined by a PdN_4_ unit. *D*_max_ is the maximum *D*_i_ of the two in a Pd_2_L_4_ cage. b) Representation of the square planar order parameter *q*_sqp_.^[72]^

**Figure 5:**
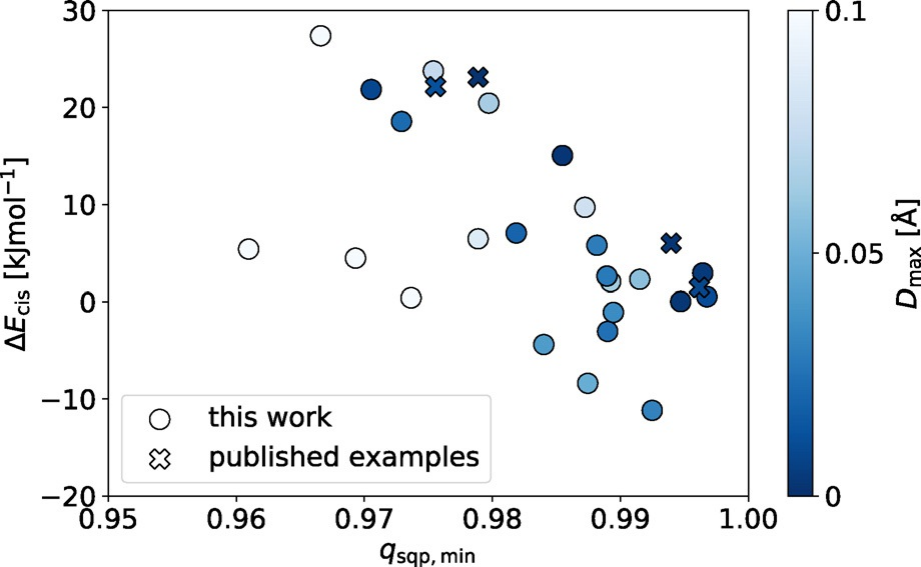
The relative stability of the *cis* cage isomer (Δ*E*_cis_) for each ligand as a function of *q*_sqp,min_ of the calculated *cis*‐Pd_2_L_4_ structure. Negative values of Δ*E*_cis_ indicate that the *cis* isomer is not the most stable. Only structures with *q*_sqp,min_ >0.95 are shown here; the full data set is shown in Figure S3 a.

While the relative xTB energies provide information about the likely self‐sorting behaviour of Pd_2_L_4_ cage isomers, they do not indicate if the desired Pd_2_L_4_ topology will be the favoured product of self‐assembly, rather than a larger species. In fact, the prediction of the preferred topology of palladium cages requires costly computational methods.^[71]^ To bypass these methods, the assumption was made that if the Pd_2_L_4_ cage is geometrically stable, then, as the smallest possible Pd_*n*_L_2*n*_ assembly, it is likely to form as the entropically favoured product. As a simple means to probe this, two structural metrics common to every cage in the library were employed (Figure 4): the maximum sum of the deviation of the four nitrogen atoms and palladium atom from each calculated average PdN_4_ plane (*D*_max_; 0.0 Å indicating no deviation) and the minimum square planar order parameter (*q*_sqp,min_;^[72]^ 1.0 indicating a perfect square planar geometry) of the Pd^II^ ions. Both measures quantify the degree of square‐planar‐likeness in the most strained palladium ion of the cage. It is assumed that if the strain in the cage is significant, Pd_2_L_4_ formation will be enthalpically unfavourable. By applying Δ*E* (specifically Δ*E*_cis_, which is the relative stability of the *cis* cage isomer), *D*_max_ and *q*_sqp,min_ within the workflow as computationally cheap heuristics, cage ligands with desirable properties can be selected for synthesis from the generated rankings favouring those that appear most likely to give successful self‐assembly outcomes (where success is defined as exclusive formation of a single cage isomer). Figure 5 shows the relationship between the three heuristics used for ranking candidates.

Of the 34 ligands with Δ*E* values ≥6 kJ mol^−1^, 12 had lowest energy cage isomers exhibiting very favourable *D*_max_ and *q*_sqp,min_ values <0.1 Å and >0.95, respectively. Pleasingly, three had previously been synthesised and shown to exclusively form *cis*‐Pd_2_L_4_ isomers.^[45]^ Perhaps unsurprisingly, in each of these 12 instances, the *cis*‐Pd_2_L_4_ isomer was predicted to be the favoured structure, again in agreement with previous work.[^45^, ^47^, ^48^] Indeed, for most of the ligands (51 of 60; 85 %), the *cis* cage isomer was found to be the most stable (Figure S4a).

**Figure 6:**
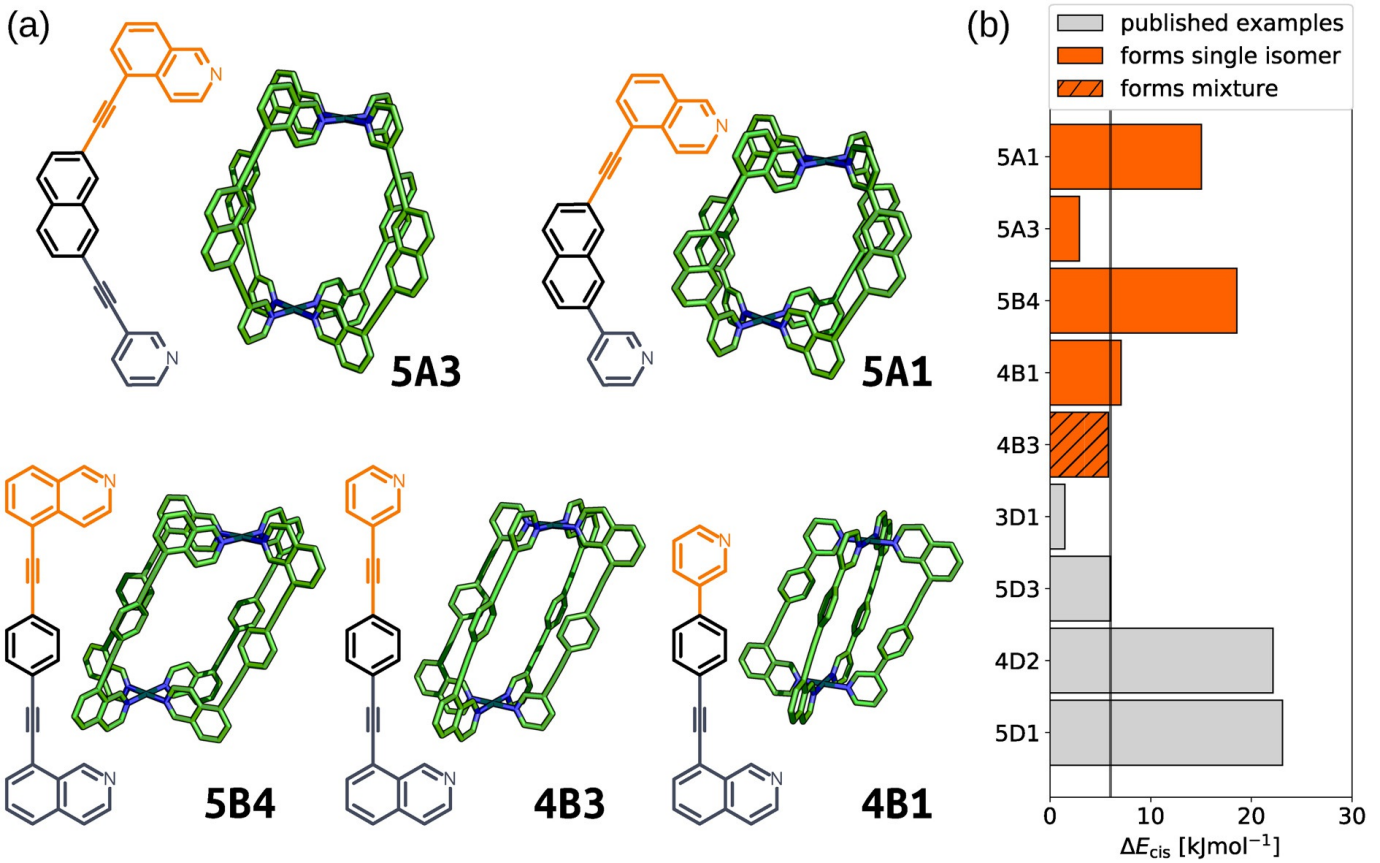
a) GFN2‐xTB optimised structure of the *cis* isomer of selected cage ligands (hydrogen atoms omitted; C green, N blue, Pd cyan). Cage ligands are shown next to each structure with orange and navy indicating inequivalent ligand fragments. b) *cis* isomer GFN2‐xTB(DMSO) stability (Δ*E*_cis_) for all published^[45]^ and newly selected ligands (patterns distinguish their self‐assembly outcomes).

Five previously unreported ligands were selected for synthesis to investigate their self‐assembly with Pd^II^ (Figure 6). These included four ligands from the 12 that adhered to the chosen parameter thresholds (Table 1), including with naphthalene (**5A1**) and para‐phenylene (**4B1**, **4B3**, **5B4**) core building blocks, with combinations of pyridyl/isoquinolyl coordinating building blocks. A fifth ligand, **5A3**, was also selected that displayed good structural parameters (*D*_max_=0.0 Å; *q*_sqp,min_=1.0) but a low energy separation (Δ*E*_cis_=2.9 kJ mol^−1^) to probe the fidelity of the Δ*E* value as a quantitative metric in predicting isomer equilibria, given the necessary simplicity of the workflow's modelling parameters. The ligands were prepared using standard synthetic techniques, and their identities confirmed by NMR spectroscopy and mass spectrometry (MS). In each instance, the ligand self‐assembly with Pd^II^ was examined by combining the ligand and [Pd(CH_3_CN)_4_](BF_4_)_2_ in a 2:1 ratio in [D_6_]DMSO (followed by standing at room or elevated temperature for a period of time, as necessary to reach equilibrium).

**Table 1:** Calculated properties of the *cis* isomer of the selected ligands.^[a]^

Ligand	*cis* isomer		Δ*E* [kJ mol^−1^]		Experimental outcome
	*q*_sqp,min_	*D*_max_	xTB	DFT	
**5B4**	0.973	0.023	18.5	71.0	*cis*‐Pd_2_L_4_
**5A1**	0.985	0.003	15.0	24.6	*cis*‐Pd_2_L_4_
**4B1**	0.982	0.020	7.0	21.1	*cis*‐Pd_2_L_4_
**4B3**	0.988	0.030	5.8	16.0	isomeric mixture
**5A3**	0.996	0.005	2.9	7.0	*cis*‐Pd_2_L_4_

[a] Energy separations (Δ*E*) are the difference in energy from the *cis* isomer to the next most stable isomer at both levels of theory. *D*_max_ is in Å.

**Figure 7:**
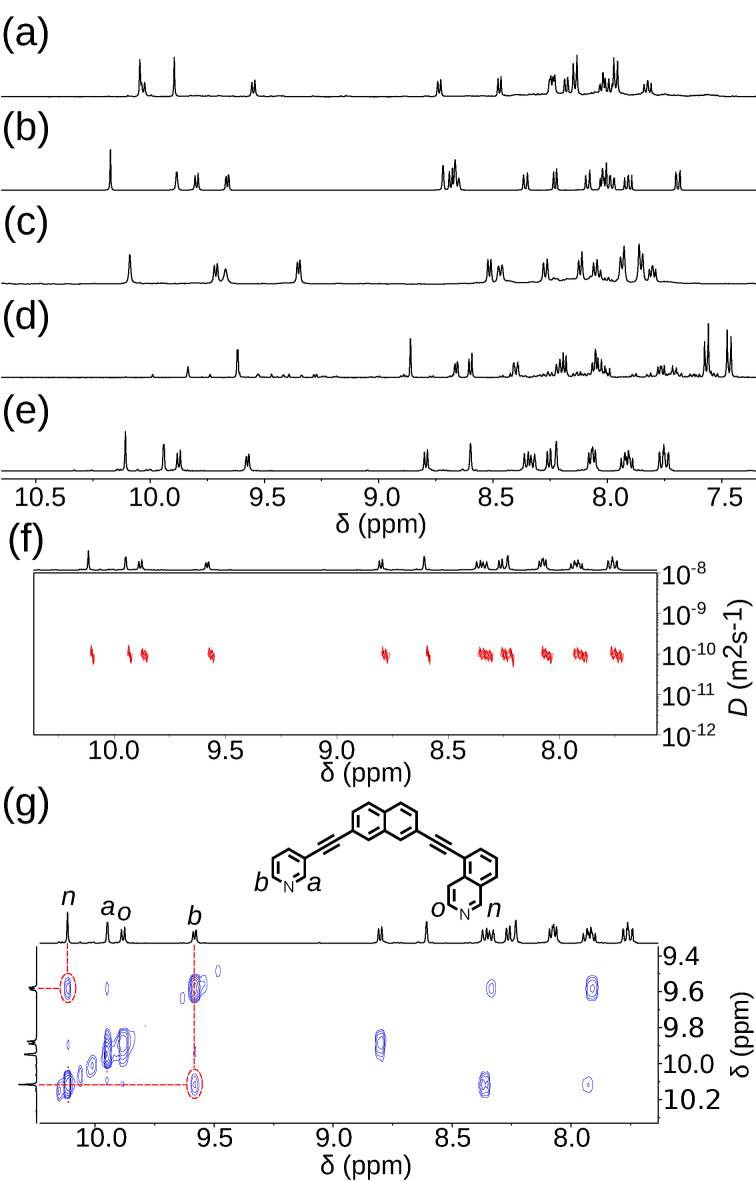
Partial ^1^H NMR spectra (500 MHz, [D_6_]DMSO, 298 K) of equilibrated mixtures of 1:2 [Pd(CH_3_CN)_4_](BF_4_)_2_ and a) **5B4**, b) **5A1**, c) **4B1**, d) **4B3**, and e) **5A3**. f) DOSY, and g) NOESY (500 MHz, [D_6_]DMSO, 298 K) spectra of [Pd_2_(**5A3**)_4_](BF_4_)_4_.

For the three ligands with calculated energy differences in excess of 6.0 kJ mol^−1^ (**5B4**, **5A1**, and **4B1**), quantitative conversion to a single species was observed by ^1^H NMR (Figure 7 a–c, respectively) and diffusion‐ordered spectroscopy (DOSY). Calculated solvodynamic radii (*R*_s_) from the latter (10.2 Å, 8.4 Å and 9.7 Å, respectively) indicated formation of assemblies of similar size to the calculated Pd_2_L_4_ cage structures. Additionally, isotopic patterns consistent with MOPs of these formulas were found by MS. Through‐space interactions between the inequivalent coordinating moieties of the ligands were observed by NOESY which, alongside the symmetry of the ^1^H NMR spectra, dictated that either the *cis* or *trans* isomers had been formed. Disappointingly, despite multiple attempts, no single crystals suitable for study by X‐ray diffraction were obtained. Based on the calculated structures and extrapolating from previous work,^[45]^ however, we are confident that the structures obtained were the anticipated *cis*‐Pd_2_L_4_ cages.

In the case of **4B3** a major product formed but, even after prolonged heating, multiple species could still be observed by ^1^H NMR (Figure 7 d). Although it could not be determined absolutely, using similar reasoning to that outlined above, it was concluded that the *cis*‐Pd_2_L_4_ cage was the major species present in solution. It was clear that, under the conditions examined, the difference in energy between this major species and other potential products was not sufficient to drive the exclusive formation of a single assembly.

Intriguingly, despite there being less than 3.6 kJ mol^−1^ difference in energy between the calculated structures of the *cis*, *trans* and “three‐up‐one‐down” isomers of [Pd_2_(**5A3**)_4_]^4+^, a single species was found to form upon the self‐assembly of **5A3** with Pd^II^ (Figure 7 e). Once again, DOSY (Figure 7 f; *R*_s_=10.4 Å), MS, the symmetry of the ^1^H NMR spectrum and cross‐peaks observed by NOESY (Figure 7 g) led to the conclusion that either the *cis* or *trans* assembly had formed. Disappointingly, without single‐crystal X‐ray diffraction, whether the *cis* or *trans* isomer of the [Pd_2_(**5A3**)_4_]^4+^ assembly had formed could not be determined with absolute certainty with the spectroscopic data available.

From the five ligands examined experimentally, the calculated *cis*‐Pd_2_L_4_ structures of three (**5B4**, **5A1** and **4B1**) adhered strictly to our estimated parameter values necessary for quantitative self‐assembly of these assemblies. In each instance, exclusive formation of a single Pd_2_L_4_ isomer was observed and concluded to be the anticipated *cis*‐Pd_2_L_4_ by spectroscopic and computational data. For two of the ligands (**4B3** and **5A3**), the xTB values of Δ*E* fell at or below the predicted threshold. Interestingly, the ligand associated with the lower value of Δ*E* (**5A3**) successfully self‐assembled into a single Pd_2_L_4_ cage isomer, whilst the other formed an isomeric mixture. It can be concluded that smaller values of Δ*E* make predictions of self‐assembly outcomes more precarious. This is likely due to effects not taken into consideration within the current computational workflow, such as template effects from anions and/or solvent molecules. Such computationally expensive factors were purposefully omitted to streamline the process and increase throughput. Higher values of Δ*E*, however, appear to be associated with increased experimental success rates and highlight the efficacy of the workflow for indicating systems with the greatest chance of forming single isomers of the desired cage topology.

For the five synthesised ligands, we compared the xTB‐calculated energy separations of their isomers with DFT single point calculations and found that the relative energy relationships were similar (Supporting Information Section S5). The relative stabilities of the *cis* isomers do change among these candidates. This suggests that ranking the ability of each ligand to self‐sort using xTB, whilst qualitatively equivalent to DFT, is not quantitative. Additionally, the value of the relative energy threshold changes from ca. 6 kJ mol^−1^ to ca. 10 kJ mol^−1^ for the methods applied here based on the energy separation of **5D3**. These energy differences are well within DFT error for such complex systems and GFN2‐xTB^[59]^ is not parameterised to produce energies accurately. However, this validation supports that the suggestions made by the computational rankings would be equivalent if more costly DFT methods were used.

**Figure 8:**
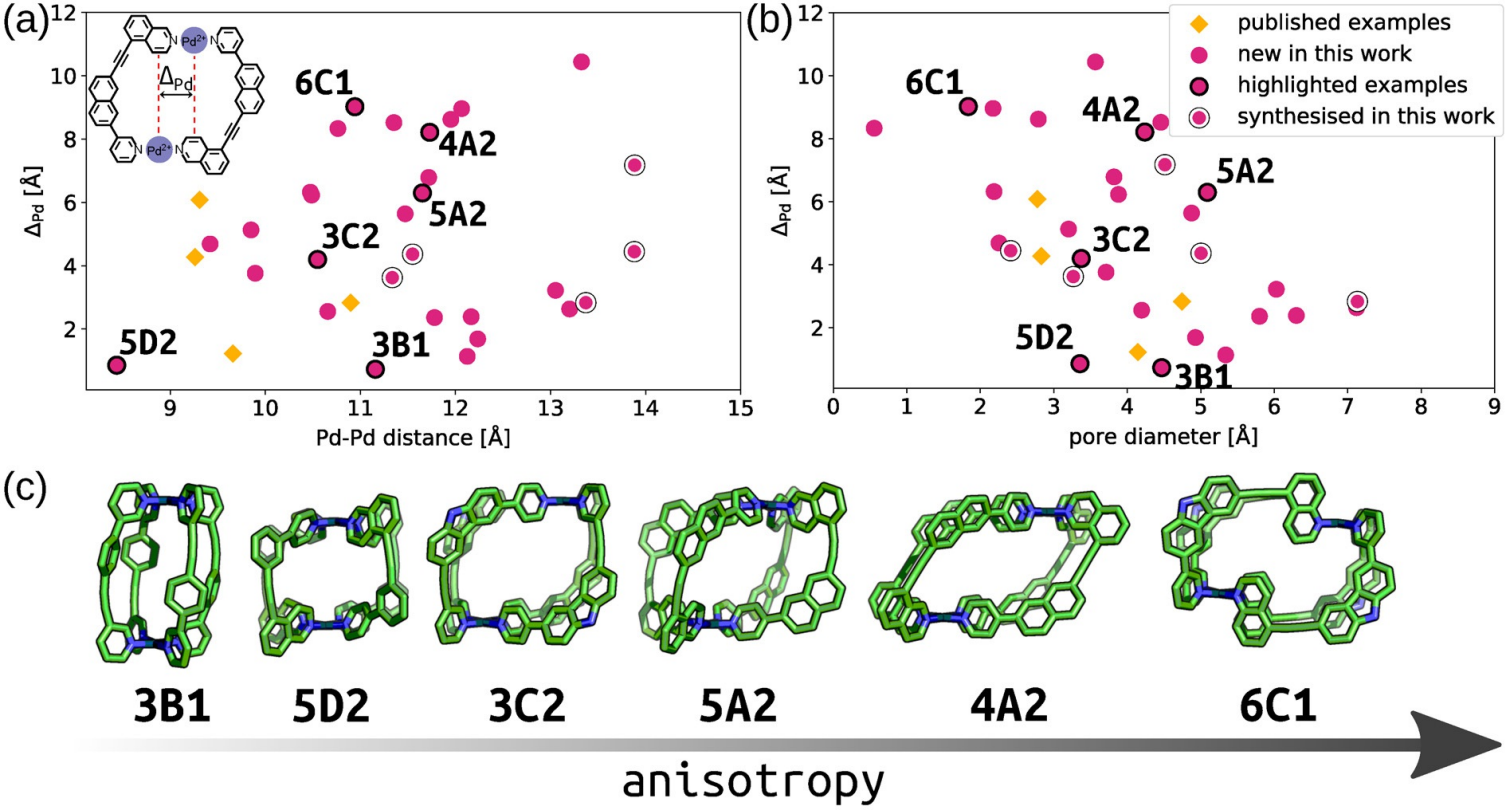
Cage anisotropy (Δ_Pd_) as a function of a) Pd⋅⋅⋅Pd distance (Δ_Pd_ inset) and b) pore diameter (only cages with *q*_sqp,min_ >0.9 shown). c) Example cages with increasing anisotropy; none of these examples were synthesised and are not necessarily thermodynamically favourable structures.

Although five high‐ranking candidates were selected for synthesis, models of many potential assemblies were generated in the computational workflow. This allows for an exploration of these calculated systems to search for those with desirable structural properties. The controlled introduction of anisotropy is of particular interest to the development of cages with advanced functionality.^[33]^ For these Pd_2_L_4_ systems, a simple definition of anisotropy would be the displacement of the Pd^II^ centres from alignment perpendicular to the PdN_4_ planes (Δ_Pd_, shown inset in Figure 8 a). For Pd_2_L_4_ systems assembled from symmetrical ligands, Δ_Pd_ should be 0 Å. To this end, an analysis of Δ_Pd_ values compared to Pd⋅⋅⋅Pd distance (Figure 8 a) and pore size^[73]^ (Figure 8 b) was undertaken; example structures with increasing anisotropy are shown in Figure 8 c.

The combined family of *cis*‐Pd_2_L_4_ cages previously reported^[45]^ and realised in this work are represented within this analysis. A diverse anisotropy‐property space is demonstrated with Δ_Pd_ values ranging from 1.2 to 7.2 Å and Pd⋅⋅⋅Pd distances between 9.3 and 11.3 Å. The successfully synthesised cages also show interesting, non‐linear, relationships between their size (Pd⋅⋅⋅Pd) and calculated pore diameter (using a spherical probe), which we expect to be a crucial property to control for the application of reduced‐symmetry cages. The calculated *cis*‐Pd_2_L_4_ structures from ligands **4A2** and **6C1**, for example, are of interest as they represent high‐anisotropy assemblies with large and small pore diameters, respectively. Indeed, *cis*‐[Pd_2_(**6C1**)_4_]^4+^ possesses two effectively isolated binding pockets within the cavity, in contrast to the large, single pore of *cis*‐[Pd_2_(**4A2**)_4_]^4+^ (Figure 8 c).

## Conclusion

The synthesis and investigation of unsymmetrical Pd_2_L_4_ assemblies with asymmetric pores, with potential utility in high specificity and affinity guest binding properties, is a growing field. The design of such systems to ensure high‐fidelity self‐assembly, however, remains non‐trivial. Here we have shown that a simple and low‐cost computational workflow can be used to inform decisions in experimental work, resulting in a “high hit‐rate” synthesis of targeted unsymmetrical *cis*‐Pd_2_L_4_ cages. The open‐source and generalisable computational procedure provided efficient, and sufficiently accurate, predictions of cage structures starting from a combinatorially constructed library of 60 unsymmetrical ligands. Using a computational ranking scheme based on a small number of cheap and calculable metrics (parameterised based on limited existing experimental results), we have realised four previously unreported low‐symmetry, *cis*‐Pd_2_L_4_ cages, greatly expanding the existing repertoire of these systems and validating our workflow. Additionally, this work is a platform for further exploring the chemical space of unsymmetrical Pd_2_L_4_ assemblies. In this manner, the synthetic chemist can choose ligands and/or cages with desirable properties with confidence in the reliability of their self‐assembly profile.

It was shown that a hierarchical and combinatorial computational screening approach, facilitated by open‐source software, allowed the construction of large precursor and cage libraries for high‐throughput screening. While focussed initially on a limited number of common building blocks, expanding the initial precursor library is trivial because the only required inputs to the automated workflow are SMILES strings. Additionally, the use of common building blocks did not limit the generation of unconventional and novel unsymmetrical cage ligands in this work. The applied computational workflow can be generalised to future problems to explore a much larger chemical space of metal‐organic cages and other materials classes.

Finally, our experimental efforts led to one ligand out of five for which self‐assembly with Pd^II^ did not produce a single species. In agreement with the metrics employed, however, a single cage isomer did appear to be predominate. This highlights that the heuristics applied do not capture all of the necessary information to ensure absolute fidelity of self‐sorting, and we suggest that the role of ligand flexibility and explicit solvent/counter‐ion templation could be significant. However, given the simplicity and high‐throughput nature of this approach, it is remarkably effective for informing experimental decisions. As experimental data in this field is still limited, additional information obtained from this and future studies will help recognise metrics of importance to incorporate into the workflow, leading to a refinement of the process. Ultimately this will lead to improved certainty in future synthetic decisions using a joint computational and experimental discovery workflow. All code used in this work is available at https://github.com/andrewtarzia/unsymm match. All structure data and ligand ranking is available at https://github.com/andrewtarzia/citable data/tree/master/tarzia lewis 2021.

## Conflict of interest

The authors declare no conflict of interest.

## Supporting information

As a service to our authors and readers, this journal provides supporting information supplied by the authors. Such materials are peer reviewed and may be re‐organized for online delivery, but are not copy‐edited or typeset. Technical support issues arising from supporting information (other than missing files) should be addressed to the authors.

Supporting Information
