# Supplementary material for: High‐Throughput Computational Evaluation of Low Symmetry Pd2L4 Cages to Aid in System Design
Source: Angew Chem Int Ed Engl. 2021 Aug 11;60(38):20879–87. doi: 10.1002/anie.202106721 (PMC8518684; doi:10.1002/anie.202106721)
Supplement: Supplementary file 1 — Supporting Information [file ANIE-60-20879-s001.pdf]

Angewandte Chemie
Eine Zeitschrift der Gesellschaft Deutscher Chemiker

Supporting Information

High-Throughput Computational Evaluation of Low Symmetry Pd_2L_4 Cages to Aid in System Design**

Andrew Tarzia, James E. M. Lewis, and Kim E. Jelfs**

anie_202106721_sm_miscellaneous_information.pdf

Contents

1. Input to screening workflow 2

2. Ligand and cage assembly 3

3. Density functional theory calculations 5

4. Cage analysis 7

5. Selected candidate DFT validation 9

6. Definition of the cage anisotropy 10

7. General Experimental 11

8. Synthetic Procedures 12

Synthesis of **S1** 12

Synthesis of **S2** 16

Synthesis of **5A1** 19

Synthesis of **S3** 22

Synthesis of **S4** 25

Synthesis of **5A3** 28

Synthesis of **S5** 31

Synthesis of **S6** 34

Synthesis of **4B1** 37

Synthesis of **S7** 40

Synthesis of **4B3** 43

Synthesis of **S8** 46

Synthesis of **S9** 49

Synthesis of **5B4** 52

Synthesis of $[Pd_2(\mathbf{5A1})_4](BF_4)_4$ 55

Synthesis of $[Pd_2(\mathbf{5A3})_4](BF_4)_4$ 60

Synthesis of $[Pd_2(\mathbf{4B3})_4](BF_4)_4$ 65

Synthesis of $[Pd_2(\mathbf{4B1})_4](BF_4)_4$ 71

Synthesis of $[Pd_2(\mathbf{5B4})_4](BF_4)_4$ 76

9. Solvodynamic Radii Calculations 81

10. References 82

1. Input to screening workflow

Table S1 shows the input SMILES strings for the cage ligand building blocks. SMILES is a text-based representation of molecular connectivity used throughout cheminformatics.

Table S1 SMILES strings and names for the coordinating and core building blocks used in this work. Bromine functional groups are used as the connection points in *stk* for constructing the ligands.

name	SMILES
	coordinating building blocks
1	Brc1cccnc1
2	Brc1ccncc1
3	BrC#Cc1cccnc1
4	BrC#Cc1cccc2ccncc12
5	BrC#Cc1cccc2cnccc12
6	BrC#Cc1cccc2ncccc12
	core building blocks
A	Brc1ccc2ccc(Br)cc2c1
B	Brc1ccc(Br)cc1
C	Brc1ccc2[nH]c3ccc(Br)cc3c2c1
D	Brc1cccc(Br)c1

2. Ligand and cage assembly

The cage ligands and cages were assembled with our Python software, *stk*,[1,2] using the linear polymer and M_2L_4 lantern topology graphs, respectively. Recent updates to *stk* allow the handling of metal-containing systems including a diverse variety of metal-organic cages and metal-complexes. The *stk* assembly process, which robustly handles a wide variety of cage molecules,[3,4] i) places building blocks on the nodes of a topology graph, and ii) aligns them, based on their functional groups, along the edges connecting that node to its neighbours. The linear polymer simply places all building blocks in a line and aligns the functional groups of neighbouring building blocks along that line. In this work, cage ligands were built from three building blocks that were connected through bromine functional groups (the choice of bromine is arbitrary and was not meant to represent a realistic chemical disconnection). Cage ligands were initially optimised using version 2 of the ETKDG algorithm in RDKit, which builds a reasonable 3D structure of the ligand.[5,6] A ligand conformer where the two coordinating nitrogen atoms are pointing in the same direction improves robustness of the cage construction process (Figure S1). Therefore, we automatically selected the ligand conformer (out of 100 ETKDG conformers) with the smallest angle between the two coordinating nitrogen atoms and the ligands centre of mass. This ligand conformer was optimised at the “very tight” level using GFN2-xTB[7] (version 6.2 was used throughout this work) and used as a building block in the cage assembly process. All structure optimisation was performed using our software package, *stko* (https://github.com/JelfsMaterialsGroup/stko).

The cage assembly process places single palladium atoms on two four-connected nodes of the M_2L_4 lantern topology graph and the cage ligand building blocks on four two-connected nodes, which bridge the two four-connected nodes. In this work, all cage ligands had two nitrogen-centred pyridyl functional groups, which were used to align them on the topology graph. In our software, *stko*, we have recently implemented methods to optimise metal-containing systems using the UFF4MOF forcefield[8,9] in the General Utility Lattice Program (GULP; version 5.1),[10,11] and the xtb software.[7,12] UFF4MOF is an extended version of the universal force field (UFF)[13] that handles metal environments common in metal—organic framework structures (note that only the original parameters[15] were required for square planar palladium metal centres). The GFN*n*-xTB methods are recently developed and very efficient semiempirical quantum mechanical methods that were parametrised for large parts of the periodic table (up to Z=86)[7,14] and have been demonstrated to be reliable for the optimisation of large transition-metal containing structures.[12]

After construction with *stk*, the lowest energy cage conformer was found using the following sequence of optimisation steps:

1. *stk* assembles structures based on predefined topology graphs with unphysical, long bonds between building blocks (nodes). The expanded structure is collapsed to a realistic size, while maintaining the shape of the assembled structure, by translating each rigid building block toward the centre of mass of the assembled structure. The algorithm (“Collapser” in *stko*) stops when the inter-building block distance is less than 2 Å to avoid steric clashes.
2. The cage structure is geometry optimised using UFF[13] in GULP. The atom typing is handled by a Python implementation of the “ForceFieldHelpers” module in RDKit,[6] except for the metal atoms, which are manually typed to match the target types in UFF (because RDKit does not handle the metal-atom typing). Palladium atoms are assigned the square planar atom type, “Pd4+2”. The bonding used within GULP matches the bonding in the *stk* molecule.

3. A conformer search is performed starting from the UFF optimised cage structure using high-temperature molecular dynamics (MD). Two sequential MD runs in the NVT ensemble, using the leapfrog verlet integrator, are performed using UFF and GULP at 1000 K. The first run is a short equilibration with a time step of 0.25 fs for 1.0 ps. The production run is performed for 100.5 ps with a time step of 0.75 fs. From the production run, 100 conformers are extracted at 1.0 ps intervals.
4. Each extracted conformer is optimised at the “normal” level using GFN2-xTB with DMSO as the implicit solvent (with the “very tight” solvent grid option).
5. The lowest energy cage conformer is optimised using GFN2-xTB with the “extreme” convergence criteria and DMSO as the implicit solvent (with the “very tight” solvent grid option). Frequency calculations at the GFN2-xTB level were only performed on the top candidates and previously synthesised structures and confirmed no imaginary frequencies.

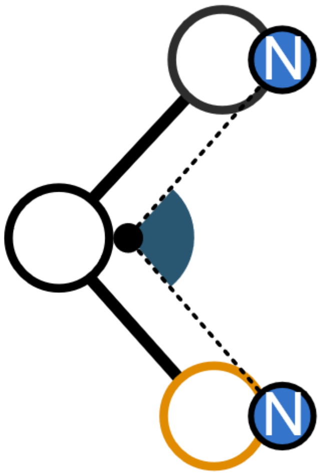

Figure S1 Schematic of the optimal cage ligand conformation where the nitrogen—centre of mass—nitrogen angle (shown in dark blue) is minimised.

The workflow we implemented takes only the linker and ligand building blocks SMILES strings as input and automatically assembles, optimises and analyses the cage ligands and resultant cage isomers. Such an approach relies on reasonable default parameters at each step that can handle cages with vastly different chemical and physical properties. We parametrised this workflow on the four experimental cases from ref. 15, governed by only geometrical constraints, to ensure that the same cage properties (energy and structure) were obtained over multiple runs of our procedure. During production, we found a very small number of examples where the optimisation sequence could not be completed automatically, which we handled by manually performing the optimisation sequence. Our automated optimisation process can lead to very unstable structures for cage ligands that cannot, geometrically, form Pd_2L_4 cages or are very flexible, which we aimed to overcome by the high temperature MD step to “escape” unstable configurations. As part of our selection process, we visualised all top candidate cage structures and found false positives based on our three metrics (energy separation, $\boldsymbol{q}_{\mathrm{sqp,min}}$ and $\boldsymbol{D}_{\mathrm{max}}$) where all four isomers are strained and would not be expected to form in solution. We found examples of high ligand strain that may be stabilised by π··· π interactions between the cage ligands, which leads to helicate-like structures. Our findings suggest that there is a balance between cage ligand flexibility and geometric complementarity that governs Pd_2L_4 formation.

3. Density functional theory calculations

We use density functional theory (DFT) single point energy (SPE) energy calculations to validate the relative energetics of the four isomers of many cage structures obtained from the screening workflow. We did not perform geometry optimisations at any DFT level because of the cost of these calculations. We tested a variety of methods on four sets of cage isomers using Gaussian 16 with the "Superfinegrid" option.[16] Figure S2 shows that the relative energetics between the *cis* isomer (C isomer) are equivalent in these cases between the xTB energy and DFT energies (of a series of methods). For the remainder of this work, we perform SPE calculations using the PBE0[17] (or "PBE1PBE" in Gaussian 16) method with the def2-SVP basis set[18,19] and Grimme's D3BJ dispersion correction,[20] in implicit dimethyl sulfoxide (DMSO) using the polarizable continuum model (PCM).[21] This method was recently shown to be effective for Pd_2L_4 cage systems.[22] Each palladium was assigned a charge of 2 and was assumed to be in the low-spin state.

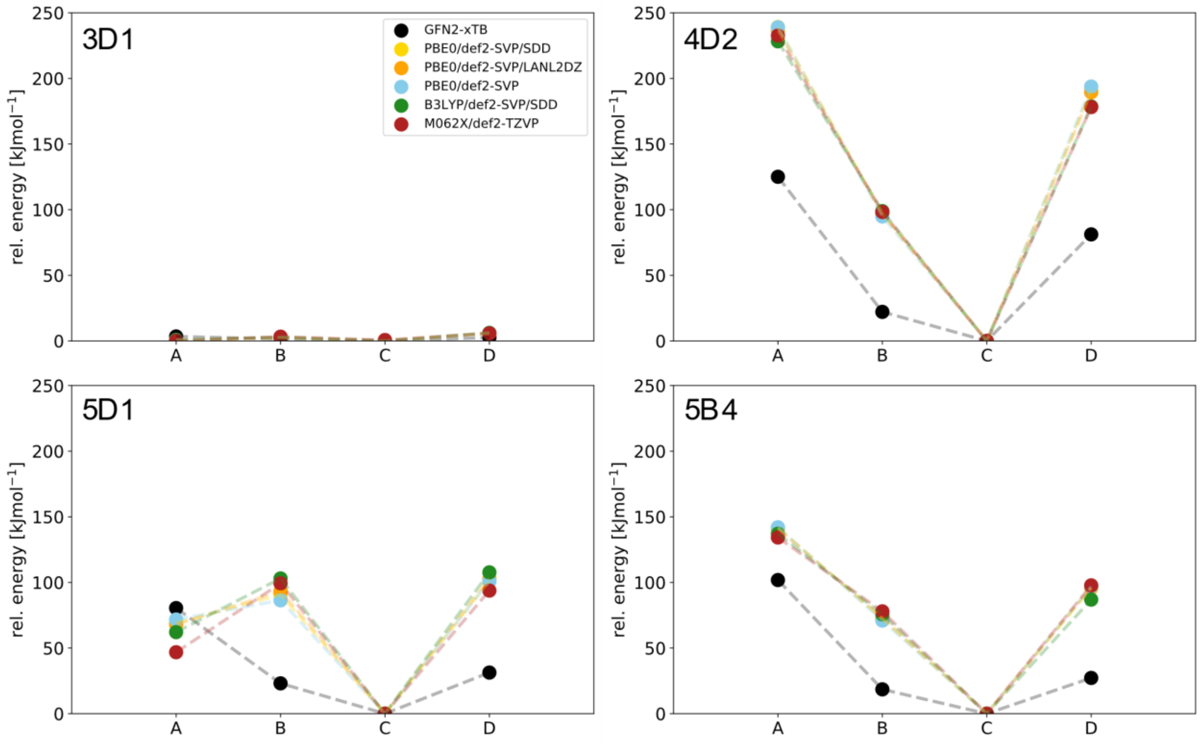

Figure S2 Relative energy of all isomers for four cage sets at different levels of theory. All energies were calculated on structures from the geometry optimised structure at the GFN2-xTB level of theory.

Table S2 and S3 show the GFN2-xTB free energies and B97-3c, a composite DFT method, energies relative to the *cis* isomer for the previously reported cage structures. The free energies were calculated using the "--hess" option in xTB from the GFN2-xTB optimized structures. No negative frequencies were found. The B97-3c[23] energies were calculated using Orca 4.2.1[24] and performed in the gas-phase due to SCC issues with the CPCM method. Importantly, similar trends are observed for both methods compared to Figure S2.

Table S2 GFN2-xTB (DMSO) cage isomer free energies for a selected set of ligands. All free energies are in kJ mol^{-1} and relative to the *cis* isomer (c) of the cage set. Δ corresponds to the difference in energy between the *cis* isomer and the next most stable isomer, where negative values indicate that the *cis* isomer is more stable.

isomer	3D1	4D2	5D1	5D3
**a**	7.1	134.6	78.9	24.3
**b**	1.5	28.1	27.6	5.4
**c**	0.0	0.0	0.0	0.0
**d**	2.6	84.2	31.0	5.4
**Δ**	-1.5	-28.1	-27.6	-5.4

Table S3 B97-3c (gas phase) Orca cage isomer energies for a selected set of ligands. All energies are in kJ mol^{-1} and relative to the *cis* isomer (c) of the cage set. Δ corresponds to the difference in energy between the *cis* isomer and the next most stable isomer, where negative values indicate that the *cis* isomer is more stable.

isomer	3D1	4D2	5D1	5D3
**a**	1.0	227.7	56.2	23.2
**b**	2.1	85.6	91.3	10.6
**c**	0.0	0.0	0.0	0.0
**d**	6.7	161.5	94.4	20.6
**Δ**	-1.0	-85.6	-56.2	-10.6

Input files and output files for xTB and DFT calculations can be accessed at https://github.com/andrewtarzia/citable_data/tree/master/tarzia_lewis_2021.

4. Cage analysis

Computationally efficient methods were used to analyse the stability and energetic preference of metal-organic cage isomers in a screening workflow. Firstly, isomer preference was determined based on the relative GFN2-xTB[7,14] energy of the lowest energy conformer obtained of each isomer with DMSO as the implicit solvent (with the "very tight" solvent grid option). Secondly, the stability of a cage was screened based on simple geometric descriptors that determine how far from an ideal square planar geometry the palladium metal-centres deviate when in the cage. Two cheap methods were used for calculating the geometric stability of a cage: the maximum plane deviation ($\boldsymbol{D}_{\text{max}}$) and the minimum square planar order parameter ($\boldsymbol{q}_{\text{sqp,min}}$)[25] of both metal centres in a cage. Both $\boldsymbol{D}_{\text{max}}$ and $\boldsymbol{q}_{\text{sqp,min}}$ are designed to quantify the degree of distortion in the most strained metal centre in a cage and are reasonably well correlated (Figure S3(b)). The plane deviation of a metal centre was calculated as the sum of the shortest distance of each bound nitrogen atom and the palladium atom from the plane of best fit defined by the palladium atom and the four nitrogen atoms (Figure 4). $\boldsymbol{q}_{\text{sqp,min}}$ was calculated using the default implementation in pymatgen (version 2019.5.8), where the neighbours of the palladium atoms were manually set to the four coordinated nitrogen atoms.[26] Throughout this work, we used the geometrical measures of stability ($\boldsymbol{D}_{\text{max}}$ and $\boldsymbol{q}_{\text{sqp,min}}$) mostly as guidelines when making experimental decisions. All pore sizes were calculated using our open-source Python package pyWindow.[27]

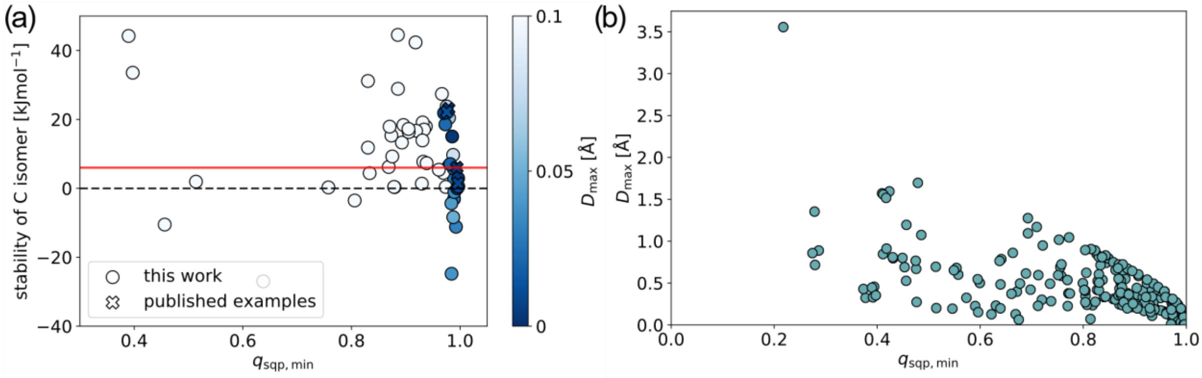

Figure S3 (a) The relative stability of the *cis* isomer formed from each cage as a function of $q_{\text{sqp,min}}$ of the *cis* isomer. Negative values for the stability indicate that the *cis* isomer is not the lowest energy isomer. The horizontal red line is the xTB energy threshold for clean isomer formation. Points are coloured by their D_{max}. (b) D_{max} of all cages as a function of $q_{\text{sqp,min}}$.

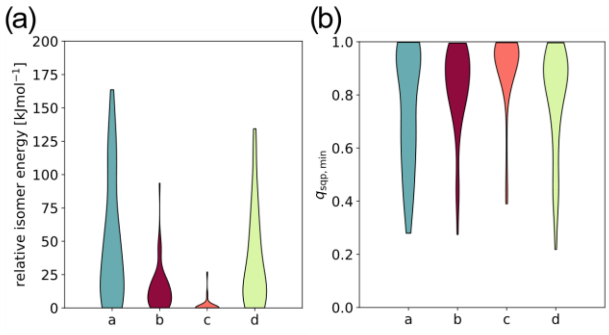

Figure S4 Violin plots showing the distribution of the (a) relative energy values and (b) $q_{\mathrm{sqp,min}}$ of all cages assembled in this work, separated by isomer.

5. Selected candidate DFT validation

Tables S4 and S5 show the GFN2-xTB and DFT energies relative to the *cis* isomer for all previously reported and selected cages. All DFT energies are from SPE calculations of the xTB optimised structure.

Table S4 GFN2-xTB (DMSO) cage isomer energies for a selected set of ligands. All energies are in kJ mol^{-1} and relative to the *cis* isomer (c) of the cage set. Δ corresponds to the difference in energy between the *cis* isomer and the next most stable isomer, where negative values indicate that the *cis* isomer is more stable.

isomer	3D1	4D2	5D1	5D3	4B1	4B3	5A1	5A3	5B4
a	3.4	125.1	80.5	22.0	50.5	41.9	42.4	12.8	101.8
b	1.5	22.1	23.1	6.1	15.4	12.0	15.0	3.6	18.5
c	0.0	0.0	0.0	0.0	0.0	0.0	0.0	0.0	0.0
d	2.6	81.2	31.3	6.0	7.0	5.8	17.2	2.9	27.2
Δ	-1.5	-22.1	-23.1	-6.0	-7.0	-5.8	-15.0	-2.9	-18.5

Table S5 PBE0-D3BJ/def2-SVP/PCM(DMSO) cage isomer energies for a selected set of ligands. All energies are in kJ mol^{-1} and relative to the *cis* isomer (c) of the cage set. Δ corresponds to the difference in energy between the *cis* isomer and the next most stable isomer, where negative values indicate that the *cis* isomer is more stable.

isomer	3D1	4D2	5D1	5D3	4B1	4B3	5A1	5A3	5B4
a	0.8	239.0	71.8	23.3	50.6	46.9	36.7	14.1	142.0
b	2.9	94.8	86.4	10.3	21.1	16.0	24.6	7.0	71.0
c	0.0	0.0	0.0	0.0	0.0	0.0	0.0	0.0	0.0
d	6.2	193.8	100.8	21.6	54.3	66.0	64.4	13.8	97.0
Δ	-0.8	-94.8	-71.8	-10.3	-21.1	-16.0	-24.6	-7.0	-71.0

6. Definition of the cage anisotropy

We define a measure of cage anisotropy, “Pd displacement”, based on the relative geometry of the square-planar palladium (II) centres in the assembled cage structures. The algorithm for calculating Pd displacement (Δ_{Pd}) is:

1. Calculate the plane of best fit, and normal to the plane $\hat{n}$, of one Pd atom and its four bonded N atoms.
2. Calculate the Pd-Pd vector, $\hat{v}$.
3. Calculate the projection, $\hat{p}$, of $\hat{v}$ on the plane of best fit as

$$\hat{p} = \hat{v} - \frac{\hat{v}\cdot\hat{n}}{\|\hat{n}\|^2}\hat{n} \qquad (1)$$

4. Calculate $\Delta_{\mathrm{Pd}} = \|\hat{p}\|$.

7. General Experimental

Synthesis: Unless otherwise stated, all reagents, including anhydrous solvents, were purchased from commercial sources and used without further purification. $CDCl_3$ and NEt_3 were stored over 4 Å molecular sieves prior to use. All reactions were carried out under an atmosphere of N_2 using degassed, anhydrous solvents unless otherwise stated. Petrol refers to the fraction of petroleum ether boiling in the range 40-60 °C. Analytical TLC was performed on pre-coated silica gel plates (0.25 mm thick, 60F254, Merck, Germany) and observed under UV light. EDTA solution refers to a 0.1 M solution of EDTA-Na_2 in 3% $NH_{3(aq)}$.

Analysis: NMR spectra were recorded on Bruker AV400 or AV500 instrument, at a constant temperature of 298 K. Chemical shifts are reported in parts per million from low to high field and referenced to residual solvent. Standard abbreviations indicating multiplicity were used as follows: m = multiplet, quint = quintet, q = quartet, t = triplet, d = doublet, s = singlet, app. = apparent, br. = broad. Signal assignment was carried out using 2D NMR methods (HSQC, HMBC, COSY, NOESY) where necessary. In the case of some signals absolute assignment was not possible. Here indicative either/or assignments (e.g. H_A/H_B for H_A or H_B) are provided. All melting points were determined using a hot stage apparatus and are uncorrected. Mass spectrometry was carried out by the Imperial College London, Department of Chemistry Mass Spectroscopy Service using Waters LCT Premier for HR-ESI-MS and Thermo Scientific Q-Exactive for tandem MS.

The following compounds were synthesised according to a literature procedure: 5-iodoisoquinoline[28] and 8-iodoisoquinoline.[29]

8. Synthetic Procedures

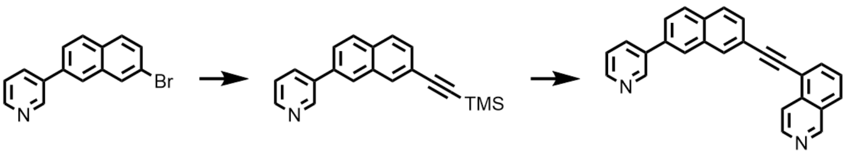

S1 **S2** **5A1**

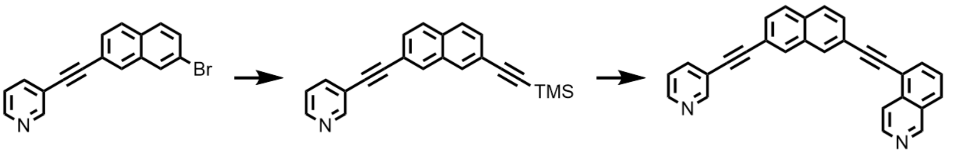

S3 **S4** **5A3**

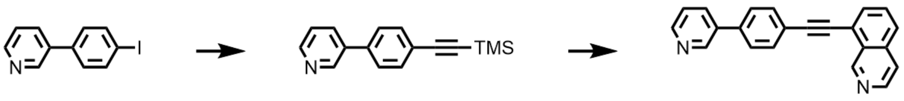

S5 **S6** **4B1**

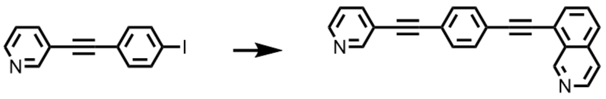

S7 **4B3**

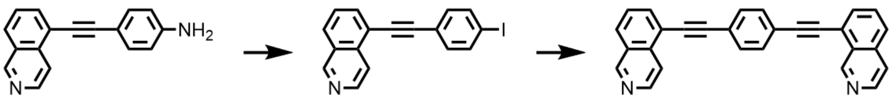

S8 **S9** **5B4**

Synthesis of S1

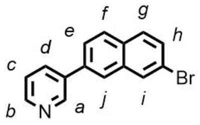

2,7-Dibromonaphthalene (0.572 g, 2.0 mmol, 1.0 eq.), 3-pyridinylboronic acid (0.246 g, 2.0 mmol, 1.0 eq.), $Pd(PPh_3)_2Cl_2$ (0.035 g, 0.050 mmol, 2.5 mol%) and K_2CO_3 (0.691 g, 5.0 mmol, 2.5 eq.) were stirred at 100 °C in 2:1 dioxane/H_2O (12 mL) in a sealed vial for 18 h. EDTA solution (15 mL) was added and the mixture extracted with CH_2Cl_2 (2 × 25 mL). The combined organic extracts were dried ($MgSO_4$) and the solvent removed *in vacuo*. After purification by column chromatography on silica (CH_2Cl_2 followed by 1:9 EtOAc/CH_2Cl_2) the product was obtained as a white solid (0.231 g, 41%). ^{1}H NMR (400 MHz, $CDCl_3$) δ: 8.97 (d, J = 1.9 Hz, 1H, H_a), 8.65 (dd, J = 4.9, 1.6 Hz, 1H, H_b), 8.08 (d, J = 1.9 Hz, 1H, H_i), 8.04 (ddd, J = 7.9, 2.4, 1.6 Hz, 1H, H_d), 7.94-7.92 (m, 2H, H_f, H_j), 7.76 (d, J = 8.8 Hz, 1H, H_g), 7.71 (dd, J = 8.5, 1.9 Hz, 1H, H_e), 7.60 (dd, J = 8.7, 2.0 Hz, 1H, H_h), 7.47 (ddd, J = 7.9, 4.9, 0.9 Hz, 1H, H_c). ^{13}C NMR (101 MHz, $CDCl_3$) δ: 148.1, 147.9, 136.7, 136.0, 135.5, 134.8, 131.5, 130.4, 130.1, 129.5, 129.1, 125.5, 125.4, 124.1, 120.9. HR-ESIMS m/z = 284.0069 $[M+H]^+$ calc. 284.0069.

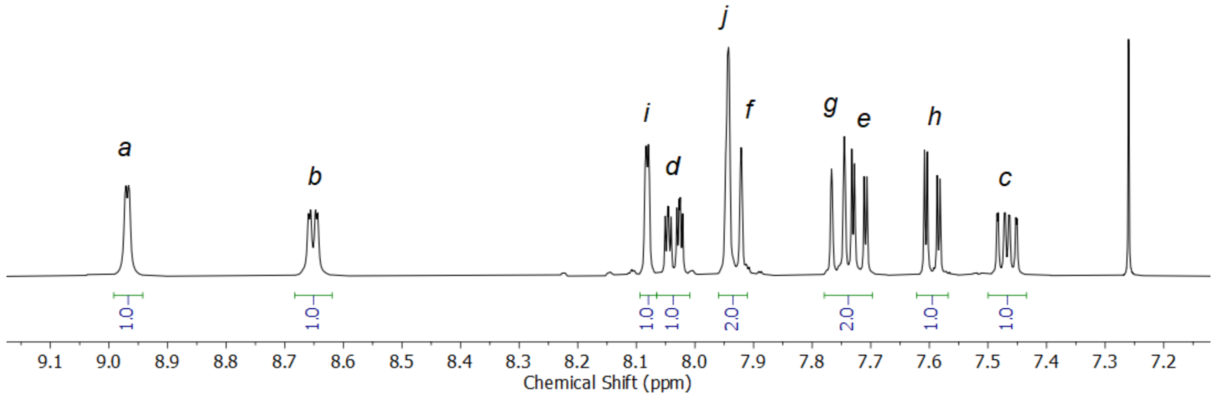

Figure S5 ^{1}H NMR ($CDCl_3$, 400 MHz) of **S1**.

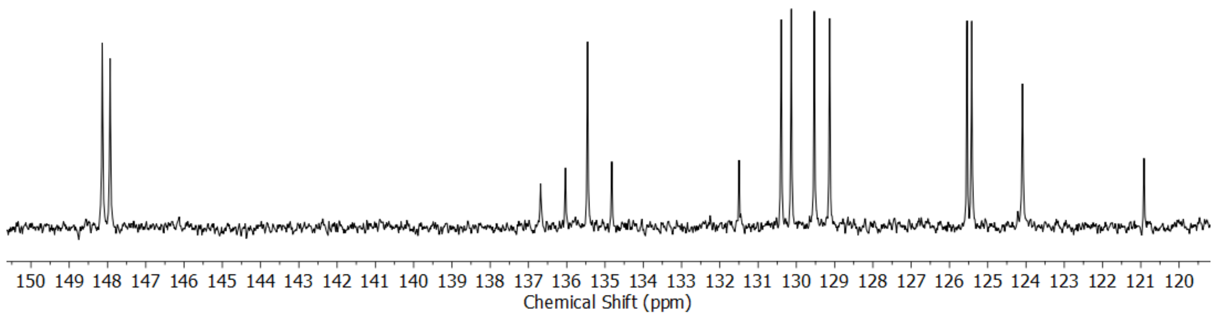

Figure S6 ^{13}C NMR ($CDCl_3$, 101 MHz) of **S1**.

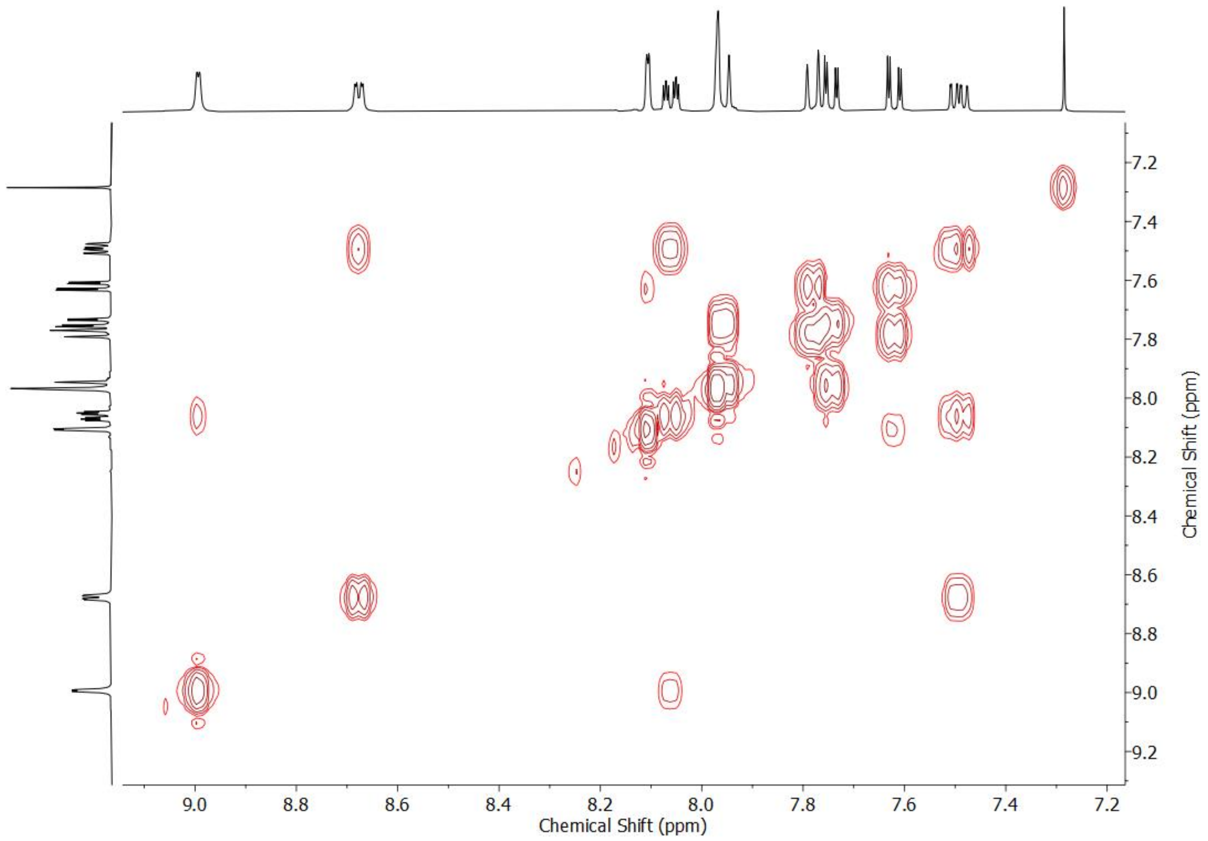

Figure S7 COSY NMR ($CDCl_3$) of **S1**.

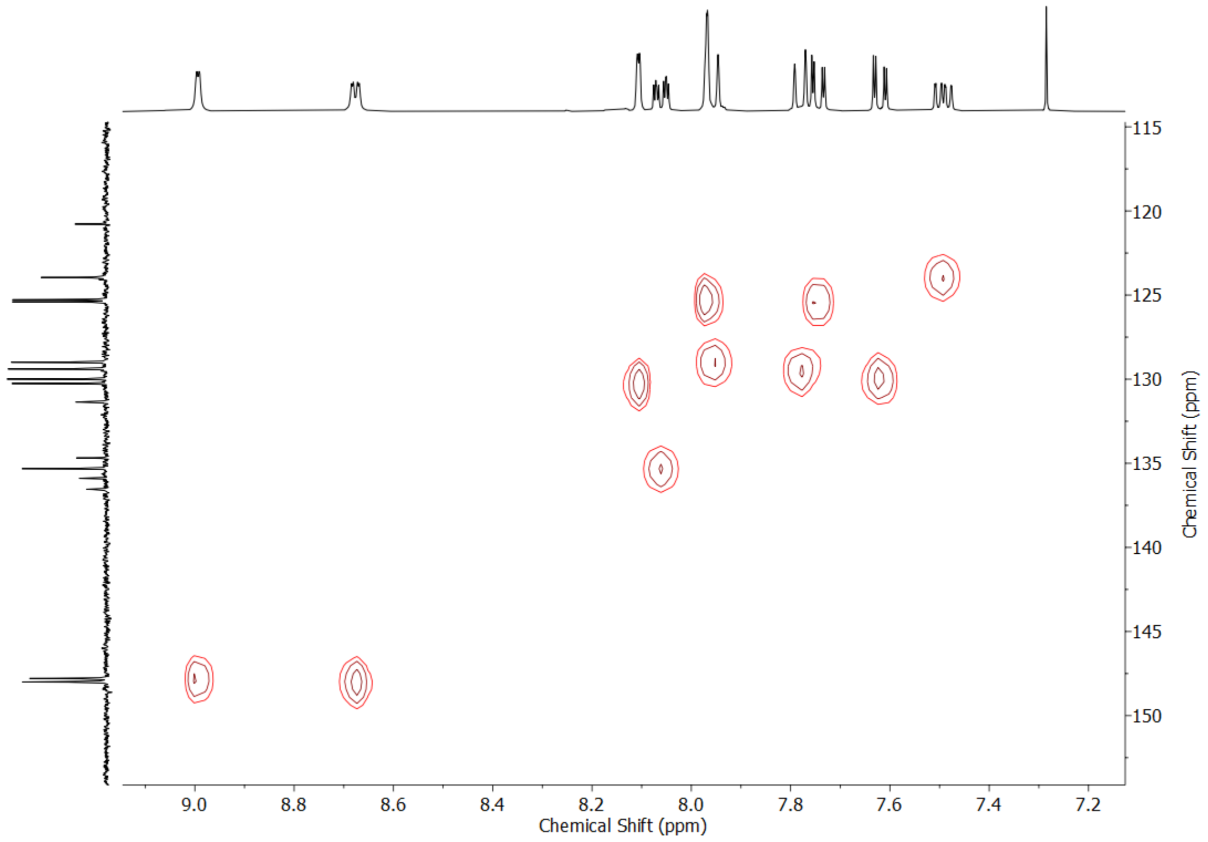

Figure S8 HSQC NMR ($CDCl_3$) of **S1**.

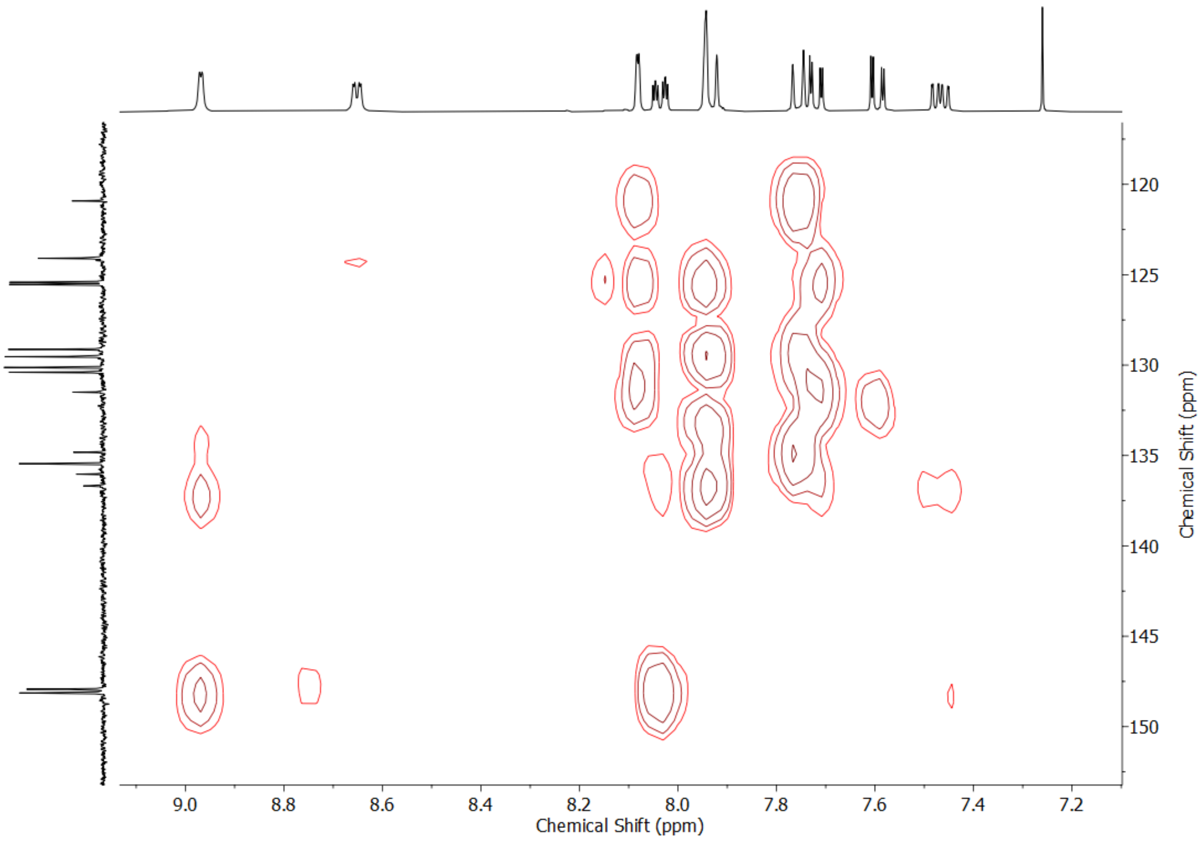

Figure S9 HMBC NMR ($CDCl_3$) of **S1**.

Synthesis of S2

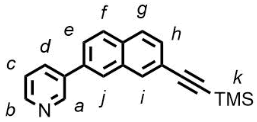

S1 (218 mg, 0.767 mmol, 1.0 eq.), trimethylsilylacetylene (0.13 mL, 0.96 mmol, 1.25 eq.), [Pd(PPh_3)$_2$$Cl_2$] (13.5 mg, 0.0192 mmol, 2.5 mol%) and CuI (7.3 mg, 0.038 mmol, 5 mol%) were stirred at 80 °C in $^iPr_2NH$ (7.5 mL) in a sealed vial for 21 h. After the solvent was removed *in vacuo*, EDTA solution (25 mL) was added and the mixture extracted with CH_2Cl_2 (3 × 25 mL). The combined organic extracts were dried ($MgSO_4$) and the solvent removed *in vacuo*. After purification by column chromatography on silica (1:19 EtOAc/CH_2Cl_2) the product was obtained as a light yellow solid (0.218 g, 94%). ^{1}H NMR (400 MHz, $CDCl_3$) δ: 8.97 (br. s, 1H, H_a), 8.70 (br. s, 1H, H_b), 8.07 (d, *J* = 0.9 Hz, 1H, H_i), 8.00-7.97 (m, 2H, H_d, H_j), 7.92 (d, *J* = 8.5 Hz, 1H, H_f), 7.80 (d, *J* = 8.4 Hz, 1H, H_g), 7.71 (dd, *J* = 8.5, 1.8 Hz, 1H, H_e), 7.54 (dd, *J* = 8.5, 1.6 Hz, 1H, H_h), 7.42 (br. dd, *J* = 7.7, 4.6 Hz, 1H, H_c), 0.30 (s, 9H, H_k). ^{13}C NMR (101 MHz, $CDCl_3$) δ: 148.9, 148.7, 136.5, 136.4, 136.1, 134.7, 133.2, 132.4, 132.4, 129.4, 128.9, 127.8, 126.1, 123.8, 121.4, 105.3, 95.3, 0.1. HR-ESIMS m/z = 302.1359 [M+H]$^+$ calc. 302.1360.

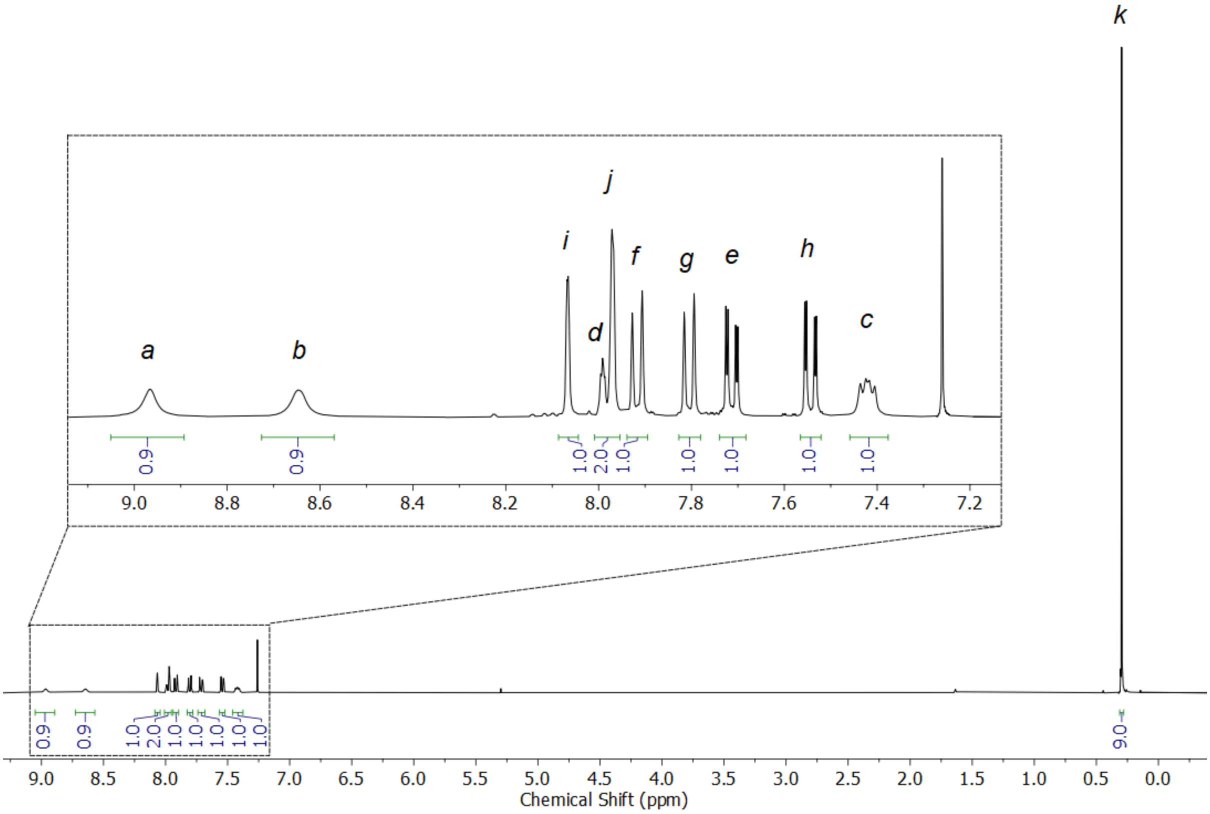

Figure S10 ^{1}H NMR ($CDCl_3$, 400 MHz) of **S2**.

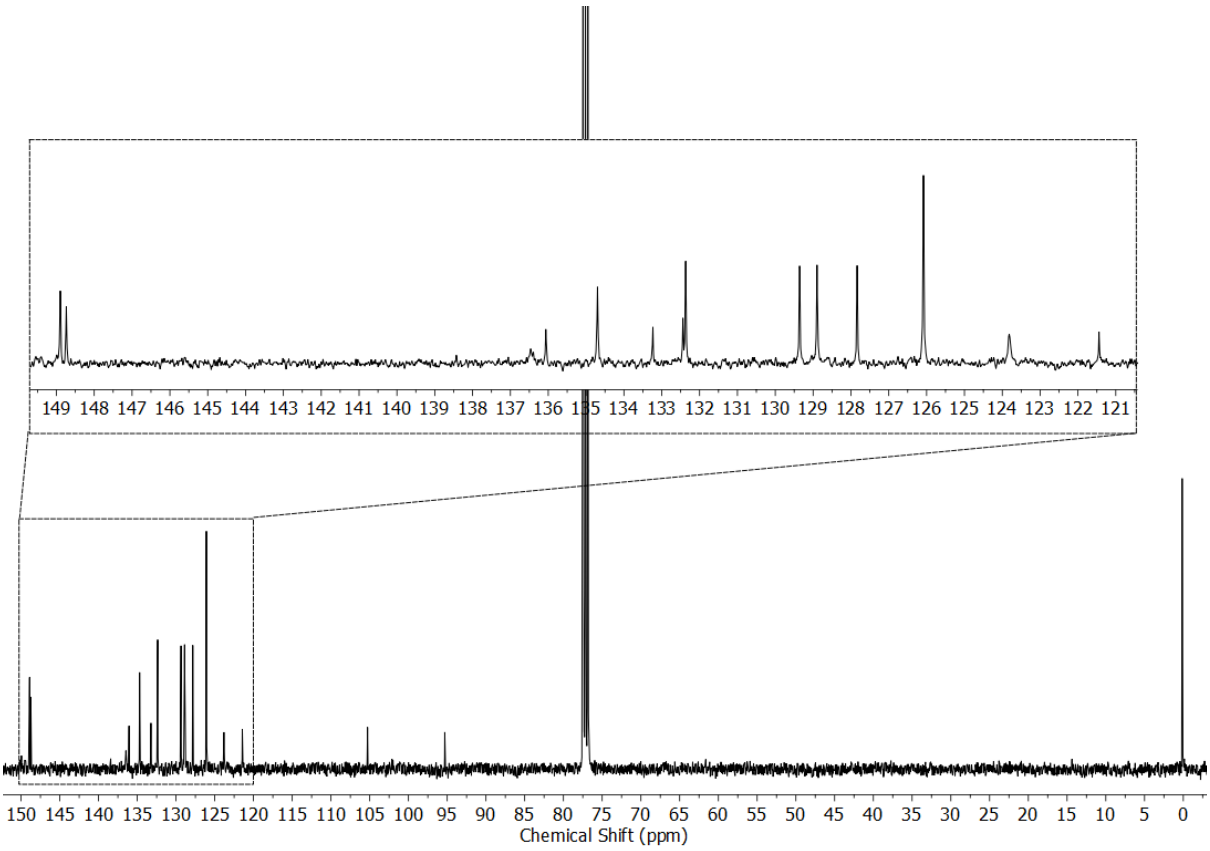

Figure S11 ^{13}C NMR ($CDCl_3$, 101 MHz) of **S2**.

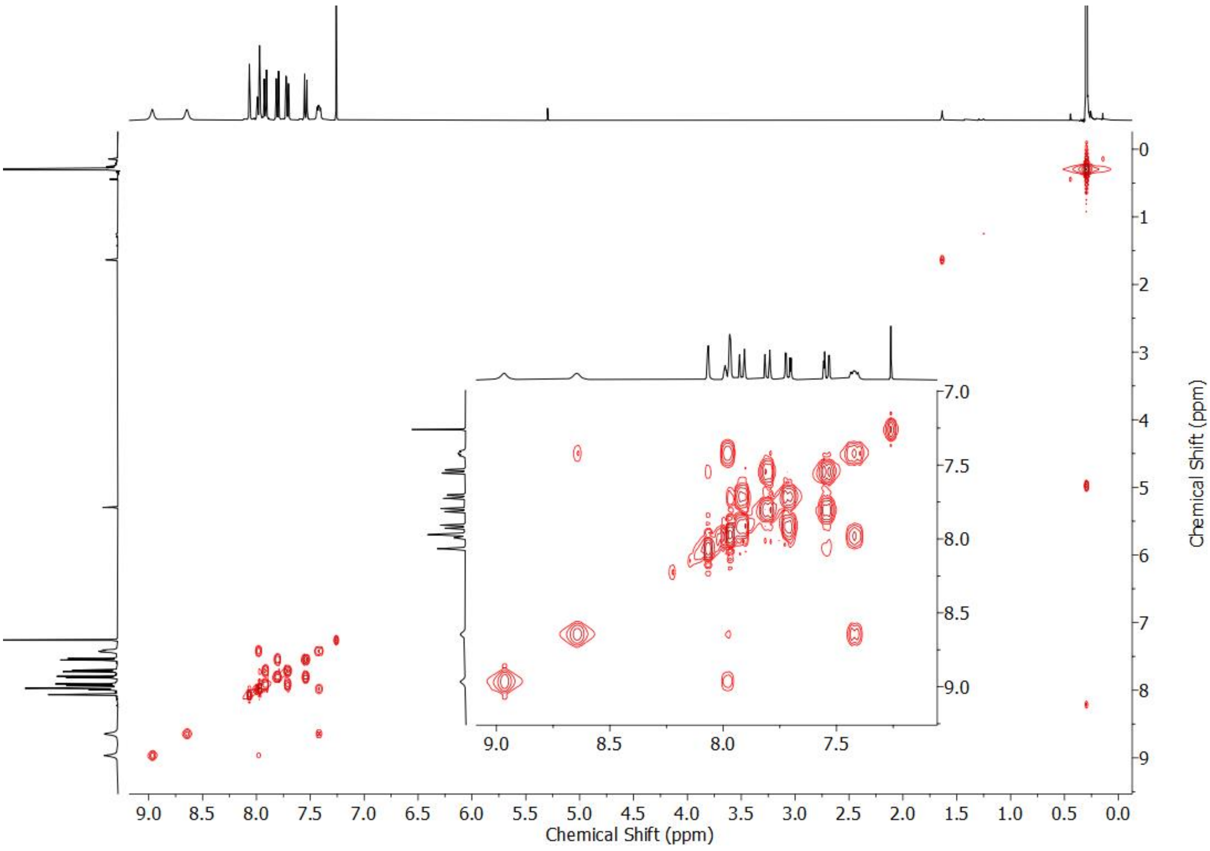

Figure S12 COSY NMR ($CDCl_3$) of **S2**.

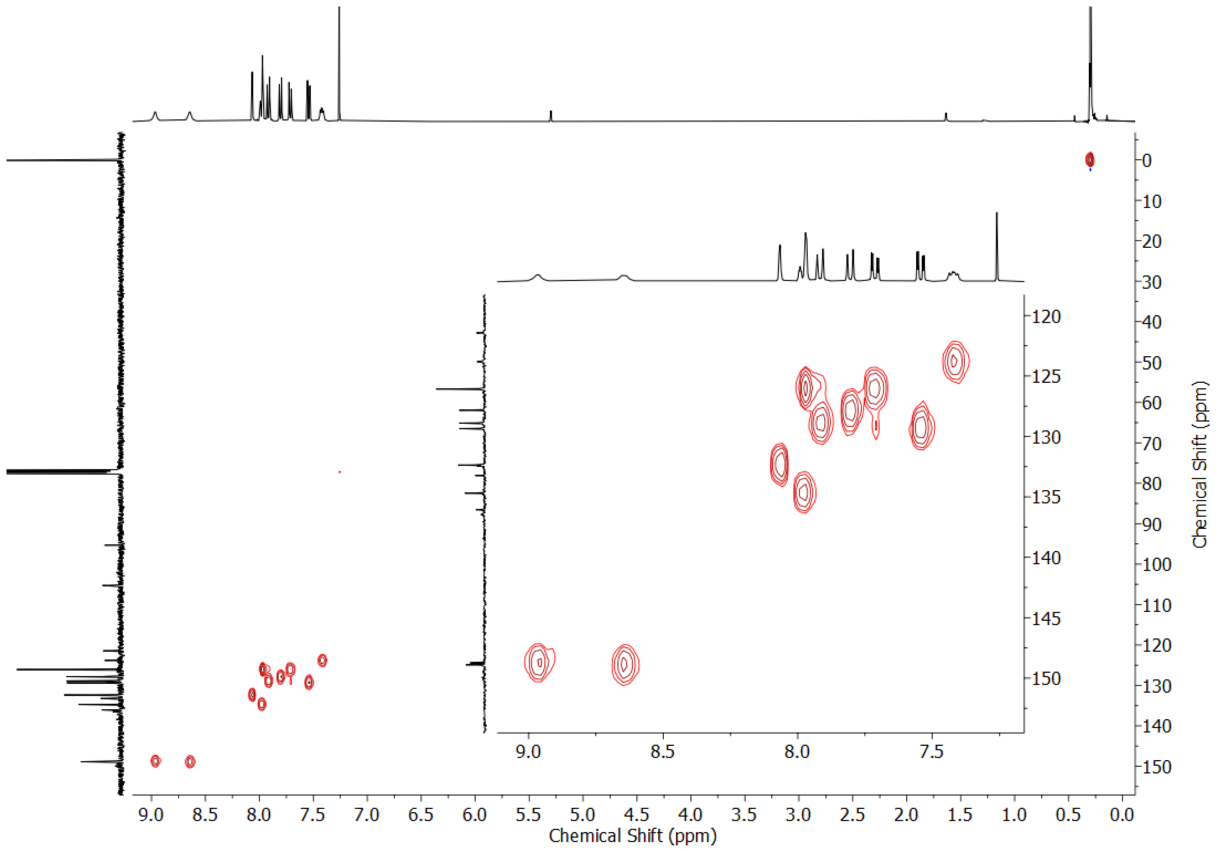

Figure S13 HSQC NMR ($CDCl_3$) of **S2**.

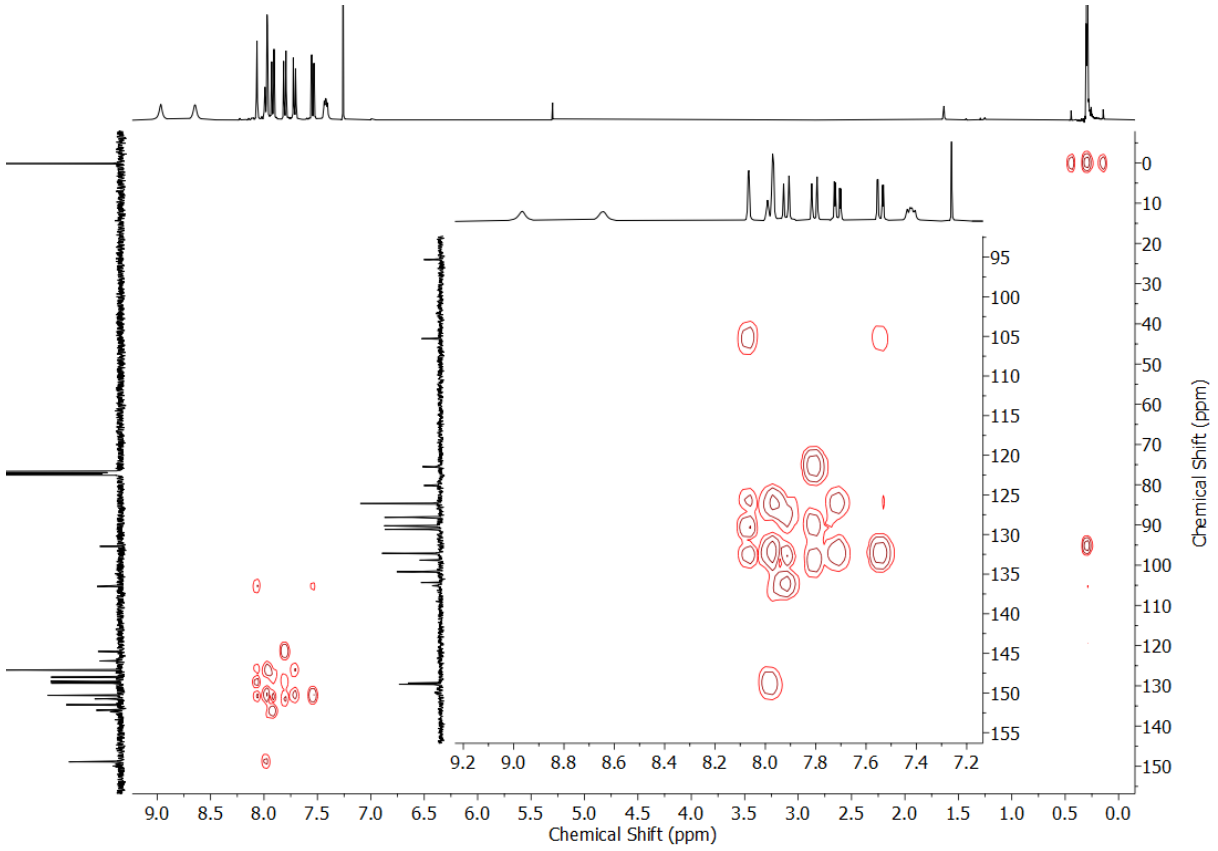

Figure S14 HMBC NMR ($CDCl_3$) of **S2**.

Synthesis of 5A1

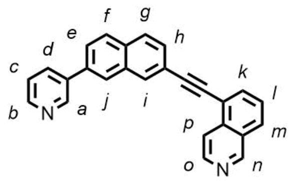

5-Iodoisoquinoline (0.168 g, 0.657 mmol, 1 eq.), **S2** (0.218 g, 0.723 mmol, 1.1 eq.), [Pd(PPh_3)$_2$$Cl_2$] (0.0115 g, 0.0164 mmol, 2.5 mol%), CuI (0.0063 g, 0.033 mmol, 5 mol%) and DBU (0.59 mL, 3.94 mmol, 6 eq.) were stirred at rt in CH_3CN (6.5 mL) for 20 h. EDTA solution (20 mL) was added and the mixture extracted with CH_2Cl_2 (3 × 20 mL). The combined organic extracts were dried ($MgSO_4$) and the solvent removed *in vacuo*. Following column chromatography on silica (step gradient acetone/CH_2Cl_2 10:90 ⟶ 30:70 in 5% increments) the obtained residue was dissolved in CH_2Cl_2 (50 mL) and washed with H_2O (10 mL), sat. aq. $NaHCO_3$ (10 mL), brine (10 mL), dried ($MgSO_4$) and the solvent removed to give the product as a light yellow solid (0.152 g, 65%). ^{1}H NMR (400 MHz, $CDCl_3$) δ: 9.30 (s, 1H, H_n), 9.00 (s, 1H, H_a), 8.69-8.65 (m, 2H, H_b, H_o), 8.25-8.23 (m, 2H, H_i, H_p), 8.05 (d, *J* = 1.8 Hz, 1H, H_j), 8.02-7.96 (m, 4H, H_d, H_f, H_k, H_m), 7.91 (d, *J* = 8.5 Hz, 1H, H_g), 7.75 (dd, *J* = 8.5, 1.8 Hz, 1H, H_e), 7.71 (dd, *J* = 8.4, 1.6 Hz, 1H, H_h), 7.62 (dd, *J* = 8.3, 7.2 Hz, 1H, H_l), 7.43 (m, 1H, H_c). ^{13}C NMR (101 MHz, $CDCl_3$) δ: 152.9, 148.9, 148.7, 144.2, 136.4, 136.3, 136.2, 134.7, 134.3, 133.4, 132.6, 132.1, 129.0, 129.0, 128.6, 128.3, 128.2, 127.0, 126.3, 126.1, 123.8, 121.1, 120.4, 119.0, 95.8, 86.9, 77.5, 77.2, 76.8. HR-ESIMS m/z = 357.1385 $[M+H]^+$ calc. 357.1386.

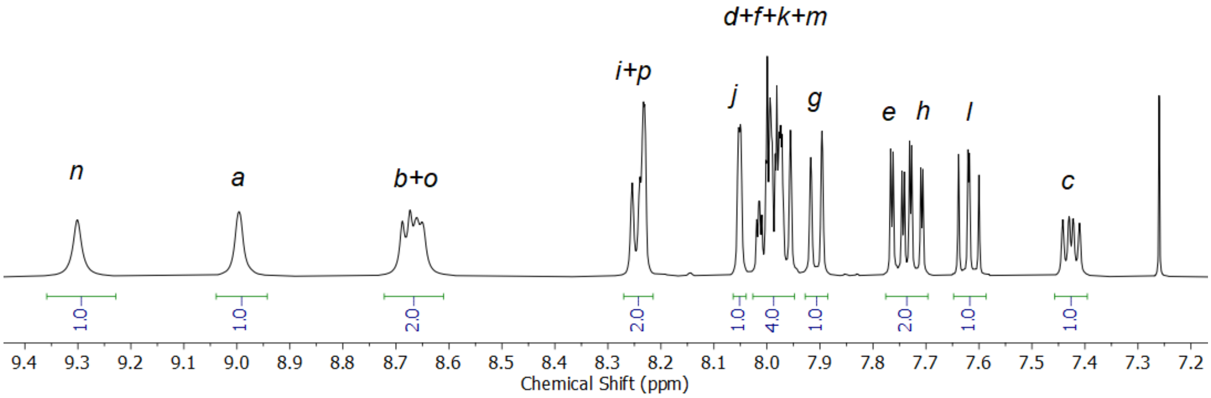

Figure S15 ^{1}H NMR ($CDCl_3$, 400 MHz) of **5A1**.

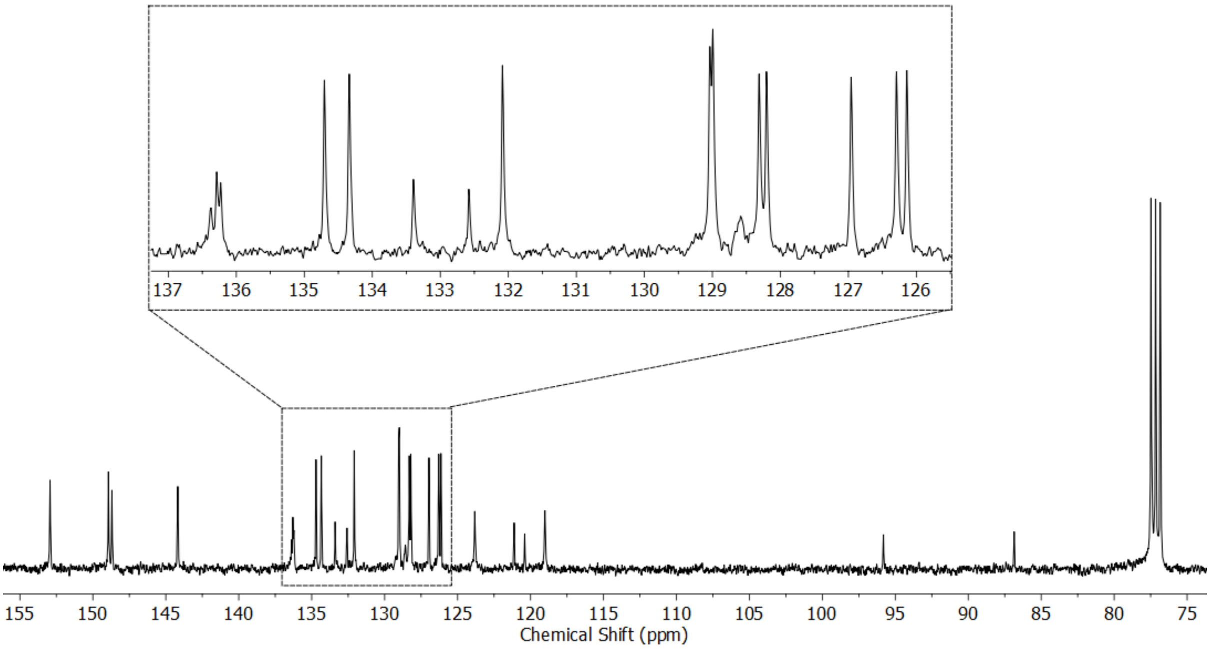

Figure S16 ^{13}C NMR ($CDCl_3$, 101 MHz) of **5A1**.

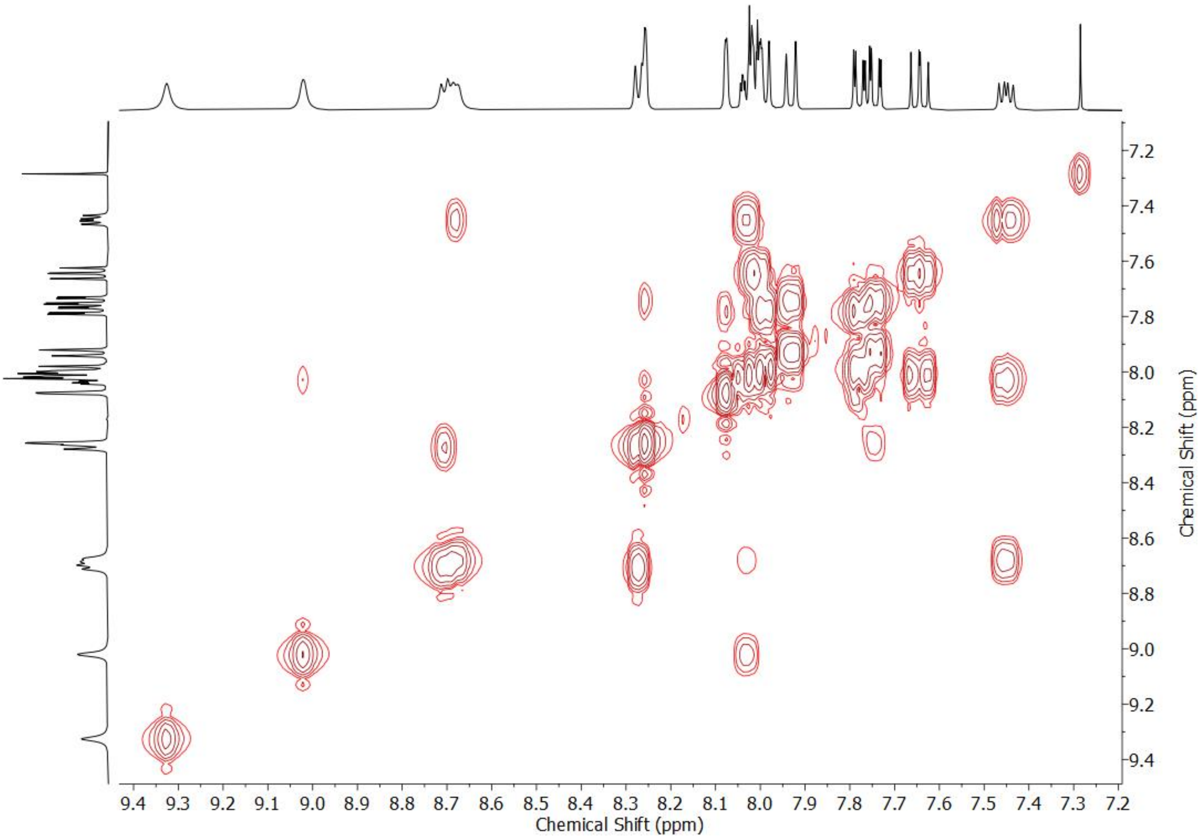

Figure S17 COSY NMR ($CDCl_3$) of **5A1**.

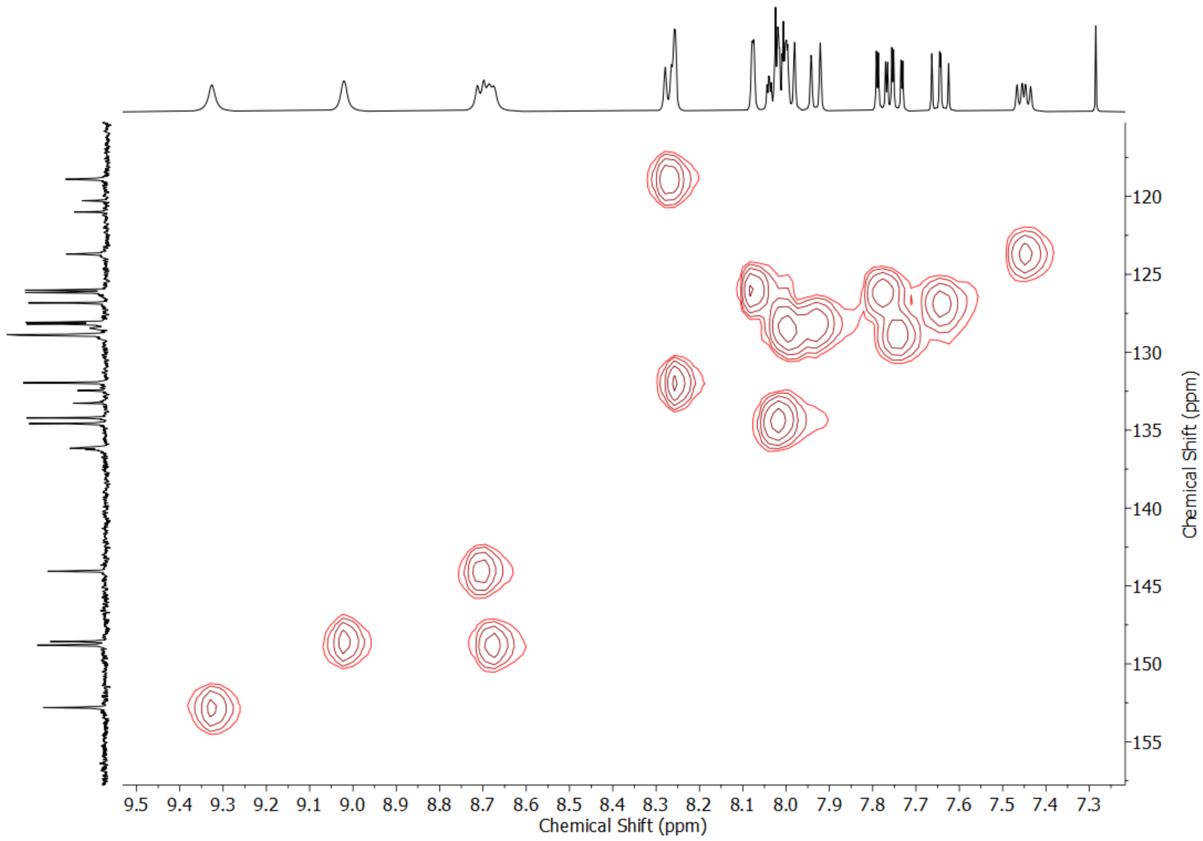

Figure S18 HSQC NMR ($CDCl_3$) of **5A1**.

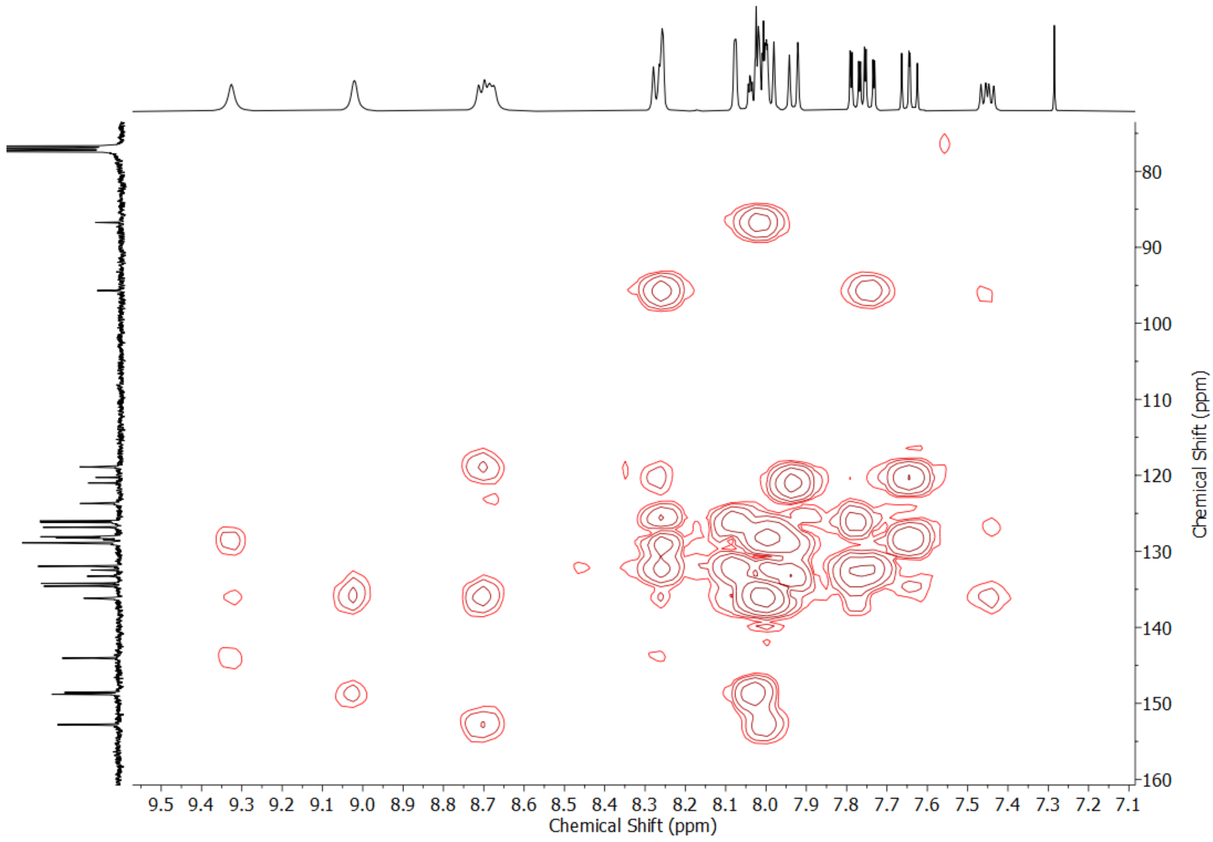

Figure S19 HMBC NMR ($CDCl_3$) of **5A1**.

Synthesis of S3

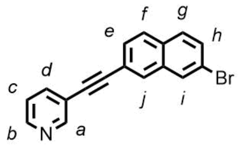

3-Ethynylpyridine (0.206 g, 2.0 mmol, 1.0 eq.), 2,7-dibromonaphthalene (0.858 g, 3.0 mmol, 1.5 eq.), $Pd(PPh_3)_2Cl_2$ (0.035 g, 0.050 mmol, 2.5 mol%) and CuI (0.019 g, 0.10 mmol, 5 mol%) were stirred at 80 °C in $^{i}Pr_2NH$ (15 mL) in a sealed vial for 21 h. EDTA solution (25 mL) was added and the mixture extracted with CH_2Cl_2 (3 × 20 mL). The combined organic extracts were dried ($MgSO_4$) and the solvent removed *in vacuo*. Following purification by column chromatography of silica (1:19 EtOAc/CH_2Cl_2) the product was obtained as a white solid (0.354 g, 57%). ^1H NMR (400 MHz, $CDCl_3$) δ: 8.82 (s, 1H, H_a), 8.58 (d, *J* = 5.1 Hz, 1H, H_b), 7.99-7.97 (m, 2H, H_j, H_i), 7.87-7.84 (m, 1H, H_d), 7.80 (d, *J* = 8.4 Hz, 1H, H_f/H_g), 7.71 (d, *J* = 8.7 Hz, 1H, H_f/H_g), 7.61-7.57 (m, 2H, H_e, H_h), 7.34-7.30 (m, 1H, H_c). ^{13}C NMR (101 MHz, $CDCl_3$) δ: 152.5, 148.9, 138.7, 134.2, 131.5, 130.8, 130.5, 129.9, 129.6, 128.8, 128.3, 123.3, 121.2, 120.9, 120.5, 92.7, 87.2. HR-ESIMS m/z = 308.0070 $[M+H]^+$ calc. 308.0069.

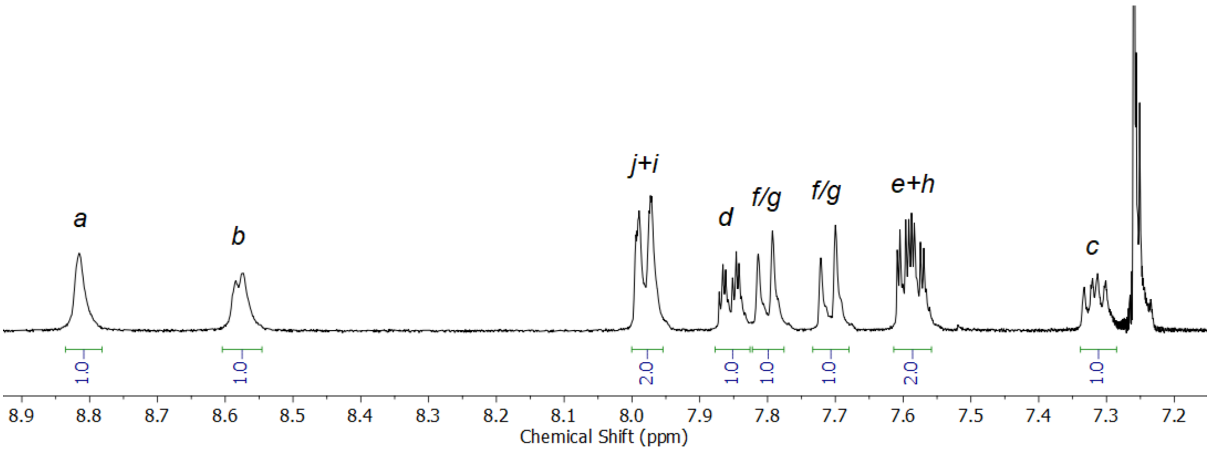

Figure S20 ^1H NMR ($CDCl_3$, 400 MHz) of **S3**.

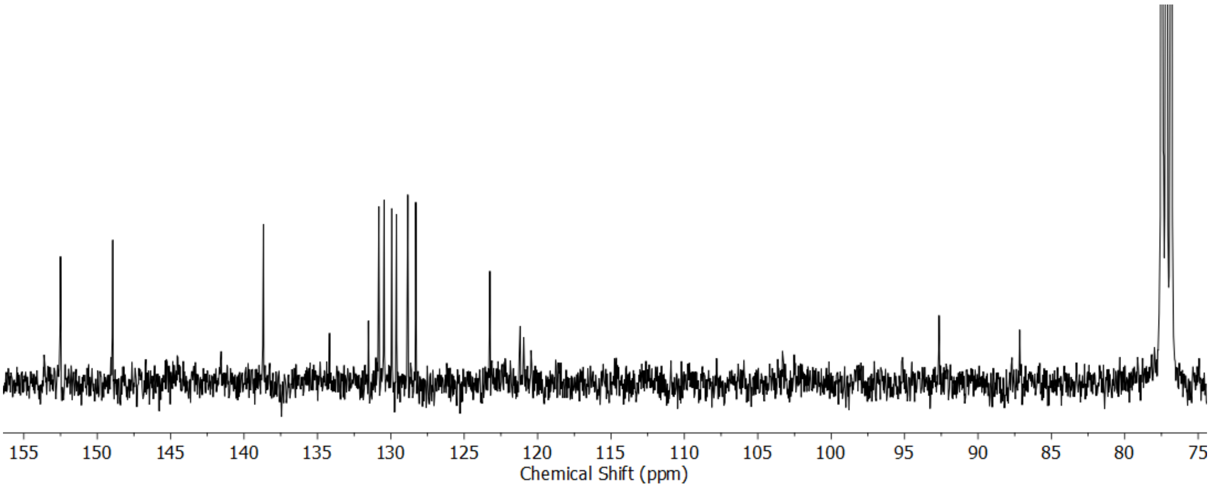

Figure S21 ^{13}C NMR ($CDCl_3$, 101 MHz) of **S3**.

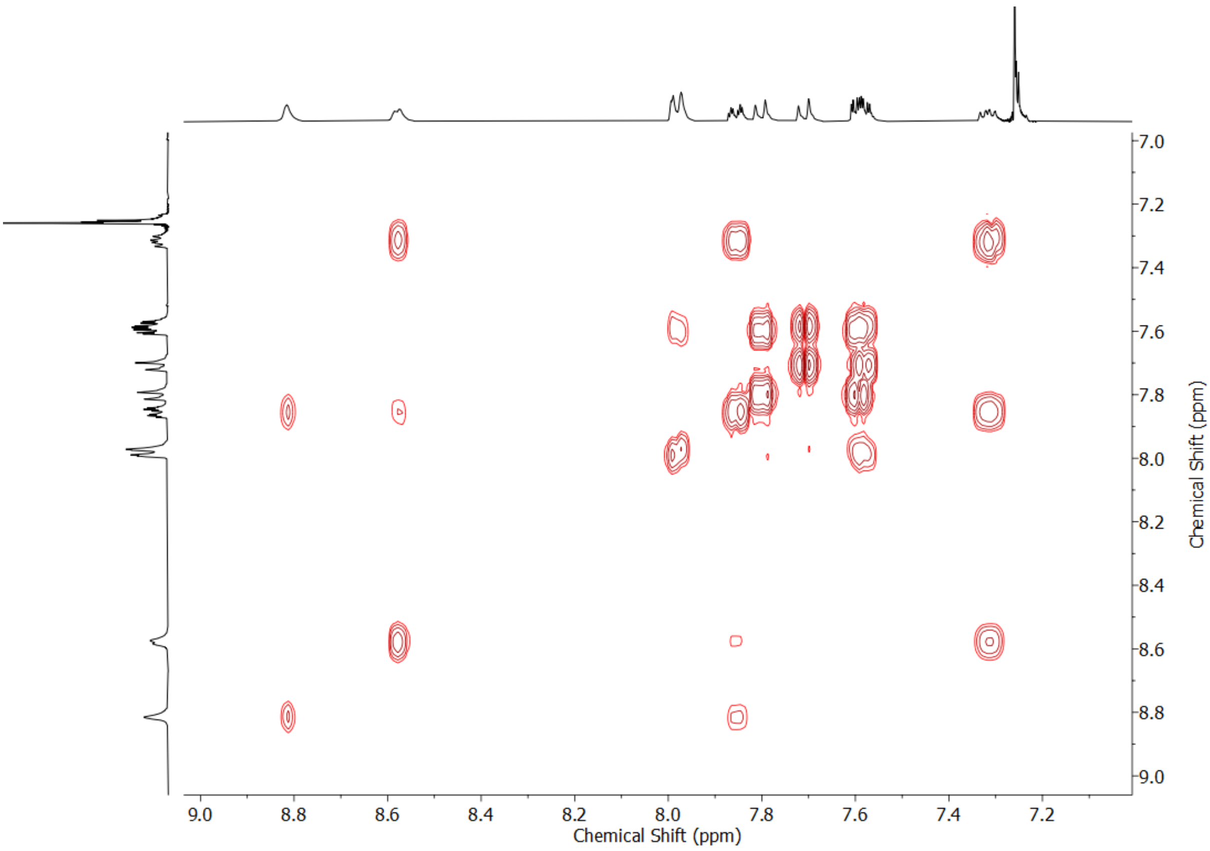

Figure S22 COSY NMR ($CDCl_3$) of **S3**.

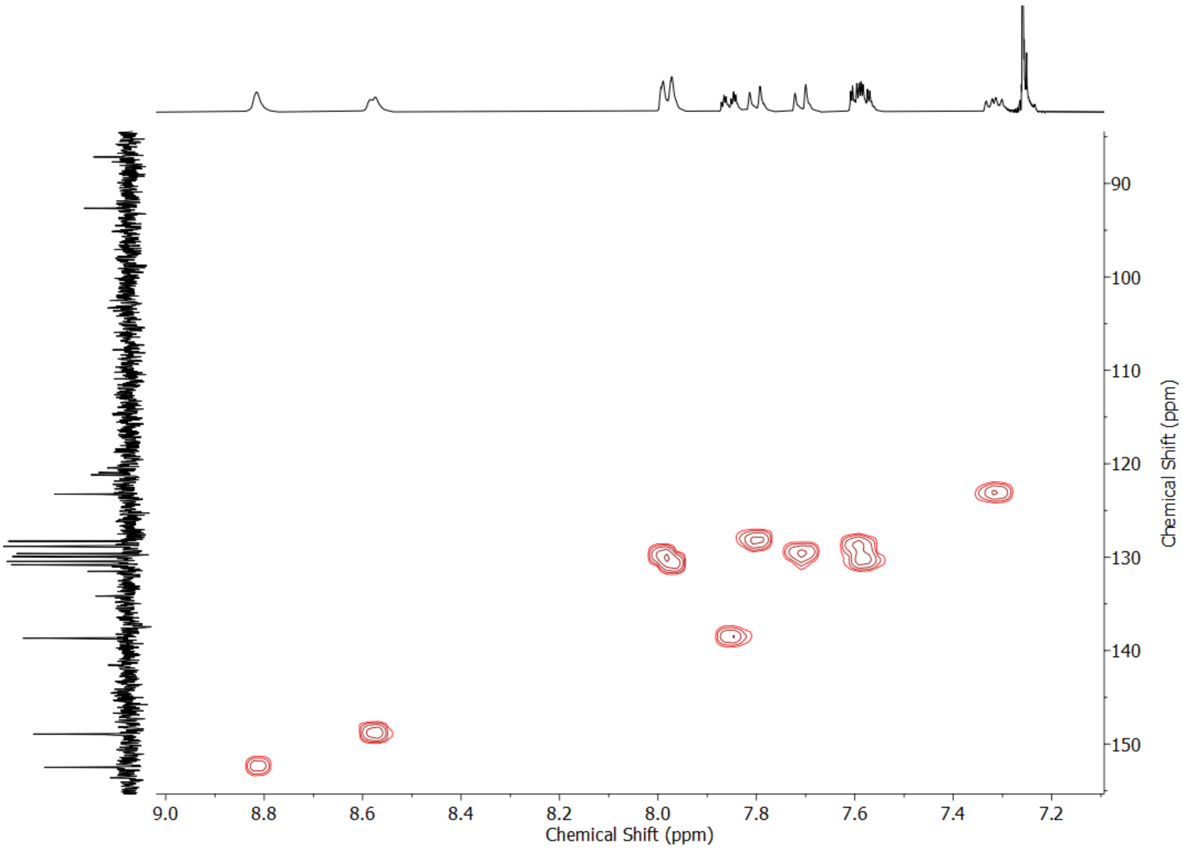

Figure S23 HSQC NMR ($CDCl_3$) of **S3**.

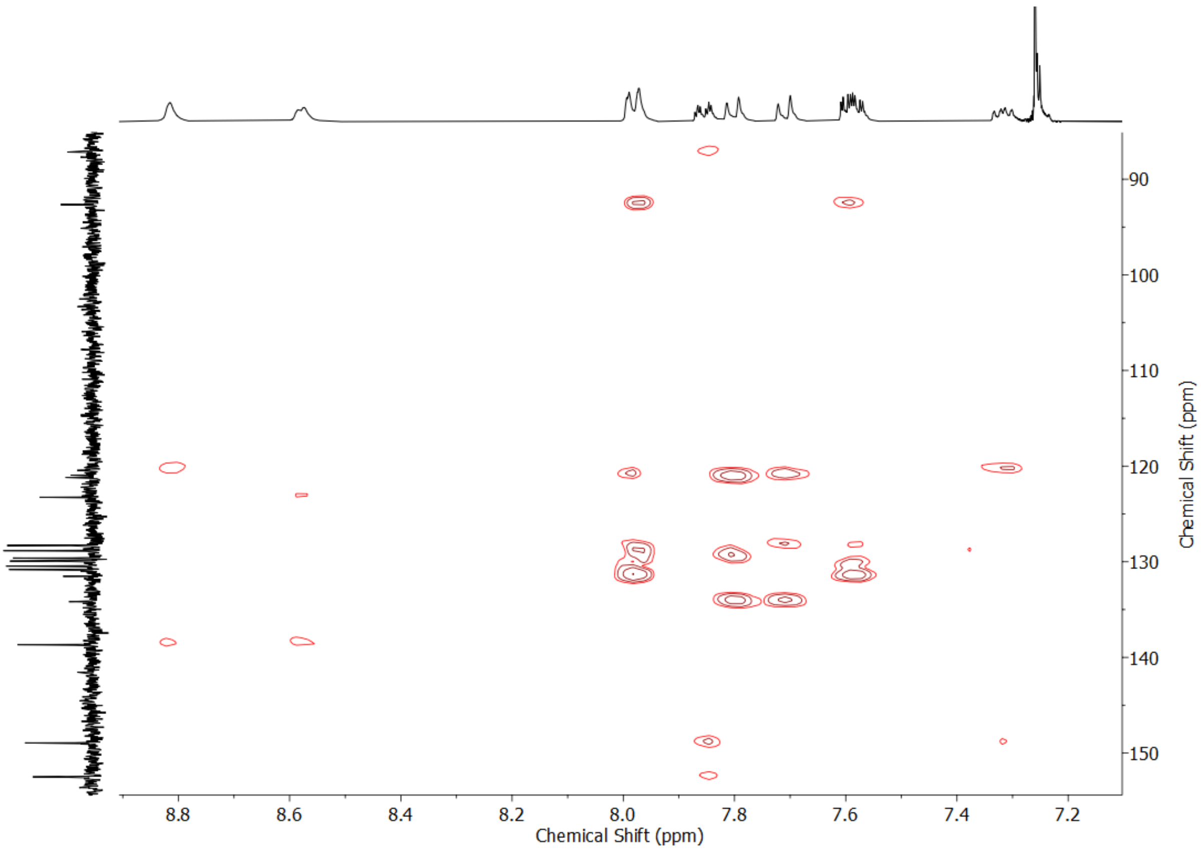

Figure S24 HMBC NMR ($CDCl_3$) of **S3**.

Synthesis of S4

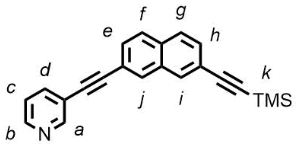

S3 (0.308 g, 1.0 mmol, 1.0 eq.), trimethylsilylacetylene (0.17 mL, 1.2 mmol, 1.2 eq.), $[Pd(PPh_3)_2Cl_2]$ (0.018 g, 0.025 mmol, 2.5 mol%) and CuI (0.010 g, 0.050 mmol, 5 mol%) were stirred at 80 °C in $^{i}Pr_2NH$ (10 mL) in a sealed vial for 18 h. EDTA solution (20 mL) was added and the mixture extracted with CH_2Cl_2 (2 × 20 mL). The combined organic extracts were dried ($MgSO_4$) and the solvent removed *in vacuo*. After purification by column chromatography on silica (1:19 EtOAc/CH_2Cl_2) the product was obtained as an off-white solid (0.318 g, 98%). ^{1}H NMR (400 MHz, $CDCl_3$) δ: 8.81 (br. s, 1H, H_a), 8.59 (br. s, 1H, H_b), 7.99 (d, *J* = 0.8 Hz, 1H, H_i/H_j), 7.97 (d, *J* = 0.8 Hz, 1H, H_i/H_j), 7.86 (br. d, *J* = 7.9 Hz, 1H, H_d), 7.79 (d, *J* = 8.4 Hz, 1H, H_f/H_g), 7.76 (d, *J* = 8.6 Hz, 1H, H_f/H_g), 7.58 (dd, *J* = 8.5, 1.6 Hz, 1H, H_e/H_h), 7.54 (dd, *J* = 8.4 ,1.6 Hz, 1H, H_e/H_h), 7.32 (br. dd, *J* = 7.9, 4.8 Hz, 1H, H_c), 0.29 (s, 9H, H_k). ^{13}C NMR (101 MHz, $CDCl_3$) δ: 152.5, 148.9, 138.6, 132.6, 132.6, 131.9, 131.6, 129.9, 129.2, 128.2, 127.9, 123.2, 121.6, 120.8, 105.1, 95.6, 92.9, 86.9, 0.1. HR-ESIMS m/z = 326.1356 $[M+H]^+$ calc. 326.1360.

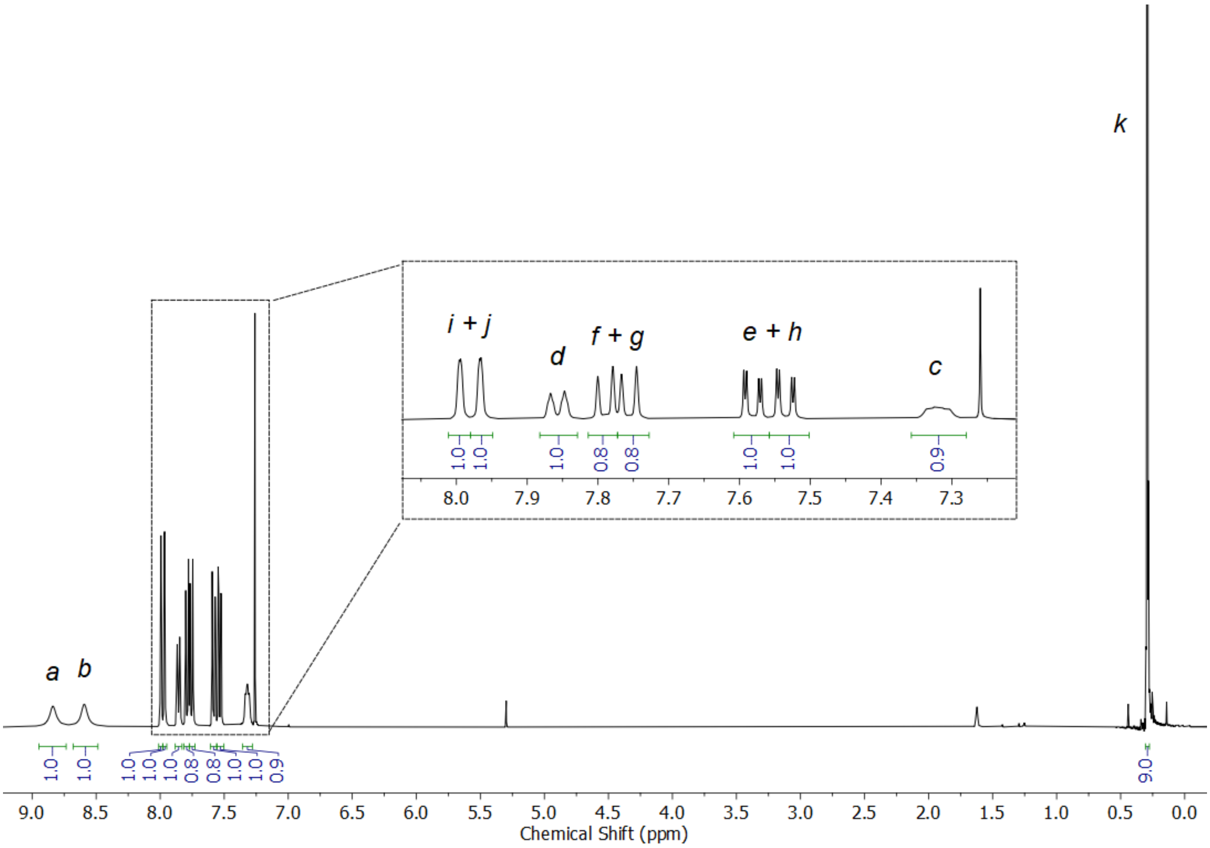

Figure S25 ^{1}H NMR ($CDCl_3$, 400 MHz) of **S4**.

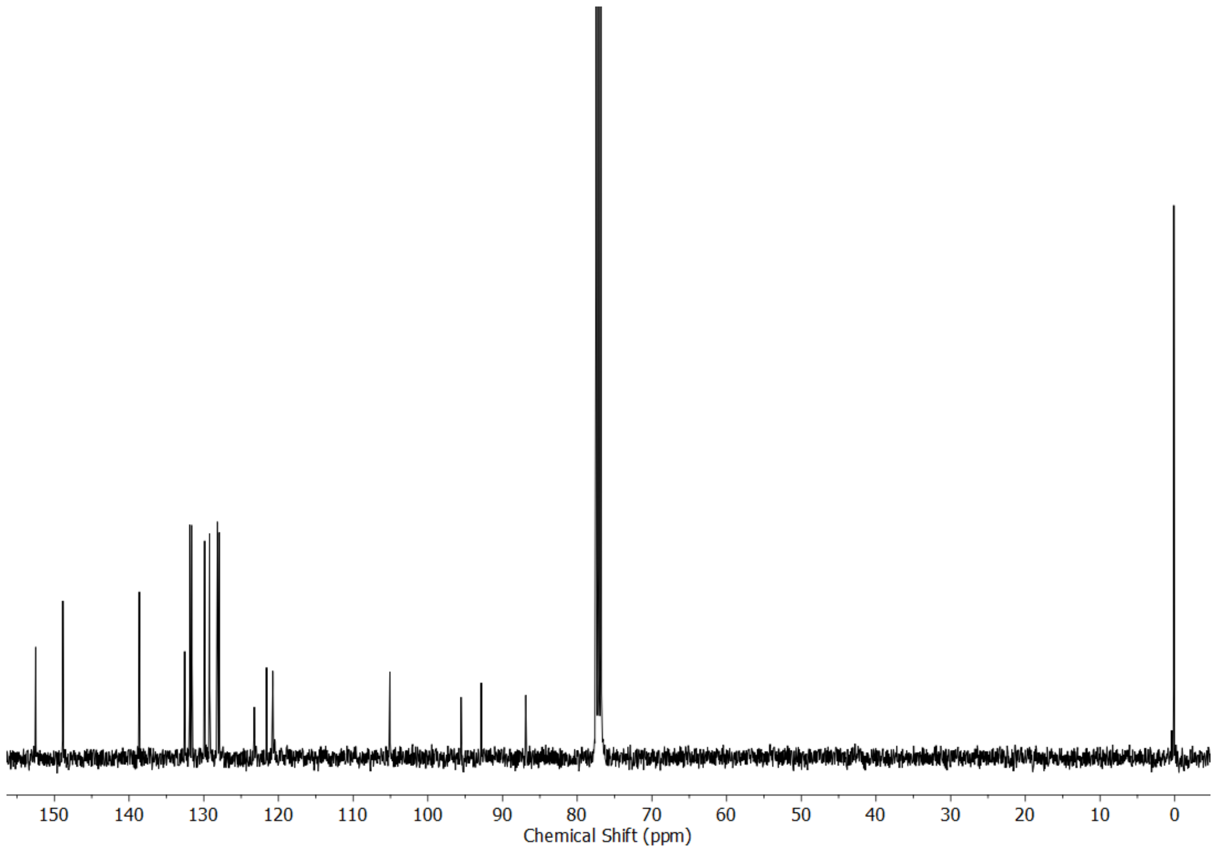

Figure S26 ^{13}C NMR ($CDCl_3$, 101 MHz) of **S4**.

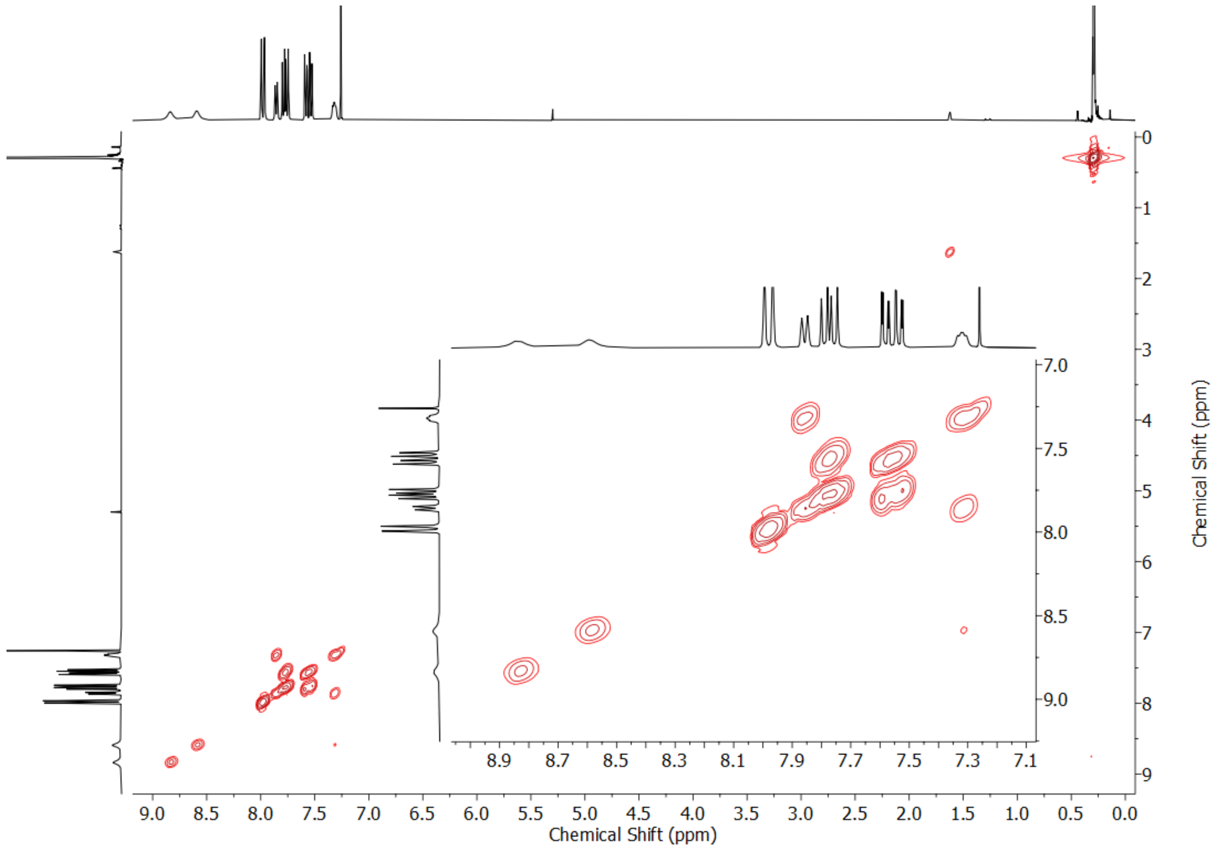

Figure S27 COSY NMR ($CDCl_3$) of **S4**.

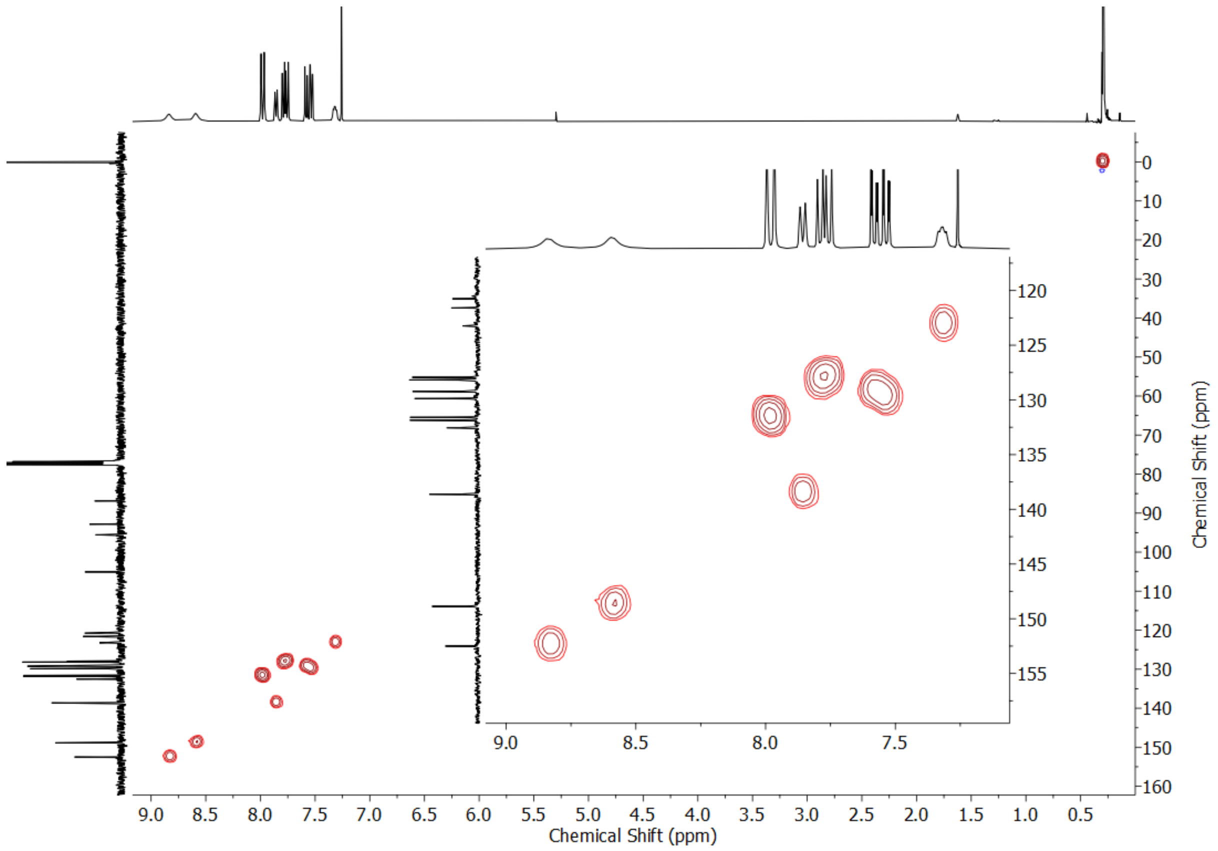

Figure S28 HSQC NMR ($CDCl_3$) of **S4**.

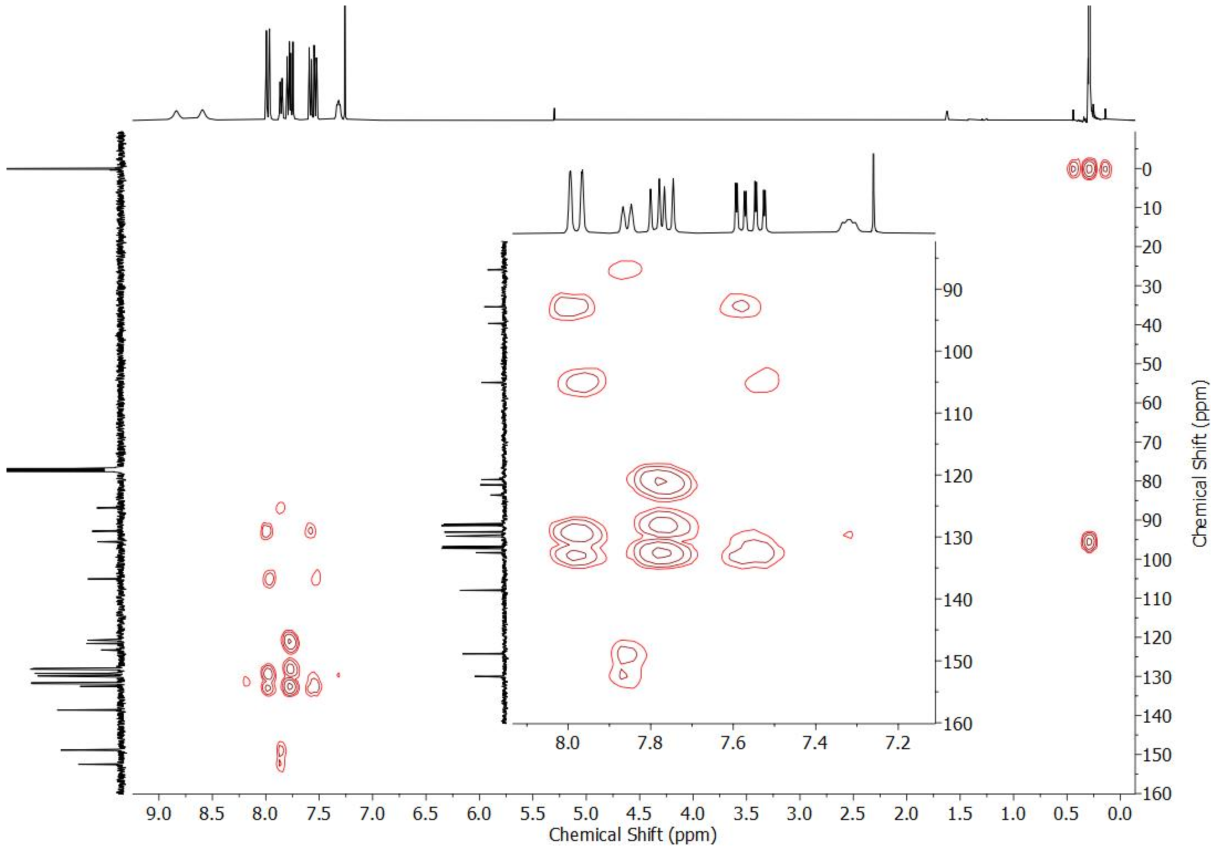

Figure S29 HMBC NMR ($CDCl_3$) of **S4**.

Synthesis of 5A3

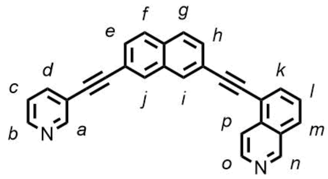

5-Iodoisoquinoline (0.227 g, 0.888 mmol, 1.0 eq.), **S4** (0.318 g, 0.977 mmol, 1.1 eq.), $Pd(PPh_3)_2Cl_2$ (0.016 g, 0.022 mmol, 2.5 mol%), CuI (0.0085 g, 0.044 mmol, 5 mol%) and DBU (0.80 mL, 5.3 mmol, 6.0 eq.) were stirred at rt in CH_3CN (10 mL) for 23 h. EDTA solution (25 mL) was added and the reaction mixture extracted with CH_2Cl_2 (3 × 25 mL). The combined organic extracts were dried ($MgSO_4$) and the solvent removed *in vacuo*. Following purification by column chromatography on silica (step gradient acetone/CH_2Cl_2 0:100 ⟶ 20:80 in 5% increments) the product was obtained as a light yellow solid (0.120 g, 36%). ^{1}H NMR (400 MHz, $CDCl_3$) δ: 9.30 (s, 1H, H_n), 8.83 (d, J = 1.2 Hz, 1H, H_a), 8.68 (d, J = 5.8 Hz, 1H, H_o), 8.58 (dd, J = 4.9, 1.7 Hz, 1H, H_b), 8.23 (d, J = 5.8 Hz, 1H, H_p), 8.13 (d, J = 0.7 Hz, 1H, H_i/H_j), 8.08 (d, J = 0.8 Hz, 1H, H_i/H_j), 8.00-7.97 (m, 2H, H_k, H_m), 7.88-7.83 (m, 3H, H_d, H_f, H_g), 7.71 (dd, J = 8.5, 1.6 Hz, 1H, H_e/H_h), 7.64-7.60 (m, 2H, H_e/H_h, H_l), 7.31 (ddd, J = 7.9, 4.9, 0.9 Hz, 1H, H_c). ^{13}C NMR (101 MHz, $CDCl_3$) δ: 152.8, 152.3, 148.7, 144.1, 138.5, 136.0, 134.2, 132.6, 132.5, 131.5, 131.4, 129.4, 129.3, 128.4, 128.2, 128.1, 128.1, 126.8, 123.1, 121.1, 120.8, 120.3, 120.1, 118.8, 95.4, 92.6, 86.9, 86.8. HR-ESIMS m/z = 381.1384 $[M+H]^+$ calc. 381.1386.

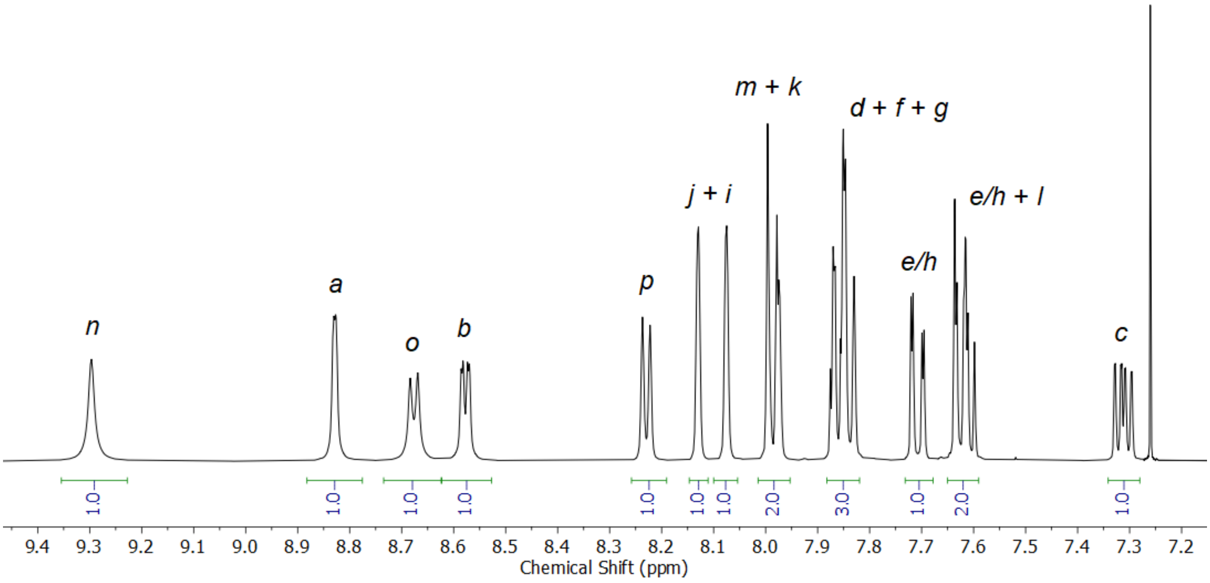

Figure S30 ^{1}H NMR ($CDCl_3$, 400 MHz) of **5A3**.

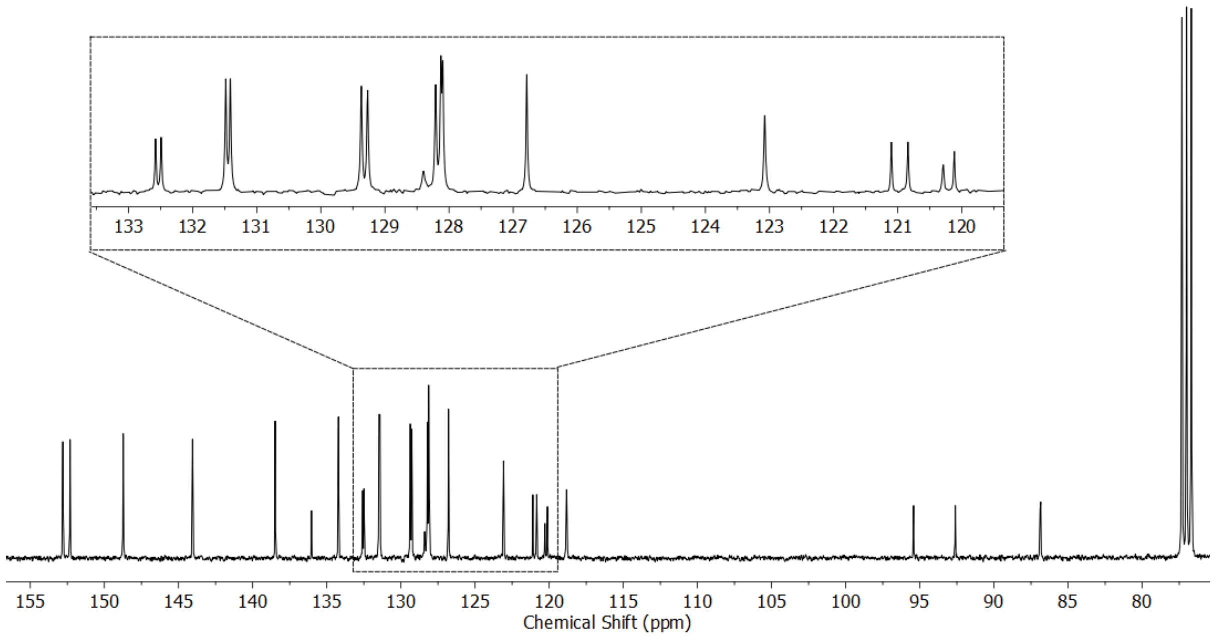

Figure S31 ^{13}C NMR ($CDCl_3$, 101 MHz) of **5A3**.

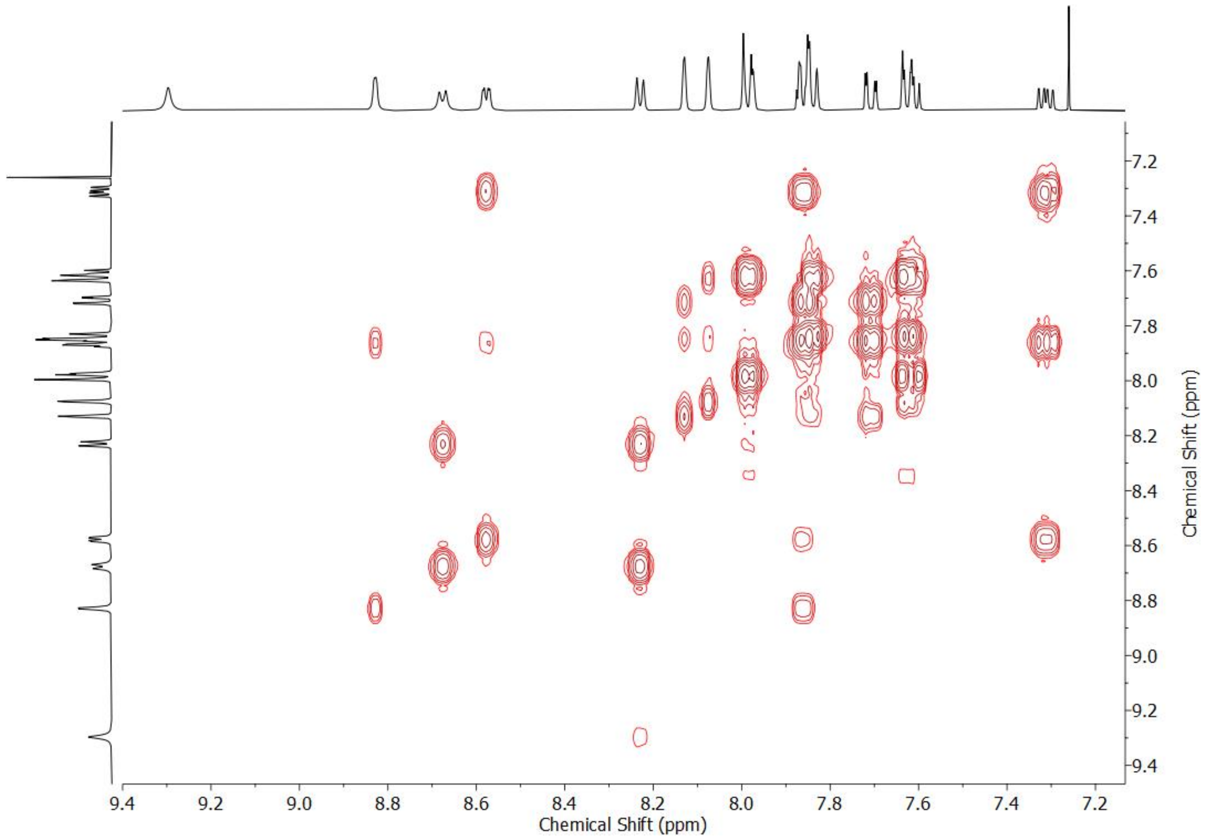

Figure S32 COSY NMR ($CDCl_3$) of **5A3**.

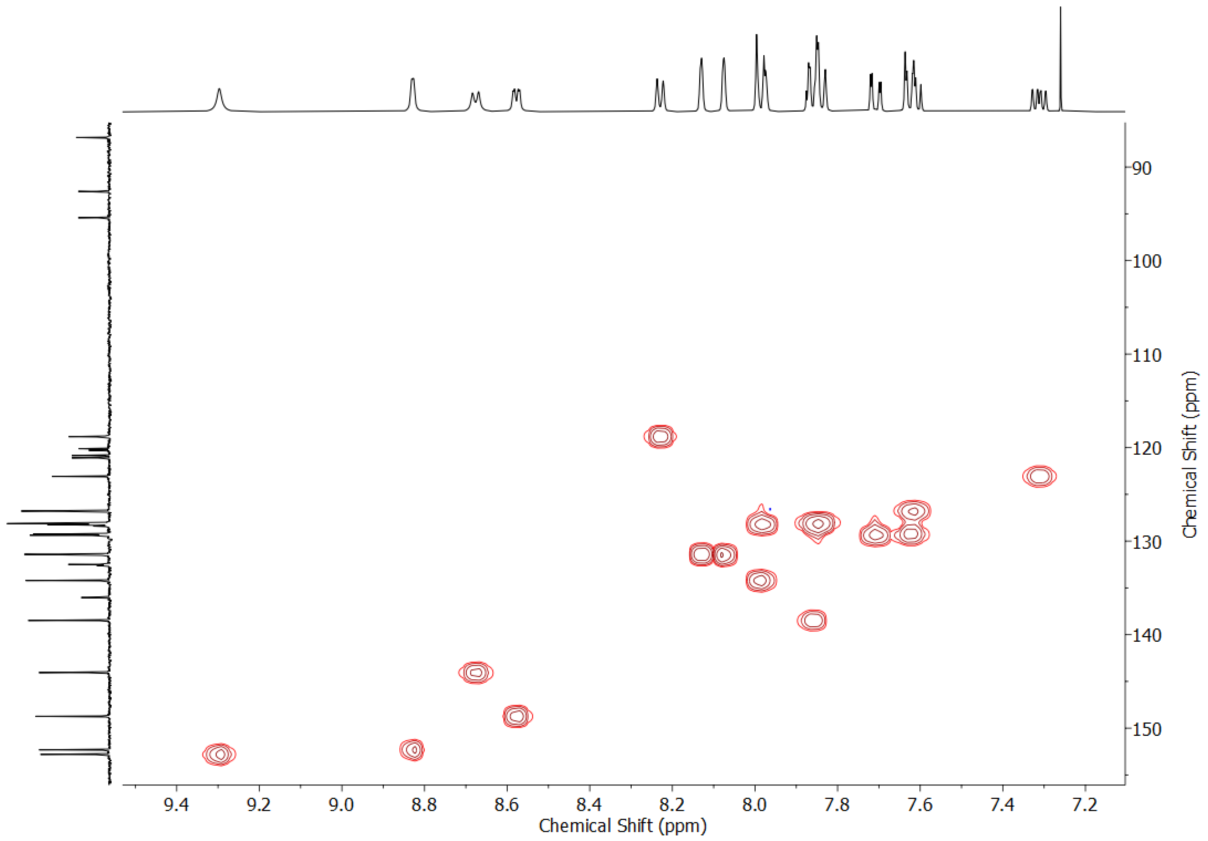

Figure S33 HSQC NMR ($CDCl_3$) of **5A3**.

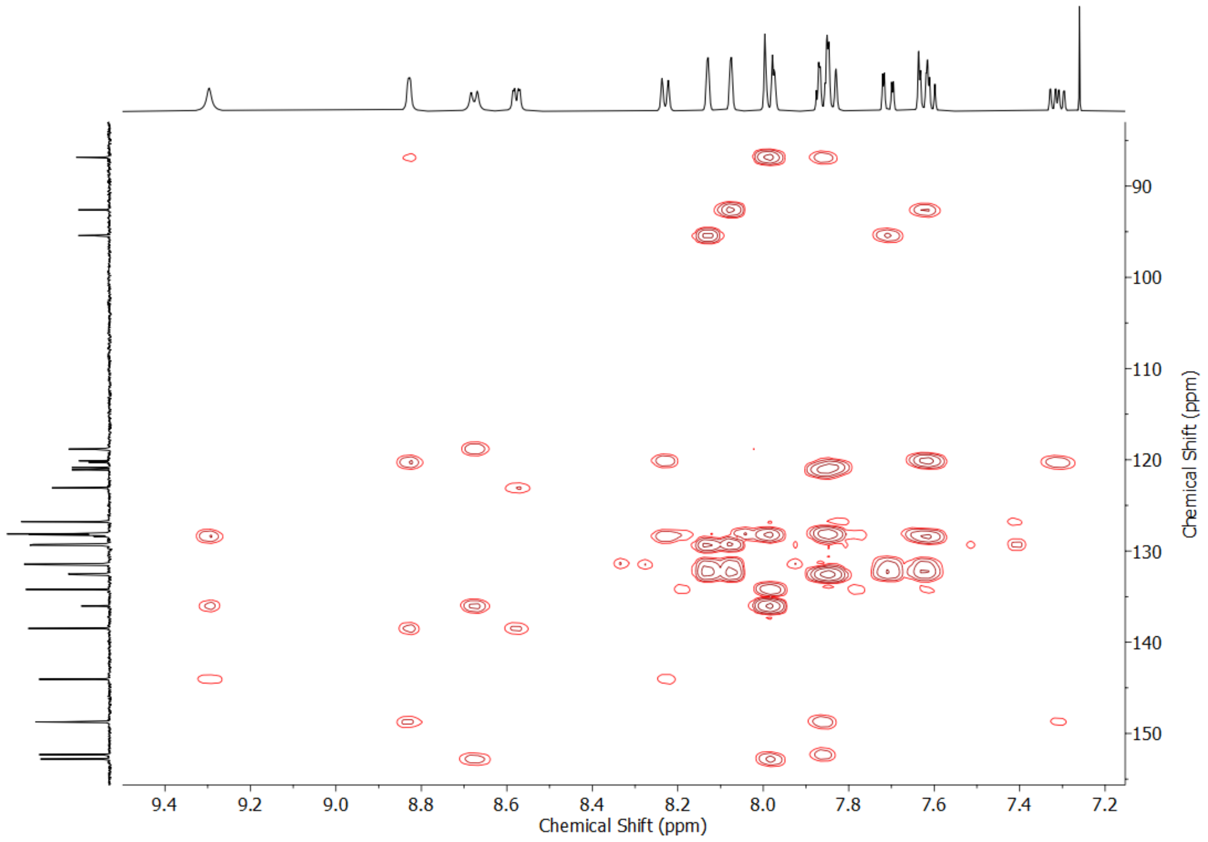

Figure S34 HMBC NMR ($CDCl_3$) of **5A3**.

Synthesis of S5

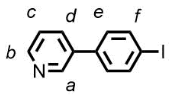

3-Pyridylboronic acid (0.369 g, 3.0 mmol, 1.0 eq.), 1,4-diiodobenzene (1.98 g, 6.0 mmol, 2.0 eq.), $Pd(PPh_3)_2Cl_2$ (0.053 g, 0.075 mmol, 2.5 mol%) and K_2CO_3 (1.04 g, 7.5 mmol, 2.5 eq.) in 2:1 dioxane/H_2O (18 mL) were stirred at 80 °C in a sealed vial for 17 h. EDTA solution (25 mL) was added and the mixture extracted with CH_2Cl_2 (2 × 25 mL). The combined organic extracts were dried ($MgSO_4$) and the solvent removed *in vacuo*. After purification by column chromatography on silica (1:9 EtOAc/CH_2Cl_2) the product was obtained as an orange solid (0.167 g, 20%). ^{1}H NMR (400 MHz, $CDCl_3$) δ: 8.82 (dd, *J* = 2.4, 0.9 Hz, 1H, H_a), 8.61 (dd, *J* = 4.8, 1.6 Hz, 1H, H_b), 7.86-7.81 (m, 3H, H_d, H_f), 7.37 (ddd, *J* = 7.9, 4.8, 0.9 Hz, 1H, H_c), 7.32 (d, *J* = 8.5 Hz, 2H, H_e). ^{13}C NMR (101 MHz, $CDCl_3$) δ: 149.0, 148.2, 138.4, 137.5, 135.8, 134.3, 129.1, 123.8, 94.2. HR-ESIMS m/z = 281.9771 $[M+H]^+$ calc. 281.9774.

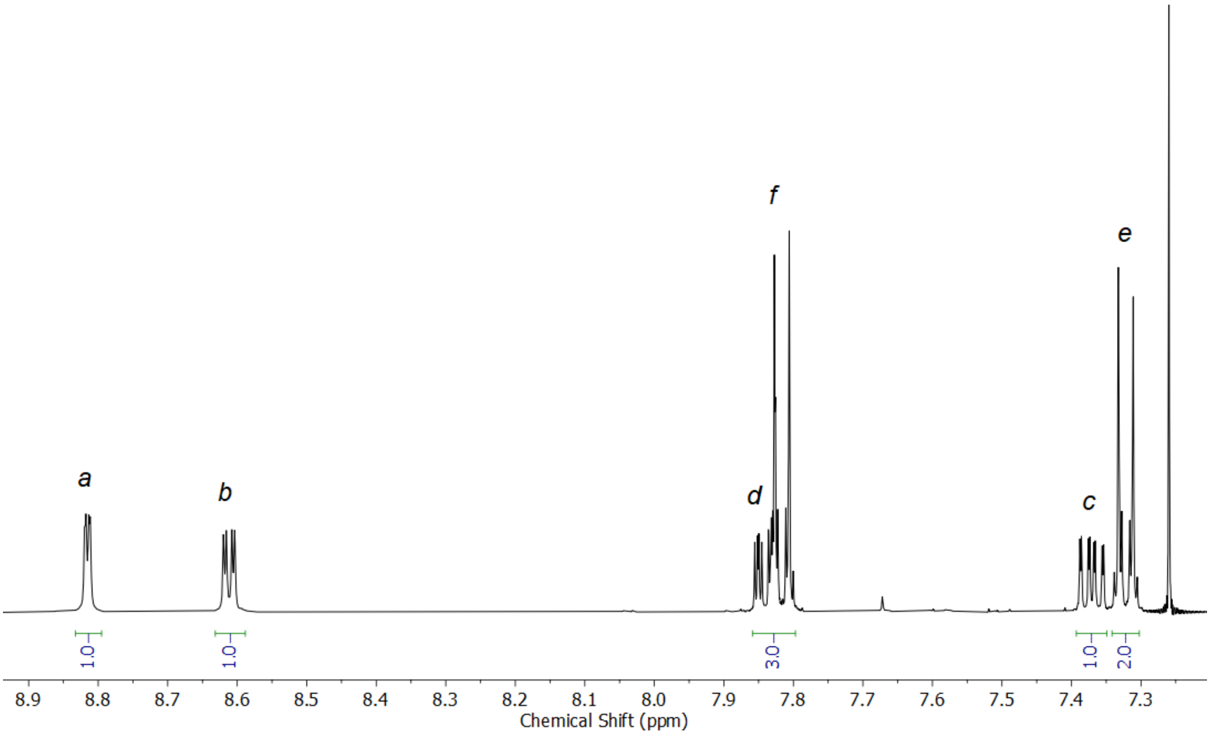

Figure S35 ^{1}H NMR ($CDCl_3$, 400 MHz) of **S5**.

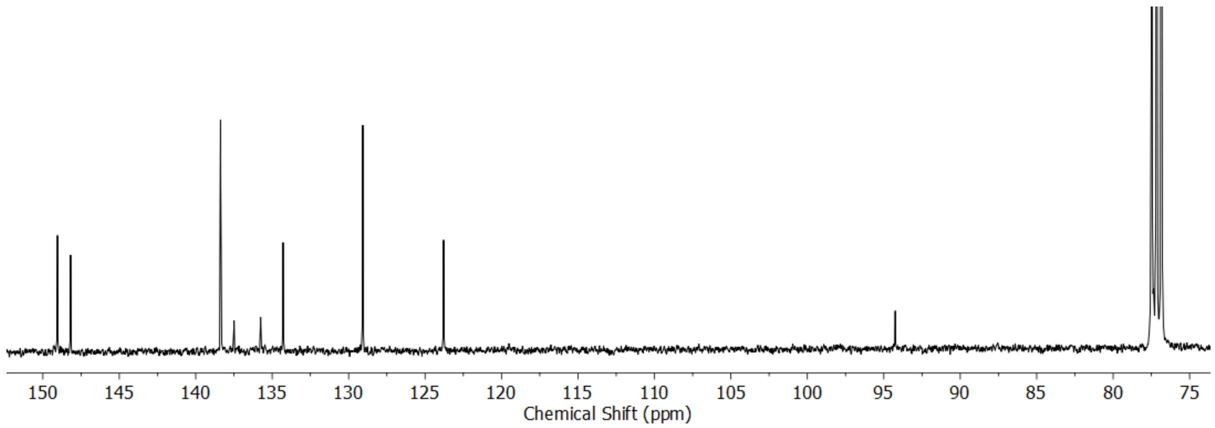

Figure S36 ^{13}C NMR ($CDCl_3$, 101 MHz) of **S5**.

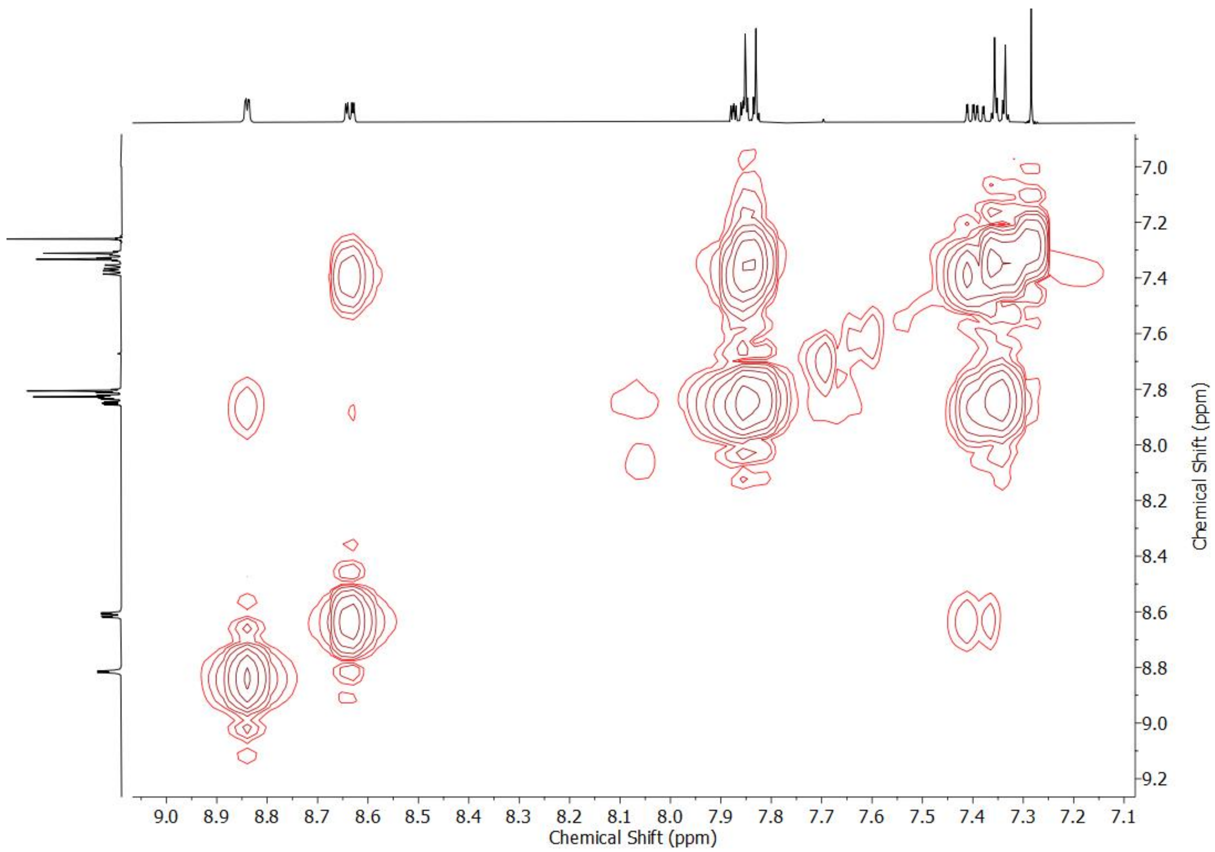

Figure S37 COSY NMR ($CDCl_3$) of **S5**.

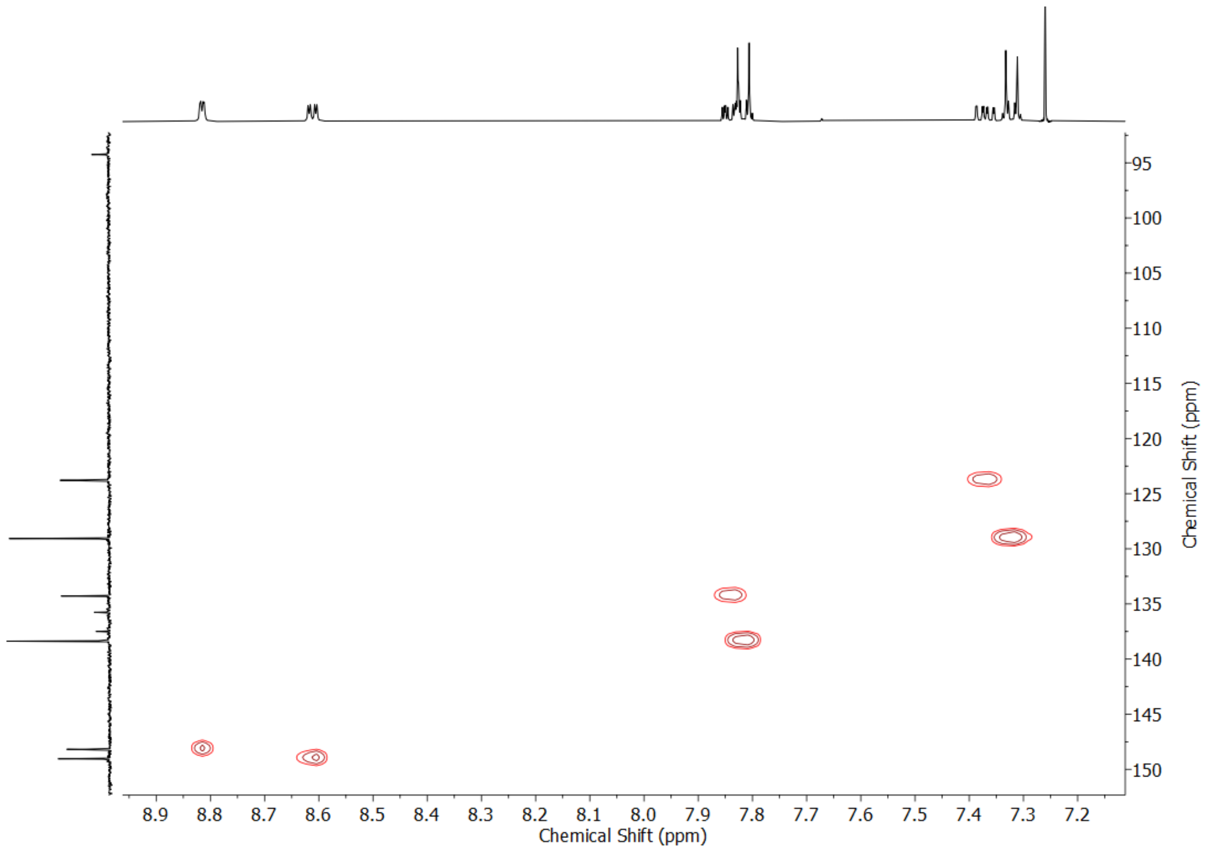

Figure S38 HSQC NMR ($CDCl_3$) of **S5**.

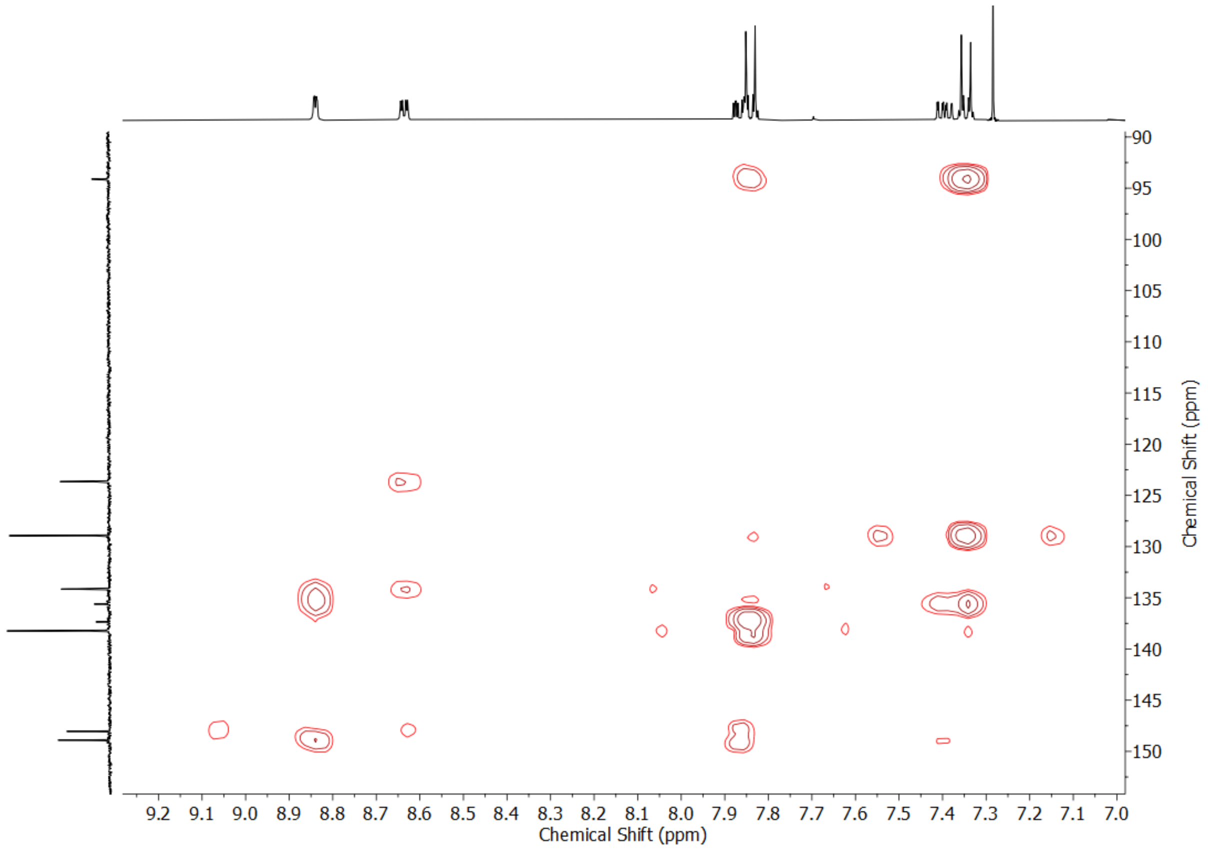

Figure S39 HMBC NMR ($CDCl_3$) of **S5**.

Synthesis of S6

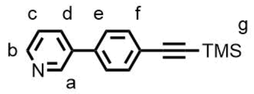

S5 (0.180 g, 0.640 mmol, 1 eq.), trimethylsilylacetylene (0.11 mL, 0.77 mmol, 1.2 eq.), $Pd(PPh_3)_2Cl_2$ (0.0112 g, 0.0160 mmol, 2.5 mol%) and CuI (0.0061 g, 0.032 mmol, 5 mol%) were stirred at rt in $^{i}Pr_2NH$ (6 mL) for 21 h. EDTA solution (25 mL) was added and the aqueous phase extracted with CH_2Cl_2 (2 × 15 mL). The combined organic phases were dried ($MgSO_4$) and the solvent removed *in vacuo*. After purification by column chromatography on silica (1:9 EtOAc/CH_2Cl_2) the product was obtained as a brown solid (0.160 g, 99%). ^{1}H NMR (400 MHz, $CDCl_3$) δ: 8.84 (br. s, 1H, H_a), 8.60 (br. d, J = 4.2 Hz, 1H, H_b), 7.88, (ddd, J = 7.9, 2.4, 1.6 Hz, 1H, H_d), 7.57 (d, J = 8.6 Hz, 2H, H_f), 7.52 (d, J = 8.6 Hz, 2H, H_e), 7.38 (m, 1H, H_c), 0.27 (s, 9H, H_g). ^{13}C NMR (101 MHz, $CDCl_3$) δ: 148.7, 148.1, 137.7, 136.1, 134.5, 132.8, 127.0, 123.8, 123.2, 104.6, 95.8, 0.1. HR-ESIMS m/z = 252.1211 $[M+H]^+$ calc. 252.1209.

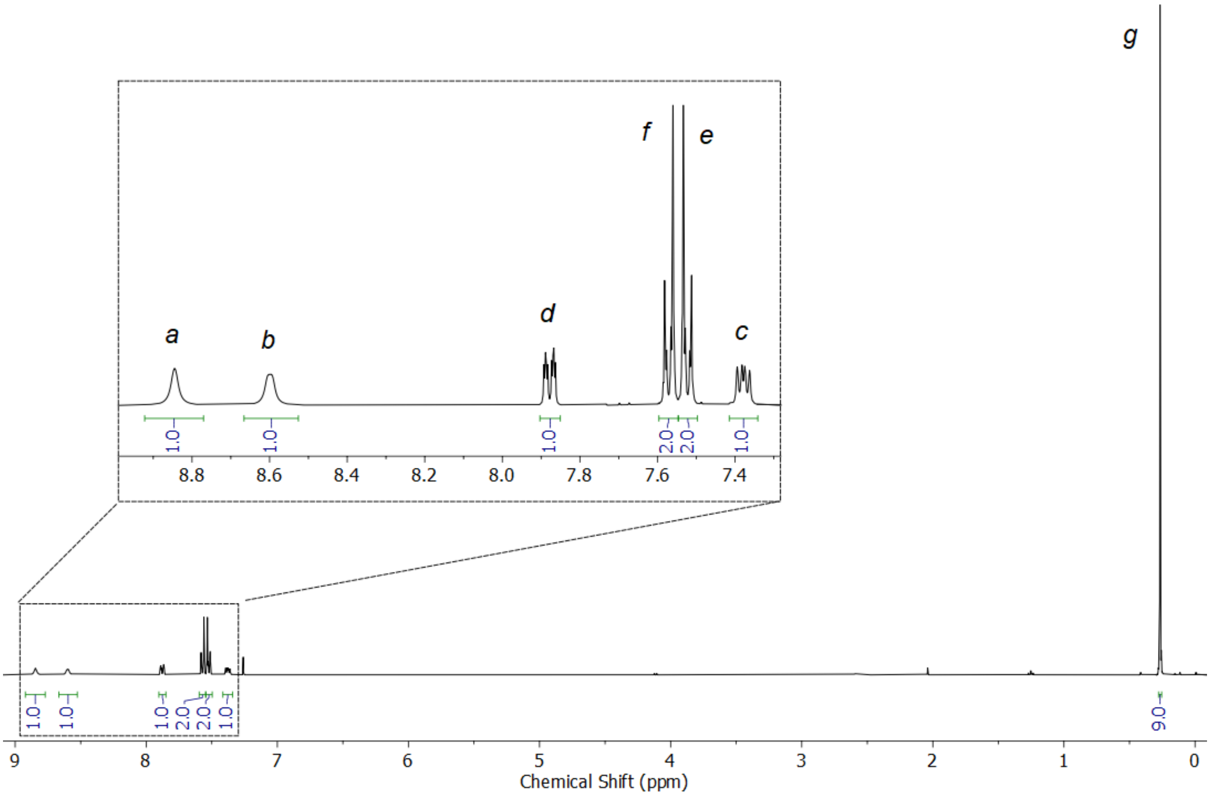

Figure S40 ^{1}H NMR ($CDCl_3$, 400 MHz) of **S6**.

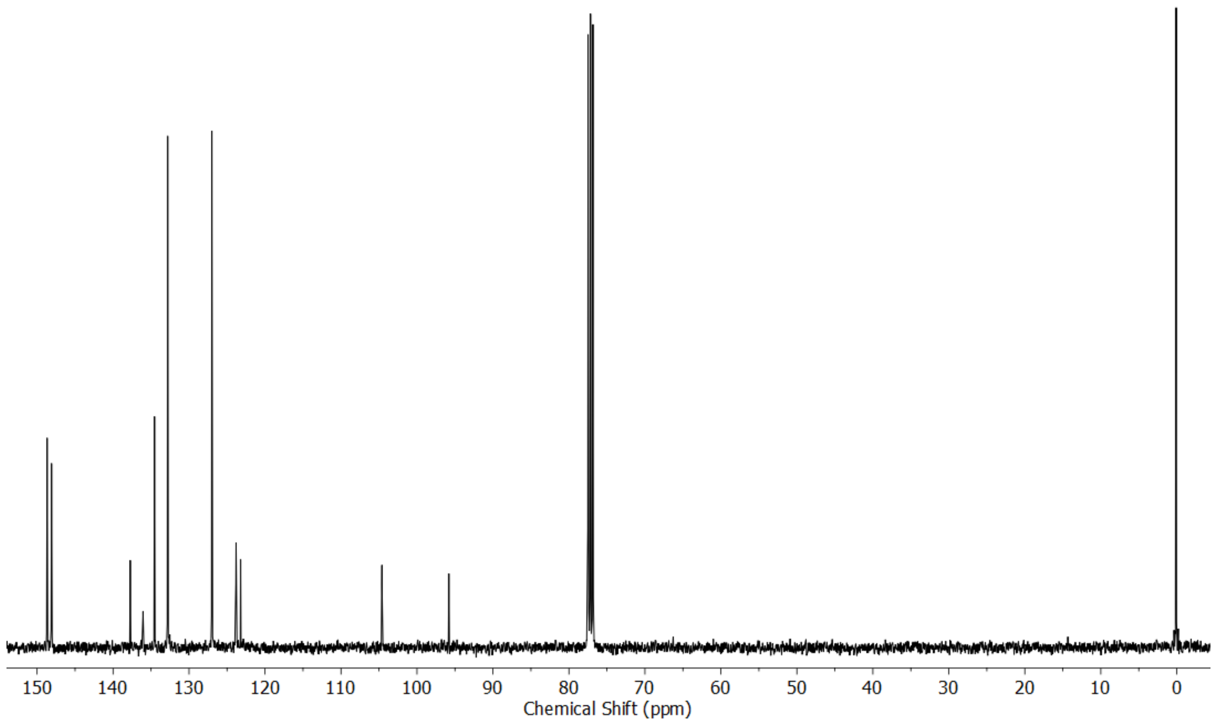

Figure S41 ^{13}C NMR ($CDCl_3$, 101 MHz) of **S6**.

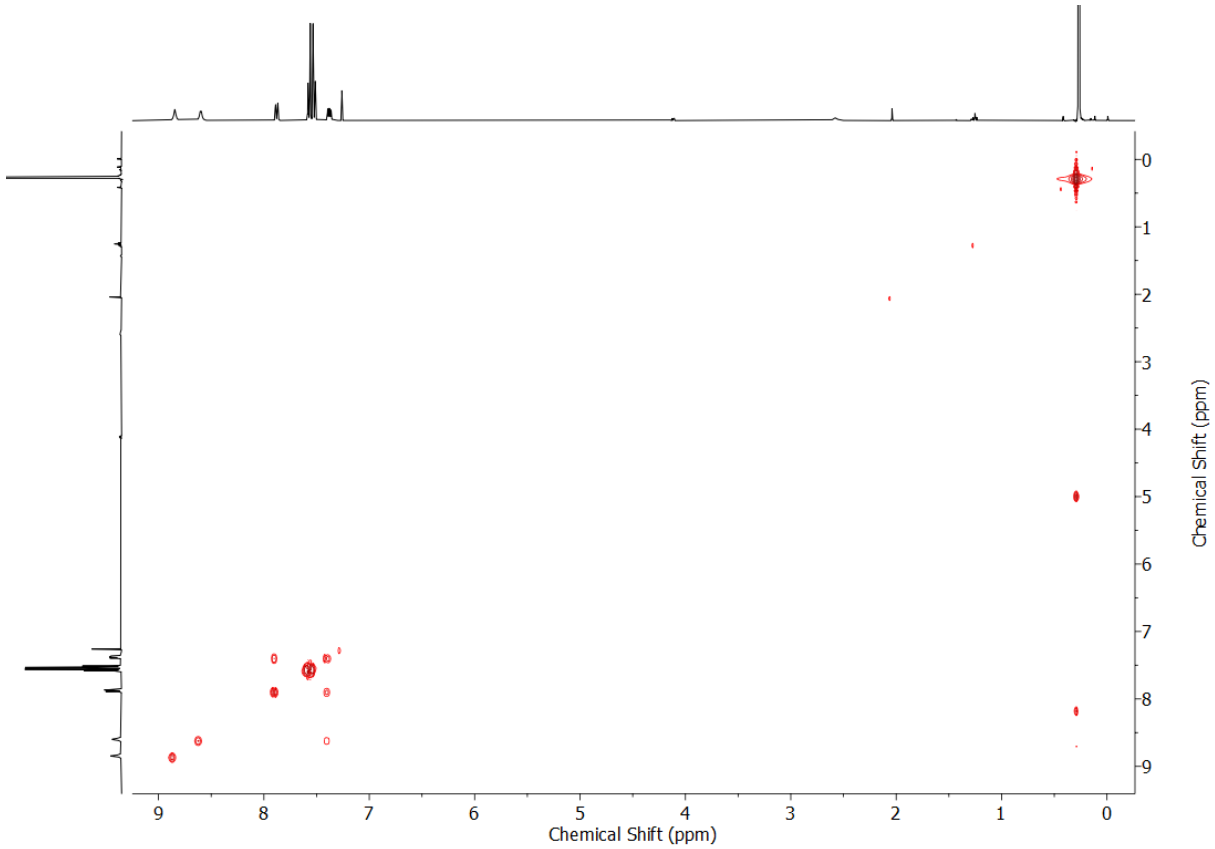

Figure S42 COSY NMR ($CDCl_3$) of **S6**.

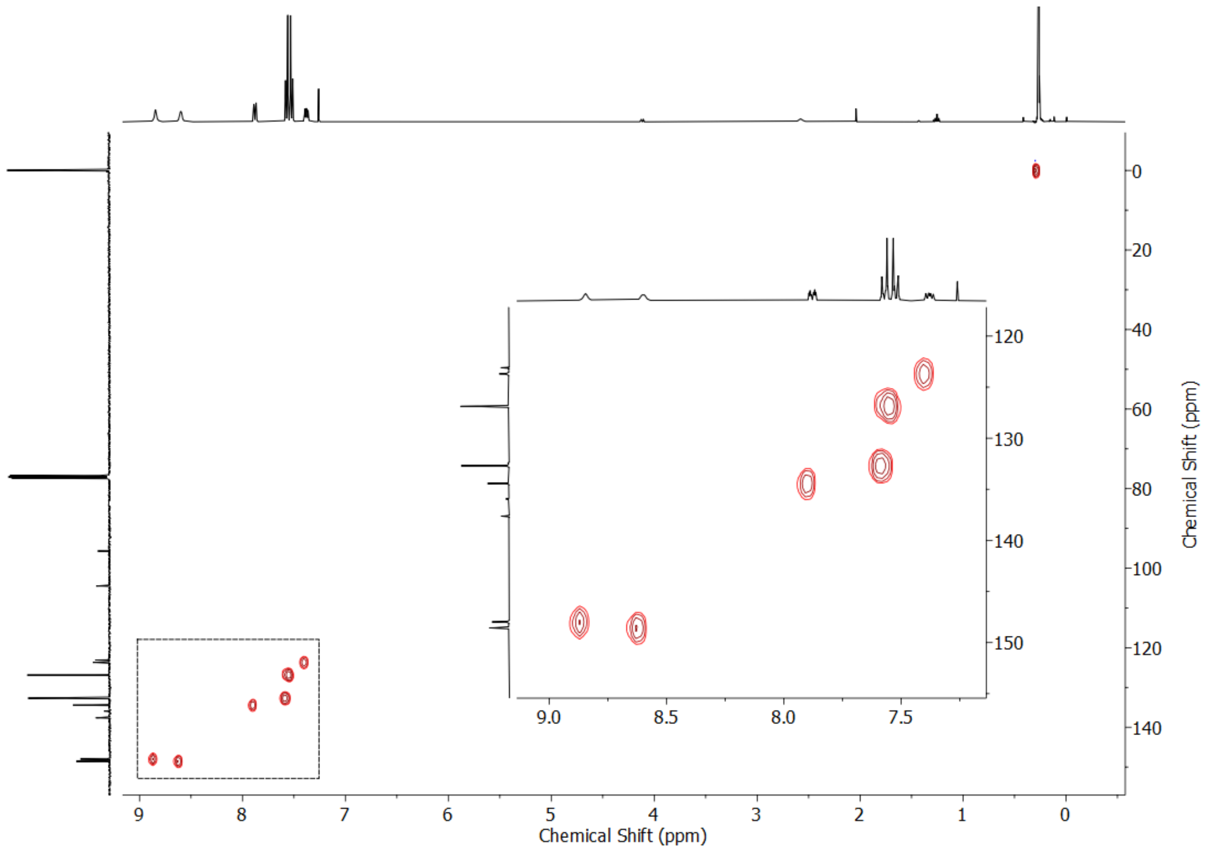

Figure S43 HSQC NMR ($CDCl_3$) of **S6**.

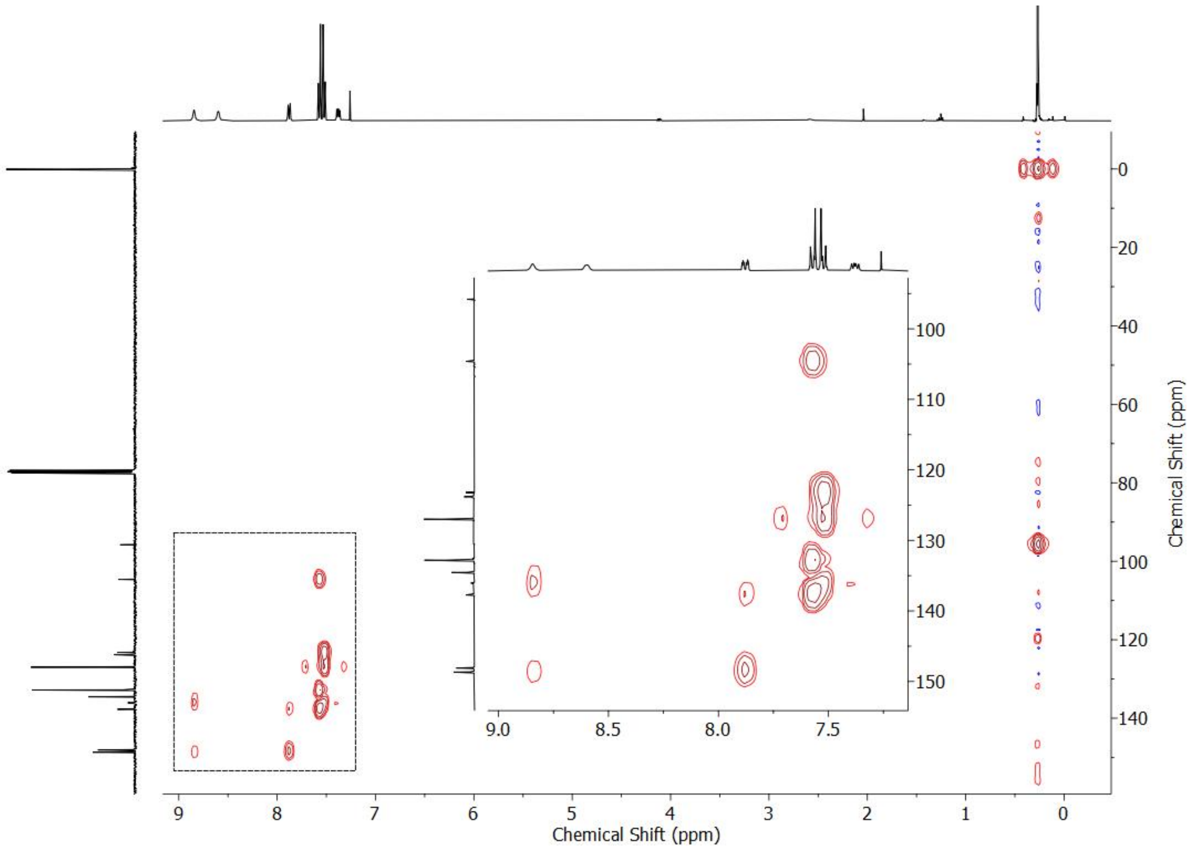

Figure S44 HMBC NMR ($CDCl_3$) of **S6**.

Synthesis of 4B1

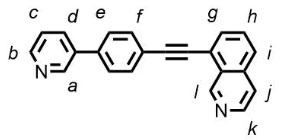

8-Iodoisoquinoline (0.148 g, 0.579 mmol, 1 eq.), **S6** (0.160 g, 0.636 mmol, 1.1 eq.), $Pd(PPh_3)_2Cl_2$ (0.0102 g, 0.0145 mmol, 2.5 mol%), CuI (0.0055 g, 0.029 mmol, 5 mol%) and DBU (0.52 mL, 3.47 mmol, 6 eq.) were stirred at rt in MeCN (5 mL) for 16 h. EDTA solution (20 mL) was added and the reaction mixture extracted with CH_2Cl_2 (3 × 20 mL). The combined organic phases were dried ($MgSO_4$) and the solvent removed *in vacuo*. Following column chromatography on silica (step gradient acetone/CH_2Cl_2 10:90 ⟶ 30:70 in 10% increments) the obtained residue was dissolved in CH_2Cl_2 (50 mL) and washed with H_2O (10 mL), sat. aq. $NaHCO_3$ (10 mL), brine (10 mL), dried ($MgSO_4$) and the solvent removed to give the product as a brown solid (0.023 g, 13%). ^{1}H NMR (400 MHz, $CDCl_3$) δ: 9.81 (s, 1H, H_l), 8.91 (d, J = 1.5 Hz, 1H, H_a), 8.64-8.61 (m, 2H, H_b, H_k), 7.94 (ddd, J = 7.9, 2.4, 1.6 Hz, 1H, H_d), 7.88 (dd, J = 7.1, 1.1 Hz, 1H, H_g), 7.85 (d, J = 8.3 Hz, 1H, H_h/H_i), 7.78 (d, J = 8.6 Hz, 2H, H_f), 7.75-7.71 (m, 2H, H_h/H_i, H_j), 7.66 (d, J = 8.5 Hz, 2H, H_e), 7.42 (ddd, J = 7.9, 4.8, 0.8 Hz, 1H, H_c). ^{13}C NMR (101 MHz, $CDCl_3$) δ: 150.8, 148.9, 148.2, 142.9, 138.3, 136.2, 136.0, 134.6, 132.7, 132.0, 130.5, 128.1, 127.4, 127.3, 123.9, 122.6, 122.0, 121.2, 95.9, 86.7. HR-ESIMS m/z = 307.1228 [M+H]$^+$ calc. 307.1235.

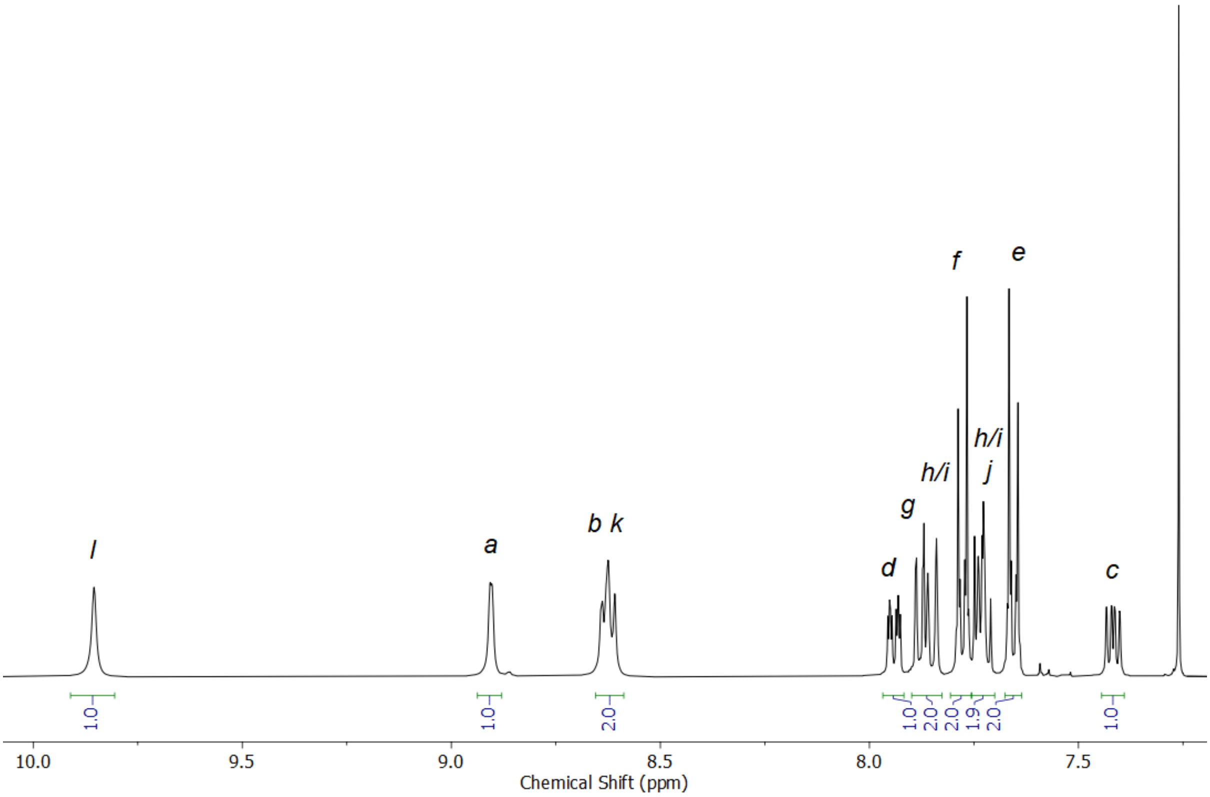

Figure S45 ^{1}H NMR ($CDCl_3$, 400 MHz) of **4B1**.

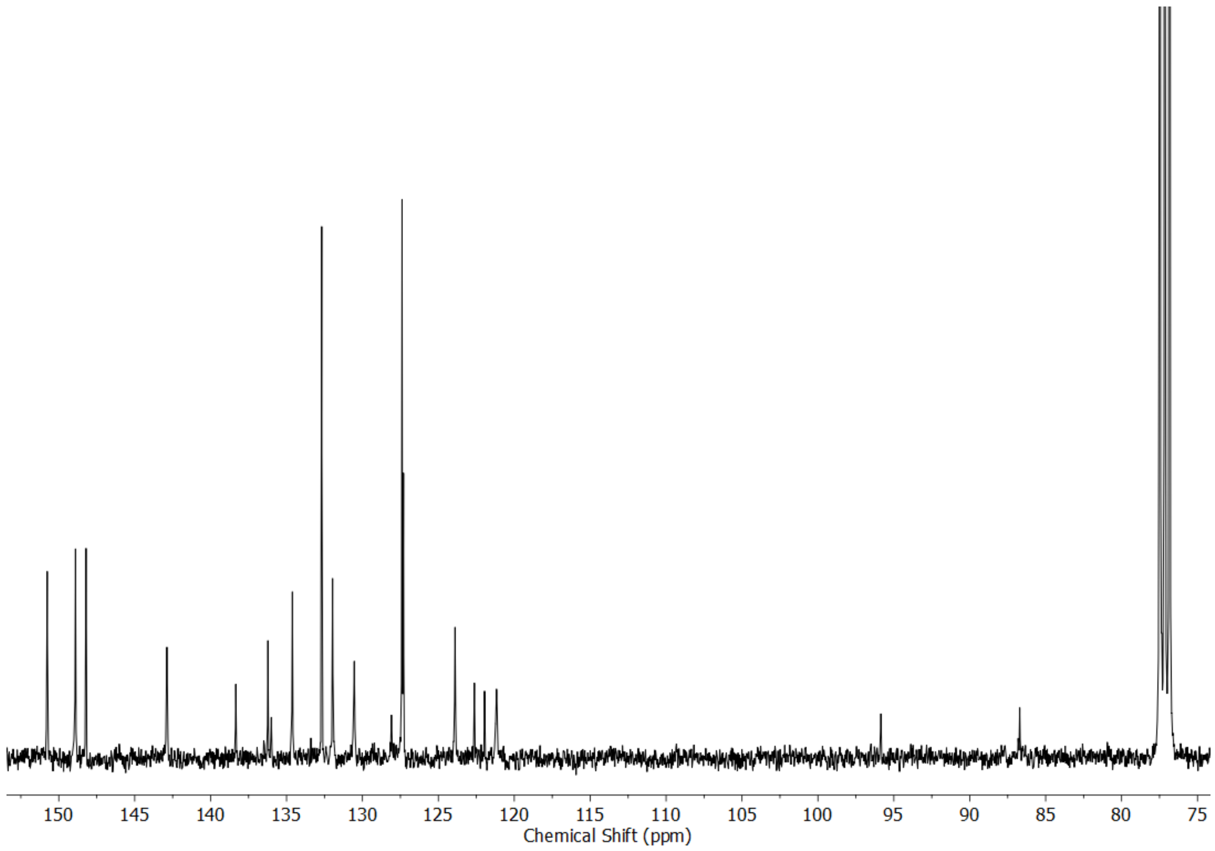

Figure S46 ^{13}C NMR ($CDCl_3$, 101 MHz) of **4B1**.

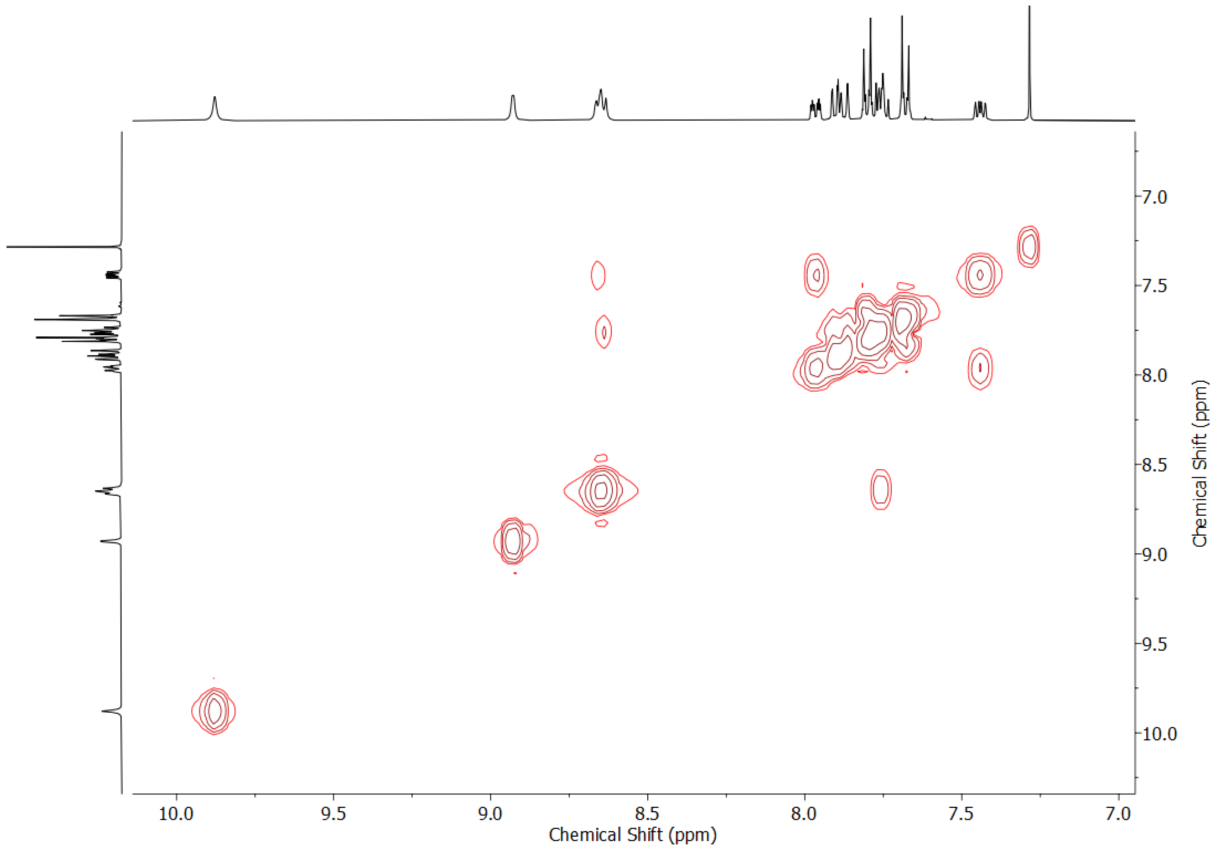

Figure S47 COSY NMR ($CDCl_3$) of **4B1**.

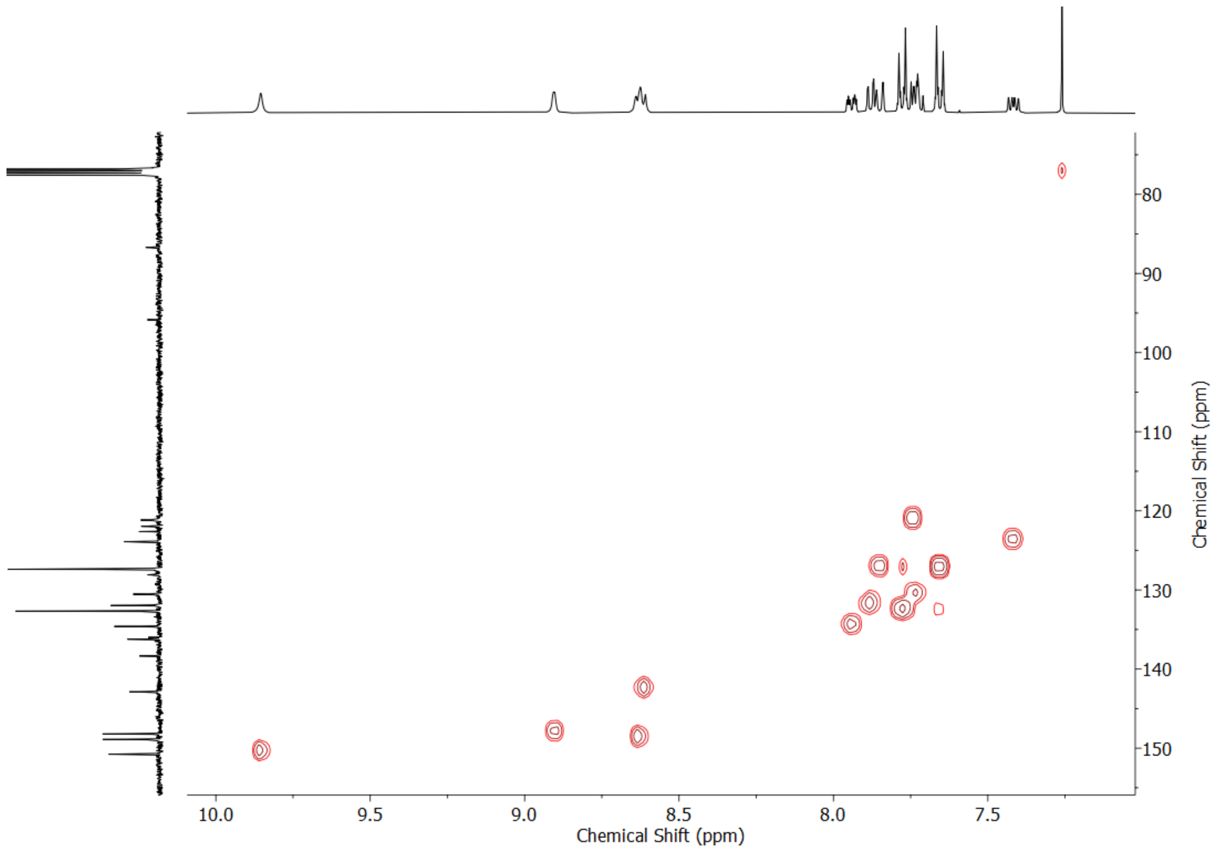

Figure S48 HSQC NMR ($CDCl_3$) of **4B1**.

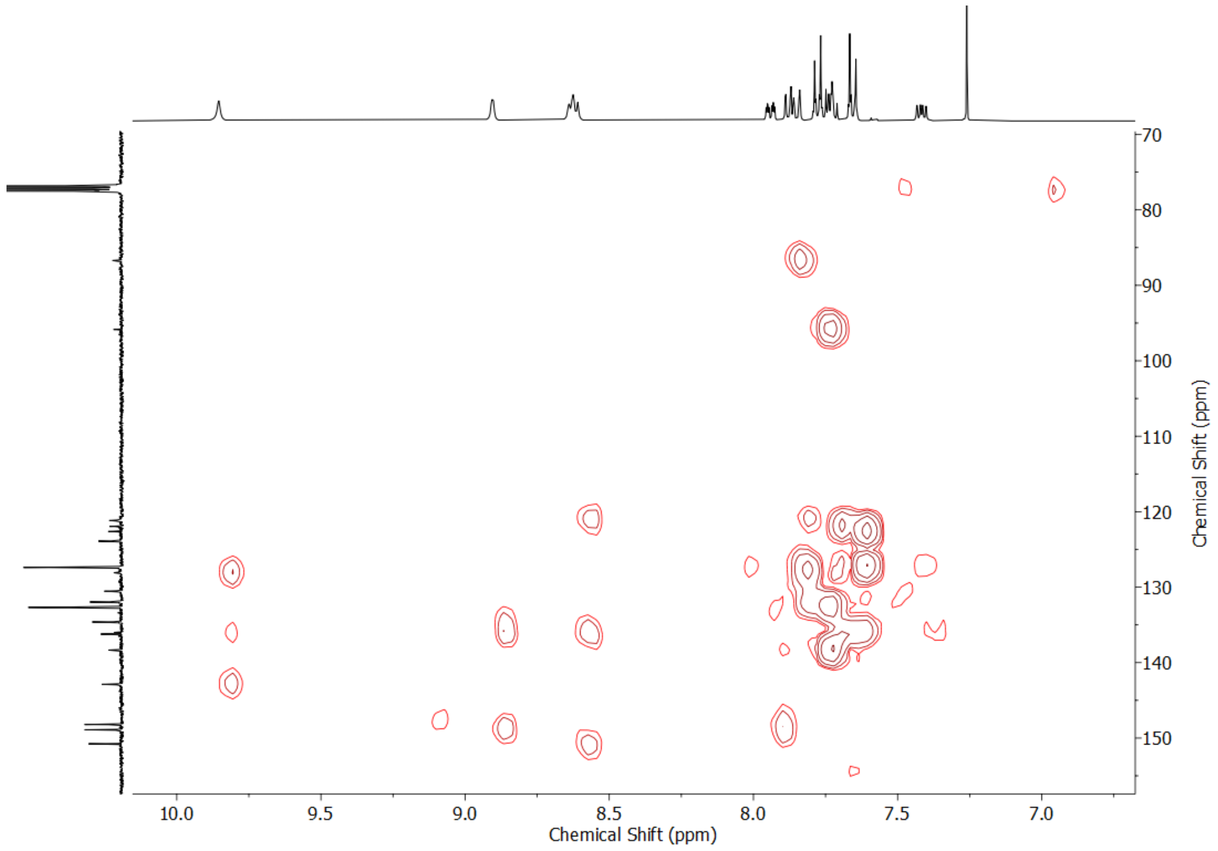

Figure S49 HMBC NMR ($CDCl_3$) of **4B1**.

Synthesis of S7

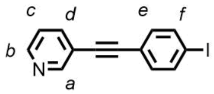

3-Ethynylpyridine (0.309 g, 3.0 mmol, 1.0 eq.), 1,4-diiodobenzene (1.98 g, 6.0 mmol, 2.0 eq.), $Pd(PPh_3)_2Cl_2$ (0.053 g, 0.075 mmol, 2.5 mol%) and CuI (0.029 g, 0.15 mmol, 5 mol%) were stirred at rt in $^{i}Pr_2NH$ (30 mL) for 18 h. After the solvent was removed *in vacuo*, EDTA solution (25 mL) and CH_2Cl_2 (50 mL) were added. The aqueous phase was further extracted with CH_2Cl_2 (2 × 10 mL); the combined organic extracts were dried ($MgSO_4$) and the solvent removed *in vacuo*. After purification by column chromatography on silica (step gradient EtOAc/petrol 0:100 ⟶ 20:80 in 10% increments) the product was obtained as a yellow solid (0.560 g, 61%). ^{1}H NMR (500 MHz, $CDCl_3$) δ: 8.76 (dd, *J* = 2.2, 0.9 Hz, 1H, H_a), 8.56 (dd, *J* = 4.9, 1.7 Hz, 1H, H_b), 7.80 (app. dt, *J* = 7.9, 1.9 Hz, 1H, H_d), 7.72 (d, *J* = 8.4 Hz, 2H, H_e), 7.30-7.25 (m, 3H, H_c, H_f). ^{13}C NMR (126 MHz, $CDCl_3$) δ: 152.4, 149.0, 138.6, 137.8, 133.3, 123.2, 122.2, 120.3, 95.0, 91.8, 87.5. HR-ESIMS m/z = 305.9775 $[M+H]^+$ calc. 305.9774.

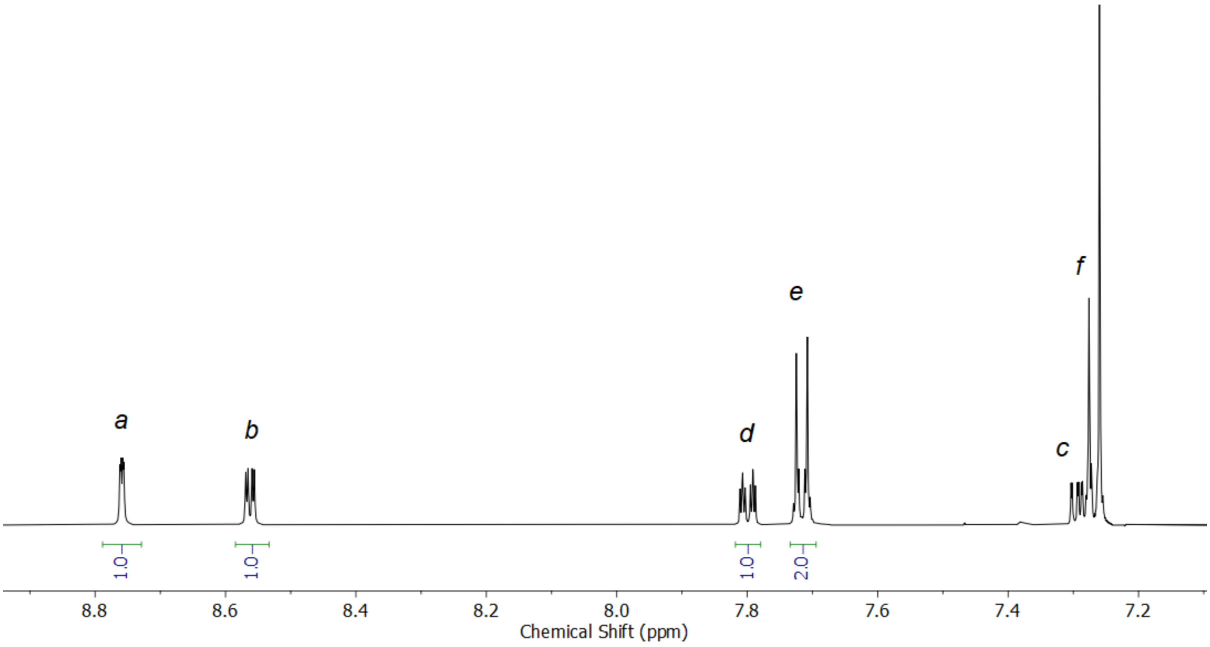

Figure S50 ^{1}H NMR ($CDCl_3$, 500 MHz) of **S7**.

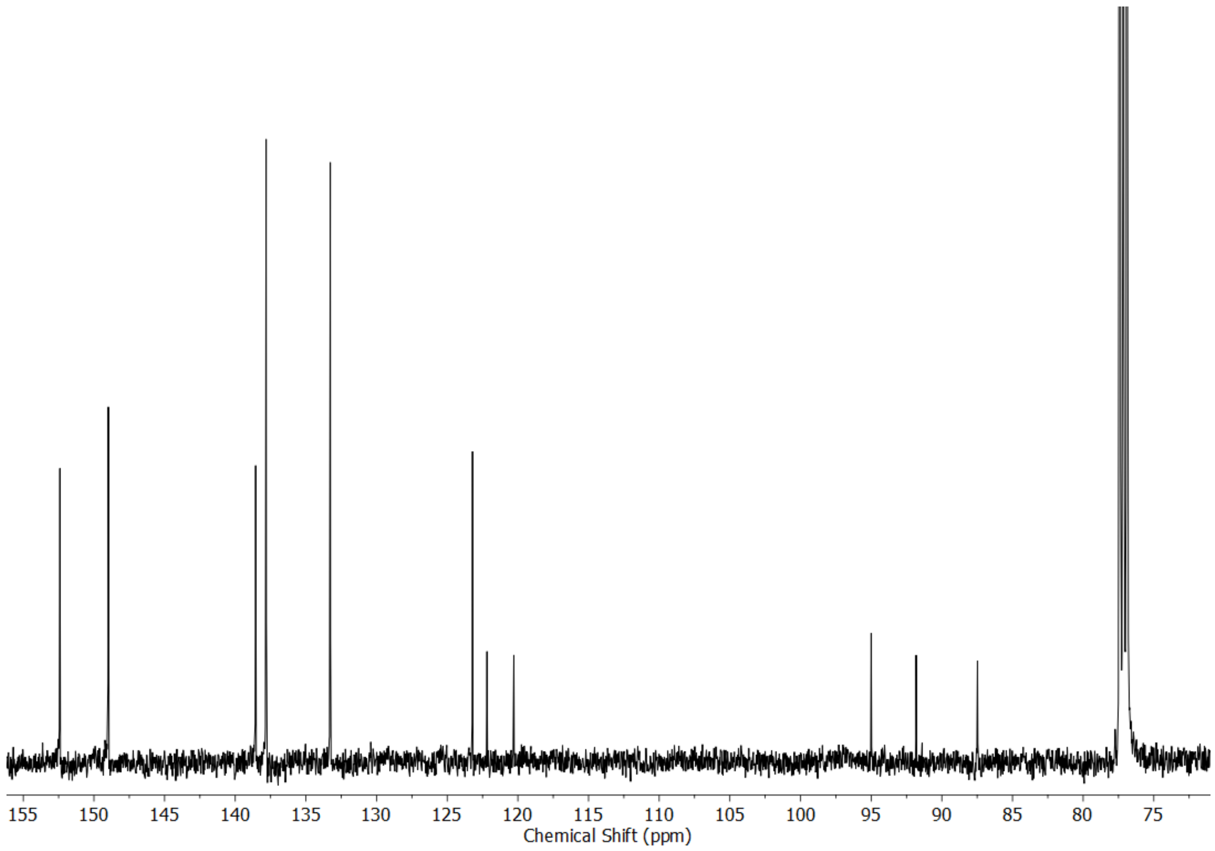

Figure S51 ^{13}C NMR ($CDCl_3$, 126 MHz) of **S7**.

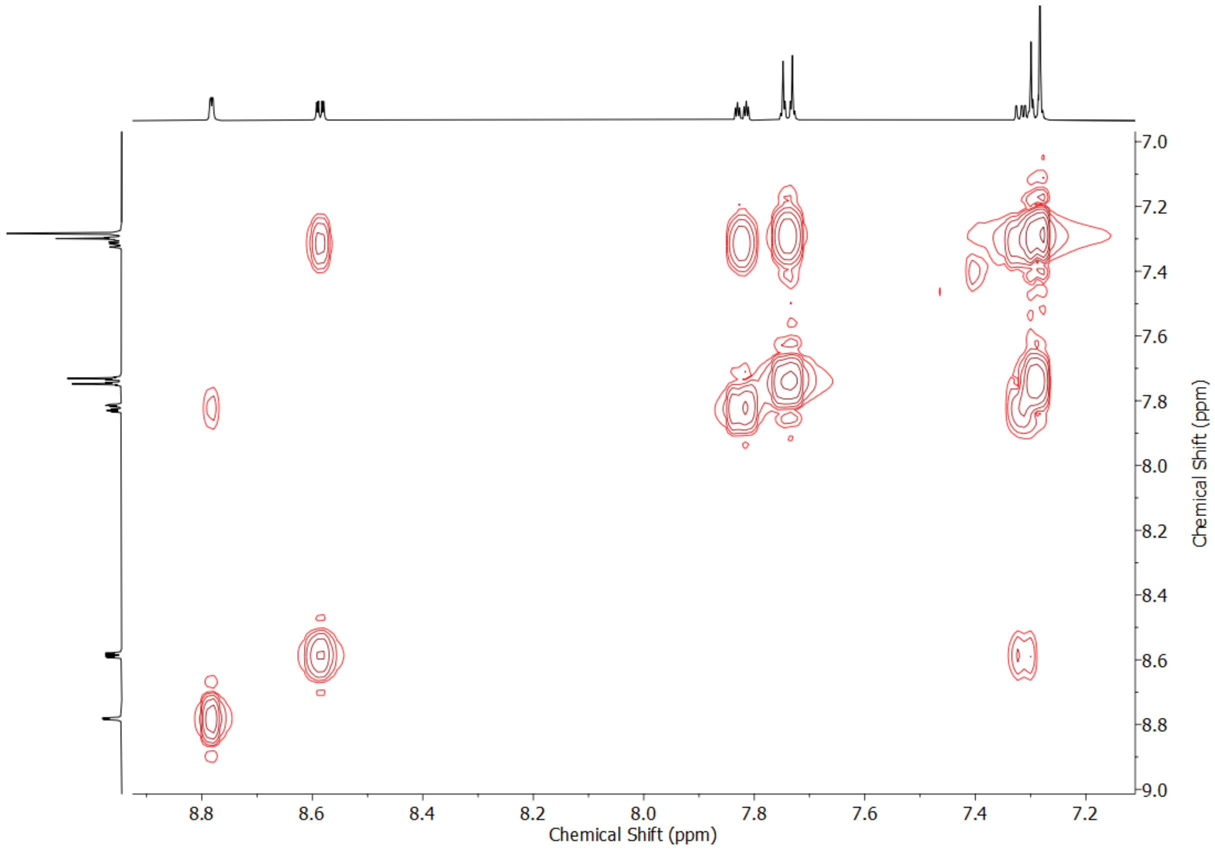

Figure S52 COSY NMR ($CDCl_3$) of **S7**.

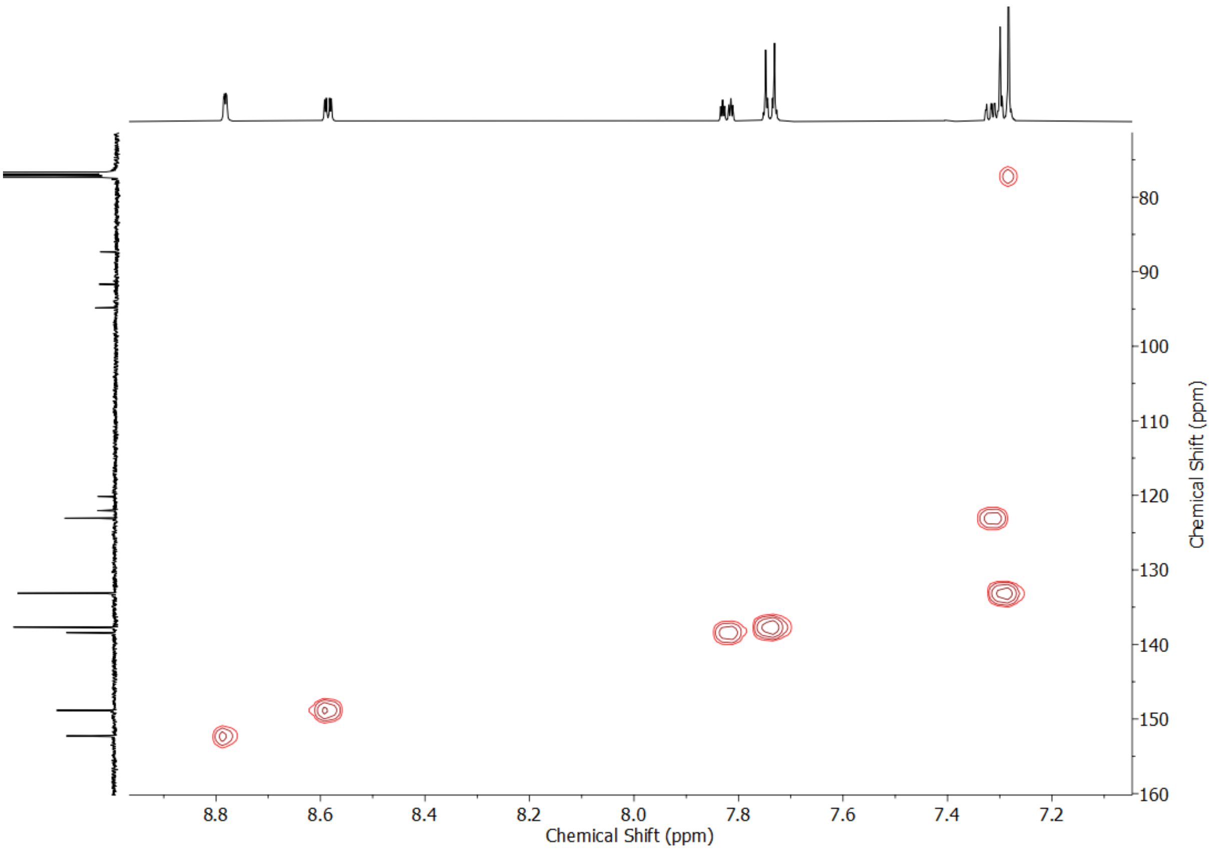

Figure S53 HSQC NMR ($CDCl_3$) of **S7**.

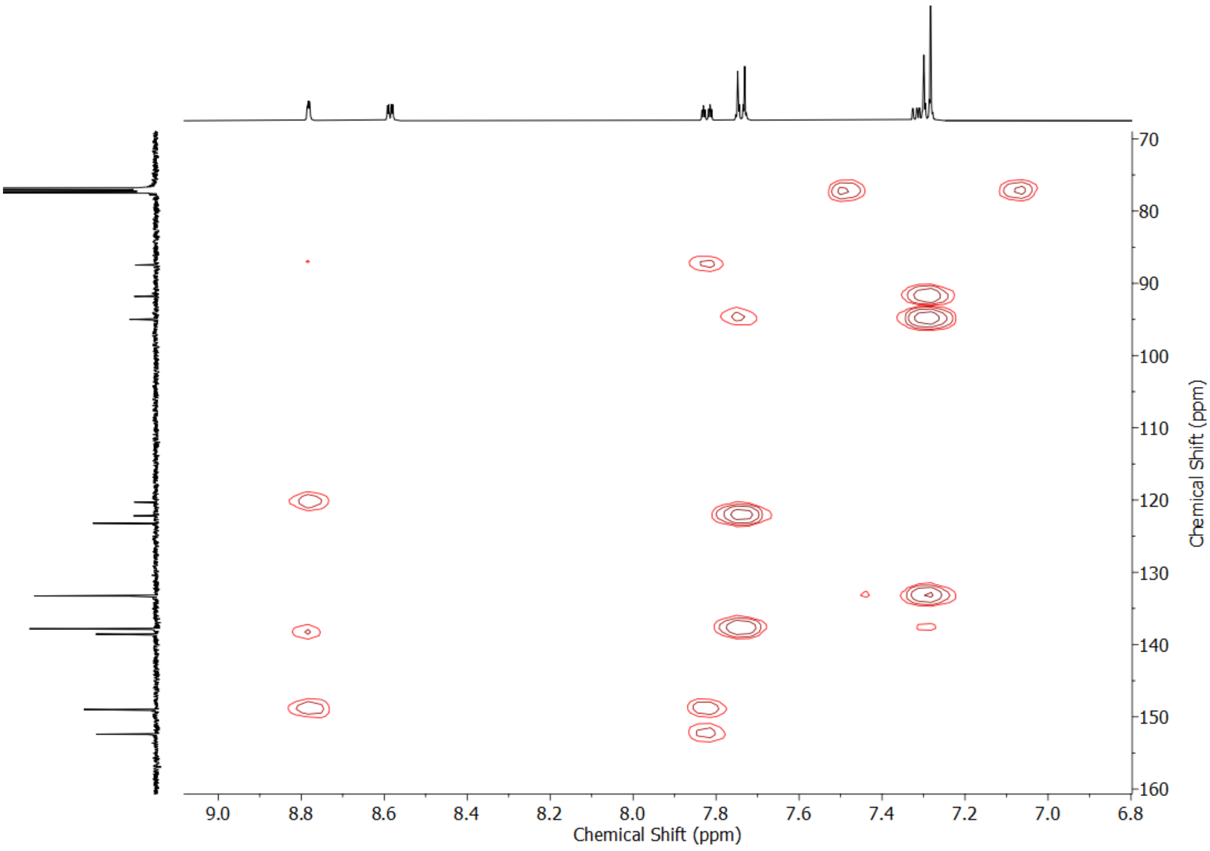

Figure S54 HMBC NMR ($CDCl_3$) of **S7**.

Synthesis of 4B3

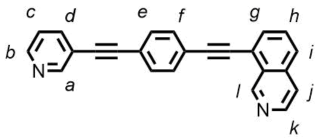

S7 (122 mg, 0.40 mmol, 1.0 eq.), 8-trimethylsilylethynylisoquinoline (99 mg, 0.44 mmol, 1.1 eq.), $Pd(PPh_3)_2Cl_2$ (7.0 mg, 0.010 mmol, 2.5 mol%), CuI (3.8 mg, 0.020 mmol, 5 mol%) and DBU (0.36 mL, 2.40 mmol, 6.0 eq.) were stirred at rt in MeCN (4.0 mL) for 17 h. EDTA solution (20 mL) was added and the mixture extracted with CH_2Cl_2 (3 × 20 mL). The combined organic extracts were dried ($MgSO_4$) and the solvent removed *in vacuo*. After purification by column chromatography on silica (1st column step gradient acetone/CH_2Cl_2 0:100 ⟶ 20:80 in 5% increments; 2nd column 1:9 EtOAc/Et_2O) the product was obtained as a light yellow solid (55.5 mg, 42%). ^{1}H NMR (400 MHz, $CDCl_3$) δ: 9.82 (s, 1H, H_l), 8.79 (dd, J = 2.2, 0.9 Hz, 1H, H_a), 8.62 (d, J = 5.7 Hz, 1H, H_k), 8.57 (dd, J = 4.9, 1.7 Hz, 1H, H_b), 7.84-7.81 (m, 3H, H_d, 2 of H_g/H_h/H_i), 7.70-7.65 (m, 4H, H_e/H_f, H_j, 1 of H_g/H_h/H_i), 7.59 (d, J = 8.3 Hz, 2H, H_e/H_f), 7.31 (ddd, J = 7.9, 4.9, 0.9 Hz, 1H, H_c). ^{13}C NMR (101 MHz, $CDCl_3$) δ: 152.5, 151.3, 149.0, 144.0, 138.6, 135.9, 131.9 (× 2), 131.7, 129.9, 128.1, 127.4, 123.2, 123.2, 123.1, 121.5, 120.7, 120.3, 95.4, 92.3, 88.2, 87.9, 77.5, 77.2, 76.8. HR-ESIMS m/z = 331.1222 [M+H]$^+$ calc. 331.1230.

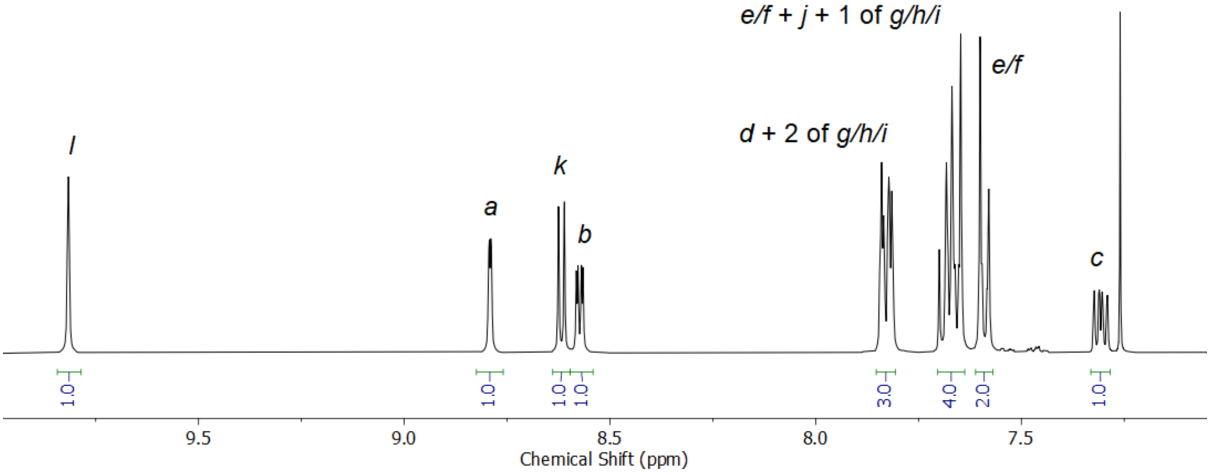

Figure S55 ^{1}H NMR ($CDCl_3$, 400 MHz) of **4B3**.

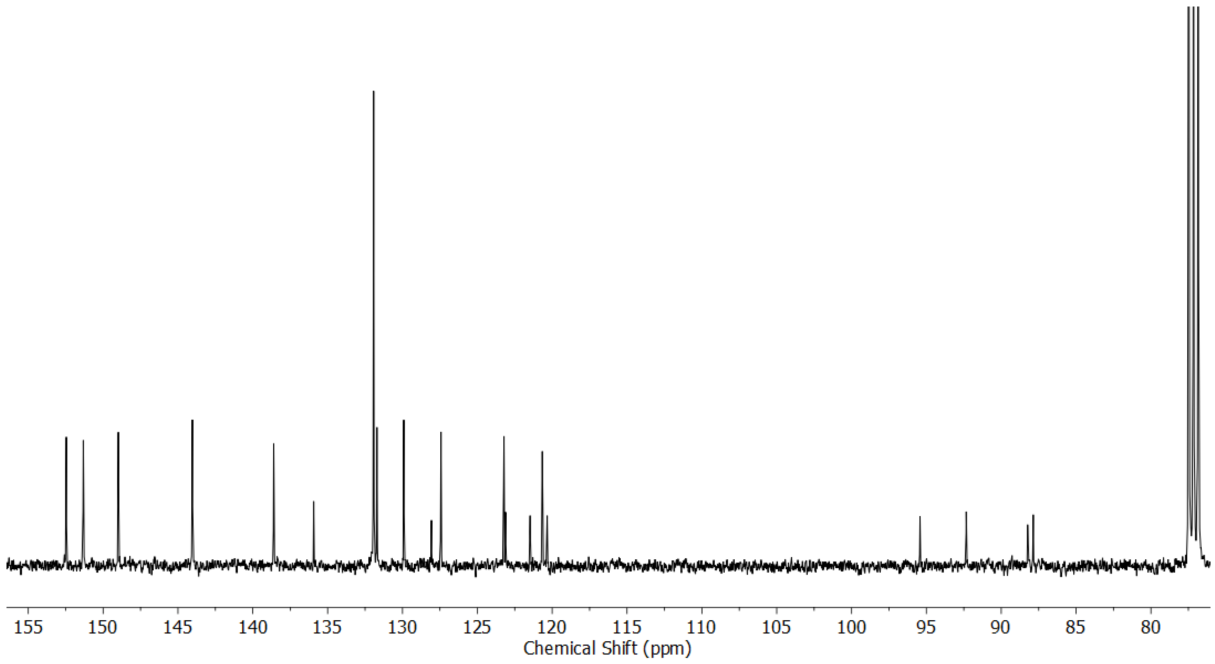

Figure S56 ^{13}C NMR ($CDCl_3$, 101 MHz) of **4B3**.

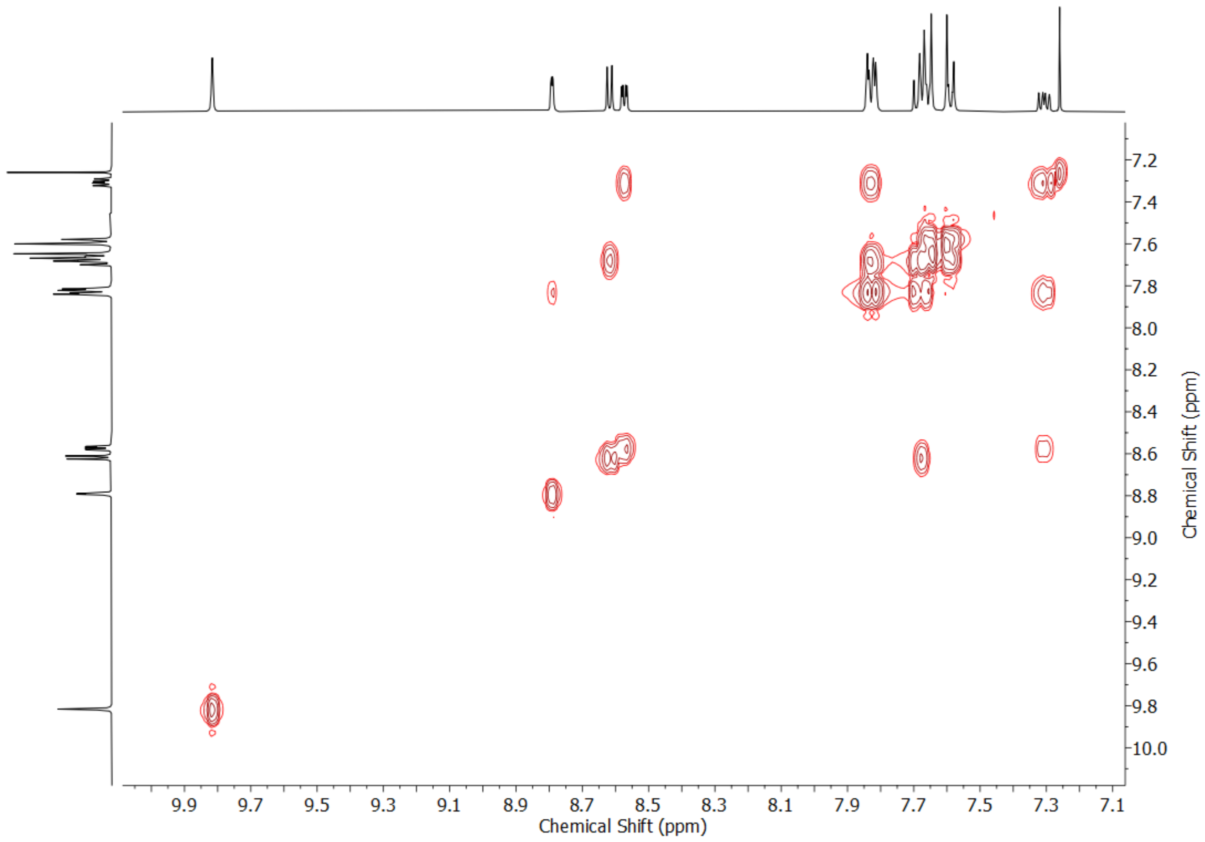

Figure S57 COSY NMR ($CDCl_3$) of **4B3**.

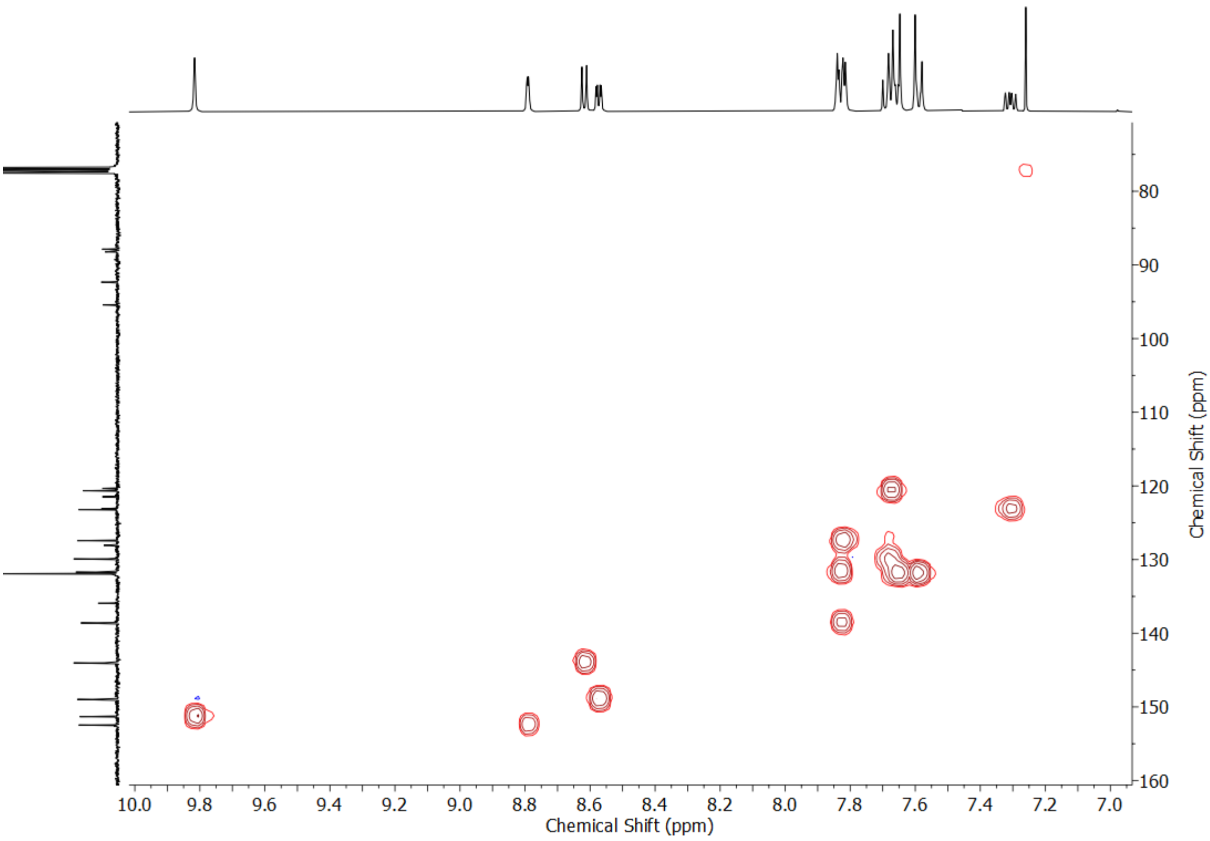

Figure S58 HSQC NMR ($CDCl_3$) of **4B3**.

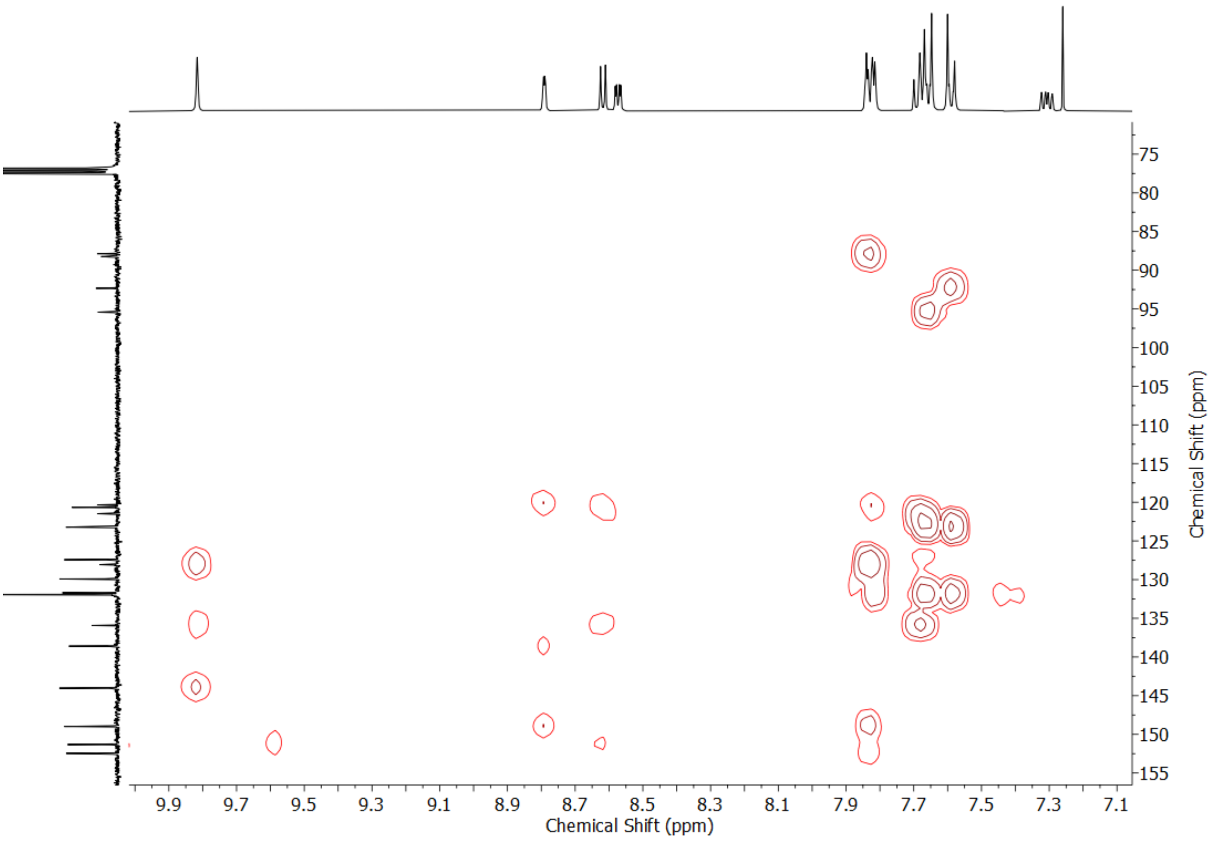

Figure S59 HMBC NMR ($CDCl_3$) of **4B3**.

Synthesis of S8

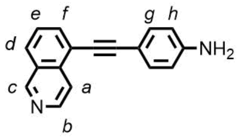

5-Bromoisoquinoline (0.626 g, 3.0 mmol, 1.0 eq.), 4-ethynylaniline (0.387 g, 3.3 mmol, 1.1 eq.), $[Pd(PPh_3)_2Cl_2]$ (0.053 g, 0.075 mmol, 2.5 mol%) and CuI (0.029 g, 0.15 mmol, 5 mol%) were stirred at 80 °C in 2:1 dioxane/$^{i}Pr_2NH$ (18 mL) in a sealed vial for 18 h. The reaction mixture was diluted with CH_2Cl_2 (50 mL), washed with EDTA solution (25 mL), dried ($MgSO_4$) and the solvent removed *in vacuo*. After purification by column chromatography (step gradient acetone/CH_2Cl_2 0:100 to 15:85 in 5% increments) the product was obtained as a yellow solid (0.618 g, 84%). ^1H NMR (400 MHz, $CDCl_3$) δ: 9.36 (br. s, 1H, H_c), 8.68 (br. s, 1H, H_b), 8.26 (br. s, 1H, H_a), 7.95-7.90 (m, 2H, H_d, H_f), 7.60 (dd, J = 8.2, 7.2 Hz, 1H, H_e), 7.45 (d, J = 8.6 Hz, 2H, H_g), 6.69 (d, J = 8.6 Hz, 2H, H_h). ^{13}C NMR (101 MHz, $CDCl_3$) δ: 152.3, 147.4, 143.0, 136.3, 134.0, 133.3, 127.5, 127.3, 121.4, 119.9, 114.9, 112.1, 96.9, 84.0. HR-ESIMS m/z = 245.1080 $[M+H]^+$ calc. 245.1079.

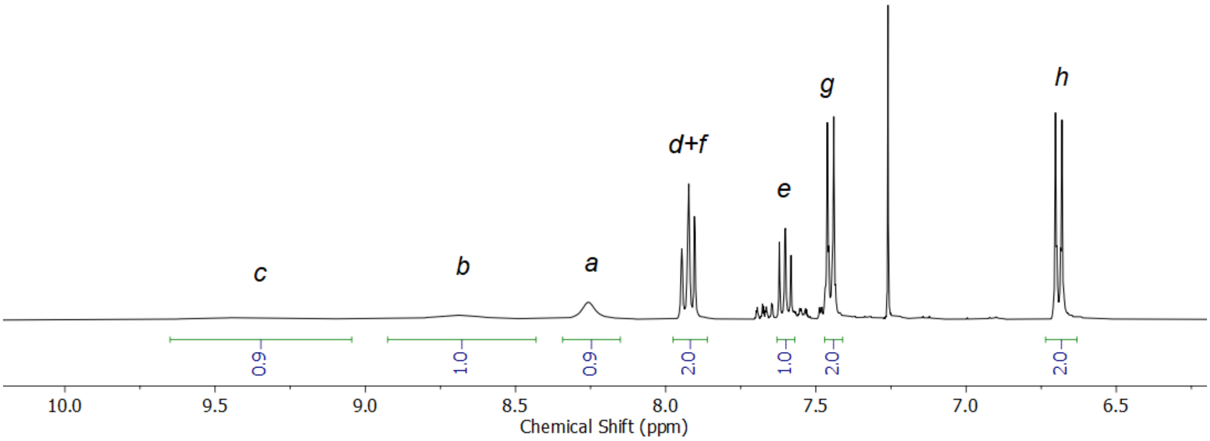

Figure S60 ^1H NMR ($CDCl_3$, 400 MHz) of **S8**.

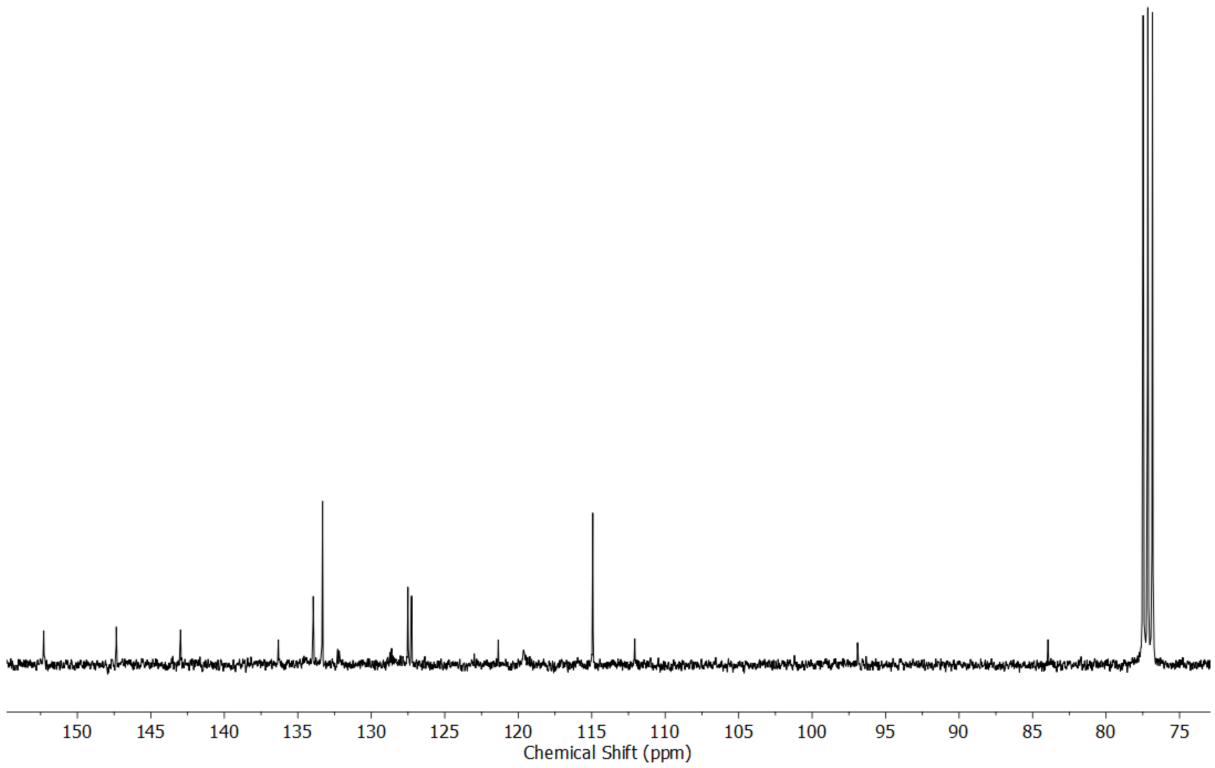

Figure S61 ^{13}C NMR ($CDCl_3$, 101 MHz) of **S8**.

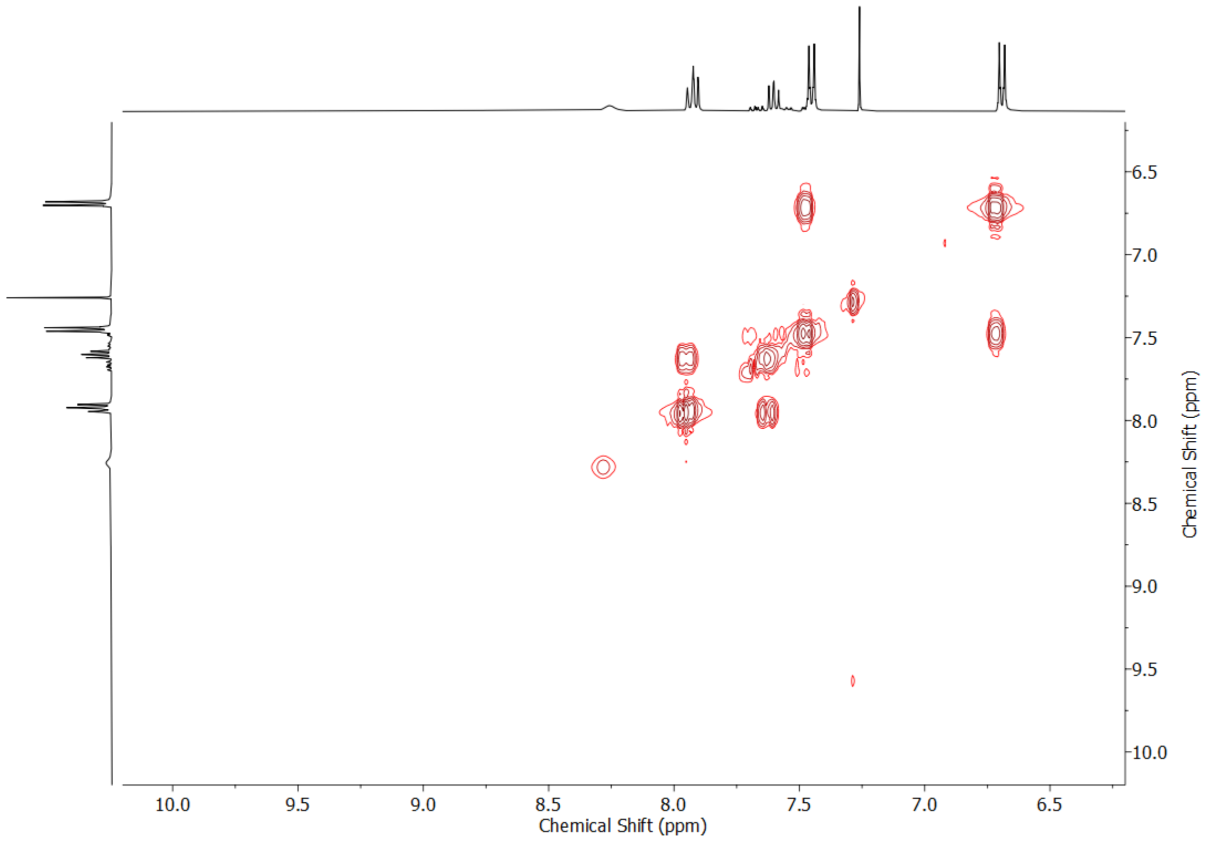

Figure S62 COSY NMR ($CDCl_3$) of **S8**.

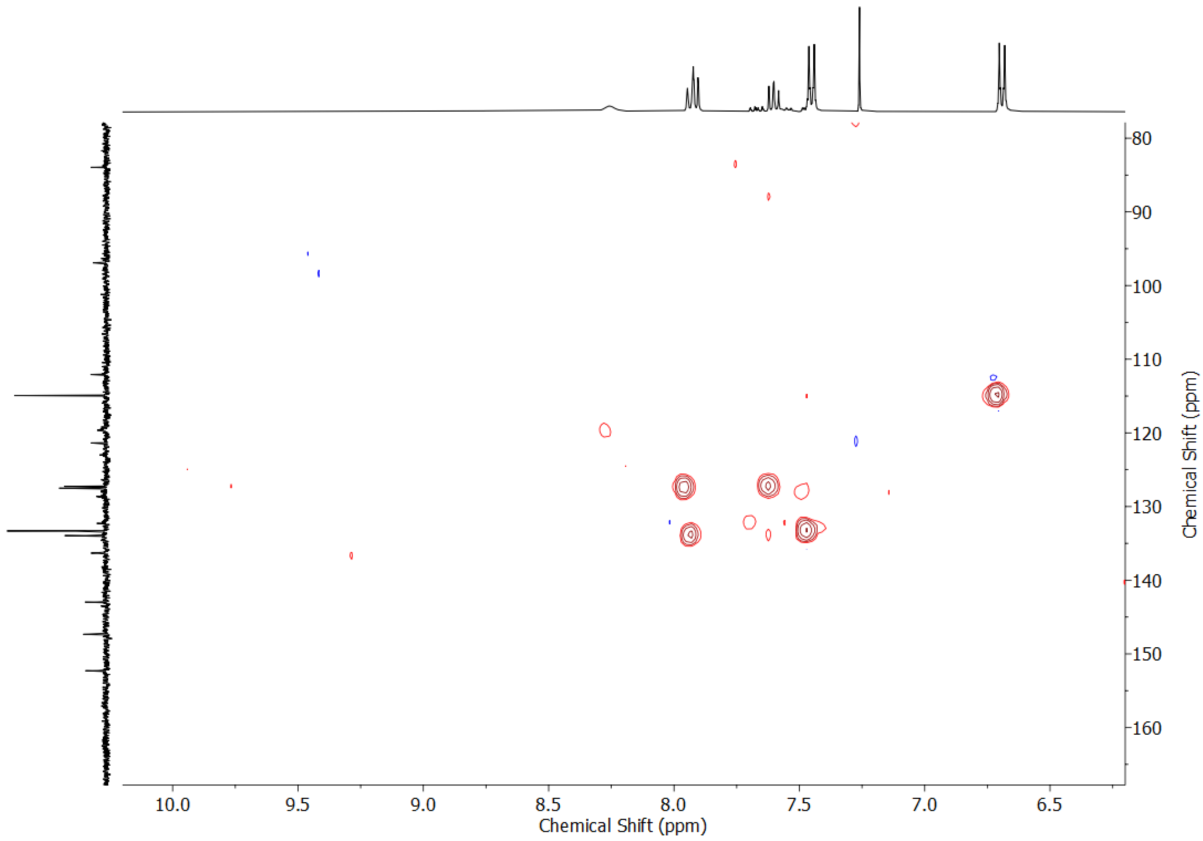

Figure S63 HSQC NMR ($CDCl_3$) of **S8**.

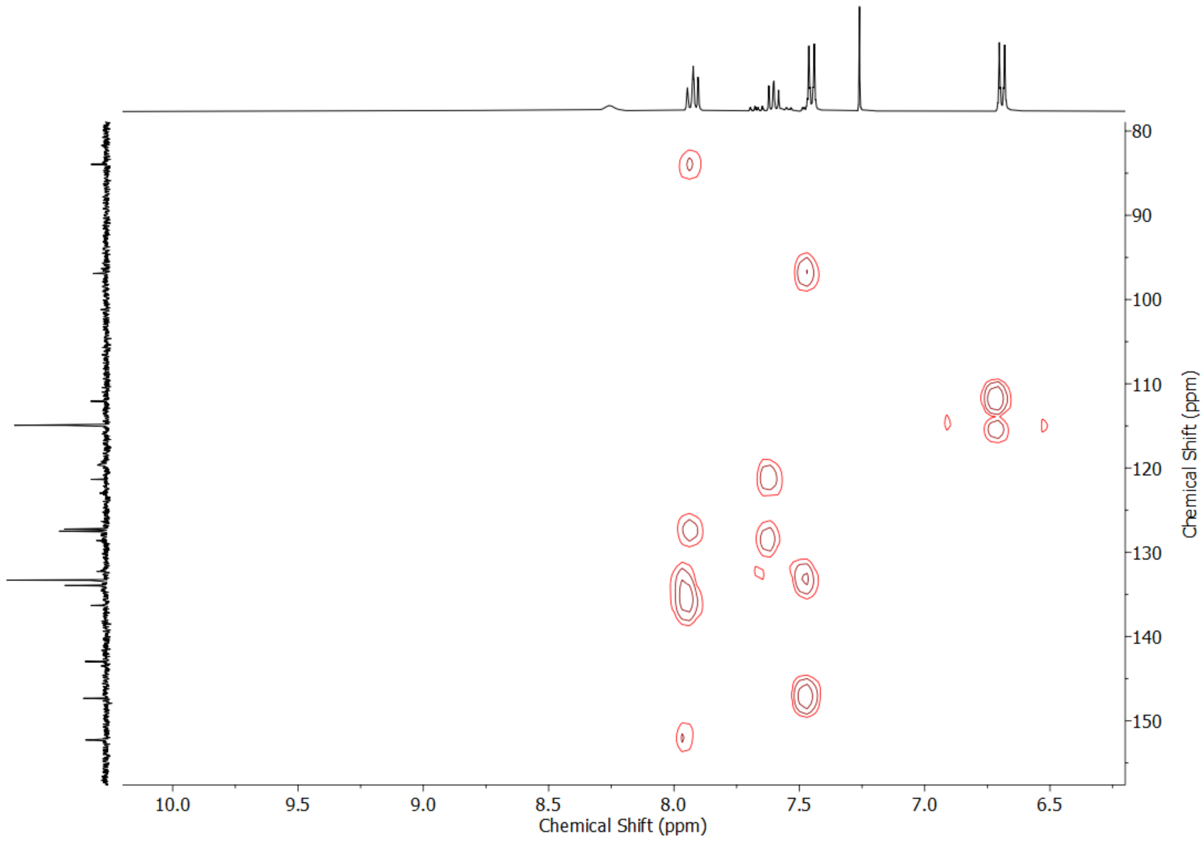

Figure S64 HMBC NMR ($CDCl_3$) of **S8**.

Synthesis of S9

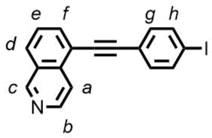

To **S8** (0.244 g, 1.0 mmol, 1.0 eq.) in 1 M $HCl_{(aq)}$ (5 mL) at 0 °C was added $NaNO_2$ (0.104 g, 1.5 mmol, 1.5 eq.) as a solid. After stirring for 30 minutes a solution of KI (0.498 g, 3.0 mmol, 3.0 eq.) in H_2O (2 mL) was added dropwise. The reaction was then allowed to warm to rt and stirred for 3 h before sat. aq. $NaHCO_3$ (50 mL) was added carefully followed by 1 M $Na_2S_2O_{3(aq)}$ (10 mL). The reaction mixture was extracted with CH_2Cl_2 (3 × 20 mL) and the combined organic extracts dried ($MgSO_4$) and the solvent removed *in vacuo*. Purification by column chromatography (1:9 EtOAc/CH_2Cl_2) gave the product as a light yellow solid (0.263 g, 74%). ^{1}H NMR (400 MHz, $CDCl_3$) δ: 9.29 (s, 1H, H_c), 8.64 (d, *J* = 5.9 Hz, 1H, H_b), 8.15 (d, *J* = 5.9 Hz, 1H, H_a), 7.99 (dt, *J* = 8.2, 1.1 Hz, 1H, H_d), 7.94 (dd, *J* = 7.2, 1.2 Hz, 1H, H_f), 7.75 (d, *J* = 8.4 Hz, 2H, H_g/H_h), 7.61 (dd, *J* = 8.2, 7.2 Hz, 1H, H_e), 7.36 (d, *J* = 8.4 Hz, 2H, H_g/H_h). ^{13}C NMR (101 MHz, $CDCl_3$) δ: 152.6, 143.6, 137.8, 136.1, 134.4, 133.2, 128.4, 127.0, 122.3, 120.1, 119.0, 94.9, 94.6, 87.2. HR-ESIMS m/z = 355.9939 $[M+H]^+$ calc. 355.9936.

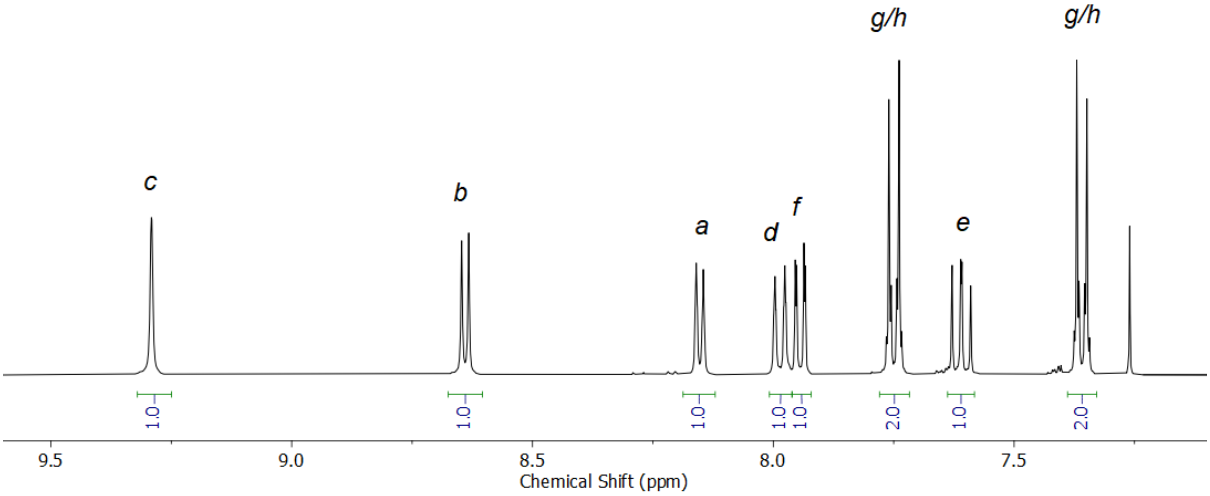

Figure S65 ^{1}H NMR ($CDCl_3$, 400 MHz) of **S9**.

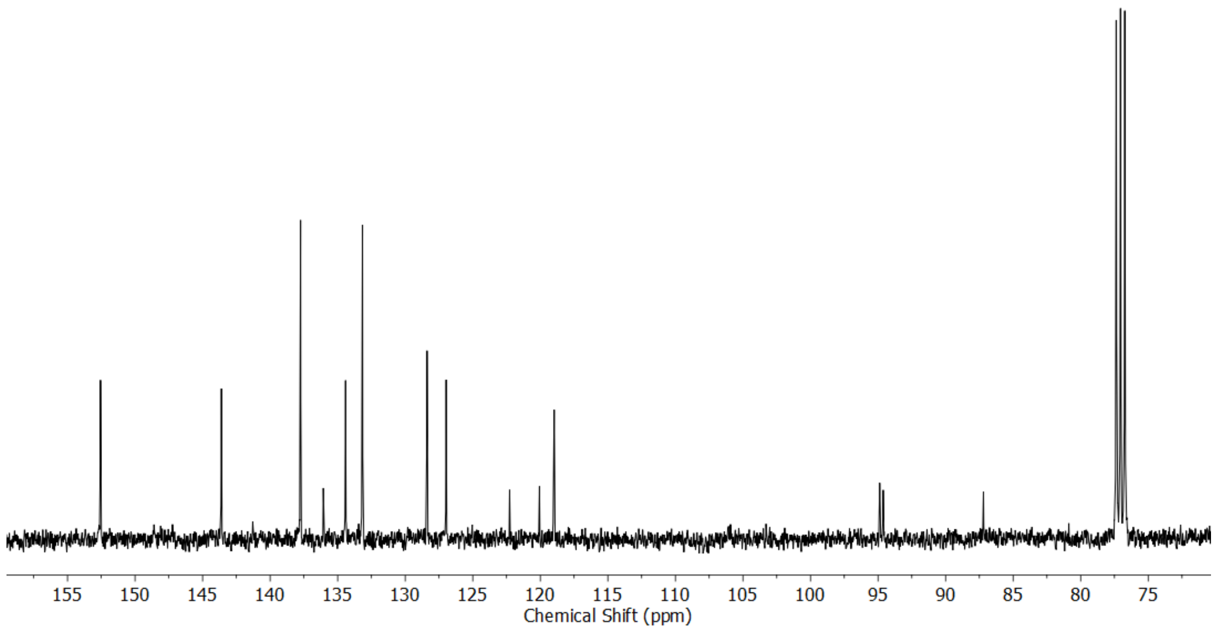

Figure S66 ^{13}C NMR ($CDCl_3$, 101 MHz) of **S9**.

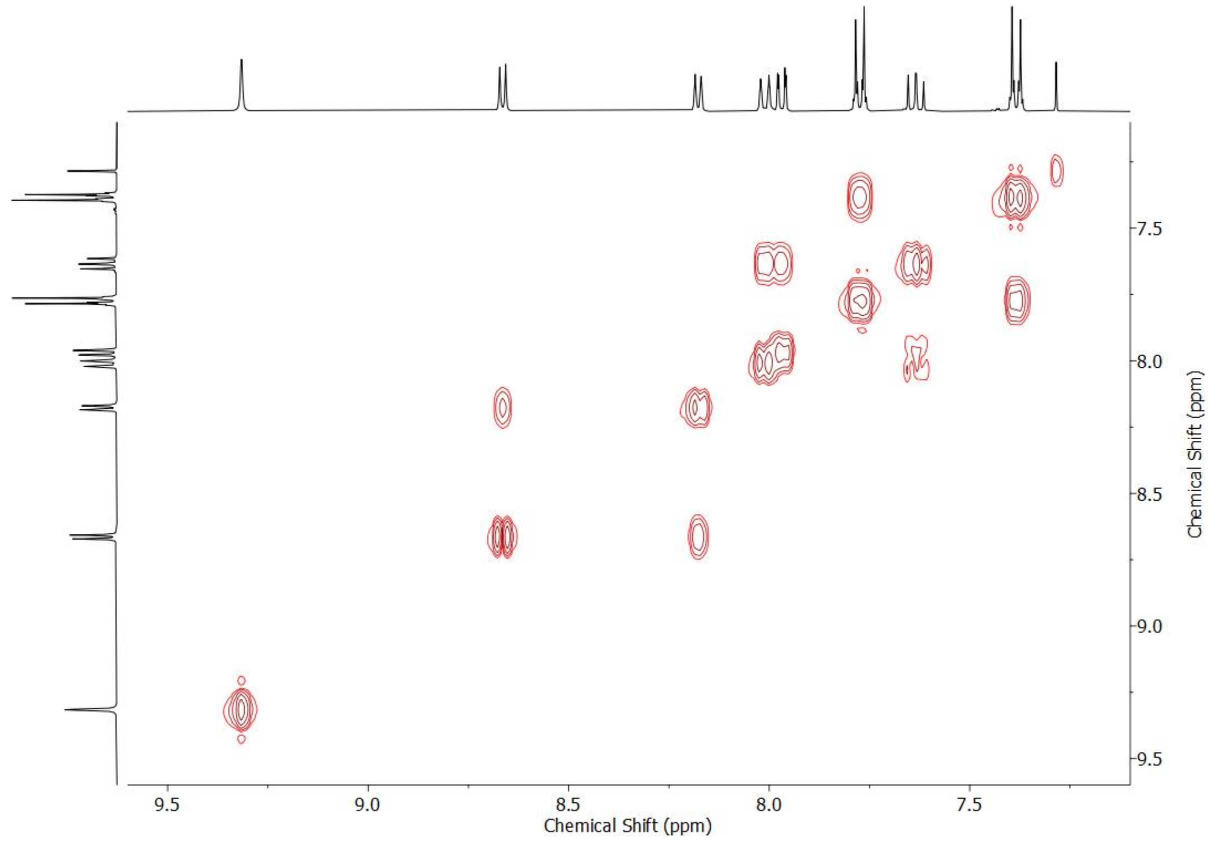

Figure S67 COSY NMR ($CDCl_3$) of **S9**.

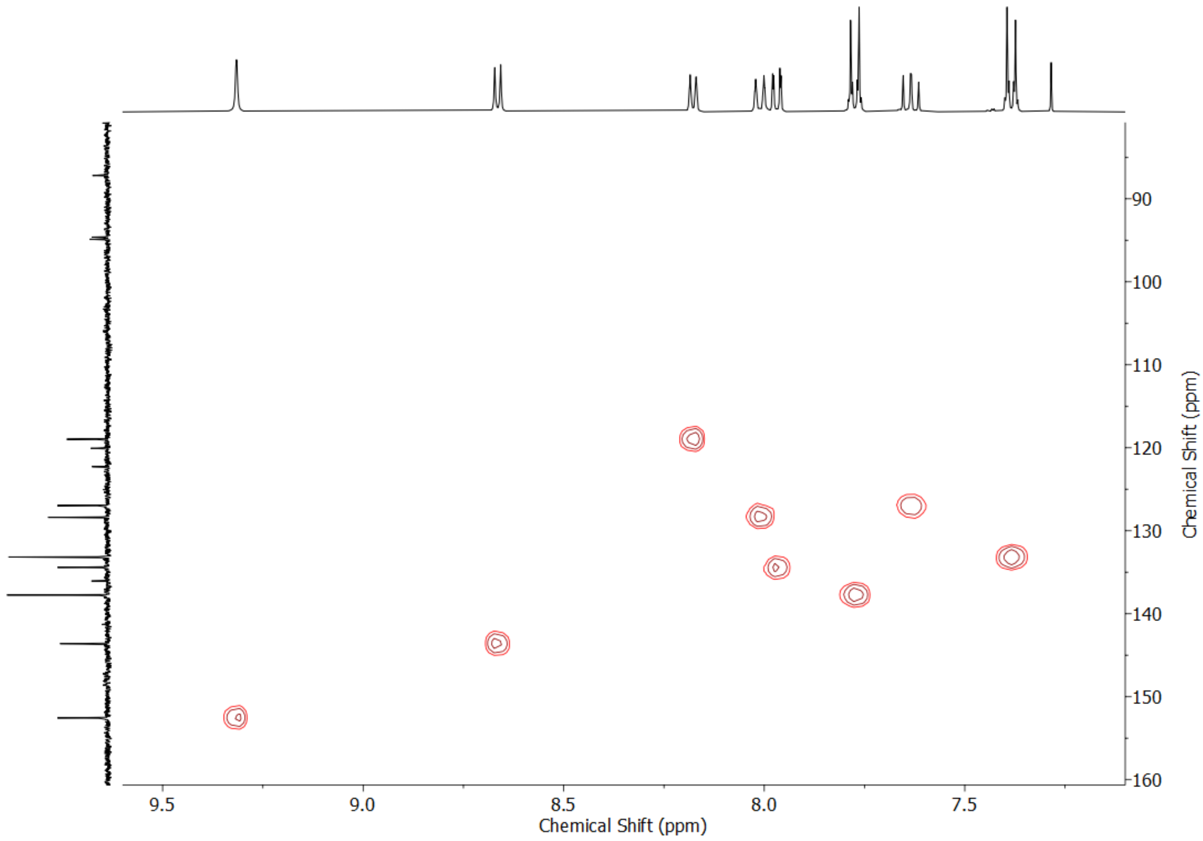

Figure S68 HSQC NMR ($CDCl_3$) of **S9**.

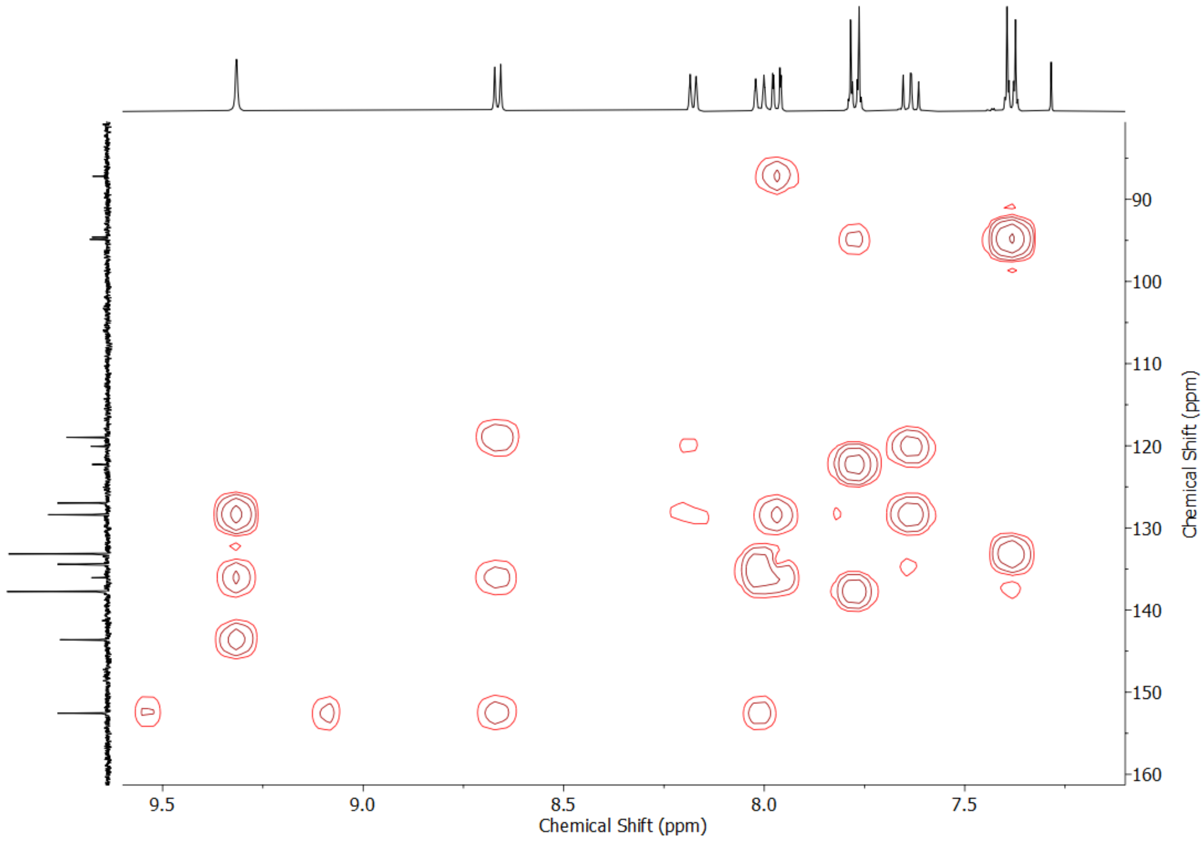

Figure S69 HMBC NMR ($CDCl_3$) of **S9**.

Synthesis of 5B4

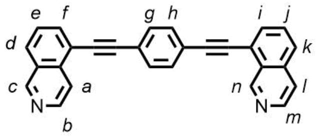

Trimethylsilylacetylene (0.07 mL, 0.5 mmol, 1.2 eq.) was added via syringe to **S9** (0.142 g, 0.4 mmol, 1 eq.), $Pd(PPh_3)_2Cl_2$ (0.014 g, 0.02 mmol, 5 mol%), CuI (0.008 g, 0.04 mmol, 10 mol%) and $^{i}Pr_2NH$ (0.34 mL, 2.4 mmol, 6 eq.) in CH_3CN (3.5 mL) and the reaction mixture stirred at rt for 20 h. DBU (0.36 mL, 2.4 mmol, 6 eq.) was subsequently added and, after stirring at rt for 2 h, N_2 bubbled through the reaction mixture for 15 mins. 8-Iodoisoquinoline (0.102 g, 0.4 mmol, 1 eq.) in CH_3CN (2 mL) was added via syringe and the reaction mixture stirred at rt for an additional 18 h. After removal of the solvent *in vacuo* the residue was dissolved in CH_2Cl_2 (25 mL) and washed with EDTA solution (25 ml). The aqueous phase was extracted with CH_2Cl_2 and the combined organic extracts dried ($MgSO_4$) and the solvent removed *in vacuo*. Following purification by column chromatography on silica (step gradient acetone/CH_2Cl_2 0:100 to 30:70 in 10% increments) the product was obtained as a beige solid (0.137 g, 90%). ^1H NMR (400 MHz, $CDCl_3$) δ: 9.85 (s, 1H, H_c/H_n), 9.39 (s, 1H, H_c/H_n), 8.67 (d, *J* = 5.9 Hz, 1H, H_b/H_m), 8.62 (d, *J* = 5.9 Hz, 1H, H_b/H_m), 8.25 (d, *J* = 5.9 Hz, 1H, H_a/H_l), 8.04-8.00 (m, 2H, H_d/H_k, H_f/H_i), 7.91-7.87 (m, 2H, H_d/H_k, H_f/H_i), 7.80-7.75 (m, 2H, H_a/H_l, H_e/H_j), 7.70 (app. d, *J* = 0.7 Hz, 4H, H_g, H_h), 7.66 (dd, *J* = 8.2, 7.3 Hz, 1H, H_e/H_j). ^{13}C NMR (101 MHz, $CDCl_3$) δ: 152.2, 150.1, 143.0, 141.8, 136.5, 136.4, 135.0, 132.3, 132.1, 132.0, 131.1, 128.7, 128.5, 128.0, 127.4, 127.4, 123.5, 123.0, 122.1, 121.6, 120.4, 119.5, 96.2, 95.4, 88.1, 87.4.

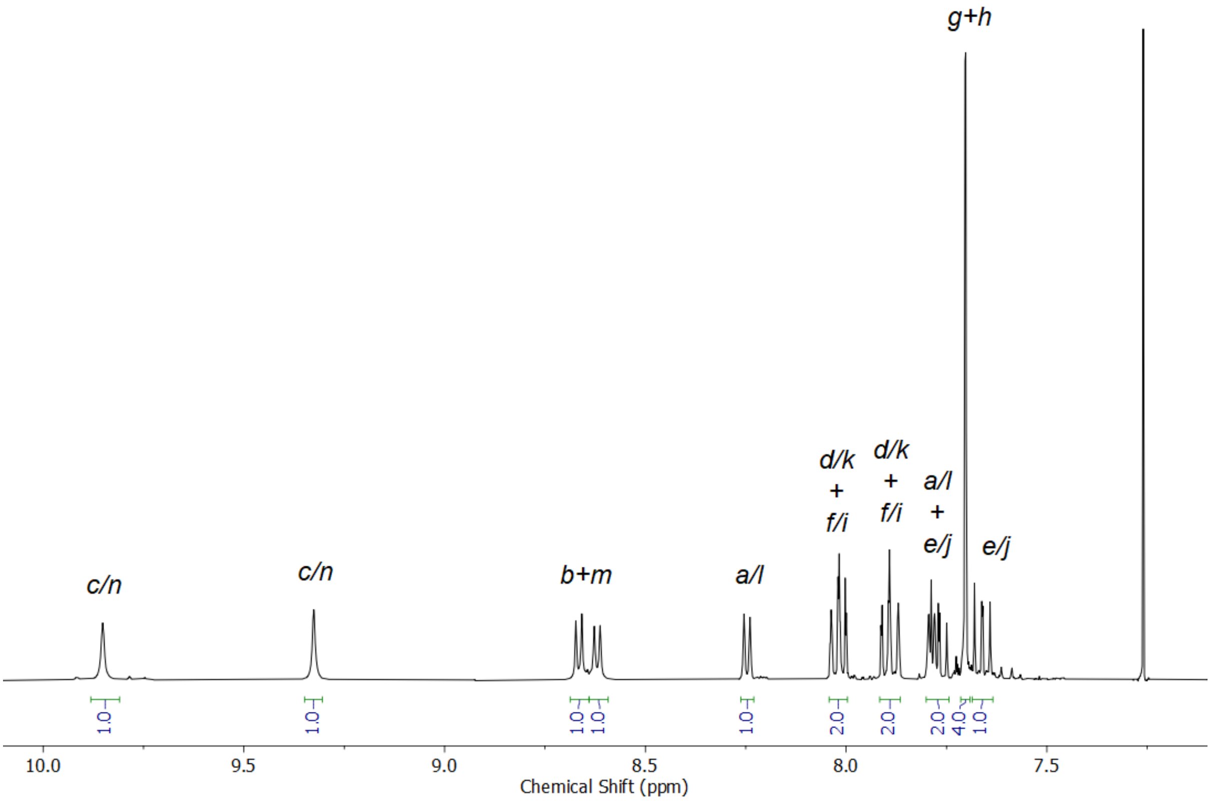

Figure S70 ^1H NMR ($CDCl_3$, 400 MHz) of **5B4**.

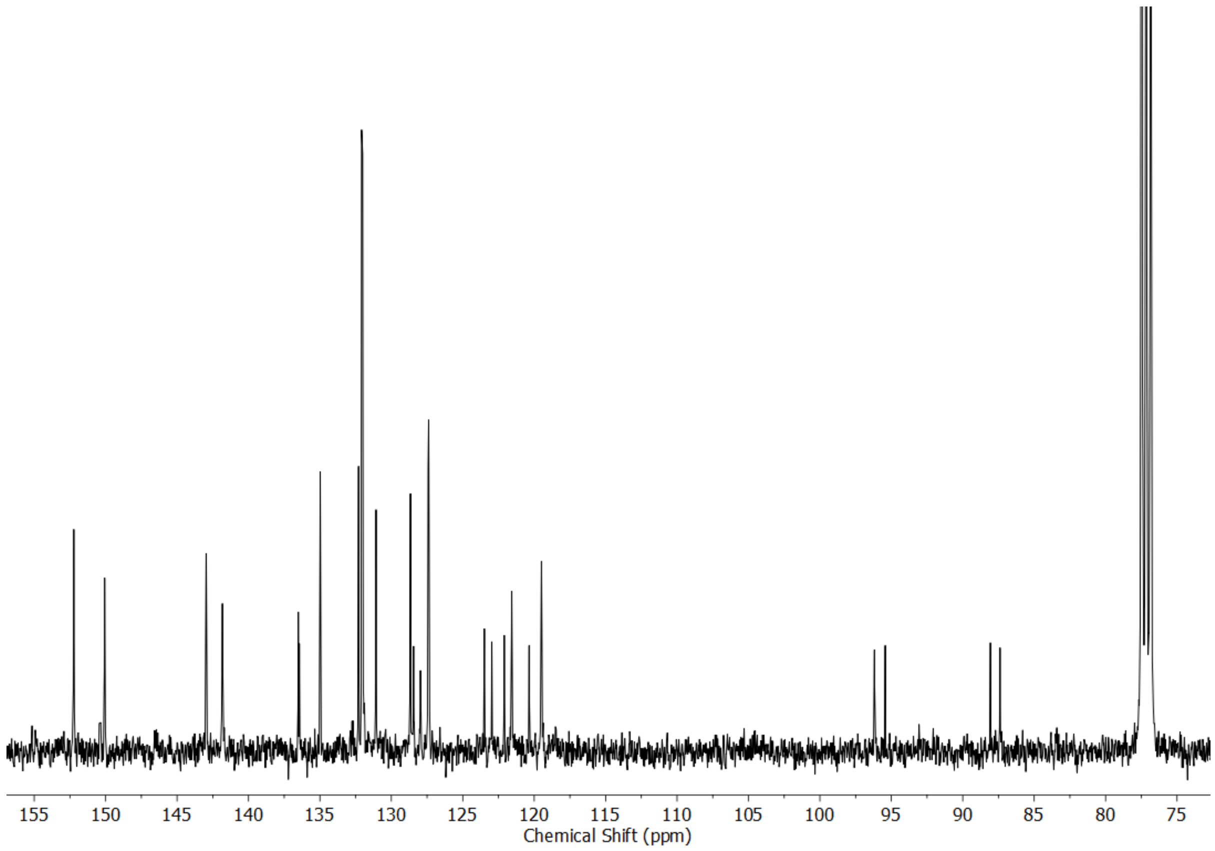

Figure S71 ^{13}C NMR ($CDCl_3$, 101 MHz) of **5B4**.

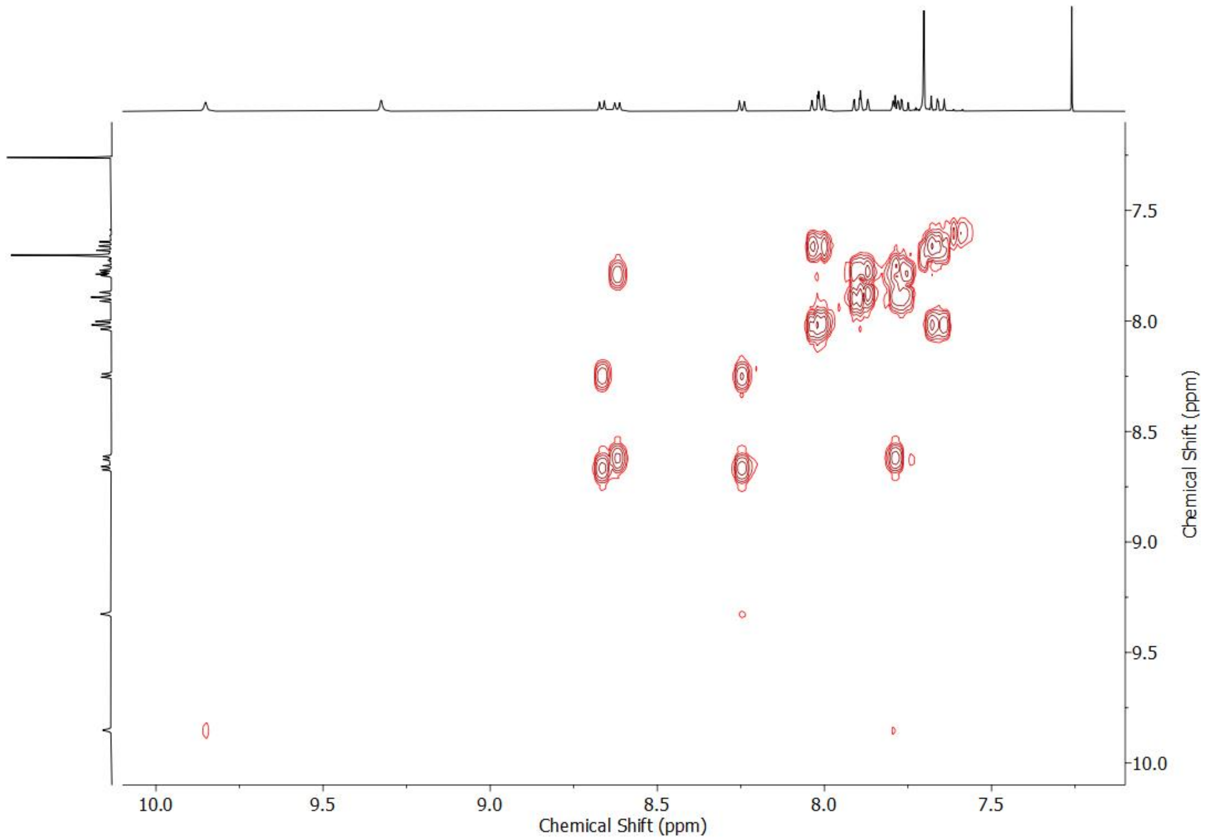

Figure S72 COSY NMR ($CDCl_3$) of **5B4**.

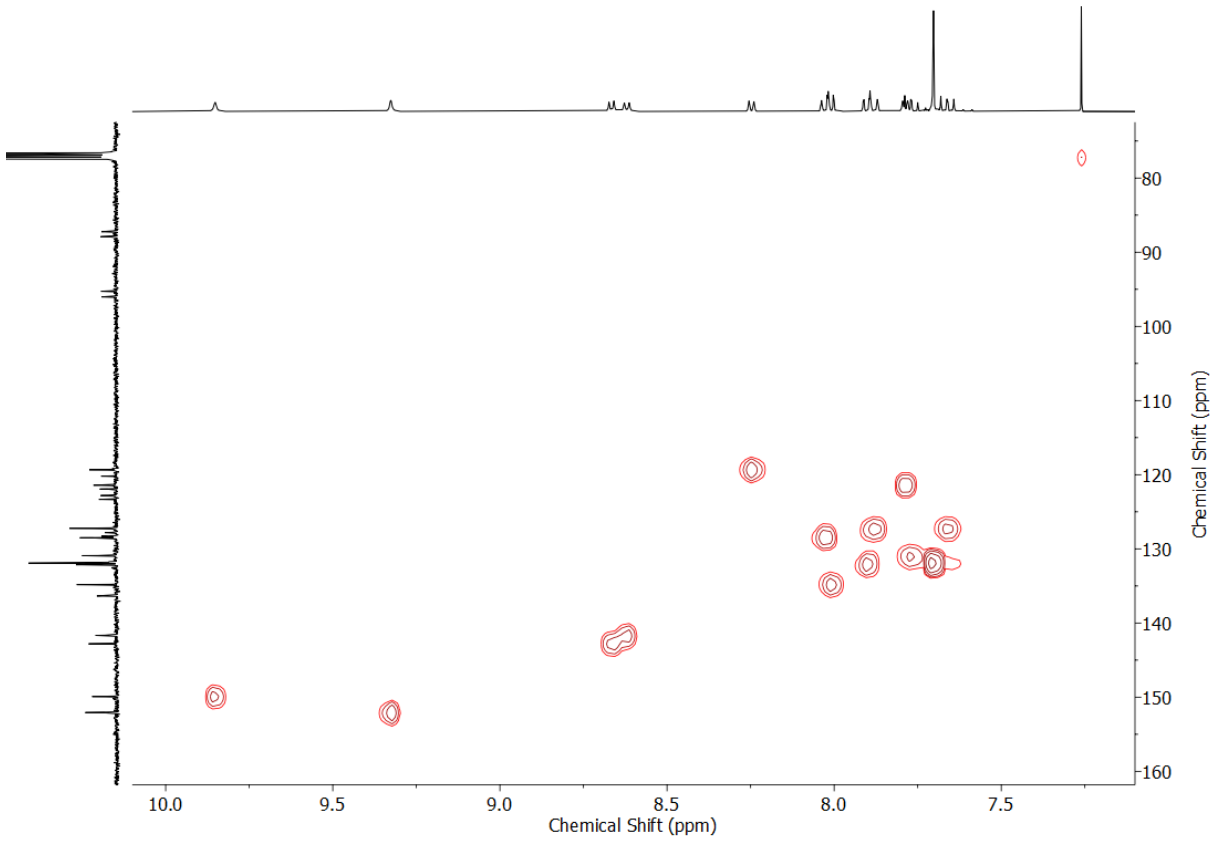

Figure S73 HSQC NMR ($CDCl_3$) of **5B4**.

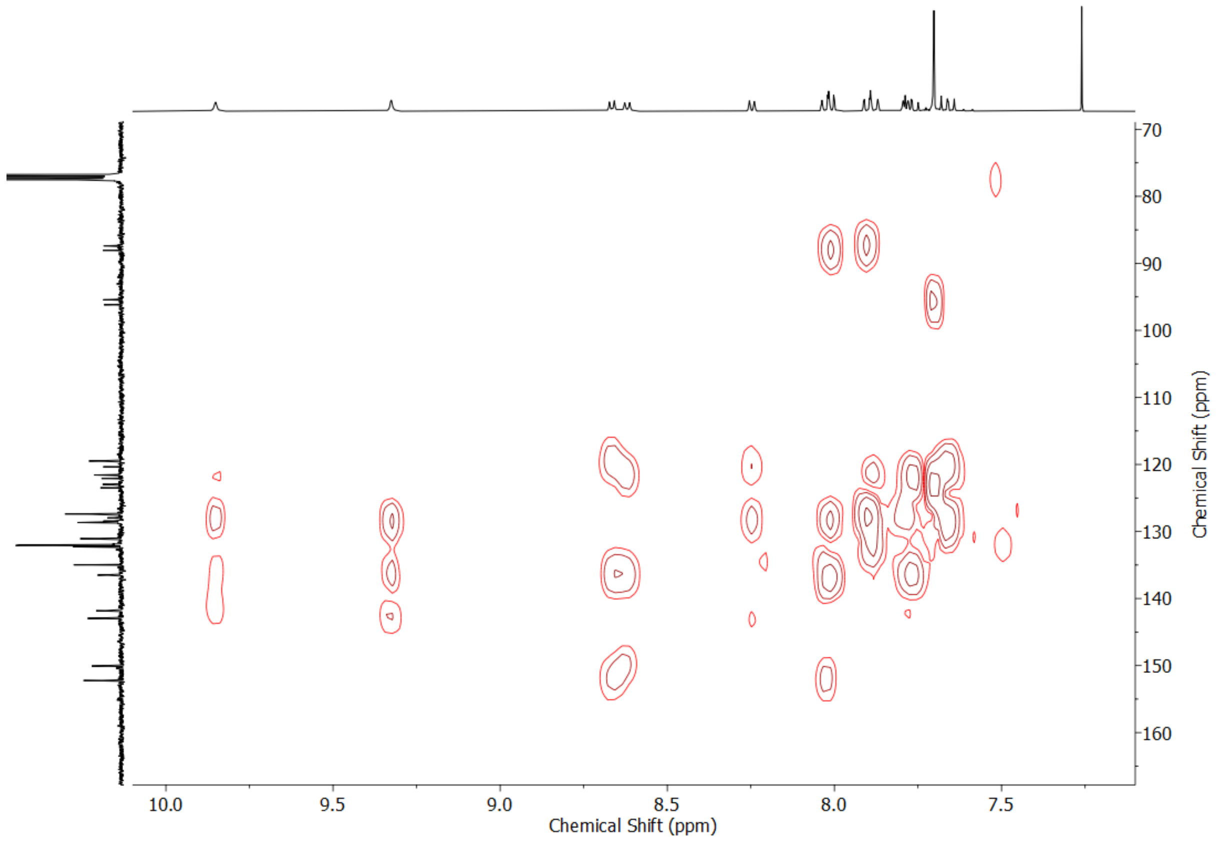

Figure S74 HMBC NMR ($CDCl_3$) of **5B4**.

Synthesis of $[Pd_2(\mathbf{5A1})_4](BF_4)_4$

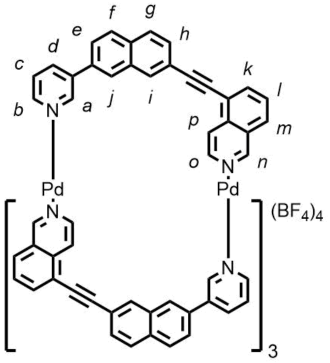

5A1 (10.7 mg, 0.030 mmol) and $[Pd(CH_3CN)_4](BF_4)_2$ (6.7 mg, 0.015 mmol) were sonicated in d_6-DMSO (0.75 mL) until all solids were dissolved. Quantitative conversion to $[Pd_2(\mathbf{5A1})_4](BF_4)_4$ was observed by ^{1}H NMR. ^{1}H NMR (400 MHz, d_6-DMSO) δ: 10.17 (s, 4H, H_n), 9.88 (d, J = 2.0 Hz, 4H, H_a), 9.80 (d, J = 6.7 Hz, 4H, H_o), 9.66 (dd, J = 5.8, 1.3 Hz, 4H, H_b), 8.72 (s, 4H, H_i), 8.39-8.65 (m, 12H, H_d, H_j, H_p), 8.36 (dt, J = 8.7, 1.0 Hz, 4H, H_m), 8.23 (dd, J = 7.2, 1.1 Hz, 4H, H_k), 8.09 (d, J = 8.8 Hz, 4H, H_f), 8.04-7.97 (m, 12H, H_c, H_e, H_g), 7.91 (dd, J = 8.4, 7.2 Hz, 4H, H_l), 7.69 (dd, J = 8.3, 1.6 Hz, 4H, H_h). Diffusion coefficient (500 MHz, d_6-DMSO) D: 1.20×10^{-10} m^2s^{-1}. ^{13}C NMR (101 MHz, d_6-DMSO) δ: 156.7, 150.0, 149.1, 142.1, 138.5, 137.6, 136.9, 135.7, 133.1, 132.8, 132.5, 132.3, 129.9, 129.7, 129.3, 128.5, 128.5, 127.9, 125.8, 125.6, 122.6, 119.7, 119.3, 96.8, 85.5. ESI-MS m/z = 863.10 $\{[Pd_2(\mathbf{5A1})_4](HCO_2)_2\}^{2+}$ calc. 863.16.

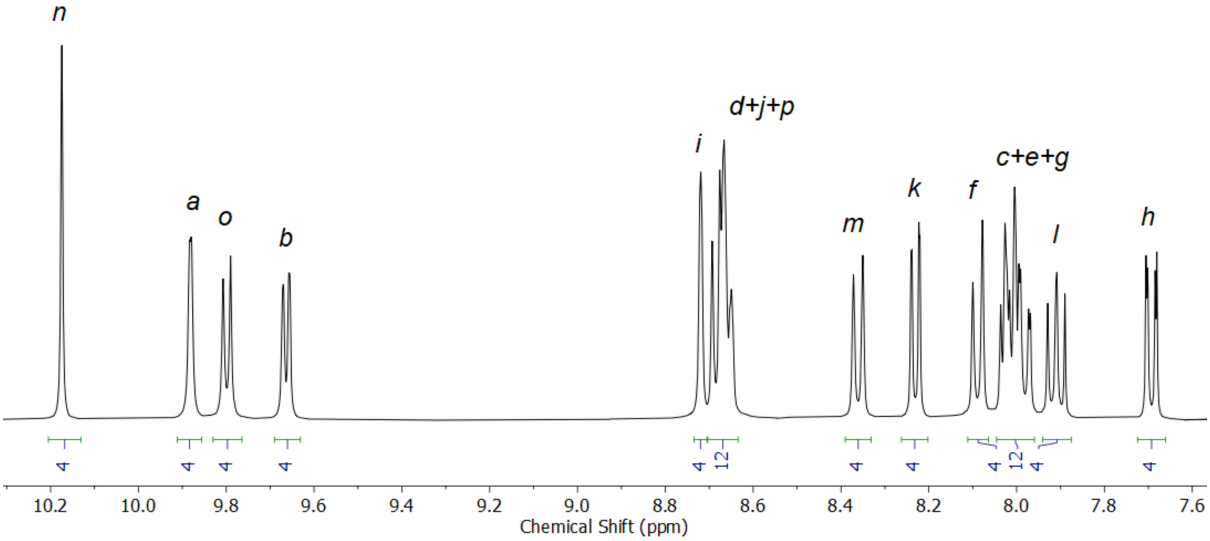

Figure S75 ^{1}H NMR (d_6-DMSO, 400 MHz) of $[Pd_2(\mathbf{5A1})_4](BF_4)_4$.

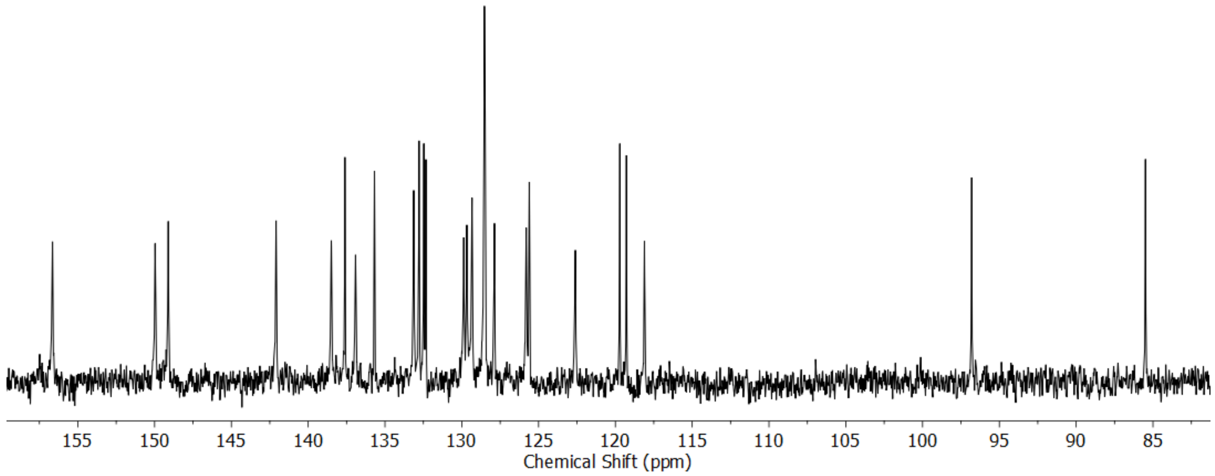

Figure S76 ^{13}C NMR (d_6-DMSO, 101 MHz) of $[Pd_2(\mathbf{5A1})_4](BF_4)_4$.

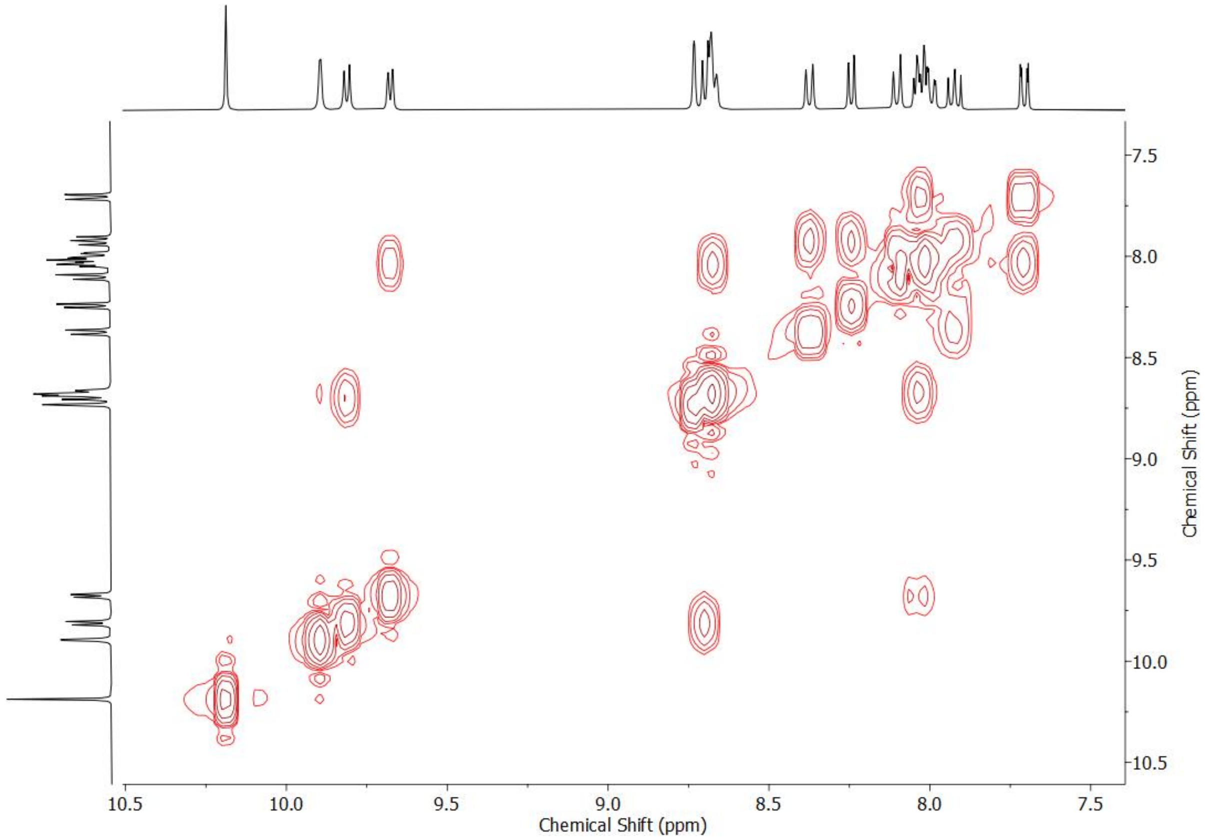

Figure S77 COSY NMR (d_6-DMSO) of $[Pd_2(\mathbf{5A1})_4](BF_4)_4$.

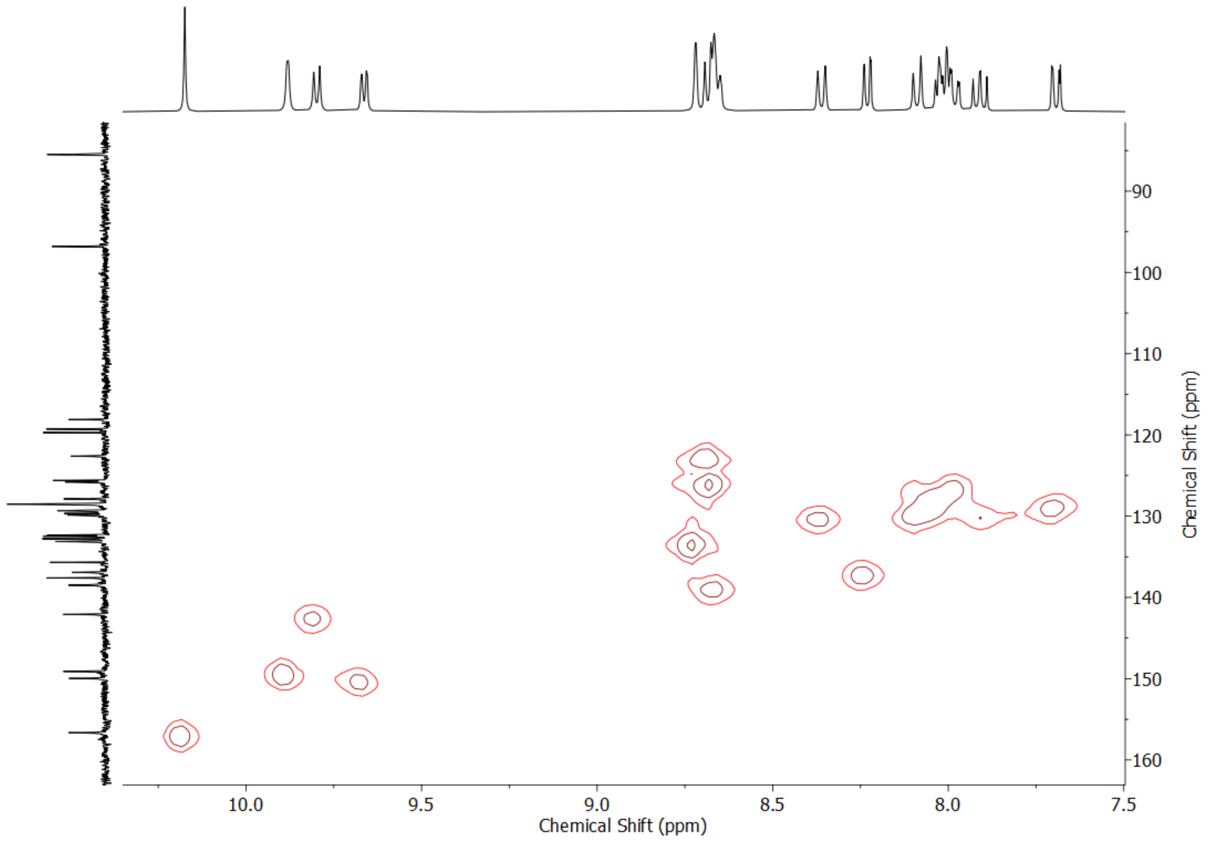

Figure S78 HSQC NMR (d_6-DMSO) of $[Pd_2(\mathbf{5A1})_4](BF_4)_4$.

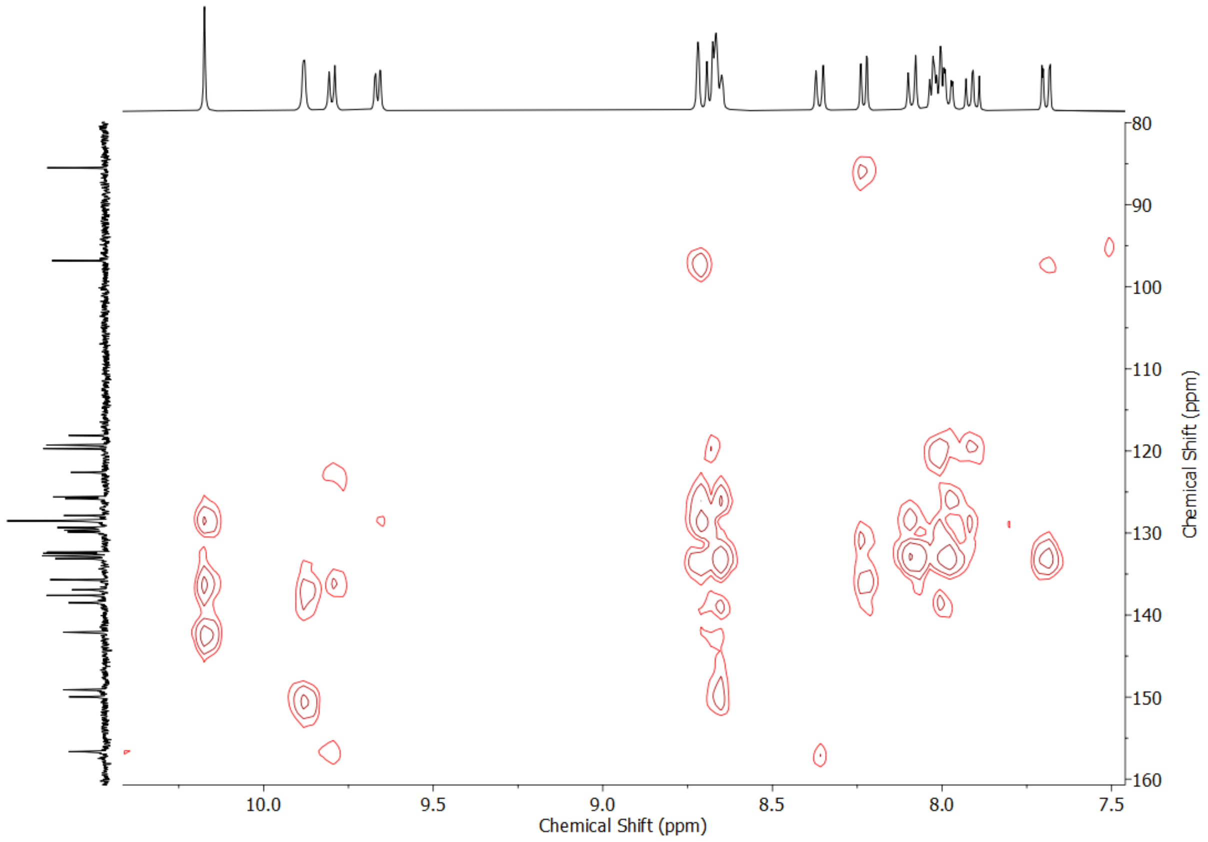

Figure S79 HMBC NMR (d_6-DMSO) of $[Pd_2(\mathbf{5A1})_4](BF_4)_4$.

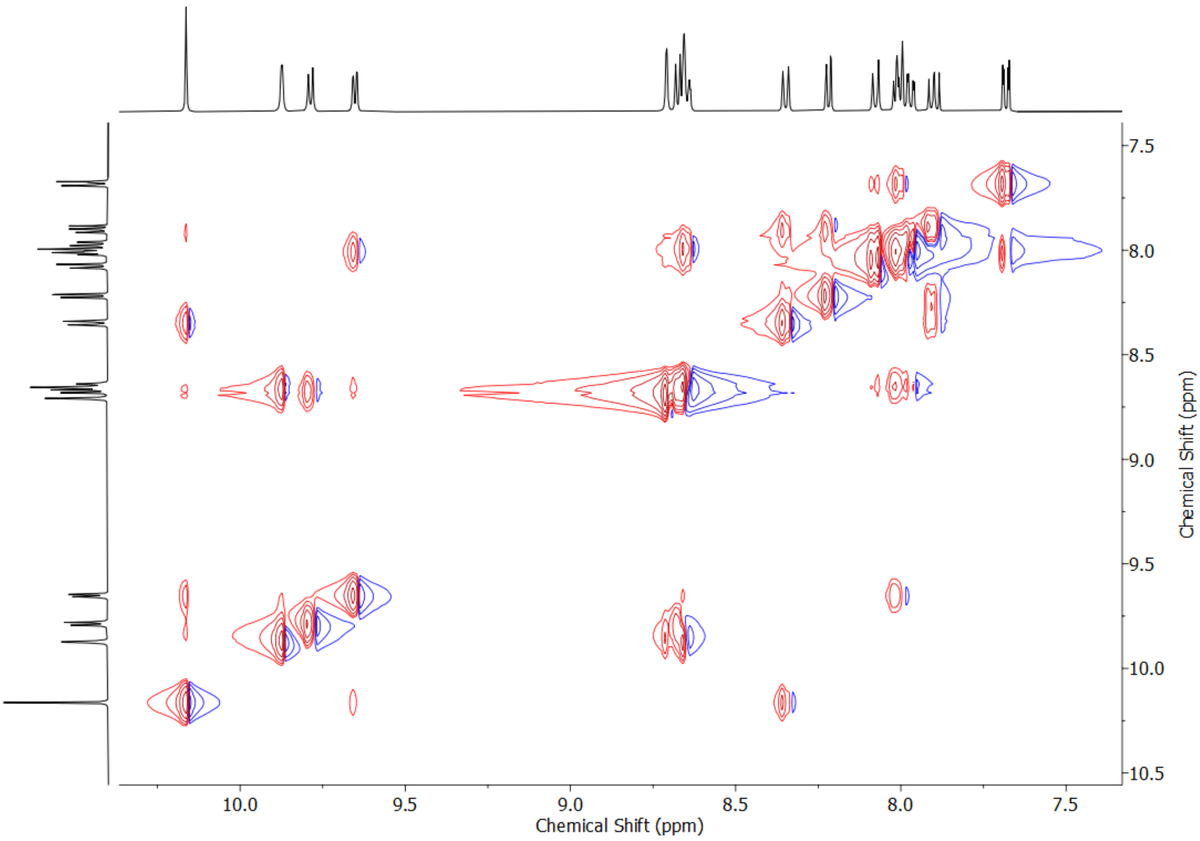

Figure S80 NOESY NMR (d_6-DMSO, 500 MHz) of $[Pd_2(\mathbf{5A1})_4](BF_4)_4$.

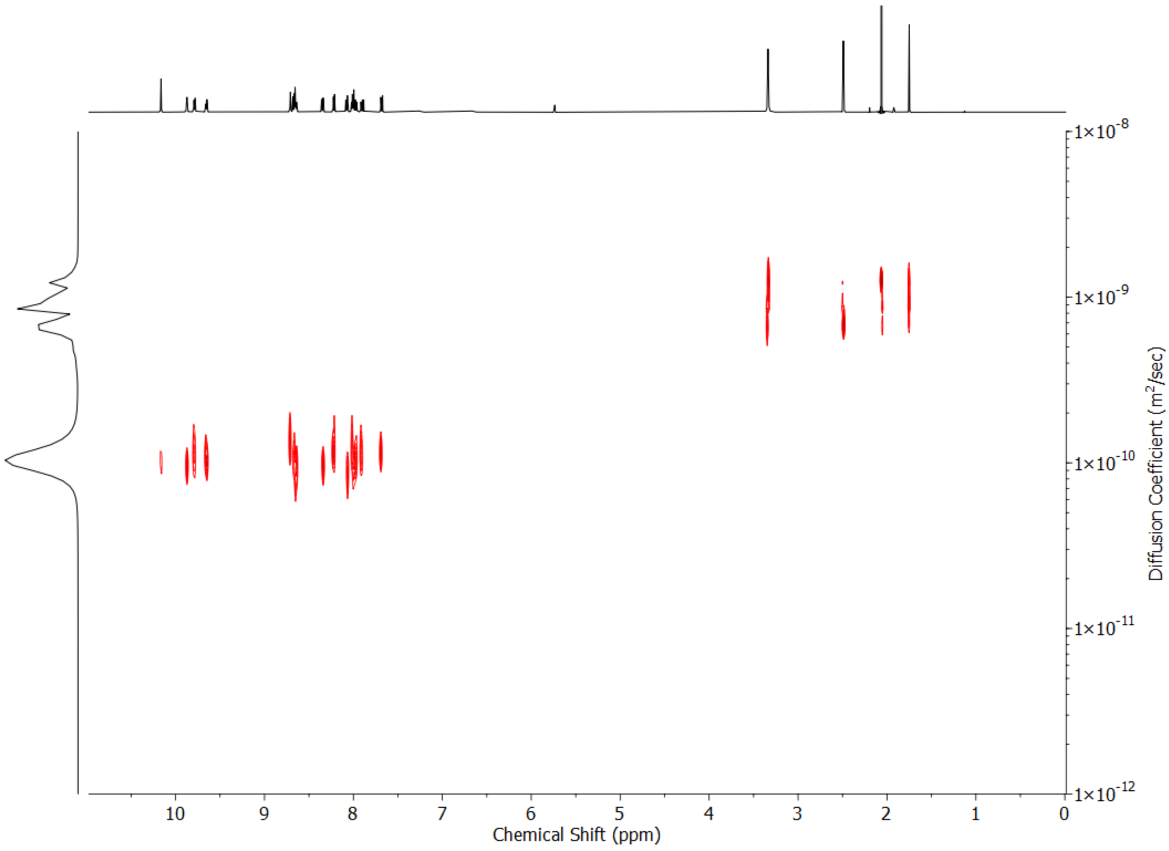

Figure S81 DOSY NMR (d_6-DMSO, 500 MHz) of $[Pd_2(\mathbf{5A1})_4](BF_4)_4$.

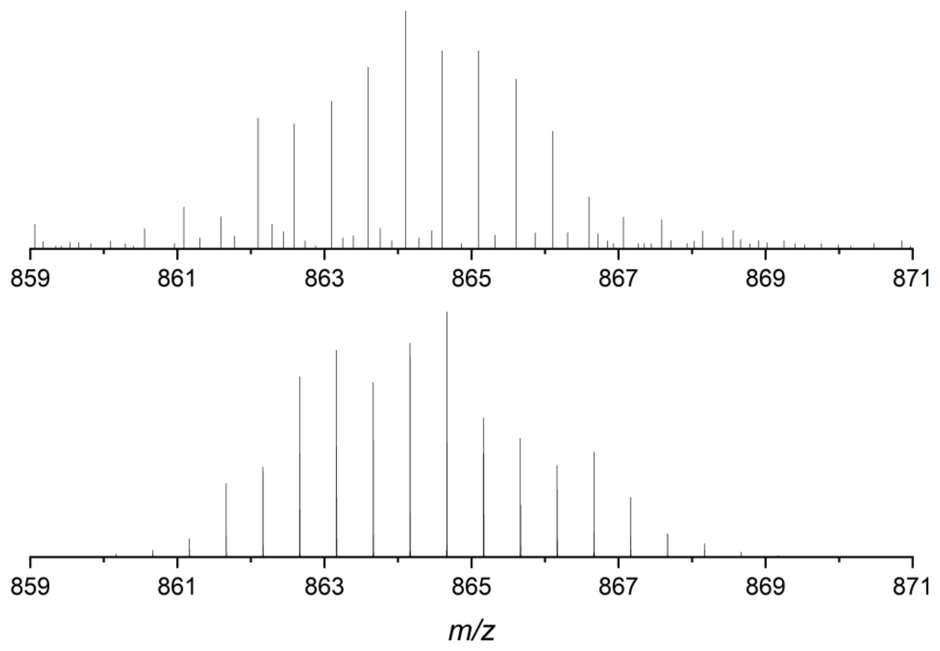

Figure S82 Observed (top) and calculated (bottom) isotopic patterns for $\{[Pd_2(\mathbf{5A1})_4](HCO_2)_2\}^{2+}$.

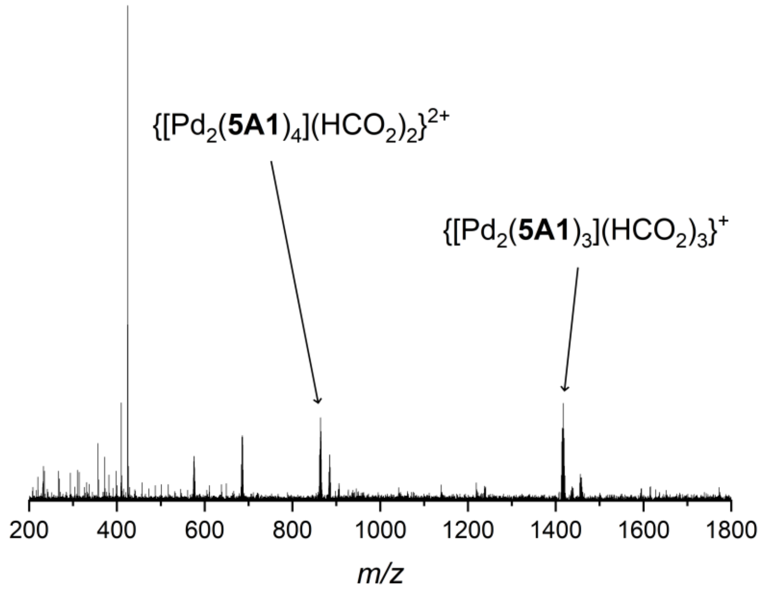

Figure S83 ESI-MS of $[Pd_2(\mathbf{5A1})_4](BF_4)_4$.

Synthesis of $[Pd_2(5A3)_4](BF_4)_4$

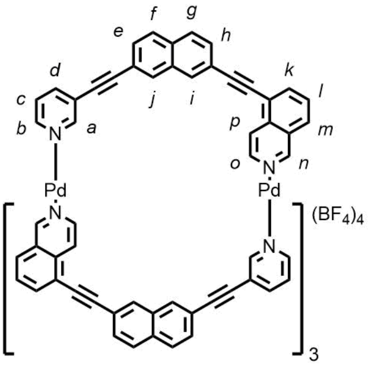

5A3 (11.4 mg, 0.030 mmol) and $[Pd(CH_3CN)_4](BF_4)_2$ (6.7 mg, 0.015 mmol) were sonicated in d_6-DMSO (0.75 mL) until all solids were dissolved. After heating at 60 °C for 3 h, quantitative conversion to $[Pd_2(\mathbf{5A3})_4](BF_4)_4$ was observed by ^{1}H NMR. ^{1}H NMR (500 MHz, d_6-DMSO) δ: 10.11 (s, 4H, H_n), 9.94 (d, J = 1.9 Hz, 4H, H_a), 9.87 (d, J = 6.8 Hz, 4H, H_o), 9.57 (dd, J = 5.9, 1.4 Hz, 4H, H_b), 8.79 (d, J = 6.7 Hz, 4H, H_p), 8.60 (s, 4H, H_i), 8.35 (d, J = 8.6 Hz, 4H, H_m), 8.33 (app. dt, J = 8.0, 1.6 Hz, 4H, H_d), 8.26 (dd, J = 7.2 ,1.1 Hz, 4H, H_k), 8.22 (s, 4H, H_j), 8.08-8.05 (m, 8H, H_f, H_g), 7.94-7.89 (m, 8H, H_c, H_l), 7.77-7.73 (m, 8H, H_e, H_h). Diffusion coefficient (500 MHz, d_6-DMSO) D: 9.61 × 10^{-11} m^2s^{-1}. ^{13}C NMR (126 MHz, d_6-DMSO) δ: 156.4, 152.9, 150.7, 143.0, 142.8, 137.0, 135.6, 132.7, 132.2, 132.0, 130.9, 129.9, 129.6, 129.6, 129.4, 129.0, 128.8, 128.5, 127.5, 123.0, 122.0, 120.1, 119.5, 119.2, 96.3, 94.7, 85.8, 85.2. ESI-MS m/z = 953.16 $\{[Pd_2(\mathbf{5A3})_4](BF_4)_2\}^{2+}$ calc. 953.17.

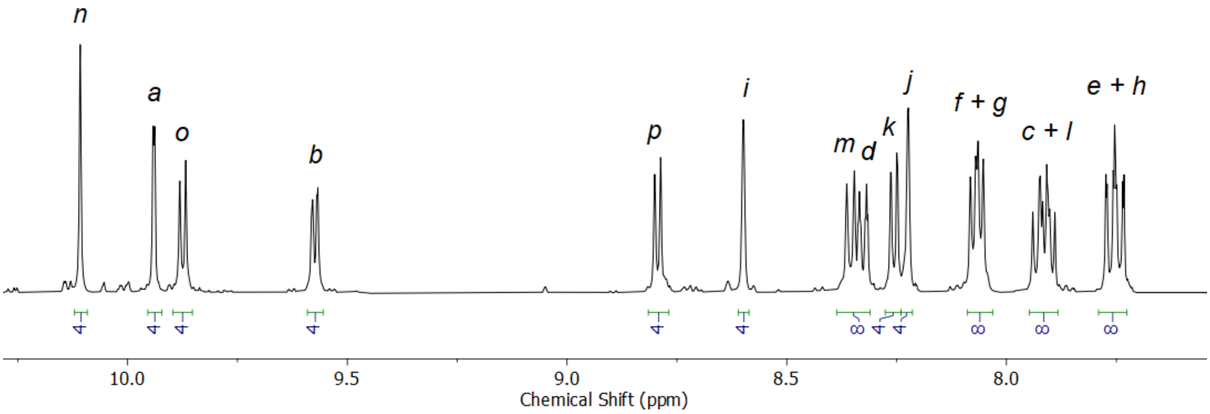

Figure S84 ^{1}H NMR (d_6-DMSO, 500 MHz) of $[Pd_2(\mathbf{5A3})_4](BF_4)_4$.

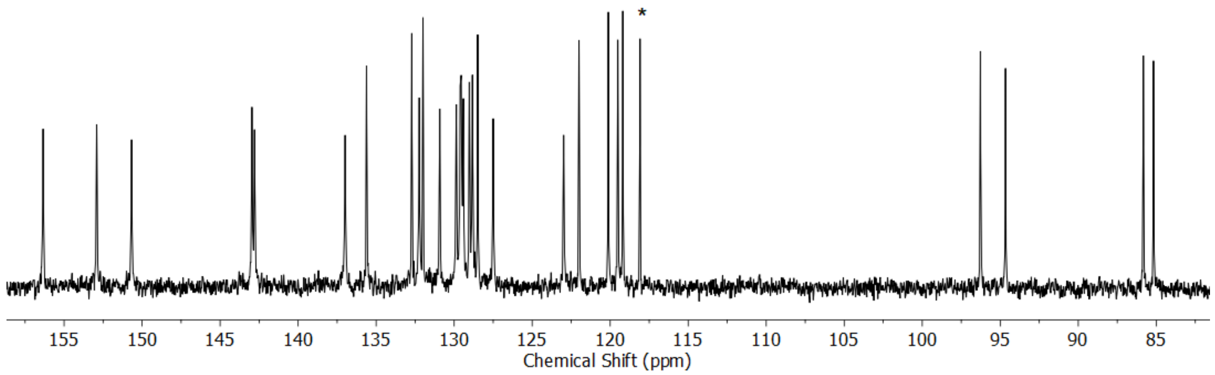

Figure S85 ^{1}H NMR (d_6-DMSO, 126 MHz) of $[Pd_2($**5A3**$)_4](BF_4)_4$.

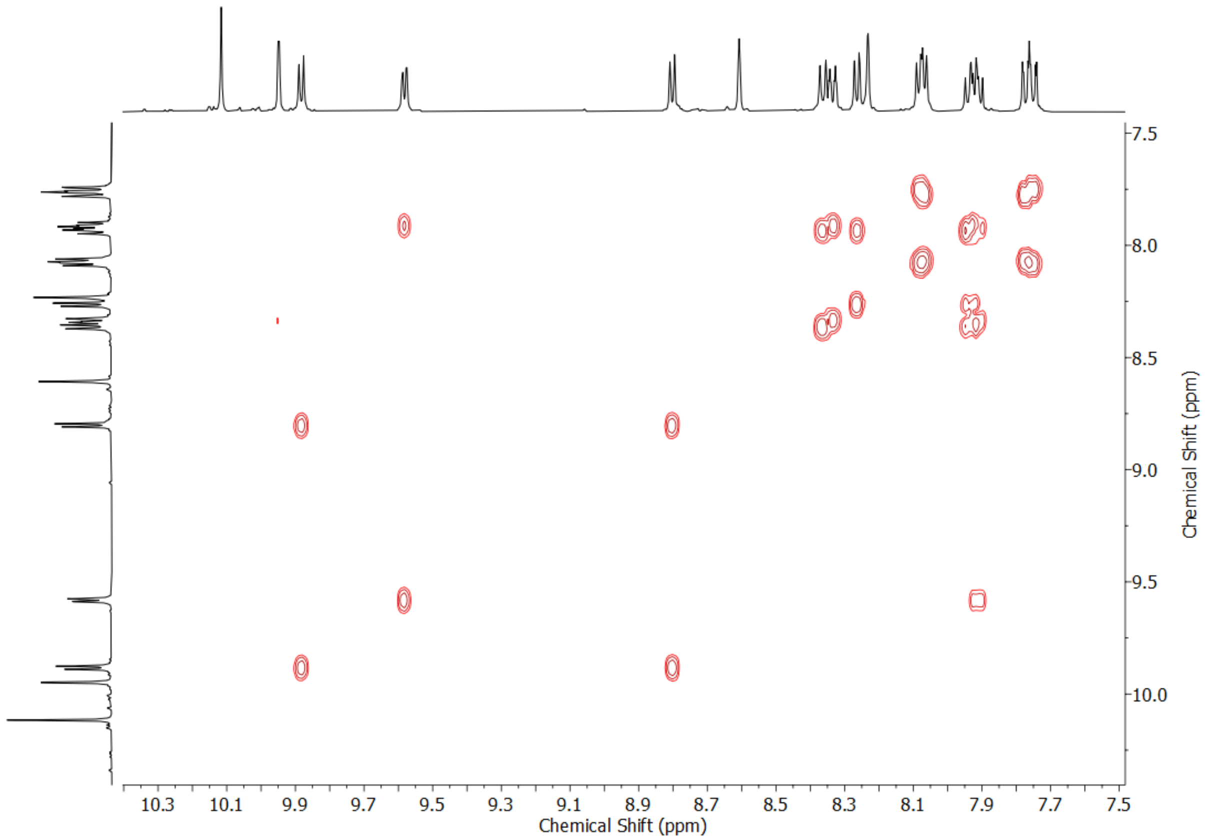

Figure S86 COSY NMR (d_6-DMSO) of $[Pd_2($**5A3**$)_4](BF_4)_4$.

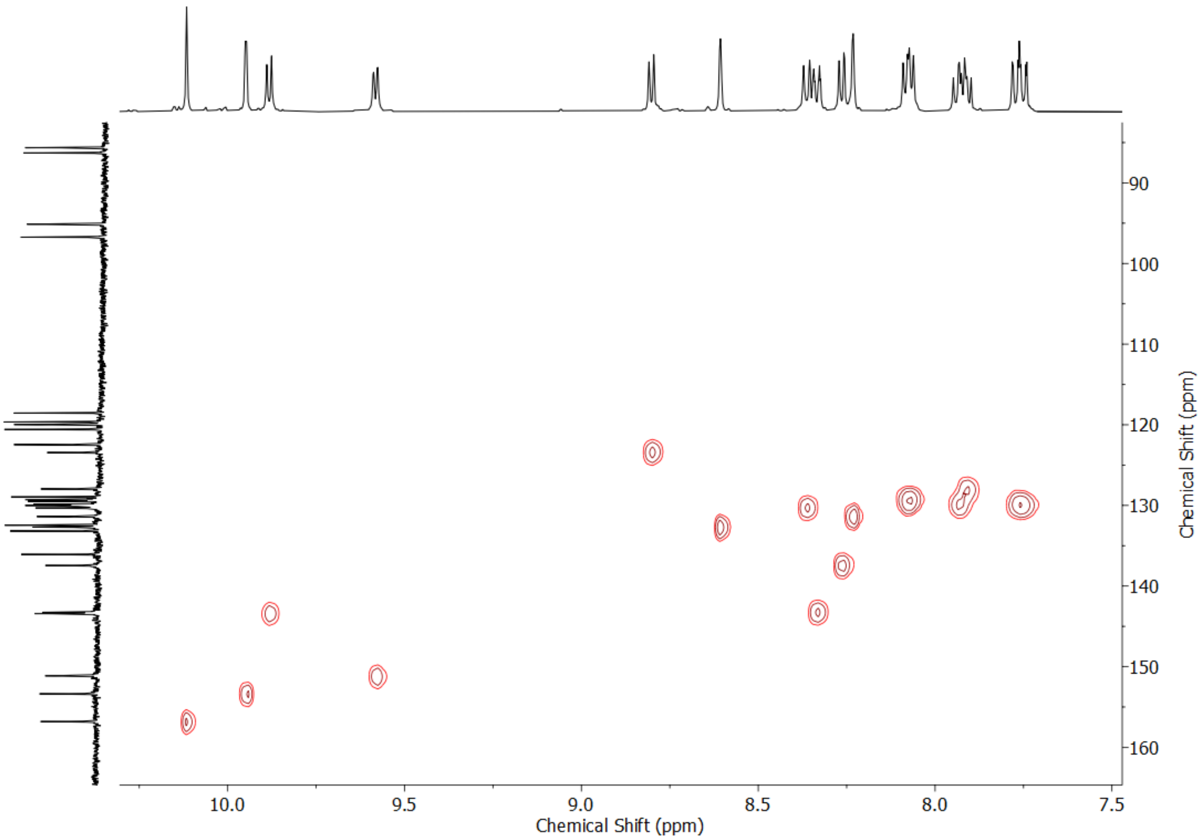

Figure S87 HSQC NMR (d_6-DMSO) of $[Pd_2(\mathbf{5A3})_4](BF_4)_4$.

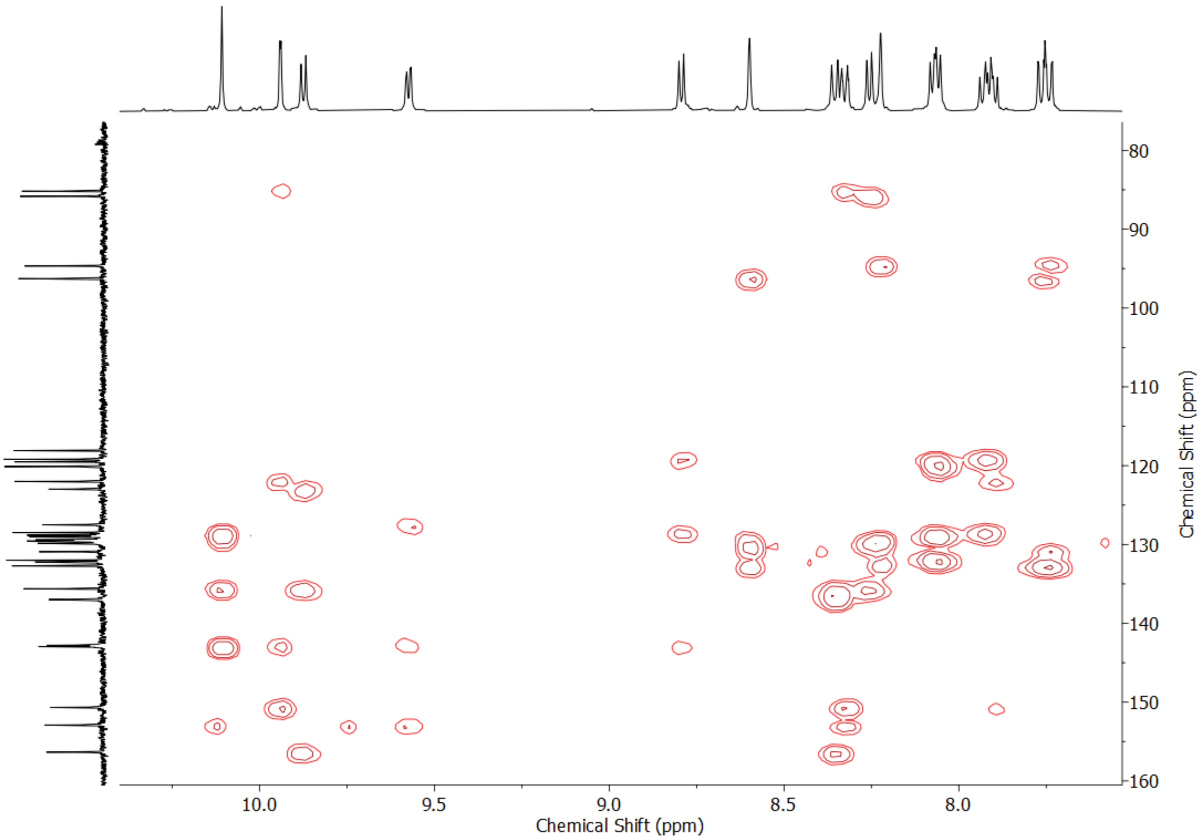

Figure S88 HMBC NMR (d_6-DMSO) of $[Pd_2(\mathbf{5A3})_4](BF_4)_4$.

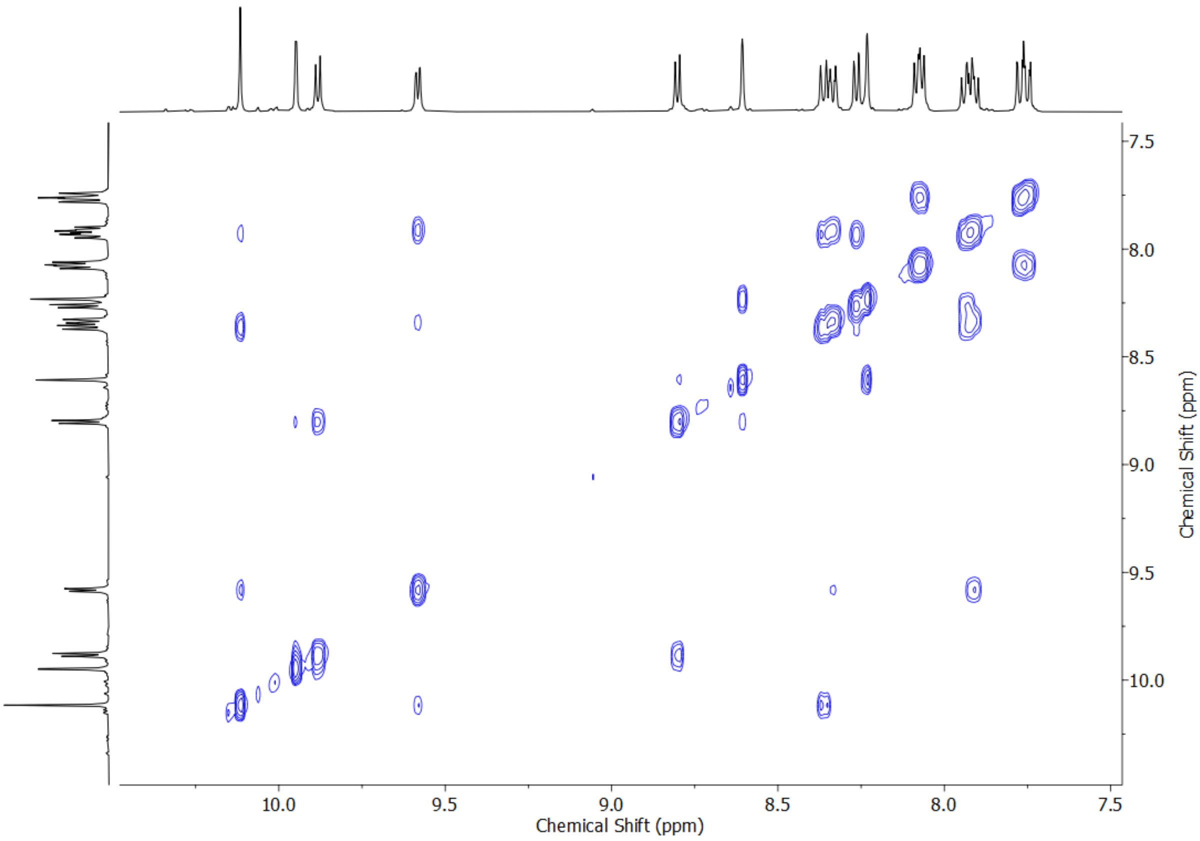

Figure S89 NOESY NMR (d_6-DMSO) of $[Pd_2(\mathbf{5A3})_4](BF_4)_4$.

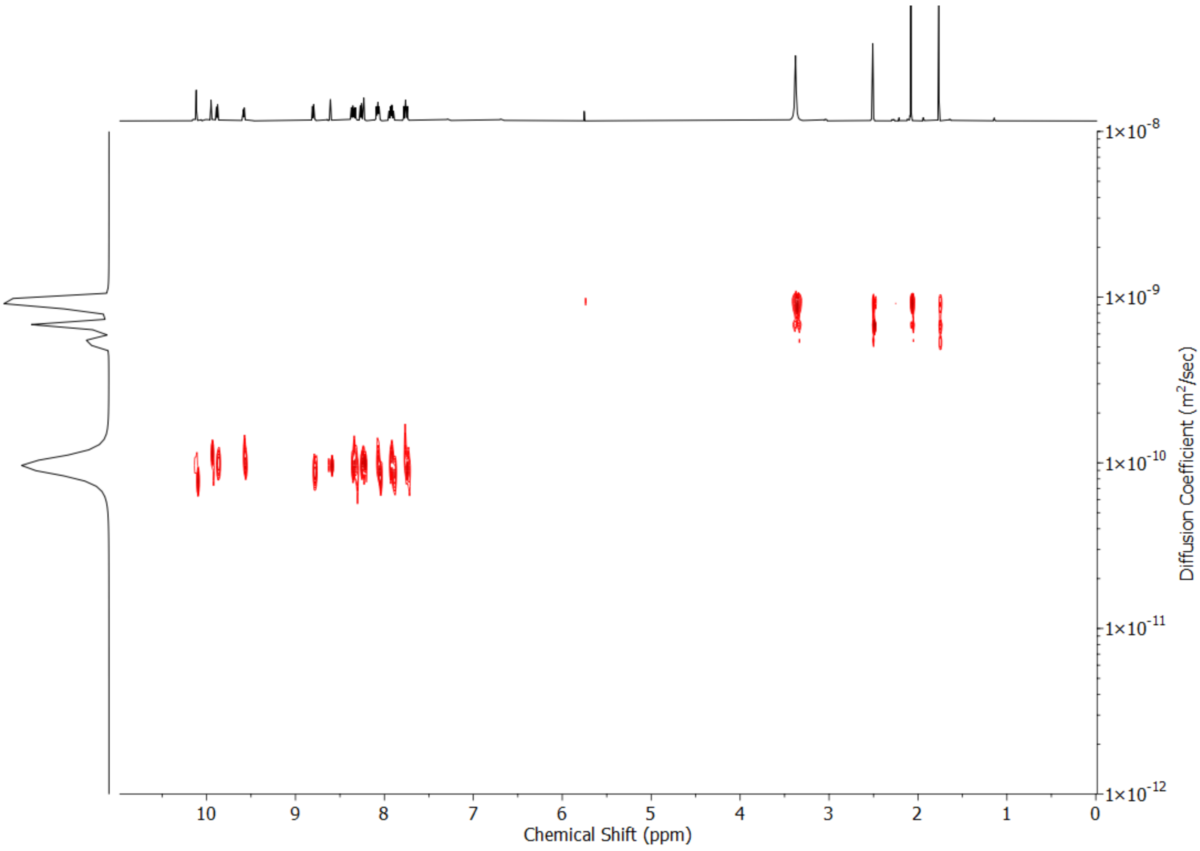

Figure S90 DOSY NMR (d_6-DMSO, 500 MHz) of $[Pd_2(\mathbf{5A3})_4](BF_4)_4$.

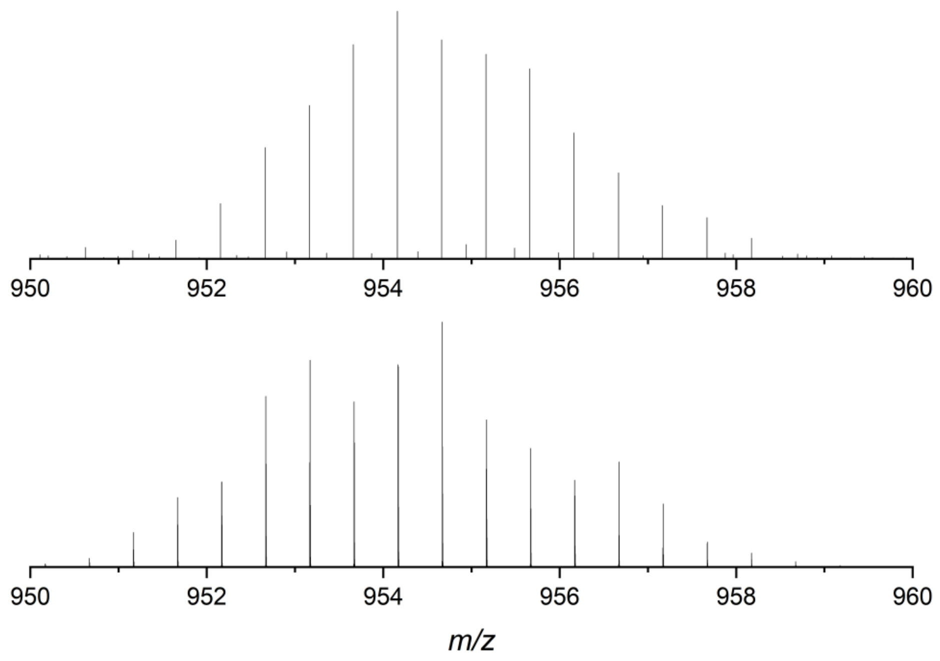

Figure S91 Observed (top) and calculated (bottom) isotopic patterns for $\{[Pd_2(\mathbf{5A3})_4](BF_4)_2\}^{2+}$.

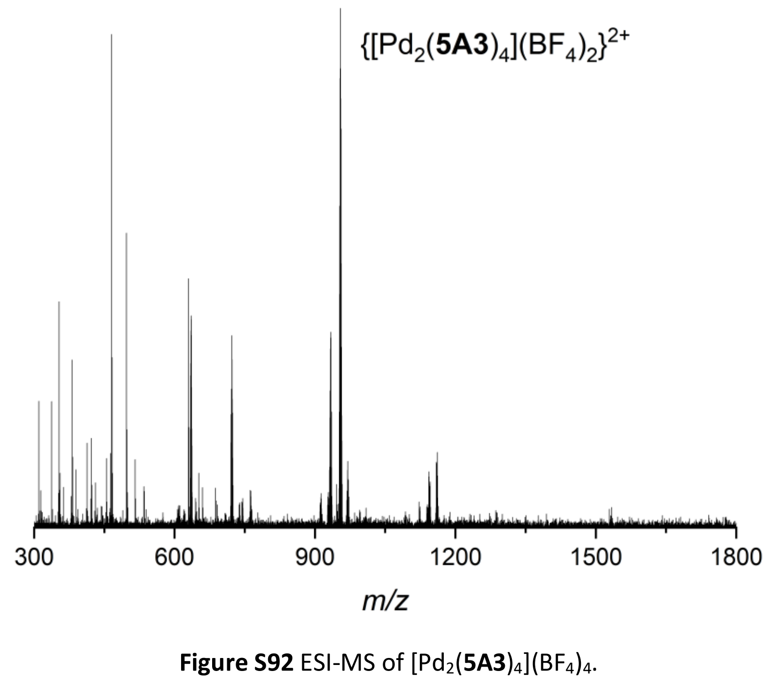

Figure S92 ESI-MS of $[Pd_2(\mathbf{5A3})_4](BF_4)_4$.

Synthesis of $[Pd_2(4B3)_4](BF_4)_4$

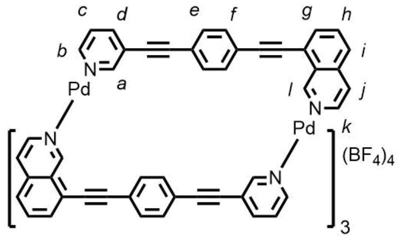

4B3 (9.9 mg, 0.030 mmol) and $[Pd(CH_3CN)_4](BF_4)_2$ (6.7 mg, 0.015 mmol) were sonicated in d_6-DMSO (0.75 mL) until all solids were dissolved. After heating at 50 °C for 2 h, conversion to mostly a single $[Pd_2(\mathbf{4B3})_4](BF_4)_4$ species was observed by ^1H NMR. Signals in the ^1H NMR spectrum were assigned where possible. ^1H NMR (500 MHz, d_6-DMSO) δ: 9.61 (d, J = 1.6 Hz, 4H, H_a), 8.85 (d, J = 0.8 Hz, 4H, H_l), 8.65 (m, 4H, H_b), 8.59 (d, J = 6.6 Hz, 4H, H_k), 8.18 (d, J = 6.2 Hz, 4H, H_j), 7.76 (dd, J = 8.1, 5.7 Hz, 4H, H_c), 7.56 (d, J = 8.5 Hz, 8H, H_e/H_f), 7.46 (d, J = 8.5 Hz, 8H, H_e/H_f). Diffusion coefficient (500 MHz, d_6-DMSO) D: 8.33 × 10^{-11} m^2s^{-1}. ESI-MS m/z = 1793.29 $\{[Pd_2(\mathbf{4B3})_4](BF_4)_3\}^+$ calc. 1793.28; 853.15 $\{[Pd_2(\mathbf{4B3})_4](BF_4)_2\}^{2+}$ calc. 853.14; 539.77 $\{[Pd_2(\mathbf{4B3})_4](BF_4)\}^{3+}$ calc. 539.76.

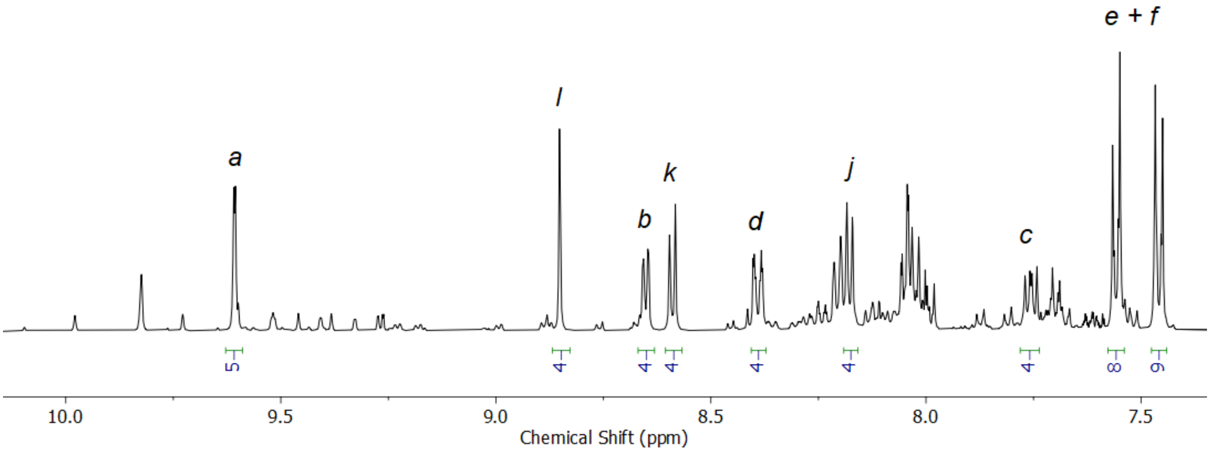

Figure S93 ^1H NMR (d_6-DMSO, 500 MHz) of $[Pd_2(\mathbf{4B3})_4](BF_4)_4$.

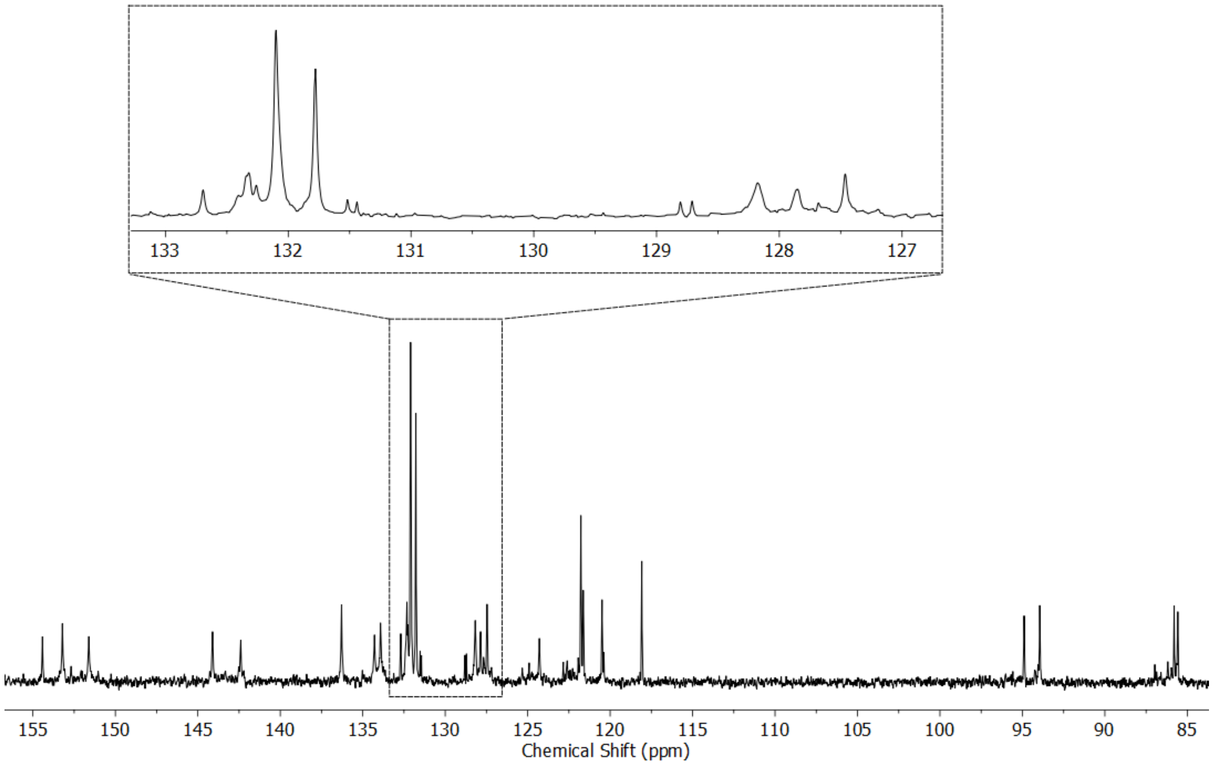

Figure S94 ^{13}C NMR (d_6-DMSO, 126 MHz) of $[Pd_2(\mathbf{4B3})_4](BF_4)_4$.

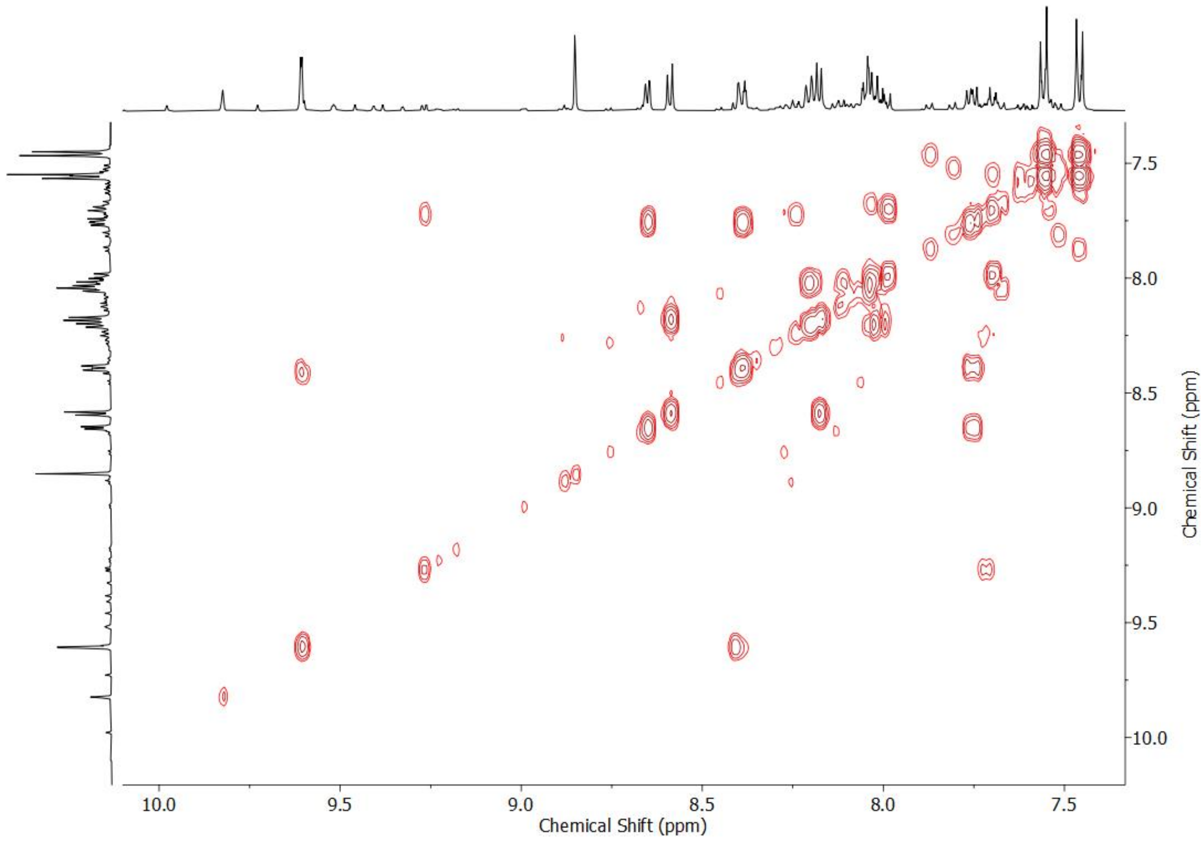

Figure S95 COSY NMR (d_6-DMSO) of $[Pd_2(\mathbf{4B3})_4](BF_4)_4$.

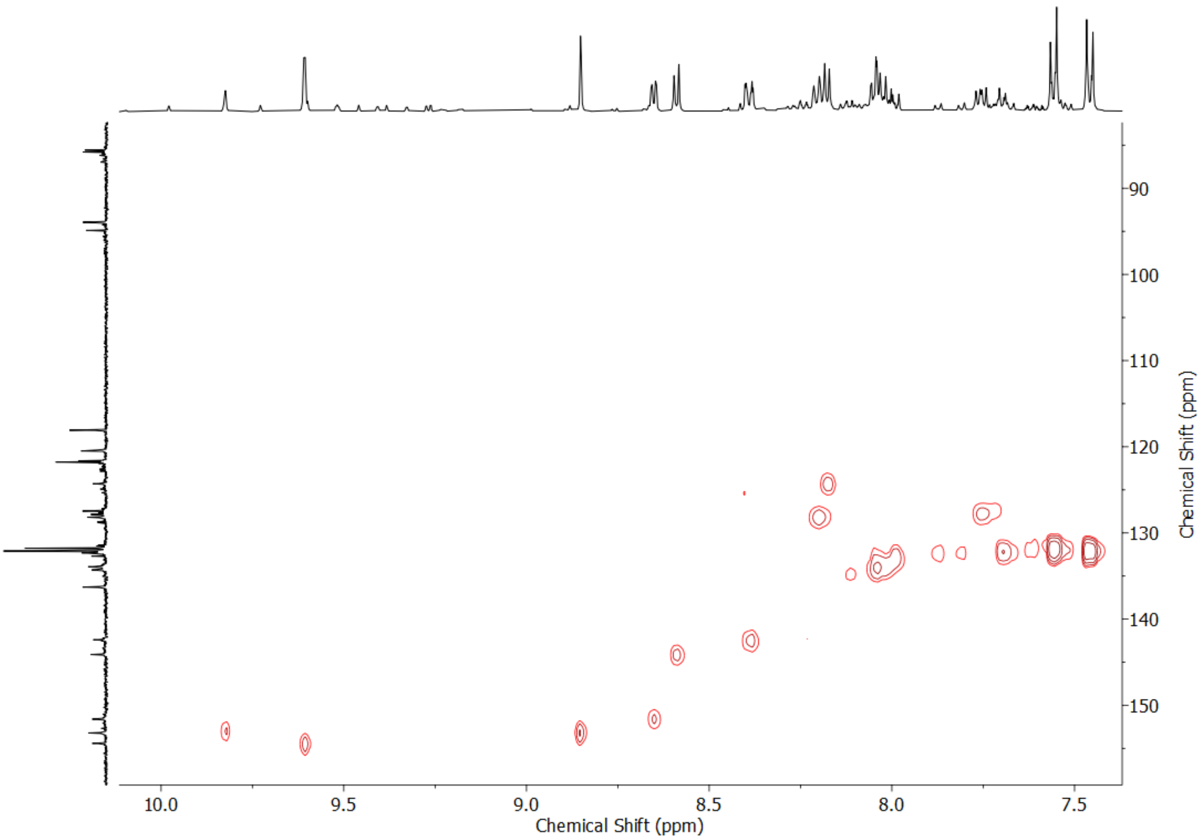

Figure S96 HSQC NMR (d_6-DMSO) of [Pd$_2$(**4B3**)$_4$](BF$_4$)$_4$.

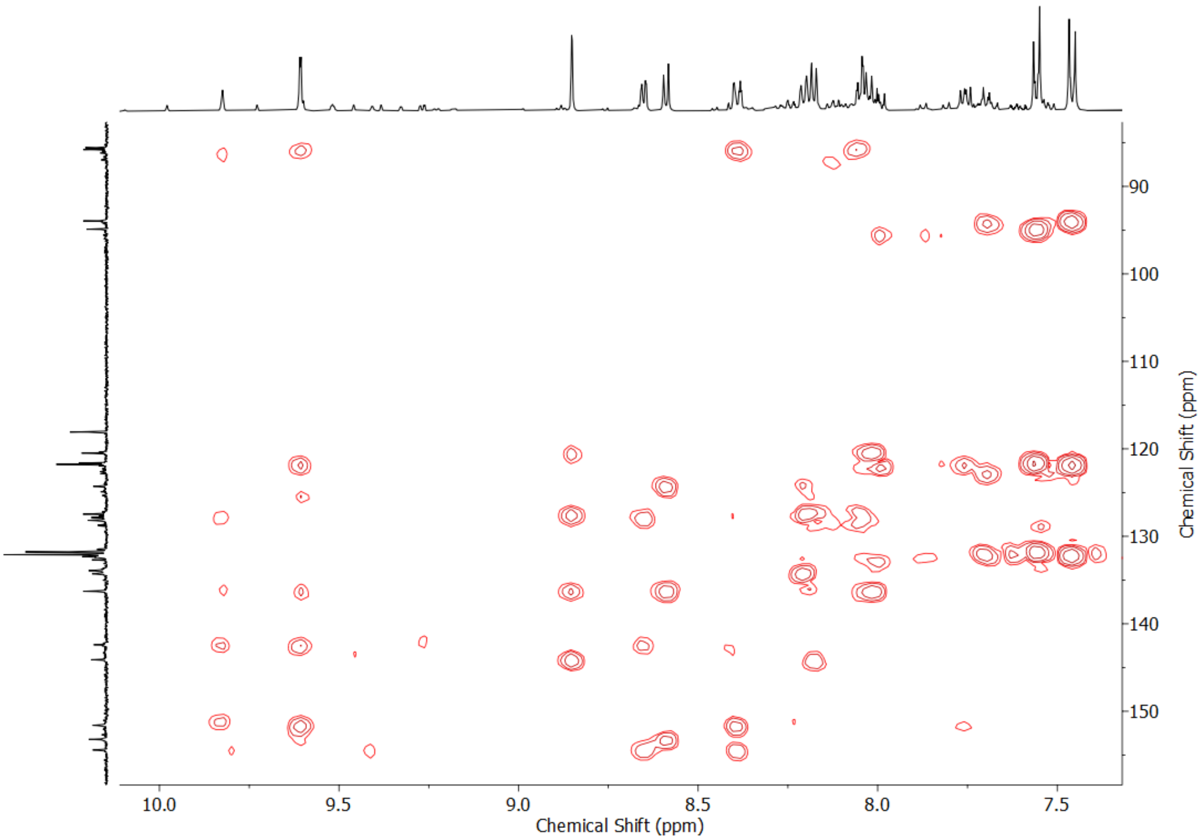

Figure S97 HMBC NMR (d_6-DMSO) of [Pd$_2$(**4B3**)$_4$](BF$_4$)$_4$.

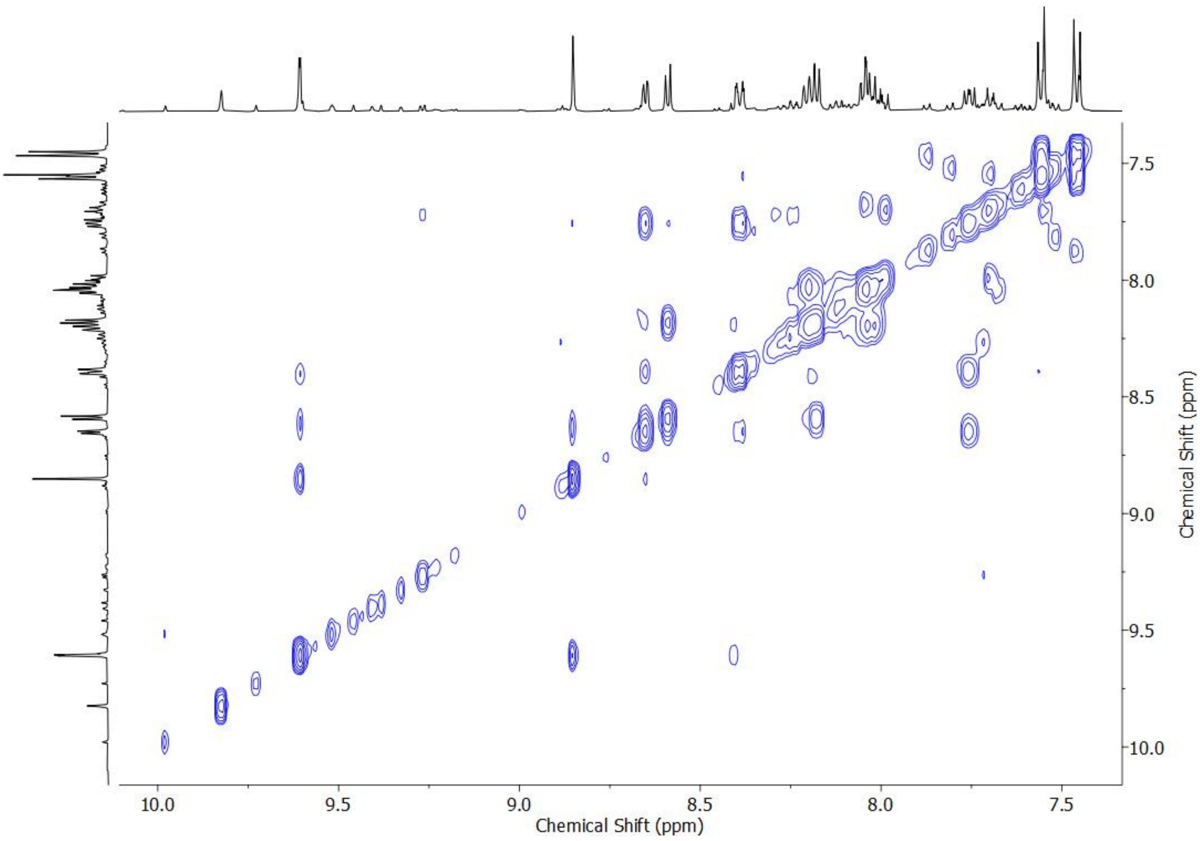

Figure S98 NOESY NMR (d_6-DMSO) of $[Pd_2(\mathbf{4B3})_4](BF_4)_4$.

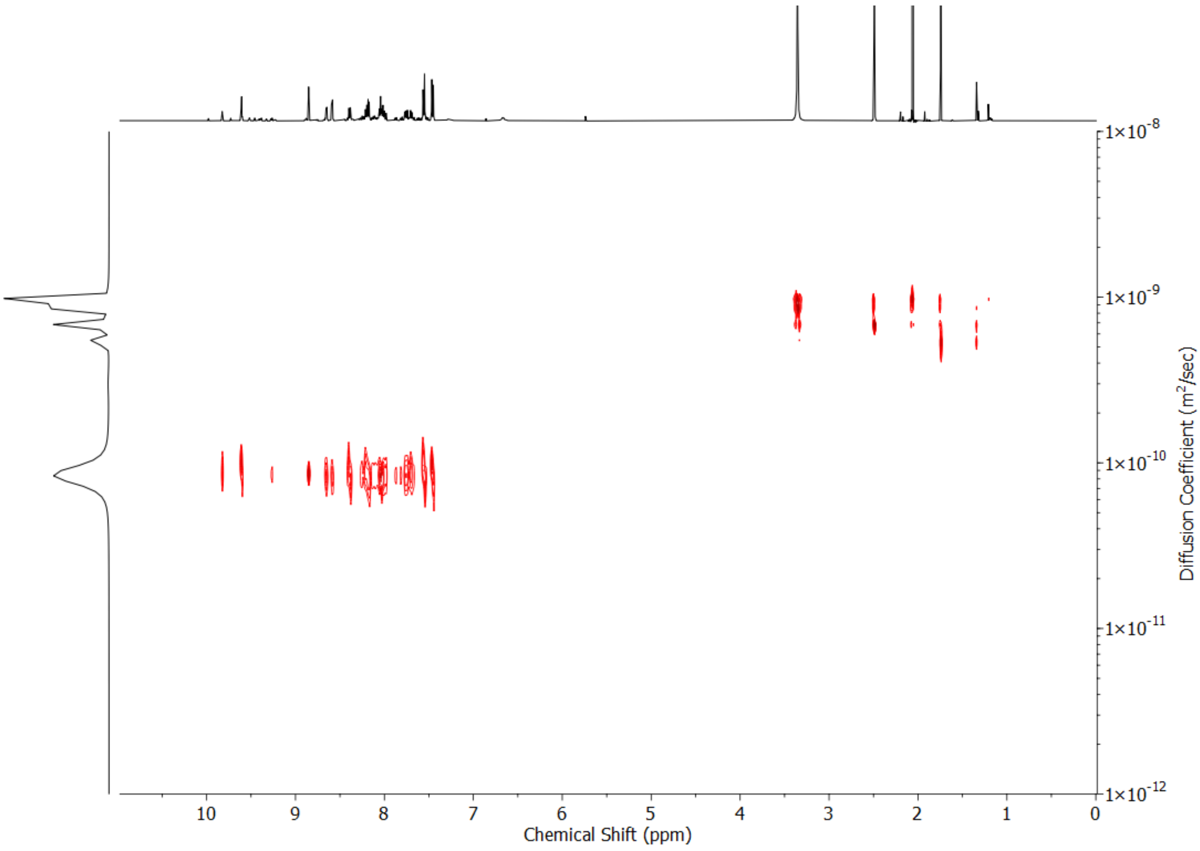

Figure S99 DOSY NMR (d_6-DMSO, 500 MHz) of $[Pd_2(\mathbf{4B3})_4](BF_4)_4$.

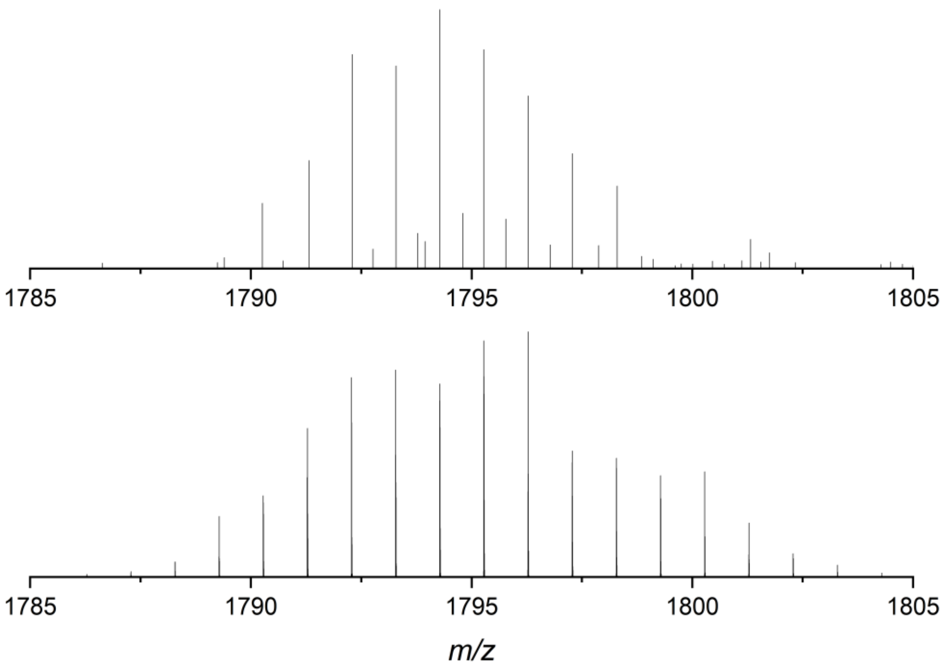

Figure S100 Observed (top) and calculated (bottom) isotopic patterns for {[Pd_2(**4B3**)$_4$](BF_4)$_3$}$^+$.

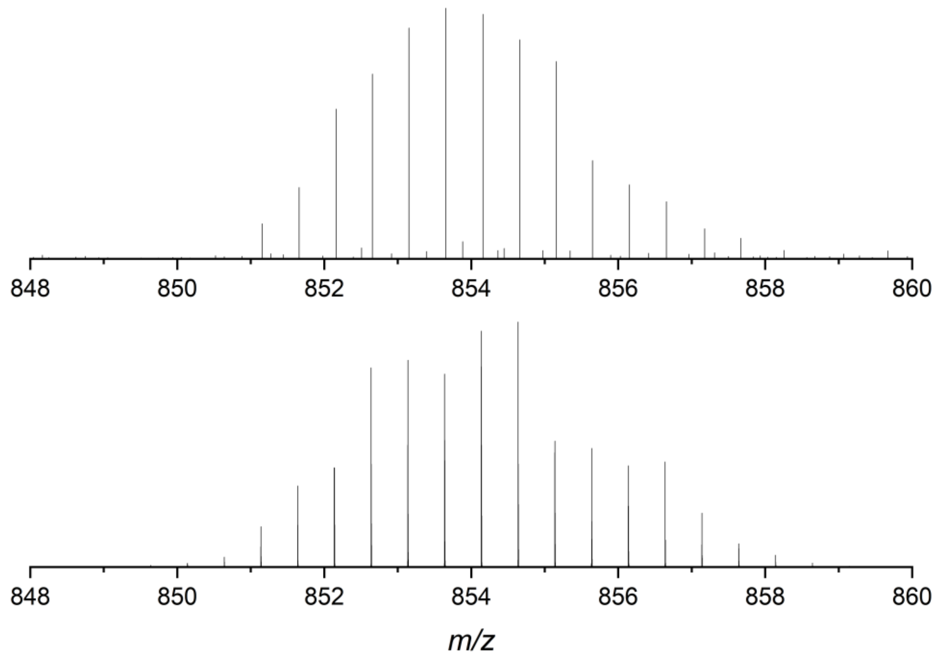

Figure S101 Observed (top) and calculated (bottom) isotopic patterns for {[Pd_2(**4B3**)$_4$](BF_4)$_2$}$^{2+}$.

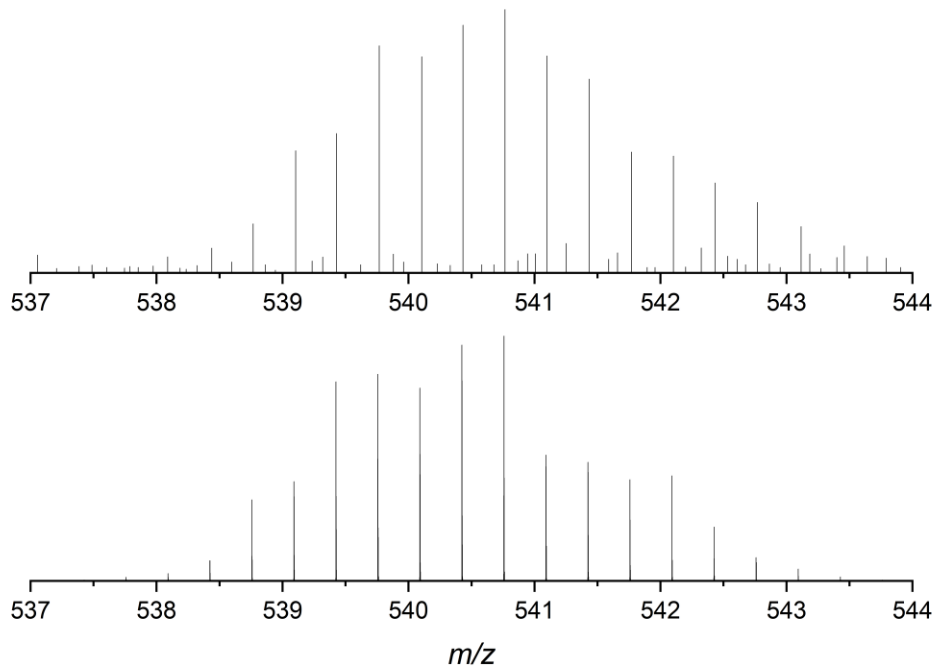

Figure S102 Observed (top) and calculated (bottom) isotopic patterns for $\{[Pd_2(\mathbf{4B3})_4](BF_4)\}^{3+}$.

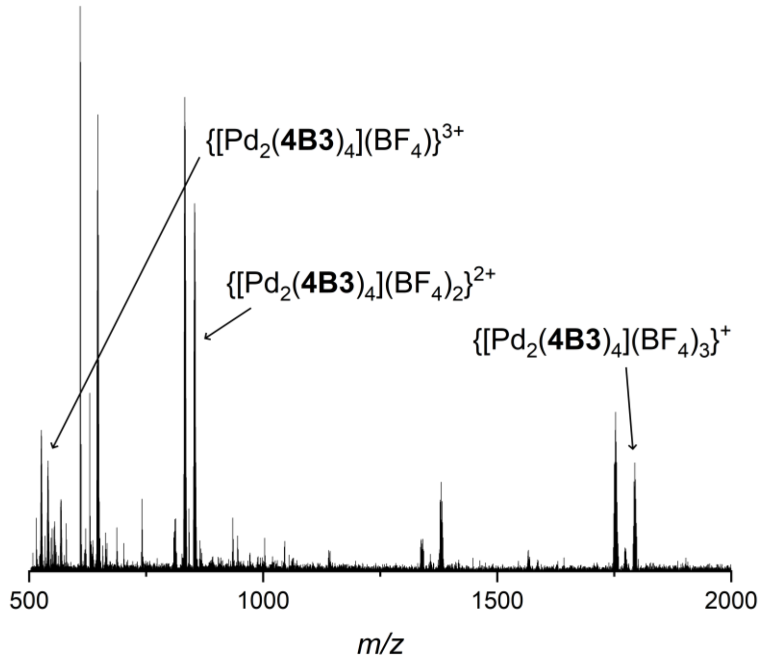

Figure S103 ESI-MS of $[Pd_2(\mathbf{4B3})_4](BF_4)_4$.

Synthesis of $[Pd_2(4B1)_4](BF_4)_4$

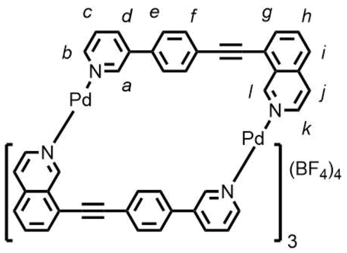

4B1 (9.2 mg, 0.030 mmol) and $[Pd(CH_3CN)_4](BF_4)_2$ (6.7 mg, 0.015 mmol) were sonicated in d_6-DMSO (0.75 mL) until all solids were dissolved. After heating at 50 °C for 2 h, quantitative conversion to $[Pd_2(\mathbf{4B1})_4](BF_4)_4$ was observed by ^{1}H NMR. ^{1}H NMR (400 MHz, d_6-DMSO) δ: 10.09 (s, 4H, H_l), 9.71 (d, *J* = 6.6 Hz, 4H, H_k), 9.67 (s, 4H, H_a), 9.35 (dd, *J* = 5.8, 1.3 Hz, 4H, H_b), 8.52 (d, *J* = 6.5 Hz, 4H, H_j), 8.47 (app. dt, *J* = 6.6, 1.1 Hz, 4H, H_d), 8.27 (d, *J* = 8.4 Hz, 4H, H_i), 8.12 (dd, *J* = 7.2, 1.2 Hz, 4H, H_g), 8.05 (dd, *J* = 8.2, 7.2 Hz, 4H, H_h), 7.94 (d, *J* = 8.2 Hz, 8H, H_e), 7.85 (d, *J* = 8.2 Hz, 8H, H_f), 7.80 (dd, *J* = 8.0, 5.7 Hz, 4H, H_c). Diffusion coefficient (500 MHz, d_6-DMSO) *D*: 1.03×10^{-10} m^2s^{-1}. ^{13}C NMR (101 MHz, d_6-DMSO) δ: 153.4, 150.8, 146.9, 142.7, 139.4, 139.0, 136.4, 135.9, 134.6, 134.0, 133.1, 128.3, 127.9, 127.7, 127.6, 125.4, 122.9, 120.2, 95.4, 86.8. ESI-MS m/z = 1697.29 $\{[Pd_2(\mathbf{4B1})_4](BF_4)_3\}^+$ calc. 1697.28; 805.16 $\{[Pd_2(\mathbf{4B1})_4](BF_4)_2\}^{2+}$ calc. 805.14.

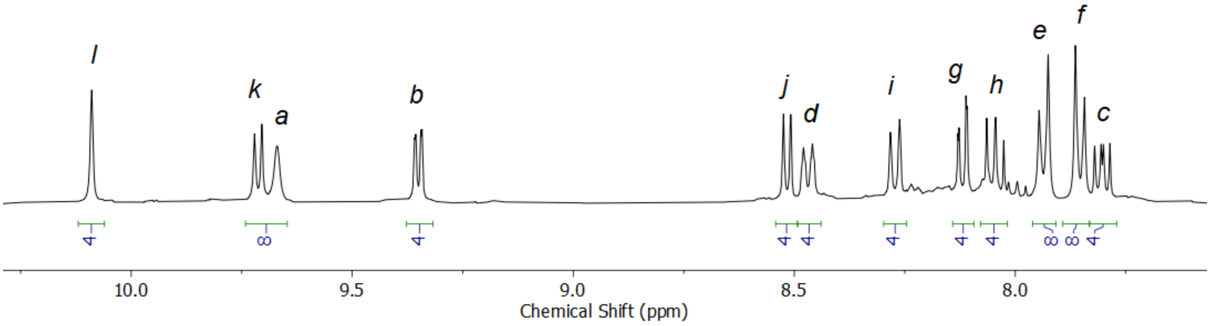

Figure S104 ^{1}H NMR (d_6-DMSO, 400 MHz) of $[Pd_2(\mathbf{4B1})_4](BF_4)_4$.

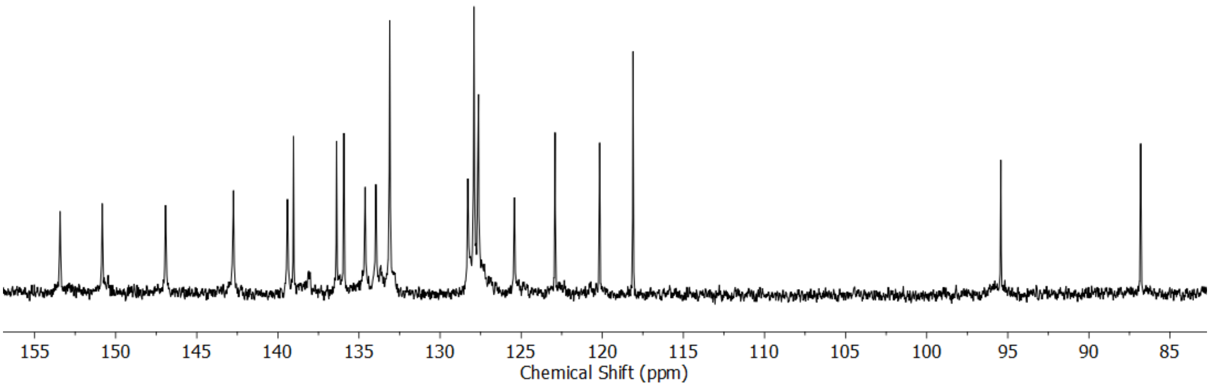

Figure S105 ^{13}C NMR (d_6-DMSO, 101 MHz) of $[Pd_2(\mathbf{4B1})_4](BF_4)_4$.

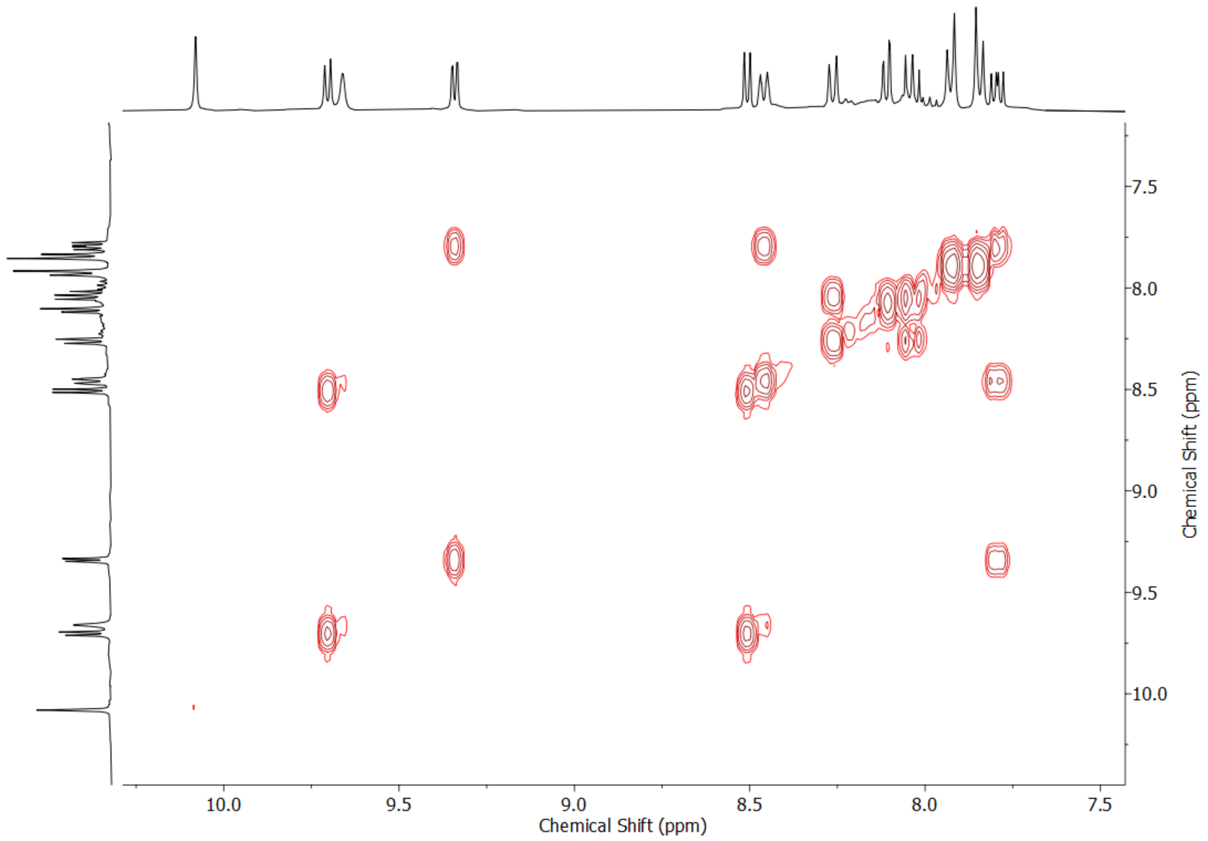

Figure S106 COSY NMR (d_6-DMSO) of $[Pd_2(\textbf{4B1})_4](BF_4)_4$.

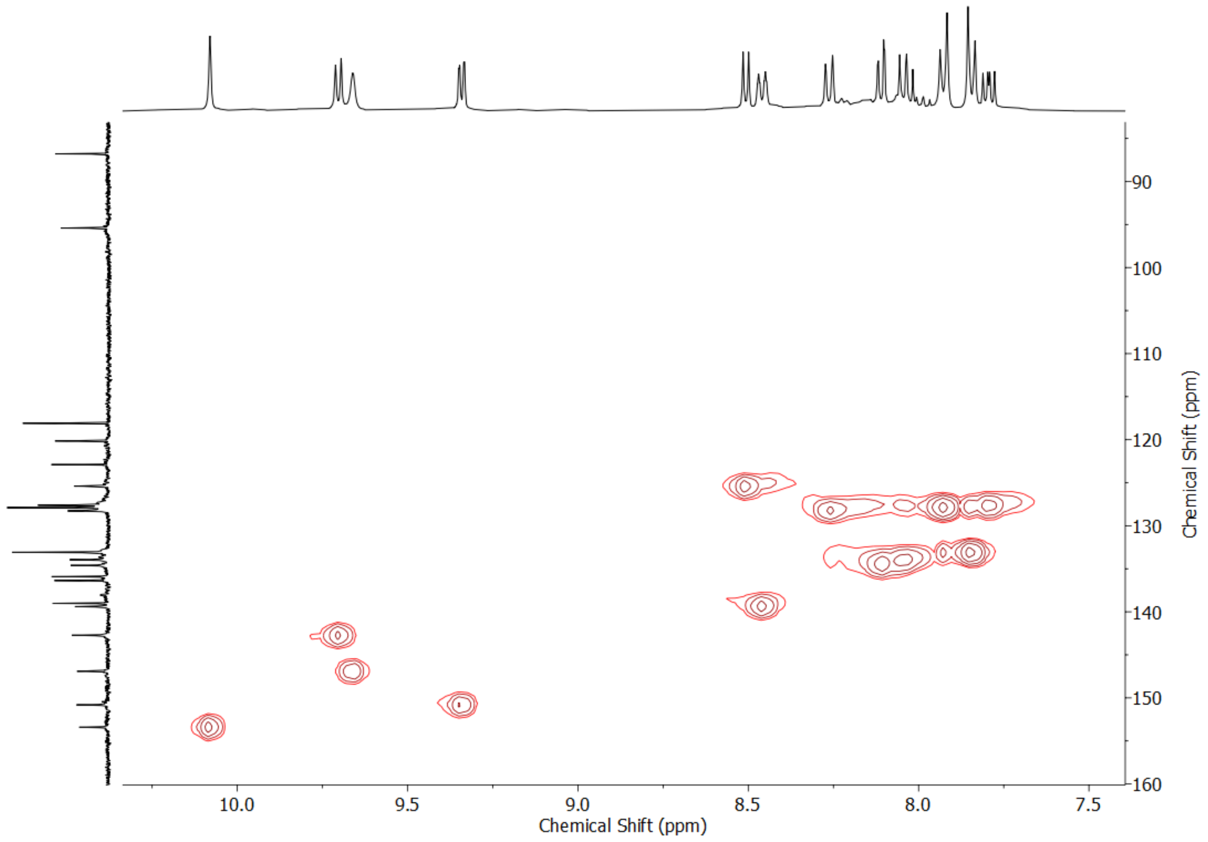

Figure S107 HSQC NMR (d_6-DMSO) of $[Pd_2(\textbf{4B1})_4](BF_4)_4$.

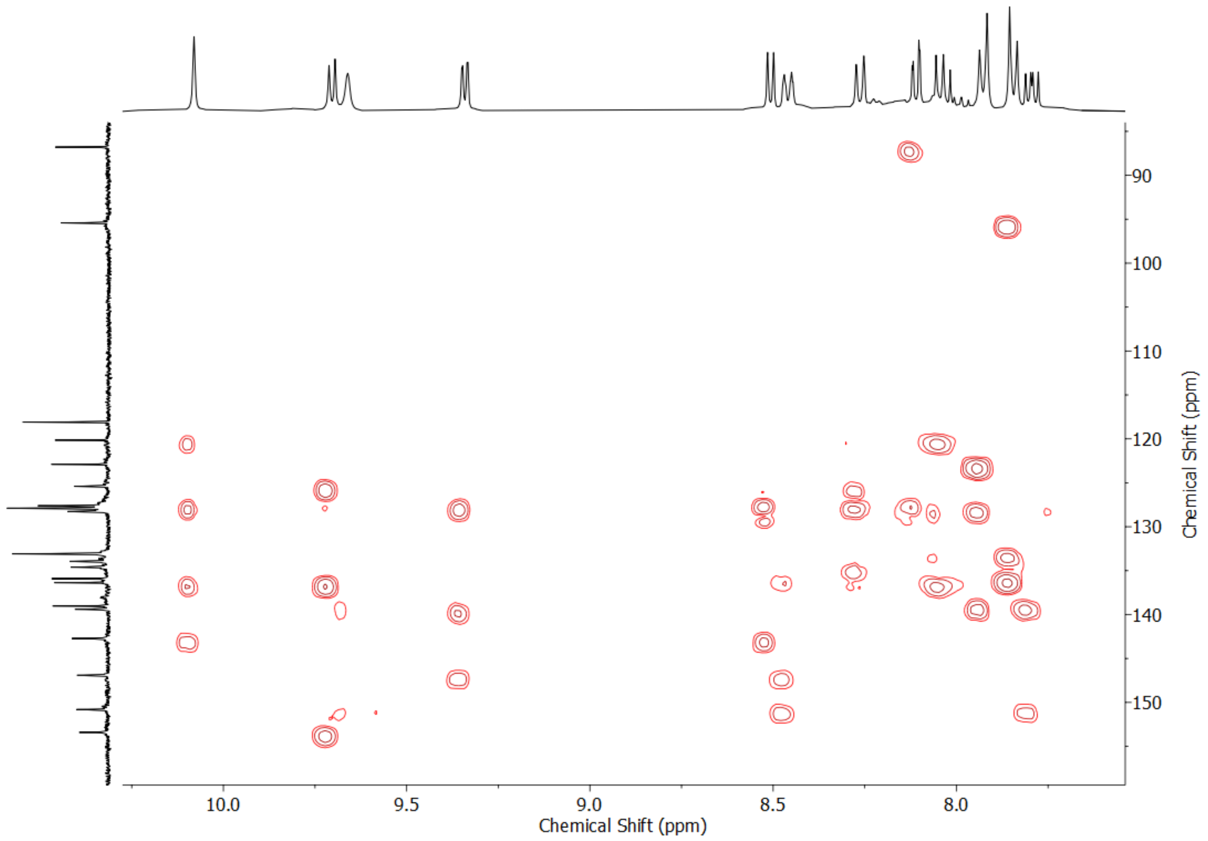

Figure S108 HMBC NMR (d_6-DMSO) of $[Pd_2(\mathbf{4B1})_4](BF_4)_4$.

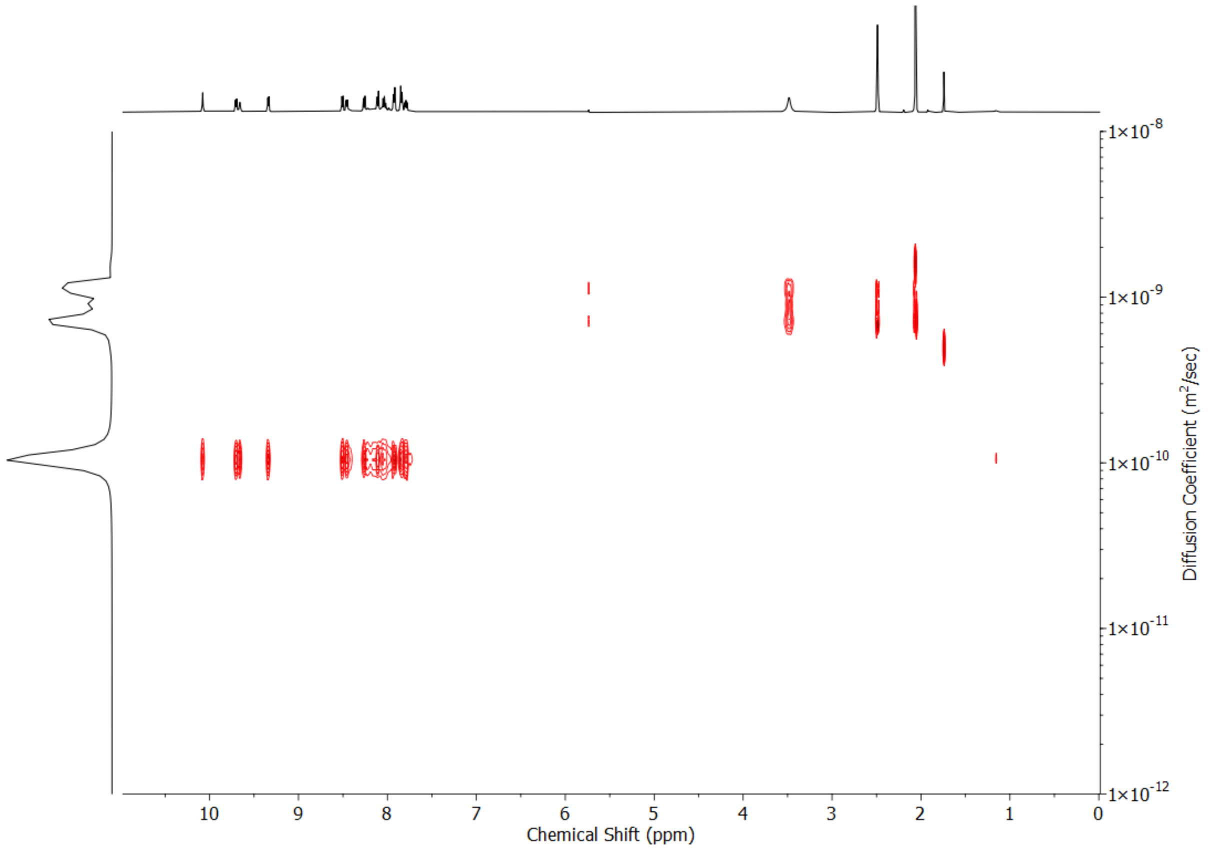

Figure S109 DOSY NMR (d_6-DMSO, 500 MHz) of $[Pd_2(\mathbf{4B1})_4](BF_4)_4$.

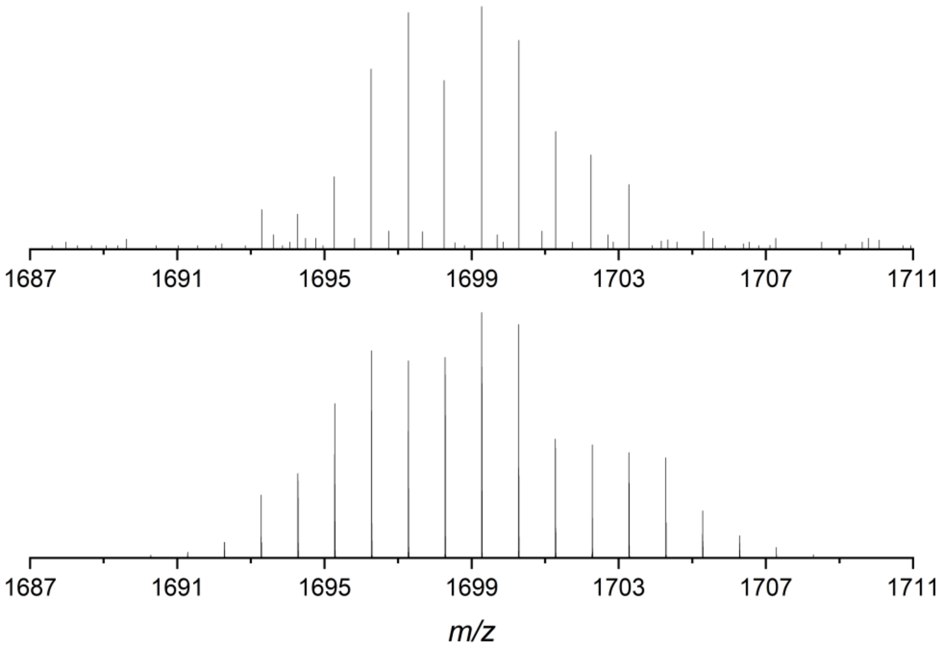

Figure S110 Observed (top) and calculated (bottom) isotopic patterns for {[Pd_2(**4B1**)$_4$](BF_4)$_3$}$^+$.

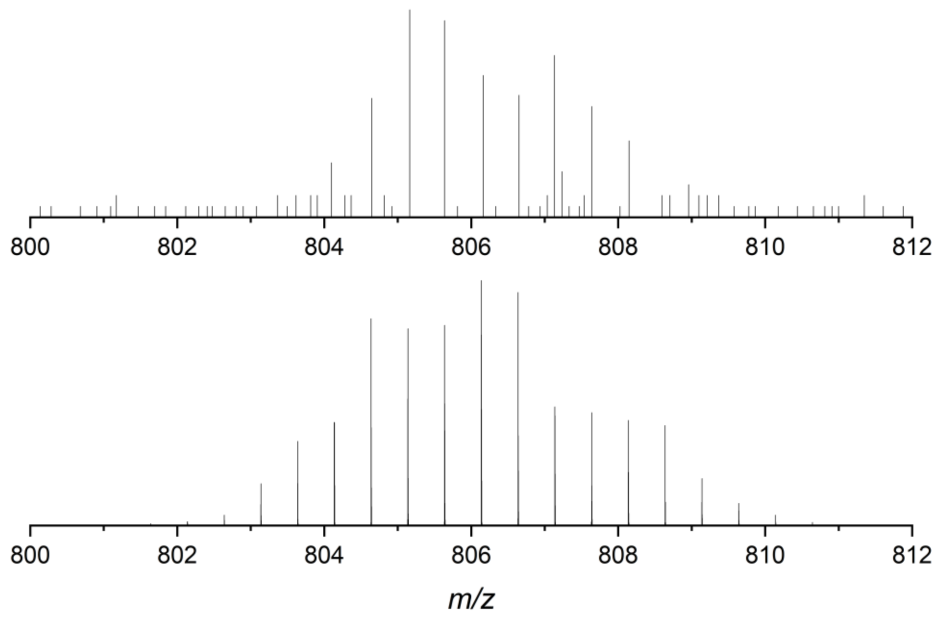

Figure S111 Observed (top) and calculated (bottom) isotopic patterns for {[Pd_2(**4B1**)$_4$](BF_4)$_2$}$^{2+}$.

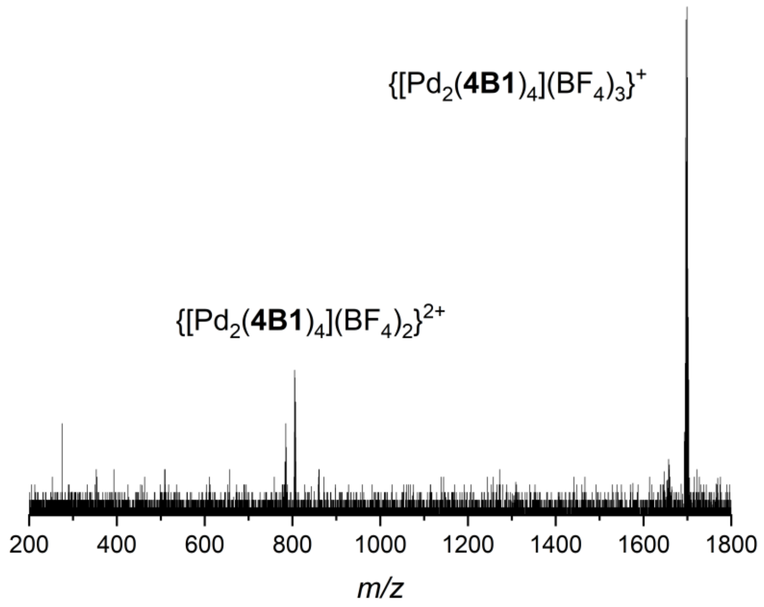

Figure S112 ESI-MS of $[Pd_2($**4B1**$)_4](BF_4)_4$.

Synthesis of $[Pd_2(5B4)_4](BF_4)_4$

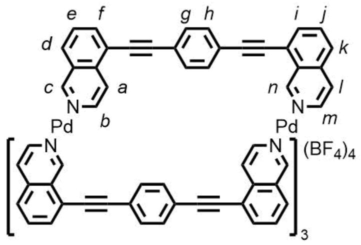

5B4 (5.7 mg, 0.015 mmol) and $[Pd(CH_3CN)_4](BF_4)_2$ (3.3 mg, 0.0075 mmol) were sonicated in d_6-DMSO (0.75 mL) until all solids were dissolved. Quantitative conversion to $[Pd_2(\mathbf{5B4})_4](BF_4)_4$ was observed by ^{1}H NMR. ^{1}H NMR (500 MHz, d_6-DMSO) δ: 10.04-10.02 (m, 8H, H_b, H_n), 9.89 (s, 4H, H_c), 9.55 (d, J = 6.6 Hz, 4H, H_m), 8.74 (d, J = 6.6 Hz, 4H, H_a), 8.47 (d, J = 6.5 Hz, 4H, H_l), 8.25-8.23 (m, 8H, H_d, H_k), 8.18 (dd, J = 7.1, 1.1 Hz, 4H, H_f/H_i), 8.14 (d, J = 8.2 Hz, 8H, H_g/H_h), 8.03-7.99 (m, 8H, H_f/H_i, H_e/H_j), 7.96 (d, J = 8.2 Hz, 8H, H_g/H_h), 7.82 (dd, J = 8.4, 7.1 Hz, 4H, H_e/H_j). Diffusion coefficient (500 MHz, d_6-DMSO) D: 9.79 × 10^{-11} m^2s^{-1}. ^{13}C NMR (126 MHz, d_6-DMSO) δ: 157.6, 153.8, 142.8, 141.7, 137.3, 136.4, 135.5, 133.9, 133.8, 132.4, 132.1, 130.1, 129.7, 128.2, 127.9, 127.4, 125.2, 122.8, 122.5, 122.1, 120.4, 119.0, 96.4, 87.2, 87.0. ESI-MS m/z = 953.18 $\{[Pd_2(\mathbf{5B4})_4](BF_4)_2\}^{2+}$ calc. 953.17.

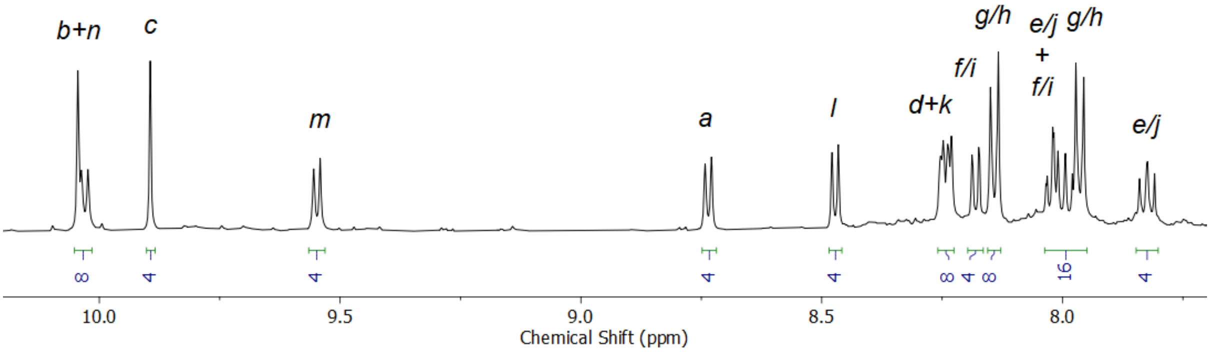

Figure S113 ^{1}H NMR (d_6-DMSO, 500 MHz) of $[Pd_2(\mathbf{5B4})_4](BF_4)_4$.

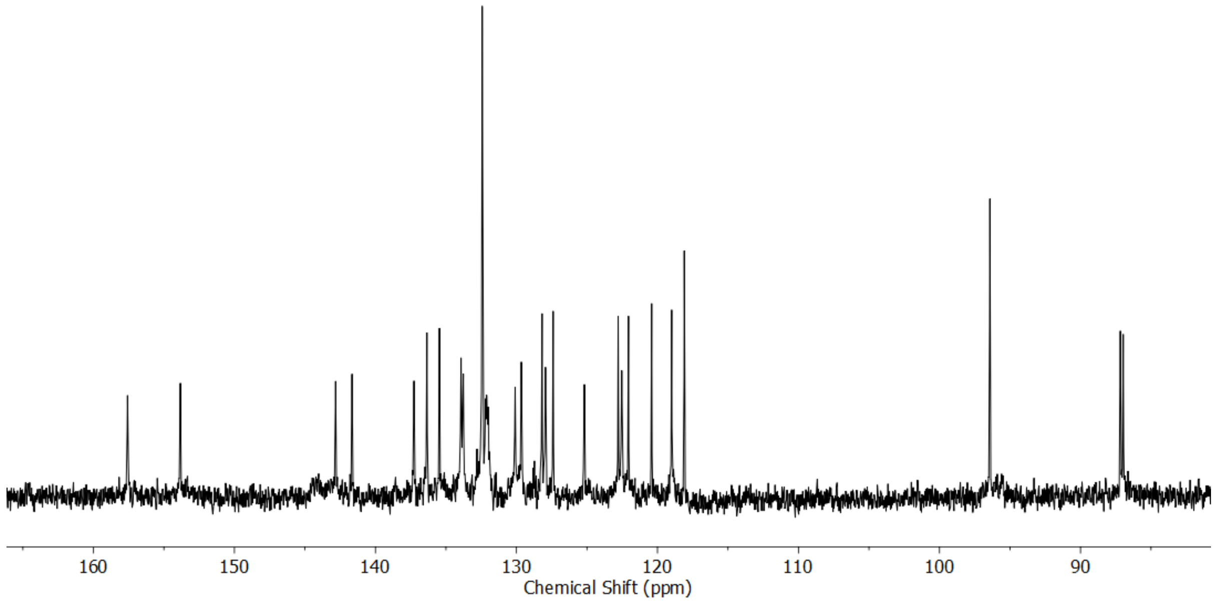

Figure S114 ^{13}C NMR (d_6-DMSO, 126 MHz) of $[Pd_2(\mathbf{5B4})_4](BF_4)_4$.

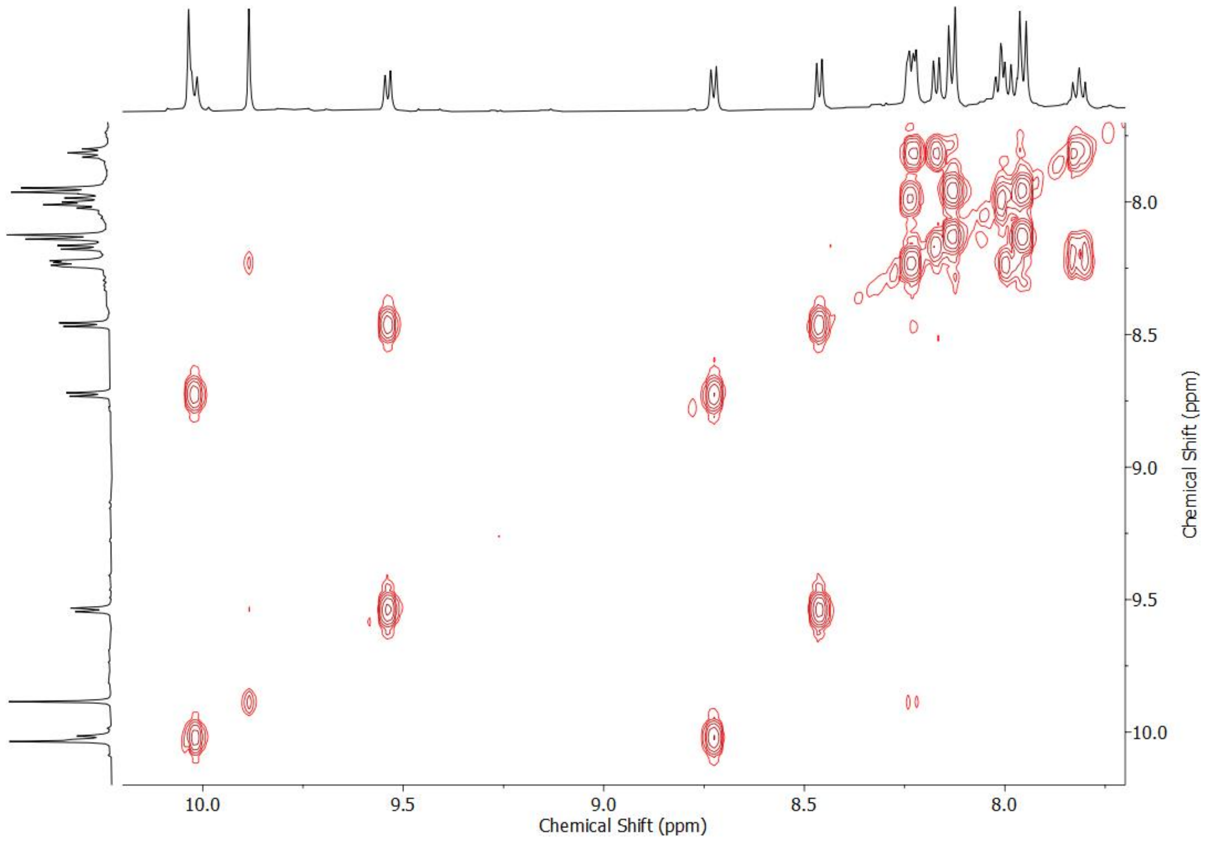

Figure S115 COSY NMR (d_6-DMSO) of $[Pd_2(\mathbf{5B4})_4](BF_4)_4$.

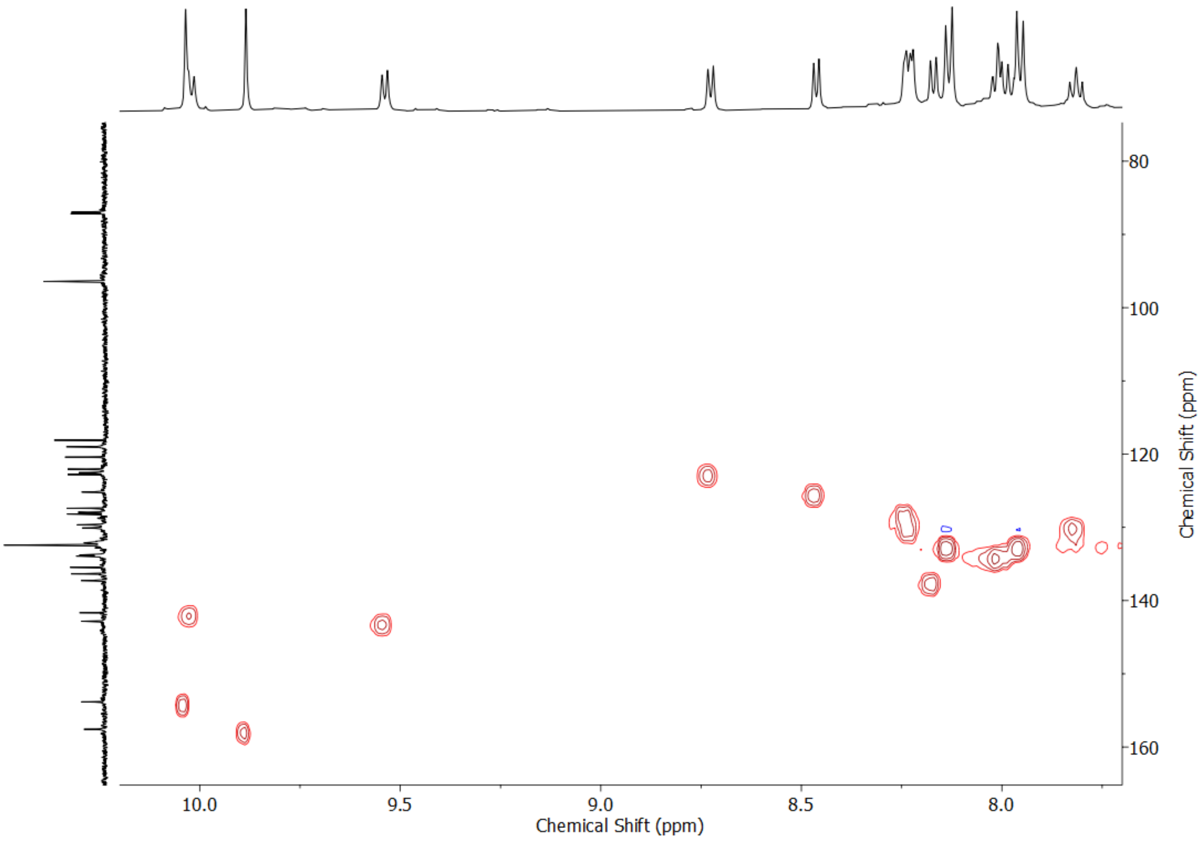

Figure S116 HSQC NMR (d_6-DMSO) of $[Pd_2($**5B4**$)_4](BF_4)_4$.

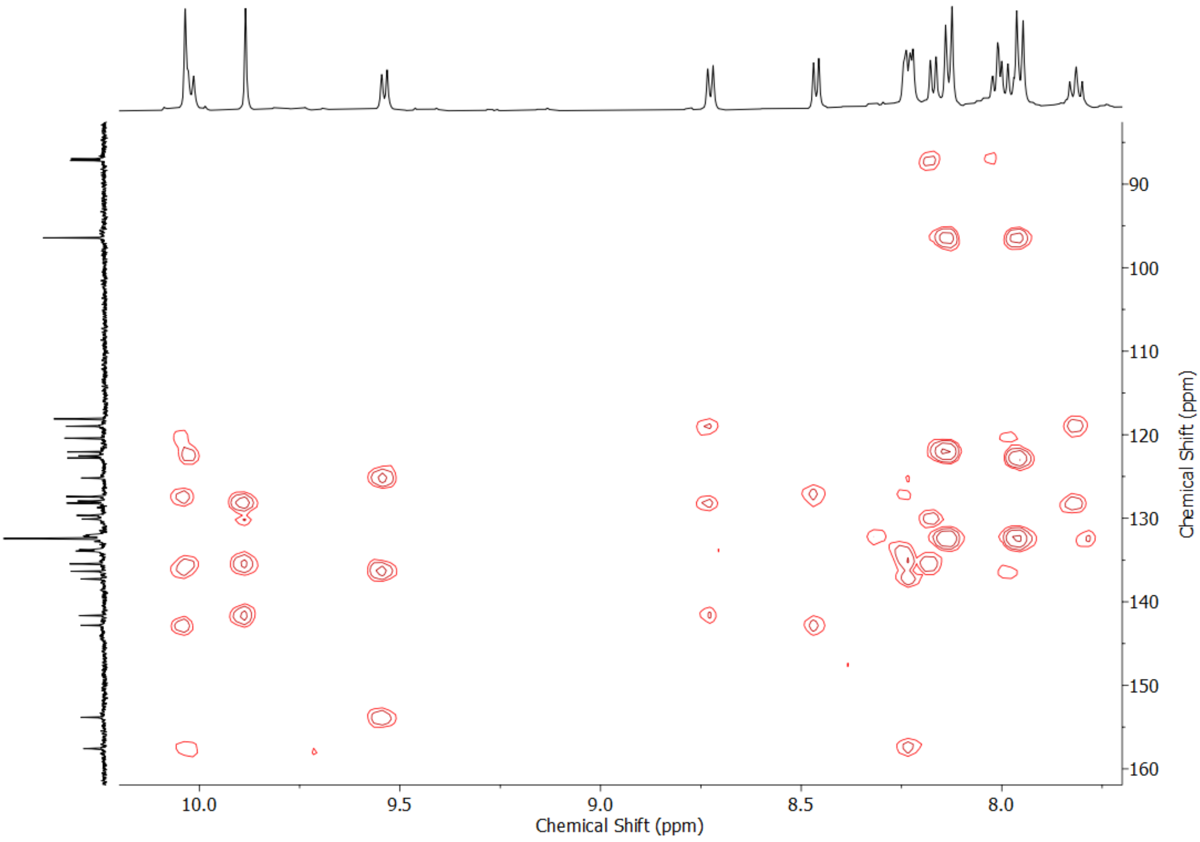

Figure S117 HMBC NMR (d_6-DMSO) of $[Pd_2($**5B4**$)_4](BF_4)_4$.

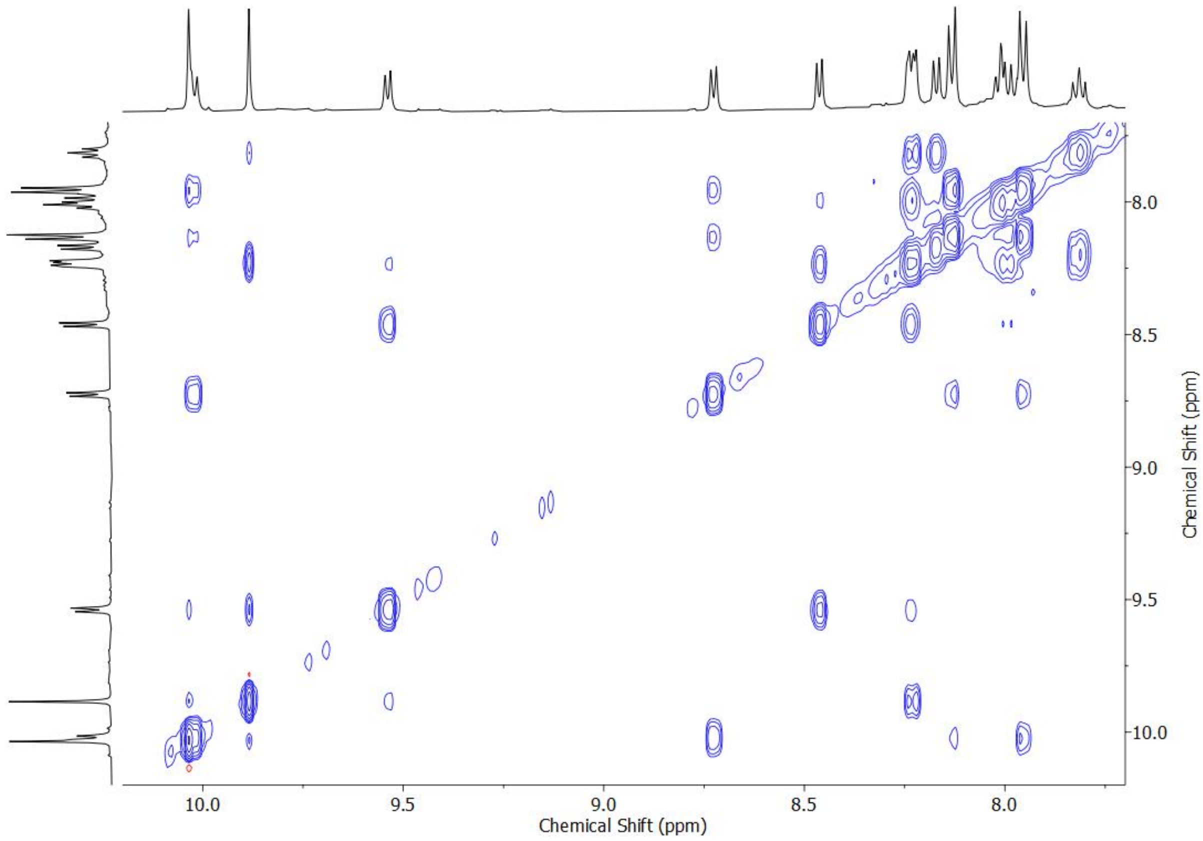

Figure S118 NOESY NMR (d_6-DMSO, 500 MHz) of $[Pd_2($**5B4**$)_4](BF_4)_4$.

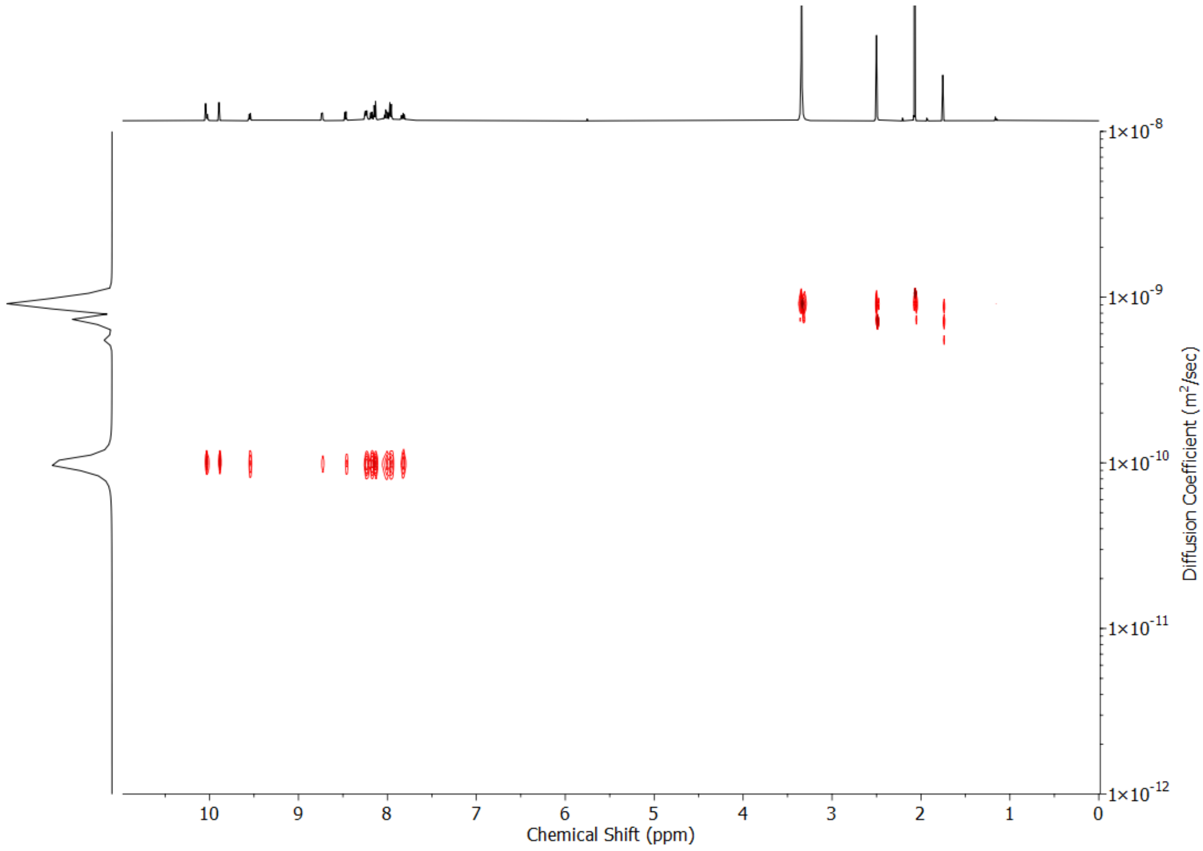

Figure S119 DOSY NMR (d_6-DMSO, 500 MHz) of $[Pd_2($**5B4**$)_4](BF_4)_4$.

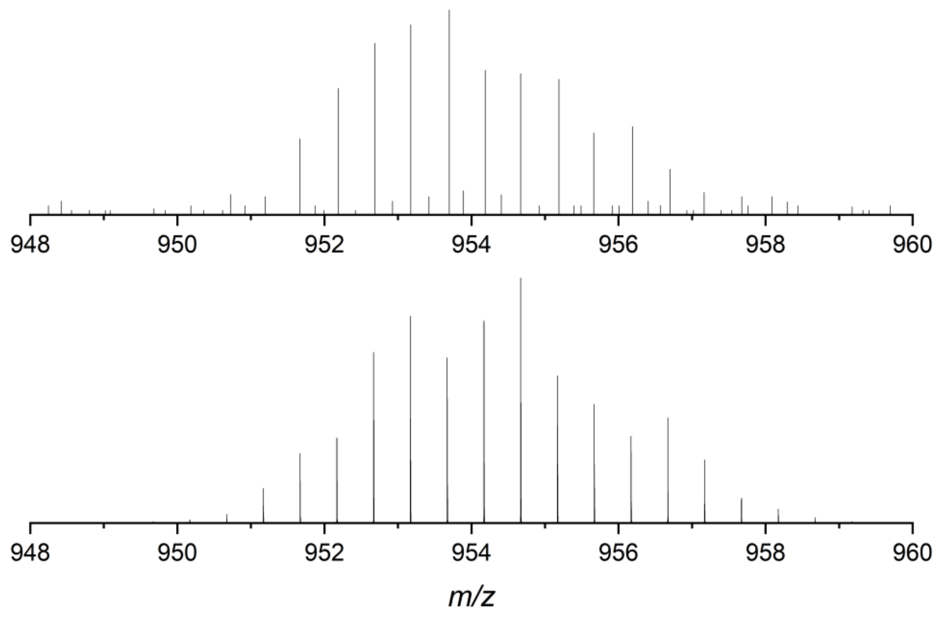

Figure S120 Observed (top) and calculated (bottom) isotopic patterns for $\{[Pd_2(\mathbf{5B4})_4](BF_4)_2\}^{2+}$.

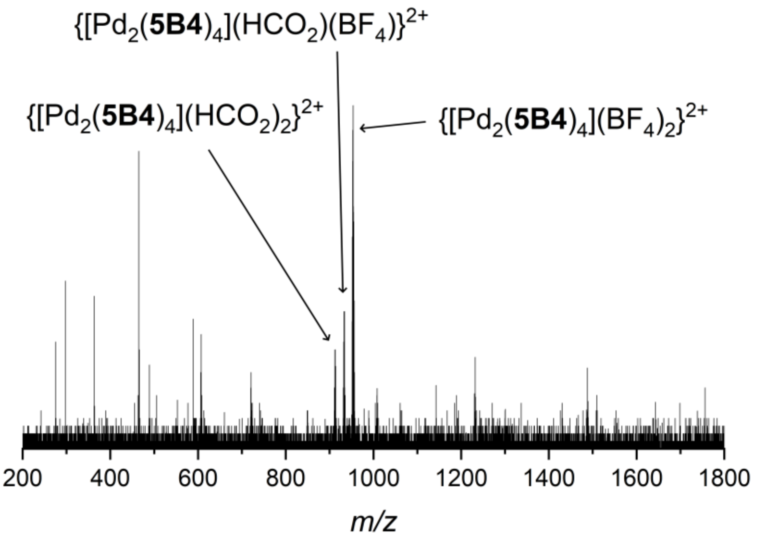

Figure S121 ESI-MS of $[Pd_2(\mathbf{5B4})_4](BF_4)_4$.

9. Solvodynamic Radii Calculations

Solvodynamic radii were calculated using a variation of the Stokes-Einstein equation:

$$R_S = \frac{k_B T}{6\pi\eta D}$$

Where R_S is the solvodynamic radius (m)

k_B is the Boltzmann constant (1.38×10^{-23} J K^{-1})

T is the temperature (K)

η is the solvent viscosity (2.180×10^{-3} kg $s^{-1}m^{-1}$ for d_6-DMSO)[30]

D is the diffusion coefficient (m^2s^{-1})

Compound	*D* (m^2s^{-1})	R_S (Å)
$[Pd_2($**5A1**$)_4](BF_4)_4$	1.20×10^{-10}	8.4
$[Pd_2($**5A3**$)_4](BF_4)_4$	9.61×10^{-11}	10.4
$[Pd_2($**4B1**$)_4](BF_4)_4$	1.03×10^{-10}	9.7
$[Pd_2($**4B3**$)_4](BF_4)_4$	8.33×10^{-11}	12.0
$[Pd_2($**5B4**$)_4](BF_4)_4$	9.79×10^{-11}	10.2

10. References

[1] L. Turcani, E. Berardo and K. E. Jelfs, *J. Comput. Chem.*, 2018, **39**, 1931-1942.
[2] L. Turcani, A. Tarzia, F. Szczypiński and K. E. Jelfs, *J. Chem. Phys*, 2021, **154**, 214102.
[3] E. Berardo, L. Turcani, M. Miklitz and K. E. Jelfs, *Chem. Sci.*, 2018, **9**, 8513-8527.
[4] L. Turcani, R. L. Greenaway and K. E. Jelfs, *Chem. Mater.*, 2019, **31**, 714-727.
[5] S. Riniker and G. A. Landrum, *J. Chem. Inf. Model.*, 2015, **55**, 2562-2574.
[6] G. A. Landrum, RDKit: Open-Source Cheminformatics., http://www.rdkit.org/, (accessed March 1, 2020).
[7] C. Bannwarth, S. Ehlert and S. Grimme, *J. Chem. Theory Comput.*, 2019, **15**, 1652-1671.
[8] D. E. Coupry, M. A. Addicoat and T. Heine, *J. Chem. Theory Comput.*, 2016, **12**, 5215-5225.
[9] M. A. Addicoat, N. Vankova, I. F. Akter and T. Heine, *J. Chem. Theory Comput.*, 2014, **10**, 880-891.
[10] J. D. Gale, *J. Chem. Soc. Faraday Trans.*, 1997, **93**, 629-637.
[11] J. D. Gale and A. L. Rohl, *Mol. Simul.*, 2003, **29**, 291-341.
[12] M. Bursch, H. Neugebauer and S. Grimme, *Angew. Chem. Int. Ed.*, 2019, **58**, 11078-11087.
[13] A. K. Rappé, C. J. Casewit, K. S. Colwell, W. A. Goddard III and W. M. Skiff, *J. Am. Chem. Soc.*, 1992, **114**, 10024-10035.
[14] C. Bannwarth, E. Caldeweyher, S. Ehlert, A. Hansen, P. Pracht, J. Seibert, S. Spicher and S. Grimme, *WIRES Comput. Mol. Sci.*, 2021, **11**, e1493.
[15] J. E. M. Lewis, A. Tarzia, A. J. P. White and K. E. Jelfs, *Chem. Sci.*, 2020, **11**, 677-683.
[16] Gaussian 16, Revision C.01, M. J. Frisch, G. W. Trucks, H. B. Schlegel, G. E. Scuseria, M. A. Robb, J. R. Cheeseman, G. Scalmani, V. Barone, G. A. Petersson, H. Nakatsuji, X. Li, M. Caricato, A. V. Marenich, J. Bloino, B. G. Janesko, R. Gomperts, B. Mennucci, H. P. Hratchian, J. V. Ortiz, A. F. Izmaylov, J. L. Sonnenberg, D. Williams-Young, F. Ding, F. Lipparini, F. Egidi, J. Goings, B. Peng, A. Petrone, T. Henderson, D. Ranasinghe, V. G. Zakrzewski, J. Gao, N. Rega, G. Zheng, W. Liang, M. Hada, M. Ehara, K. Toyota, R. Fukuda, J. Hasegawa, M. Ishida, T. Nakajima, Y. Honda, O. Kitao, H. Nakai, T. Vreven, K. Throssell, J. A. Montgomery, Jr., J. E. Peralta, F. Ogliaro, M. J. Bearpark, J. J. Heyd, E. N. Brothers, K. N. Kudin, V. N. Staroverov, T. A. Keith, R. Kobayashi, J. Normand, K. Raghavachari, A. P. Rendell, J. C. Burant, S. S. Iyengar, J. Tomasi, M. Cossi, J. M. Millam, M. Klene, C. Adamo, R. Cammi, J. W. Ochterski, R. L. Martin, K. Morokuma, O. Farkas, J. B. Foresman, and D. J. Fox, Gaussian, Inc., Wallingford CT, 2016.
[17] C. Adamo and V. Barone, *J. Chem. Phys.*, 1999, **110**, 6158-6170.
[18] F. Weigend and R. Ahlrichs, *Phys. Chem. Chem. Phys.*, 2005, **7**, 3297-3305.
[19] F. Weigend, *Phys. Chem. Chem. Phys.*, 2006, **8**, 1057-1065.
[20] S. Grimme, S. Ehrlich and L. Goerigk, *J. Comput. Chem.*, 2011, **32**, 1456-1465.
[21] J. Tomasi, B. Mennucci and R. Cammi, *Chem. Rev.*, 2005, **105**, 2999-3094.
[22] T. A. Young, R. Gheorghe and F. Duarte, *J. Chem. Inf. Model.*, 2020, **60**, 3546-3557.
[23] J. G. Brandenburg, C. Bannwarth, A. Hansen and S. J. Grimme, *J. Chem. Phys.*, 2018, **148**, 064104.
[24] F. Neese, *WIRES Comput. Mol. Sci.*, 2018, **8**, e1327.
[25] N. E. R. Zimmerman and A. Jain, *RSC Adv.*, 2020, **10**, 6063-6081
[26] S. P. Ong, W. D. Richards, A. Jain, G. Hautier, M. Kocher, S. Cholia, D. Gunter, V. L. Chevrier, K. A. Persson and G. Ceder, *Comput. Mater. Sci.*, 2013, **68**, 314-319

[27] M. Miklitz, K. E. Jelfs, *J. Chem. Inf. Model.*, 2018, **58**, 2387-2391.
[28] S. A. Gamage, J. A. Spicer, G. W. Rewcastle, J. Milton, S. Sohal, W. Dangerfield, P. Mistry, N. Vicker, P. A. Charlton and W. A. Denny, *J. Med. Chem.*, 2002, **45**, 740-743.
[29] L. M. Kirkpatrick, N. R. Vinueza, B. J. Jankiewicz, V. A. Gallardo, E. F. Archibold, J. J. Nash and H. I. Kenttämaa, *Chem. Eur. J.*, 2013, **19**, 9022-9033.
[30] M. Holz, X. Mao, D. Seiferling and A. Sacco, *J. Chem. Phys.*, 1996, **104**, 669-679.
